# Assessment of cholecystokinin 2 receptor (CCK2R) in neoplastic tissue

**DOI:** 10.18632/oncotarget.7522

**Published:** 2016-02-20

**Authors:** Jyoti Roy, Karson S. Putt, Domenico Coppola, Marino E. Leon, Farah K. Khalil, Barbara A. Centeno, Noel Clark, Valerie E. Stark, David L. Morse, Philip S. Low

**Affiliations:** ^1^ Center for Drug Discovery, Purdue University, West Lafayette IN 47907 USA; ^2^ Department of Chemistry, Purdue University, West Lafayette IN 47907 USA; ^3^ Department of Anatomic Pathology, H. Lee Moffitt Cancer Center, Tampa FL 33612 USA; ^4^ Tissue Core, H. Lee Moffitt Cancer Center, Tampa FL 33612 USA; ^5^ Department of Cancer Imaging and Metabolism, Imaging and Technology Center of Excellence, H. Lee Moffitt Cancer Center, Tampa FL 33612 USA

**Keywords:** cholecystokinin 2 receptor, CCK2R, CCKBR, gastrin receptor

## Abstract

The expression of cholecystokinin 2 receptor (CCK2R, CCKBR or gastrin receptor) has been reported on a diverse range of cancers such as colorectal, liver, lung, pancreatic, ovarian, stomach, thyroid and numerous neuroendocrine/carcinoid tumors. Some cancers of the colorectum, lung, pancreas and thyroid have been shown to overexpress CCK2R in relation to normal matched tissues of the same organ. This reported overexpression has led to the development of a number of CCK2R-ligand targeted imaging and therapeutic agents. However, no comprehensive study comparing the expression of CCK2R in multiple cancers to multiple normal tissues has been performed. Herein, we report the immunohistochemical analysis of cancer samples from gastrointestinal stromal tumor (GIST), hepatocellular carcinoma (HCC), non-small cell lung cancer (NSCLC), pancreatic adenocarcinoma, and thyroid cancer against multiple normal tissue samples from esophagus, liver, lung, pancreas, stomach, spleen and thyroid. These results show that CCK2R expression is present in nearly all cancer and normal samples tested and that none of the cancer samples had expression that was statistically greater than that of all of the normal samples.

## INTRODUCTION

Cholecystokinin 2 receptor (CCK2R), formerly known as CCKBR or gastrin receptor is a G protein-coupled receptor originally identified in the gastrointestinal tract and central nervous system [1, 2]. As early as the 1960s, gastrin-binding receptors were identified on malignant tissues [3]. Since that time, the presence of CCK2R has been reported on many cancers [4–17], especially those of neuroendocrine origin [2, 4–6, 18–22]. Reports in the literature show widely varying percentages of cancers expressing CCK2R (see Table 1 for summary), with gastric adenocarcinomas [23], medullary thyroid carcinoma [4, 20], colorectal carcinoma [7, 8], small cell lung carcinoma [6], and pancreatic carcinoma [16] overexpressing the receptor when compared to matched normal tissues.

**Table 1:** Percentages of patient cancer samples testing positive for CCK2R expression by cancer subtype

	Tumor Type	IHC	IHC	From Literature ^(reference)^ Ligand Binding	PCR
**Non-neuroendocrine tumors**	Astrocytomas				65%^(4)^
	Colorectal		33%^(15)^/39%^(15)^	56%^(11)^	11%^(12)^/38%^(9)^/44%^(7)^/69%^(5)^/100%^(8)^
	Esophageal		16%^(14)^		
	Liver (cholangiocarcinoma)		90%^(17)^		
	Liver (fibrolamellar carcinoma)		90%^(17)^		
	Liver (hepatocellular carcinoma)	98%	91%^(17)^		80%^(5)^
	Lung (non-small cell)	96%		8%^(5)^	6%^(6)^/75%^(5)^
	Ovarian				100%^(4)^
	Pancreatic	100%			57%^(5)^/100%^(10)^
	Stomach		56%^(37)^	25%^(5)^	75% ^(5)^/100%^(13)^
	Thyroid (non-medullary carcinoma)	100%			
**Neuroendocrine / carcinoid tumors**	Bronchial carcinoids			62%^(18)^	
	Carcinoids of the bowels			30%^(18)^/67%^(19)^/88%^(5)^	100%^(5)^
	Carcinoids of the stomach		100%^(22)^	95%^(19)^	
	GIST	100%	61%^(2)^	100%^(5)^	57 %^(2)^/100%^(5)^
	Lung (non-small cell neuroendocrine tumors)	100%		50%^(5)^	
	Lung (small cell)			78%^(5)^	57%^(4)^/89%^(51^/100%^(6)^
	Pancreatic neuroendocrine tumors		95%^(16)^	100%^(5)^/100%^(19)^/100%^(18)^	100%^(5)^
	Paraganglioma			33%^(19)^	
	Thyroid (medullary carcinoma)	100%	60%^(21)^	92%^(20)^	58%^(21)^/92%^(4)^

Literature reports of the expression of CCK2R as determined by immunohistochemistry (IHC), binding of radiolabeled ligands (ligand binding) or presence of mRNA (PCR/northern blot) are summarized. The percentages of samples positive for CCK2R in the literature are shown in the final three columns. The percentages of samples positive for CCK2R in our IHC studies are shown in the first column.

This reported overexpression on malignant cells has led to great interest in developing CCK2R-targeted cancer imaging agents. The endogenous peptide ligand to CCK2R or various peptide analogues have been labeled and used for imaging in a variety of mouse xenograft models with _99m_Tc [24], ^111^In [25–28], ^64^Cu [26], ^68^Ga [26, 27] and several near infrared fluorescent dyes [29, 30]. Moreover, human imaging studies with CCK2R-targeted ^111^In and _99m_Tc have been performed to visualize medullary thyroid cancers; however, efforts to image other cancers have largely been thwarted by high uptake of the targeted imaging agent in visceral organs [19, 31–36]. Furthermore, when a therapeutic CCK2R-targeted beta particle emitting ^90^Y-labeled mini-gastrin was administered to human patients, severe toxicities were observed including the development of renal failure in 25% of patients within 1 to 2 years after therapy [36]. These results raise the question whether expression of CCK2R might be too high in healthy organs to allow imaging of malignancies of the colon, pancreas, liver, lung, and stomach.

In order to better establish the levels and patterns of CCK2R staining in human tissues, a broad set of normal tissues (esophagus, liver, lung, pancreas, stomach, spleen and thyroid) and cancerous tissues (gastrointestinal stromal tumor (GIST), hepatocellular carcinoma (HCC), non-small cell lung cancer (NSCLC), pancreatic adenocarcinoma, and thyroid cancer) were analyzed via immunohistochemistry (IHC) using a monoclonal antibody raised against CCK2R. Herein, we report that CCK2R is present in every cancer type tested; however, expression levels were found to be similar to those in normal tissues except perhaps cancers of the pancreas, where CCK2R expression correlated with tumor size and stage.

## RESULTS

### Antibody and IHC

Monoclonal antibodies were induced against a unique peptide sequence in human CCK2R and four monoclonals that recognized the antigen were further evaluated for specificity and selectivity. While all monoclonals exhibited similar binding patterns, monoclonal 6C10G11 was selected for further use due to its strong staining of CCK2R-transfected cells and low/no staining of CCK2R negative cells. Monoclonal 6C10G11 was used then to perform immunohistochemistry (IHC) on various sections of cancer and normal human tissue. IHC staining was optimized and performed in the H. Lee Moffitt Cancer Center Tissue Core as described in Methods. IHC scoring was performed as described in Methods by pathologists at Moffitt that specialize in each cancer type examined.

### Cancer tissue - GIST

**Figure 1:**
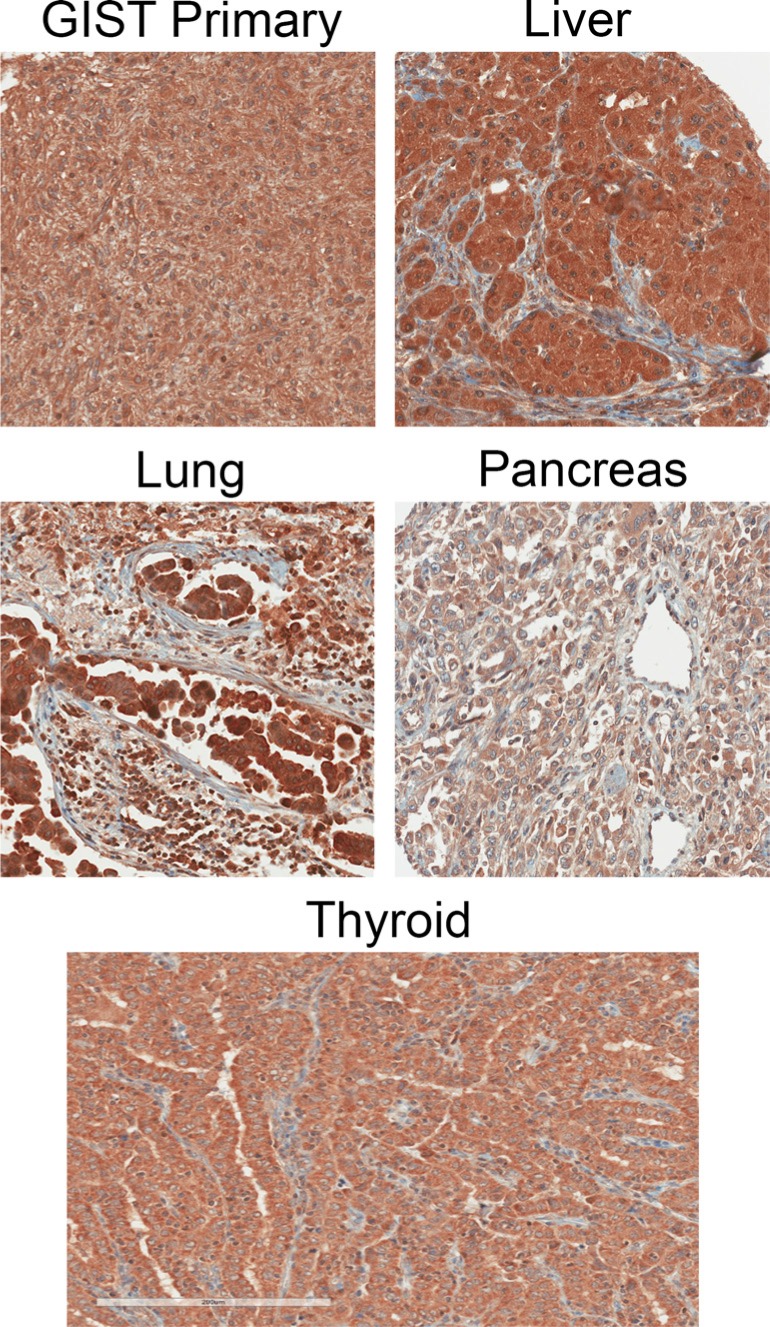
Example IHC images of cancer specimens using the CCK2R specific antibody 6C10G11

**Figure 2:**
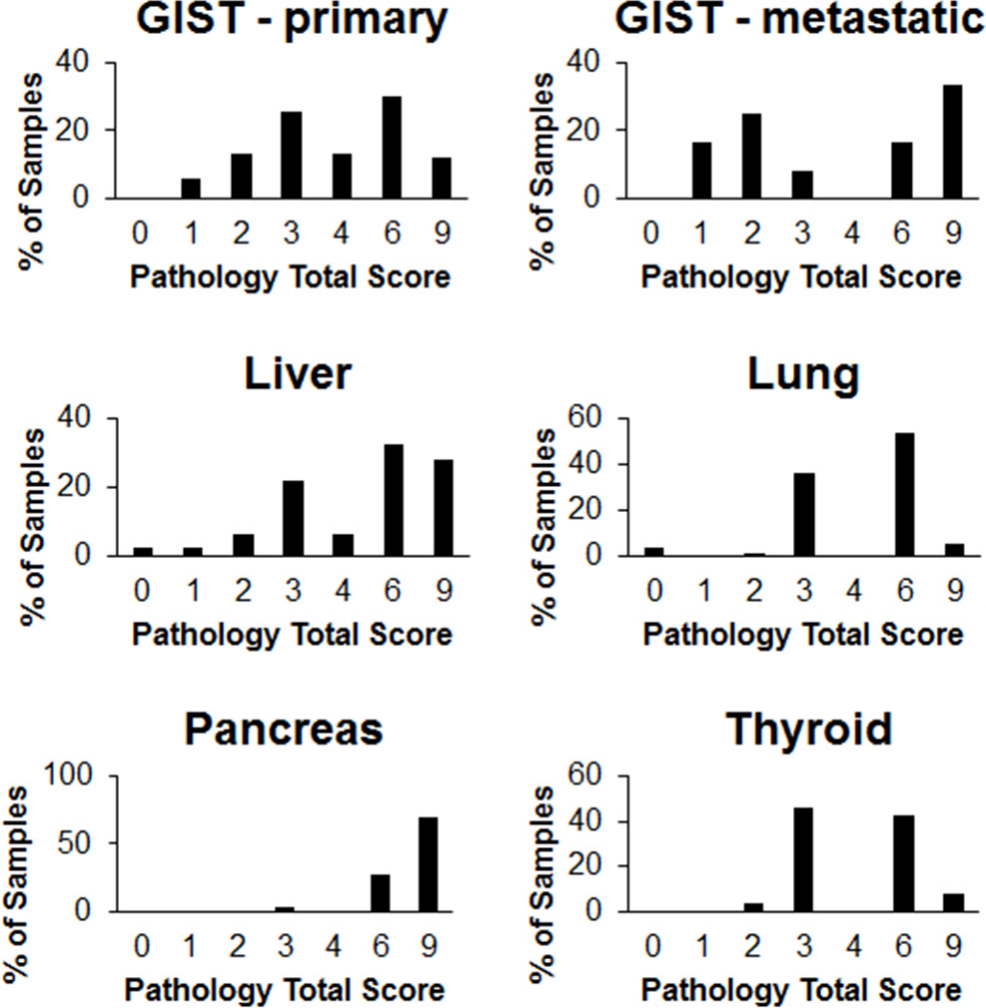
Proportion of patient cancer samples in each pathology total score category IHC was performed on tissue sections from various cancers using an antibody raised against CCK2R. The staining intensity and the proportion of tissue staining positive were each graded on a scale of 0 to 3. The pathology total score was derived by multiplying the staining intensity score and the coverage score.

**Figure 3:**
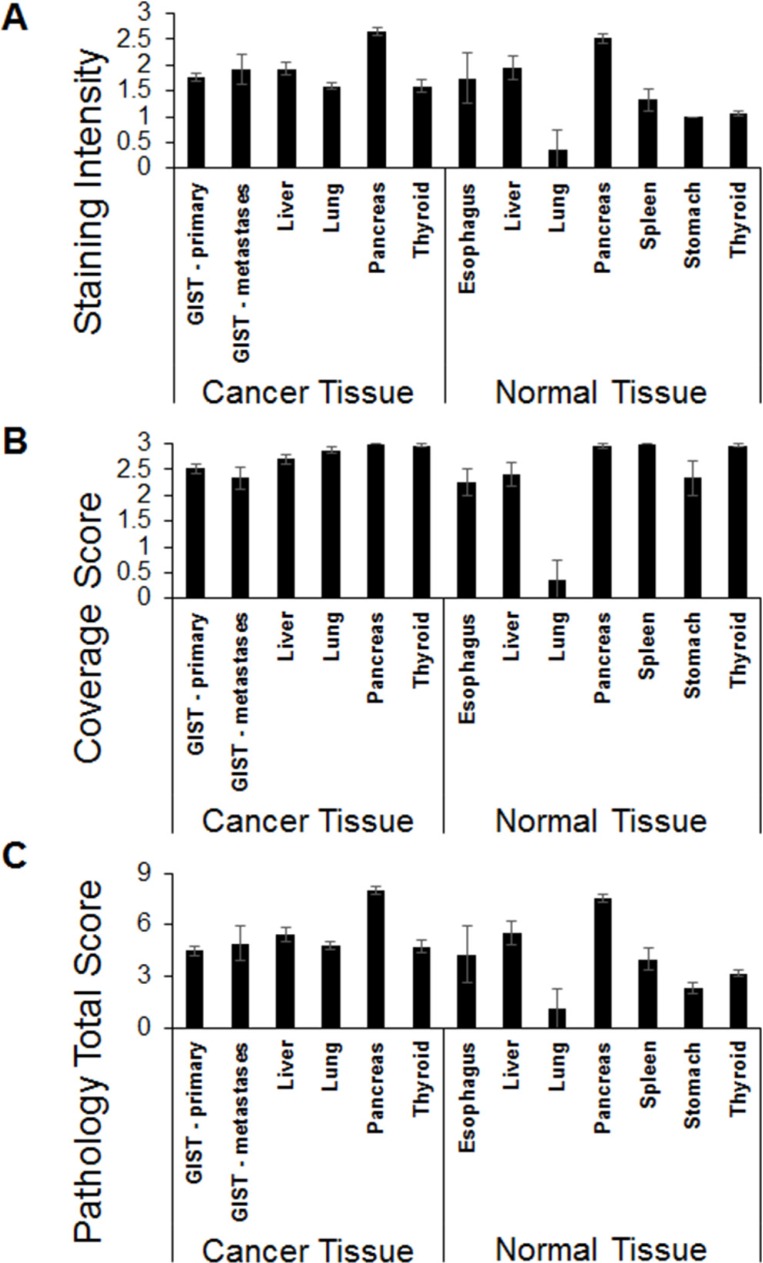
IHC staining of CCK2R in cancer and normal tissue specimens IHC was performed on tissue sections using an antibody raised against CCK2R. The staining intensity and the proportion of tissue staining positive were each graded on a scale of 0 to 3. The pathology total score was derived by multiplying the staining intensity score and the coverage score. The average value for the staining score (**A**), coverage score (**B**) and the total score (**C**) is plotted (error bars represent SEM).

Tissue microarrays (TMA) cores of GIST tumors were separated into two groups consisting of tumors from either primary or metastatic sites. Representative images are shown in Figure 1 (additional images can be found in the supporting information). As shown in Figure 2, all 67 GIST primary and 12 metastatic samples with accompanying patient data stained positive for CCK2R with an average staining intensity of 1.76 and 1.92 (out of 3), respectively. Interestingly, GIST had the lowest coverage score of any cancer tested with an average score of 2.52 and 2.33 (out of 3) for primary and metastatic tumors, respectively (Figure 3). No significant correlations or differences were identified between staining intensity, coverage score or pathology total score (See Supporting Information) when correlated with patient data (sex, age at diagnosis, primary tumor site, grade, tumor size and survival after diagnosis).

### Cancer tissue - liver

42 of 43 primary hepatocellular carcinomas stained positive for CCK2R (Figure 2). The average staining intensity was 1.93, with nearly an equal number of samples in each staining intensity group (*n* = 14, 15 and 13 in staining intensity groups 1, 2, 3, respectively). However, the coverage score was strongly biased towards high coverage, with an average score of 2.70 and only a single sample with a coverage score of 1. No significant correlations or differences were identified between staining intensity, coverage score or pathology total score (See Supporting Information) when correlated with patient data (sex, age at diagnosis, stage, grade, tumor size and survival after diagnosis).

### Cancer tissue - lung

As shown in Figure 2, 98 of 102 lung cancer samples stained positive for CCK2R. However, lung cancers were tied with thyroid cancers for the lowest staining intensities of all cancers tested (∼1.60 out of 3). In fact, only 5 samples scored in the highest staining group, while 38 and 55 samples were in the 1st and 2nd staining intensity groups, respectively. The staining intensity was not significantly different among any of the different lung cancers tested, including bronchoalveolar adenocarcinomas, clear cell adenocarcinomas, mucinous adenocarcinomas, non-small cell carcinomas and neuroendocrine carcinomas (*p*-value 0.136, see Supporting Information). The coverage of CCK2R staining was very broad, with 97 of 102 samples receiving the highest coverage score. A positive correlation between the coverage score and the stage of the cancer was identified (*p*-value 0.0143), with coverage scores less than 3 only present in Stage 1 cancers (see Supporting Information). No other significant correlations or differences were identified between staining intensities, coverage scores or pathology total scores (See Supporting Information) and patient data (sex, age at diagnosis, primary tumor site, stage, grade, tumor size, lymph node involvement, metastases, survival after diagnosis or survival after stage IV diagnosis) except for the location of metastases. In this specific case, lung metastases to the liver preferentially expressed lower levels of CCK2R (*p*-value = 0.012) than other metastatic sites.

### Cancer tissue - pancreas

All 55 pancreatic cancer samples stained positive for CCK2R. In fact, pancreatic cancers had both the highest average staining intensity (2.65) and highest average coverage score (3.0) of any cancer examined (Figure 3). Moreover, a correlation between staining intensity/pathology total score (See Supporting Information) and tumor size was observed (*p*-value = 0.009 for longest tumor dimension). This correlation most likely led to the significant difference observed between staining intensity and pathology total score (See Supporting Information) when compared with the stage of the tumor (*p*-value = 0.004). No other significant correlations or differences were identified.

### Cancer tissue - thyroid

All 27 thyroid cancer samples stained positive for CCK2R, although the average staining intensity of 1.6 was tied with lung cancer as the lowest. No obvious differences in staining intensities were found for the four different thyroid cancer types tested (follicular carcinomas, medullary carcinomas, oxyphilic adenocarcinomas and papillary adenocarcinomas) (*p*-value = 0.681). Medullary cancer had the lowest average staining intensity of 1.40 (see Supporting Information). The average coverage score of all thyroid cancers was 3, except follicular carcinomas, which had an average score of 2.67. This difference in coverage score was found to be significantly different with a *p*-value of 0.036 (see Supporting Information). Lymph node involvement was also found to be correlated positively with CCK2R staining intensity and pathology total score (See upporting Information), with *p*-values of 0.011 and 0.010, respectively. No other significant correlations or differences were identified.

### Normal tissue

**Figure 4:**
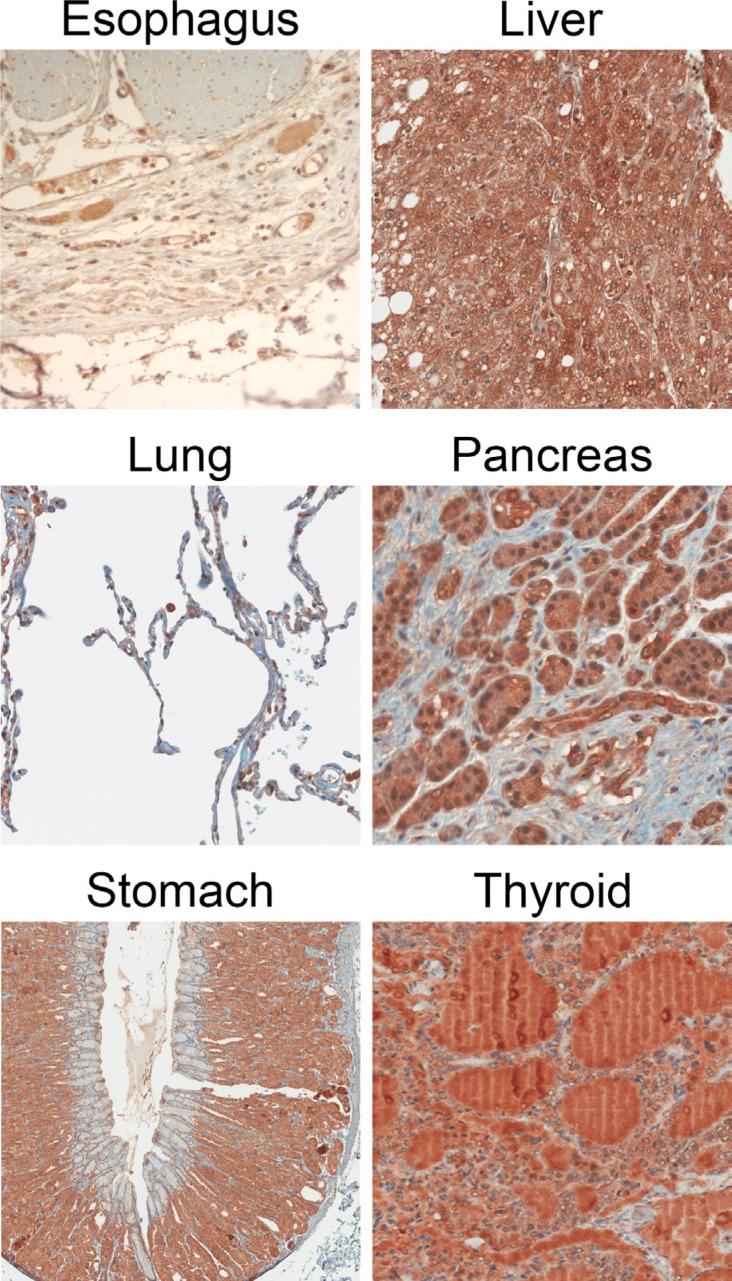
Example IHC images of normal tissue specimens using the CCK2R specific antibody 6C10G11

**Figure 5:**
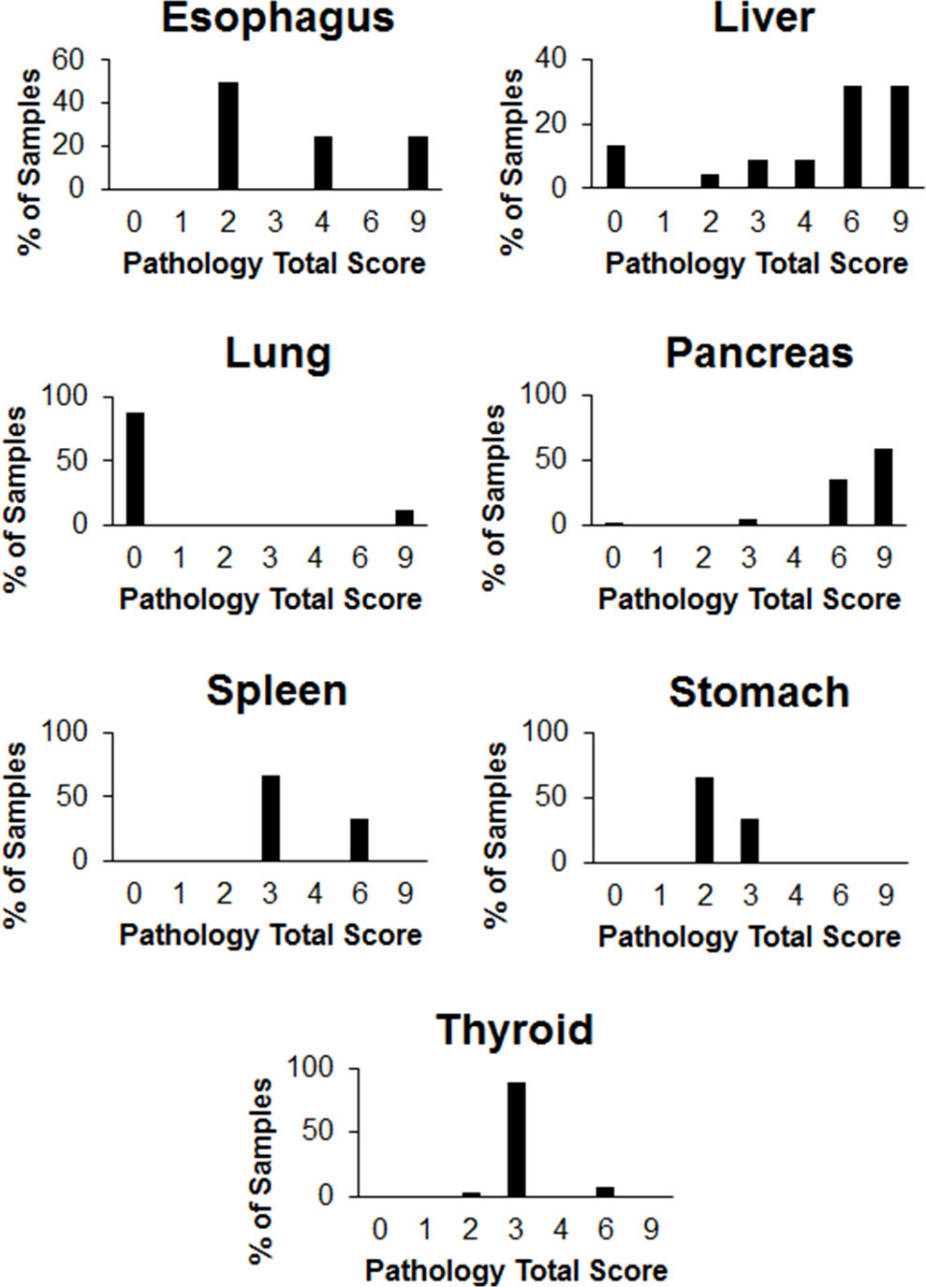
Proportion of patient normal tissue specimens in each pathology total score category IHC was performed on tissue sections from various normal organs using an antibody raised against CCK2R. The staining intensity and the proportion of tissue staining positive were each graded on a scale of 0 to 3. The pathology total score was derived by multiplying the staining intensity score and the coverage score.

Normal tissues from the esophagus, liver, lung, pancreas, spleen, stomach and thyroid also stained positive for CCK2R. Representative images can be seen in Figure 4. Liver and pancreatic tissues stained most strongly, with average staining intensities of 2.52 and 1.95, respectively (Figure 3), while normal lung tissue stained the weakest (Figure 3). Nearly all tissues except for lung had a high percentage of cells staining positive (Figure 5). Average staining intensities, coverage scores and pathology total scores can be seen in Figure 3.

### Normal pancreatic tissue

As pancreatic tissue had the highest staining intensity of any normal tissue, a more detailed analysis of the different pancreatic cells that expressed CCK2R was performed. As shown in the Supporting Information, acinar, intercalated duct (IC Duct), intermediate or large duct (Int/Large Duct) and islet cells were individually scored. Acinar, IC Duct and Int/Large Duct cells all exhibited nearly identical average staining intensities (2.58, 2.72 and 2.60, respectively), coverage scores (3.0, 3.0 and 3.0, respectively) and pathology total scores (7.75, 8.17 and 7.8, respectively). Islet cells, however, had lower CCK2R staining, with an average intensity of 1.57, coverage score of 2.57 and pathology total score of 4.71.

### Overexpression comparison

**Figure 6:**
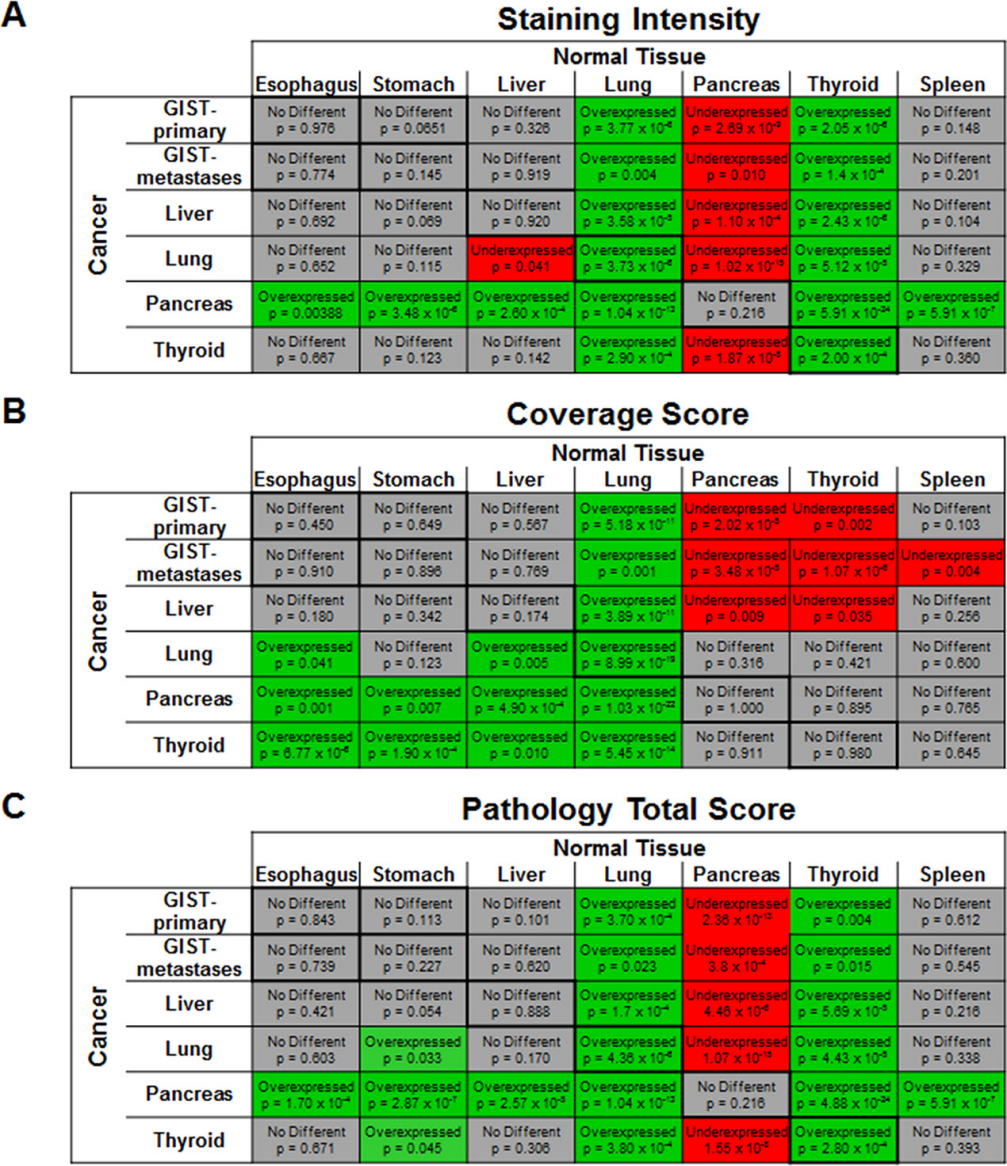
Differences in CCK2R expression in cancers vs. normal tissue IHC was performed on tissue sections using an antibody raised against CCK2R. The staining intensity and the proportion of tissue staining positive (coverage score) were each graded on a scale of 0 to 3. The pathology total score was derived by multiplying the staining intensity score and the coverage score. The CCK2R expression of each cancer was compared to each normal tissue. Differences were considered significant if the *p*-value was < 0.05. If the cancer was significantly higher than the normal tissue, the box was colored green and denoted as overexpressed. A significantly lower value in the cancer was colored red and denoted as underexpressed while a grey color represents no statistically significant difference between the cancer and normal tissue. Comparisons between the staining score (**A**), coverage score (**B**) and the pathology total score (**C**) are shown. The *p*-value is also shown in each corresponding box. Boxes with darker borders represent cancer and normal tissue of the same organ origin.

The staining intensity, coverage score and pathology total score for each cancer was compared against every normal tissue in order to identify any statistically significant increase or decrease in CCK2R expression. As shown in Figure 6, when each cancer type was compared with its corresponding normal tissue, only lung and thyroid cancers appeared to overexpress CCK2R. This overexpression is also apparent in the pathology total score. However, even though lung and thyroid cancers exhibited greater staining intensity than their corresponding normal tissues, no cancer's staining was greater than all other normal tissues tested. In fact, when compared to normal pancreas tissue, nearly every cancer's staining intensity and pathology total score was significantly less.

## DISCUSSION

CCK2R previously has been found on many cancers and in our studies it was also present on 303 out of 308 cancer sections tested (Summarized in Table 1). With such a high proportion of cancers expressing CCK2R, one might have expected to find a correlation between CCK2R level and tumor stage, tumor grade, or patient survival, etc. However, this study did not detect any overarching correlation with patient data. These results are, in fact, consistent with other CCK2R studies, where no significant relationship has been found between CCK2R expression and lymph node involvement, metastatic disease, overall survival, patient age or sex [2, 7, 9, 11, 14, 37].

Even though CCK2R could not be correlated with patient pathological data [2, 7, 9, 11, 14, 37], the previously reported overexpression of CCK2R on certain cancers [4, 6, 7, 8, 23] still promoted exploration of its use for delivery of ligand-targeted cancer imaging agents [19, 24–36, 38, 39]. Unfortunately, none of these imaging studies reported quantitation of CCK2R in the malignant disease relative to normal tissues. In fact, in the sole study where a quantitative comparison was made (by radioligand binding), the only comparator was normal tissue from the same origin [8]. This single tissue comparison may constitute a cause for concern regarding the use of CCK2R as a drug or diagnostic imaging agent target, since a wide range of normal tissues including stomach [2, 5, 13, 21–23], small intestines [5], large intestines/colon [5, 11], pancreas [5, 10, 40], liver [5, 17], thyroid [21], brain [2], monocytes [1] and T lymphocytes [1] all have been shown to express CCK2R. Indeed, we found CCK2R on every normal tissue that we stained. Moreover, imaging studies with CCK2R ligands in humans have shown high levels of accumulation in the stomach [19, 31, 34, 36, 41], breast [31, 36], nipples [31], bowels [19, 31, 41], gallbladder [34, 36, 41] and pancreas [34], also suggesting a wide distribution of CCK2R in normal human tissues. Thus, while CCK2R may allow detection of thyroid cancer within the normal thyroid gland, it would not seem to be suitable for detection of metastatic disease in many other tissues. Indeed, if the qualitative data reported here can be used as a guide for design of CCK2R targeted therapeutics, none of the cancers examined exhibit statistically greater expression than all other normal tissues. This hypothesis would, in fact, be consistent with the observation that significant toxicity was observed in a human clinical trial of a ^90^Y-labelled mini-gastrin peptide for radiotherapy of medullary thyroid cancers [36].

In conclusion, while some cancers may express more CCK2R than their normal tissue counterparts, the expression of CCK2R in other normal tissues also must be considered when CCK2R-targeted ligands are evaluated for use as imaging or therapeutic agents. Additionally, this study strongly suggests that normal tissue samples from the primary tumor organ site should be examined before a receptor is deemed overexpressed or upregulated, and normal tissues should be examined from multiple organ sites of concern for toxicity before a marker is determined to be appropriate for the ligand targeting of therapeutic agents, or of concern for background signal interference before targeting of imaging agents.

## MATERIALS AND METHODS

### Materials

EZ Prep solution, cell conditioning 1 solution, ChromoMap kit and OmniMap anti-mouse secondary antibody were purchased from Ventana Medical Systems (Tucson, AZ). Dako antibody diluent was purchased from Dako (Carpenteria, CA). Sections were obtained from existing tissue microarrays (TMAs) in the tissue microarray service and formalin-fixed paraffin-embedded (FFPE) tissue sections from the biorepository in the Moffitt Cancer Center Tissue Core facility. The following Moffitt TMAs were sectioned: the GIST TMA containing cores from 134 (of which 67 primary and 12 metastatic tumors had corresponding patient data) GIST specimens; the HCC TMA containing 46 cores (of which 43 primary tumors had corresponding patient data); a lung TMA consisting of 102 NSCLC cores from patients who all eventually developed stage IV disease and 8 cores containing normal (unaffected) lung tissue; and a pancreatic TMA containing cores from 55 pancreatic adenocarcinomas, 7 pancreatic intraepithelial neoplasms (low grade, PanIN1), and 66 normal pancreas specimens including normal intercalated duct (IC Duct), normal intermediate or large duct (Int/Large Duct), normal acini (acinar), and islet cells (islets). Since the GIST and HCC TMAs did not contain normal GI tissue or liver tissue, 7 GI tissue specimens (4 with normal esophagus and 3 with normal stomach tissue) and 18 liver specimens containing tumor and surrounding normal tissue were also sectioned and stained as controls. Since there was no thyroid cancer TMA available, 27 thyroid cancer specimens with adjacent normal thyroid tissue and 1 normal specimen without tumor were sectioned. The lung TMA also had the following normal tissue cores: 4 liver, 6 spleen and 1 thymus. The TMAs were generated prior to the beginning of the study and were originally generated with a compiled set of clinical data corresponding to individual cores. Moffitt also has a substantial database containing clinical information corresponding to patient specimens that can be de-identified and made available to researchers. All tissues and data were pre-existing and de-identified prior to transfer to the study team by the Moffitt Tissue Core honest broker or the Moffitt Information Shared Services honest broker. No new human subjects were recruited for the study. The study was reviewed by the Moffitt Protocol Support Office which determined that the study qualified as Non Human Subjects Research (NHSR) as outlined in guidance provided by the Office of Human Research protection (OHRP) in 45 CFR 46.102.

### Antibody

A custom humanized mouse monoclonal anti-human CCK2R antibody was generated against the antigen sequence CPRPPRARPRALPDE by Genscript (Piscataway, NJ). In total, four hybridoma cell lines were generated that produced four different monoclonal antibodies 1F4E8, 1F4G6, 6C10G11 and 6C10H3. These four antibodies were tested for specificity and selectivity towards the CCK2R receptor. While all four antibodies exhibited similar patterns of binding, the 6C10G11 antibody was found to be the most specific and was used for all further studies.

### Immunohistochemistry

Slides with TMA and tissue sections were stained using a Ventana Discovery XT automated system (Ventana Medical Systems, Tucson AZ) as per manufacturer's protocol with proprietary reagents. Briefly, slides were deparaffinized on the automated system with EZ Prep solution and the heat-induced antigen retrieval method was used in Cell Conditioning 1 solution. Staining conditions were optimized in consultation with the study pathologists using tissues with known positive CCK2R expression (normal stomach epithelium and normal gall bladder epithelium) and negative CCK2R expression (normal gallbladder smooth muscle) and the following conditions used to stain all specimens: The CCK2R humanized mouse monoclonal antibody was incubated for 4 hours at a 1:200 concentration diluted in Dako antibody diluent. The secondary OmniMap anti-mouse antibody was incubated for 16 min. Secondary antibody was detected using the ChromoMap kit and slides were counterstained with Hematoxylin. Tissues were scored by pathologists specializing in each tumor type, i.e. GIST and HCC (D.C), lung (F.K.K.), pancreatic (B.A.C.) and thyroid (M.E.L.). The intensity (Staining Intensity) was scored on a scale of 0 to 3 while the percent of tissue staining (Coverage Score) also was scored on a 0 to 3 scale where 0 = 0%, 1 = 1–33%, 2 = 34–66% and 3 = 67–100% stained. For cancer samples, coverage and intensity were only determined for cancer cells and not for stromal components. The pathology total score was obtained by multiplying the intensity score by the percent stained score.

### Statistics

Correlation analyses were performed using a Spearman correlation analysis while differences between groups were determined via a *t*-test (assuming equal variance and 2-tails for all patient pathology data and 1-tail for cancer overexpression levels) or 1-way ANOVA. Correlations and differences were considered significant if the *p*-value was < 0.05. Ad hoc post analyses of significant 1-way ANOVA tests were performed using a Tukey-Kramer test.
