# Addendum: Assessment of cholecystokinin 2 receptor (CCK2R) in neoplastic tissue

**DOI:** 10.18632/oncotarget.28699

**Published:** 2025-03-06

**Authors:** Jyoti Roy, Karson S. Putt, Domenico Coppola, Marino E. Leon, Farah K. Khalil, Barbara A. Centeno, Noel Clark, Valerie E. Stark, David L. Morse, Philip S. Low

**Affiliations:** ^1^Center for Drug Discovery, Purdue University, West Lafayette IN 47907 USA; ^2^Department of Chemistry, Purdue University, West Lafayette IN 47907 USA; ^3^Department of Anatomic Pathology, H. Lee Moffitt Cancer Center, Tampa FL 33612 USA; ^4^Tissue Core, H. Lee Moffitt Cancer Center, Tampa FL 33612 USA; ^5^Department of Cancer Imaging and Metabolism, Imaging and Technology Center of Excellence, H. Lee Moffitt Cancer Center, Tampa FL 33612 USA

**This article has an addendum:** The Supplementary Material file has been added to this article.

Original article: Oncotarget. 2016; 7:14605–14615. 14605-14615. https://doi.org/10.18632/oncotarget.7522

## SUPPLEMENTARY MATERIALS



