# Supplementary material for: Addendum: Assessment of cholecystokinin 2 receptor (CCK2R) in neoplastic tissue
Source: Oncotarget. 2025 Mar 6;16:132. doi: 10.18632/oncotarget.28699 (PMC11884436; doi:10.18632/oncotarget.28699)
Supplement: Supplementary file 1 [file oncotarget-16-28699-s001.pdf]

Supporting Information - Table of Contents

Cancer Tissue Global Summary

Global Staining Intensity	SI Figure 1 to 2
Global Coverage Score	SI Figure 3 to 4
Global Total Score	SI Figure 5 to 6

GIST – primary

Images	SI Figure 7 to 13
Overall Summary	SI Figure 14
Staining Intensity and Correlations	SI Figure 15 to 22
Coverage Score and Correlations	SI Figure 23 to 30
Total Staining Score and Correlations	SI Figure 31 to 38

GIST - metastases

Overall Summary	SI Figure 39
Staining Intensity and Correlations	SI Figure 40 to 45
Coverage Score and Correlations	SI Figure 46 to 51
Total Staining Score and Correlations	SI Figure 52 to 57

Liver Cancer

Images	SI Figure 58 to 65
Overall Summary	SI Figure 66
Staining Intensity and Correlations	SI Figure 67 to 77
Coverage Score and Correlations	SI Figure 78 to 88
Total Staining Score and Correlations	SI Figure 89 to 99

Lung Cancer

Images	SI Figure 100 to 102
Overall Summary	SI Figure 103
Staining Intensity and Correlations	SI Figure 104 to 118
Coverage Score and Correlations	SI Figure 119 to 133
Total Staining Score and Correlations	SI Figure 134 to 148

Supporting Information - Table of Contents

Pancreatic Cancer

Images	SI Figure 149 to 156
Overall Summary	SI Figure 157
Staining Intensity and Correlations	SI Figure 158 to 170
Coverage Score and Correlations	SI Figures 171 to 183
Total Staining Score and Correlations	SI Figures 184 to 196

Thyroid Cancer

Images	SI Figure 197 to 199
Overall Summary	SI Figure 200
Staining Intensity and Correlations	SI Figure 201 to 211
Coverage Score and Correlations	SI Figure 212 to 222
Total Staining Score and Correlations	SI Figure 223 to 233

Normal Tissue Global Summary

Global Staining Intensity	SI Figure 234 to 235
Global Coverage Score	SI Figure 236 to 237
Global Total Score	SI Figure 238 to 239

Esophagus

Images	SI Figure 240 to 241
Staining Intensity	SI Figure 242
Coverage Score	SI Figure 243
Total Staining Score	SI Figure 244

Liver

Images	SI Figure 245 to 246
Staining Intensity	SI Figure 247
Coverage Score	SI Figure 248
Total Staining Score	SI Figure 249

Supporting Information - Table of Contents

**Lung**		
	Images	SI Figure 250
	Staining Intensity	SI Figure 251
	Coverage Score	SI Figure 252
	Total Staining Score	SI Figure 253
**Pancreas**		
	Images	SI Figure 254 to 259
	Staining Intensity	SI Figure 260 to 262
	Coverage Score	SI Figure 263 to 265
	Total Staining Score	SI Figure 266 to 268
**Spleen**		
	Staining Intensity	SI Figure 269
	Coverage Score	SI Figure 270
	Total Staining Score	SI Figure 271
**Stomach**		
	Images	SI Figure 272 to 275
	Staining Intensity	SI Figure 276
	Coverage Score	SI Figure 277
	Total Staining Score	SI Figure 278
**Thyroid**		
	Overall Summary	SI Figure 279
	Staining Intensity and Correlations	SI Figure 280 to 283
	Coverage Score and Correlations	SI Figure 284 to 287
	Total Staining Score and Correlations	SI Figure 288 to 291

Cancer Tissue

Cancer Tissue
Global Summary

Cancer Tissue
Global Staining Intensity

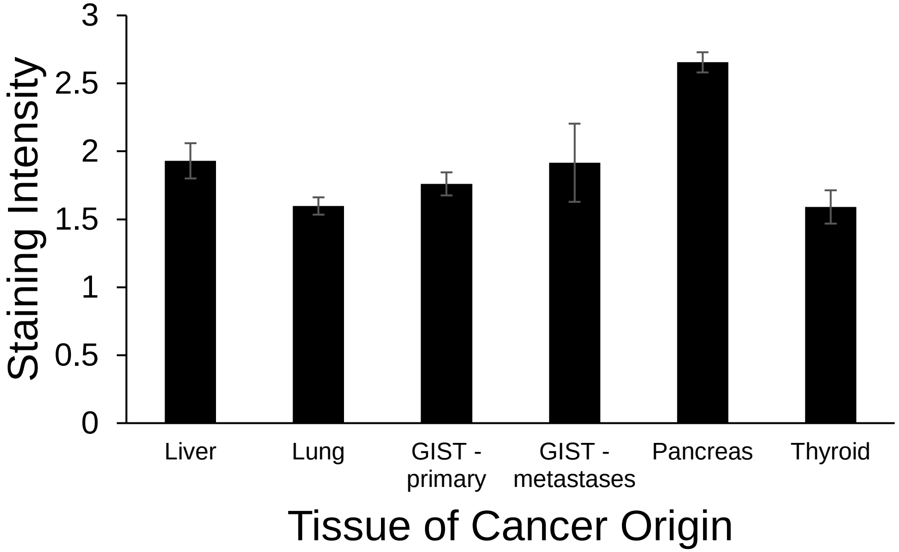

Staining Intensity

	n	Average	St. Dev	St. Error
Liver	43	1.93	0.86	0.13
Lung	102	1.60	0.65	0.06
GIST - primary	67	1.76	0.70	0.09
GIST - metastases	12	1.92	1.00	0.29
Pancreas	55	2.65	0.55	0.07
Thyroid	27	1.59	0.64	0.12

SI Figure 1. Average CCK2R Staining Intensity for all cancer tissues tested. IHC was performed on tumor tissue sections using a monoclonal antibody raised against CCK2R. The intensity of staining was graded on a scale of 0 to 3 and plotted (error bars represent standard error of the mean).

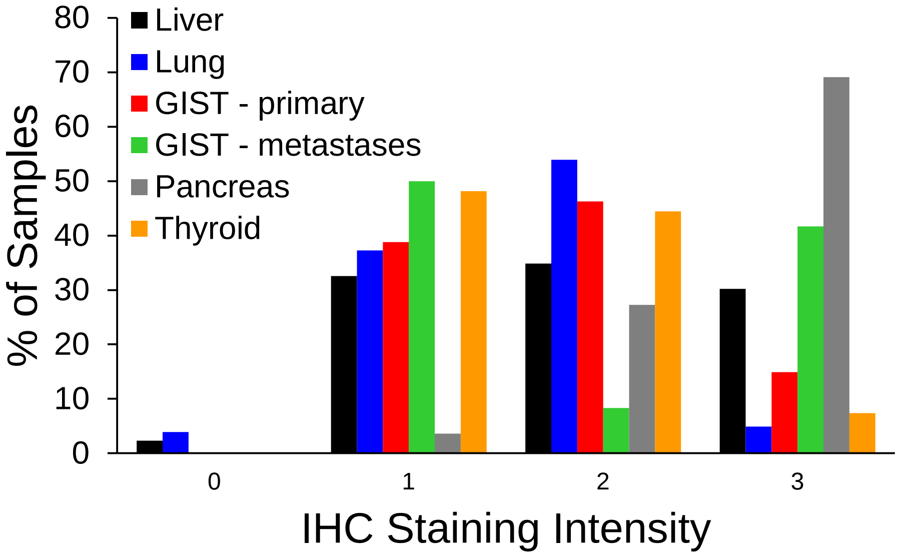

	Staining Intensity (n in each group)			
	0	1	2	3
Liver	1	14	15	13
Lung	4	38	55	5
GIST - primary	0	26	31	10
GIST - metastases	0	6	1	5
Pancreas	0	2	15	38
Thyroid	0	13	12	2

	Staining Intensity (% in each group)			
	0	1	2	3
Liver	2.33	32.56	34.88	30.23
Lung	3.92	37.25	53.92	4.90
GIST - primary	0.00	38.81	46.27	14.93
GIST - metastases	0.00	50.00	8.33	41.67
Pancreas	0.00	3.64	27.27	69.09
Thyroid	0.00	48.15	44.44	7.41

SI Figure 2. CCK2R Staining Intensity for all cancer tissues tested. IHC was performed on tumor tissue sections using a monoclonal antibody raised against CCK2R. The intensity of staining was graded on a scale of 0 to 3 and plotted.

Cancer Tissue
Global Coverage Score

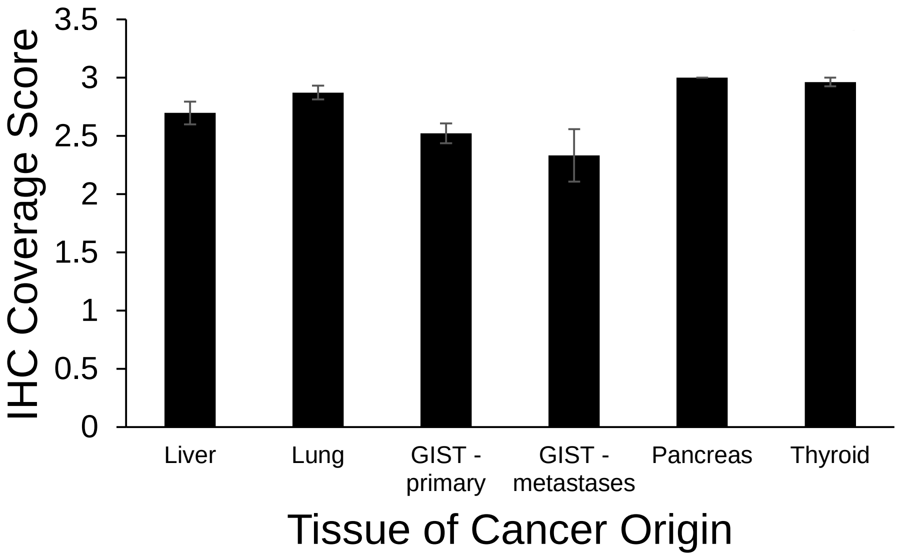

Coverage Score

	n	Average	St. Dev	St. Error
Liver	43	2.70	0.64	0.10
Lung	102	2.87	0.59	0.06
GIST - primary	67	2.52	0.70	0.09
GIST - metastases	12	2.33	0.78	0.22
Pancreas	55	3.00	0.00	0.00
Thyroid	27	2.96	0.19	0.04

SI Figure 3. Average CCK2R Coverage Score for all cancer tissues tested. IHC was performed on tumor tissue sections using a monoclonal antibody raised against CCK2R. The area stained (coverage) was graded on a scale of 0 to 3 and plotted (error bars represent standard error of the mean.

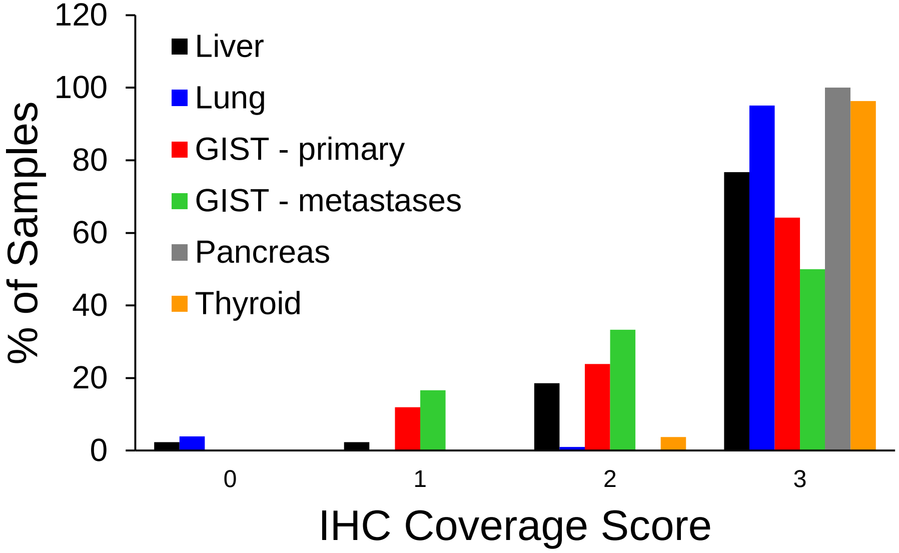

	Coverage Score (n in each group)			
	0	1	2	3
Liver	1	1	8	33
Lung	4	0	1	97
GIST - primary	0	8	16	43
GIST - metastases	0	2	4	6
Pancreas	0	0	0	55
Thyroid	0	0	1	26

	Coverage Score (% in each group)			
	0	1	2	3
Liver	2.33	2.33	18.60	76.74
Lung	3.92	0.00	0.98	95.10
GIST - primary	0.00	11.94	23.88	64.18
GIST - metastases	0.00	16.67	33.33	50.00
Pancreas	0.00	0.00	0.00	100.00
Thyroid	0.00	0.00	3.70	96.30

SI Figure 4. CCK2R Coverage Score for all cancer tissues tested. IHC was performed on tumor tissue sections using a monoclonal antibody raised against CCK2R. The area stained (coverage) was graded on a scale of 0 to 3 and plotted.

Cancer Tissue
Global Total Staining Score

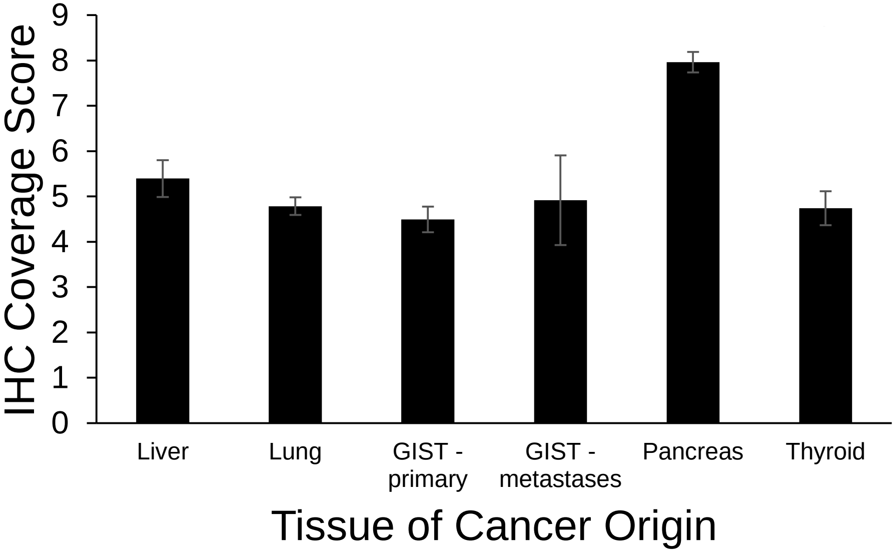

Total Staining Score

	n	Average	St. Dev	St. Error
Liver	43	5.40	2.65	0.40
Lung	102	4.78	1.96	0.19
GIST - primary	67	4.49	2.31	0.28
GIST - metastases	12	4.92	3.42	0.99
Pancreas	55	7.96	0.17	0.22
Thyroid	27	4.74	1.95	0.38

SI Figure 5. Average CCK2R Total Staining Score for all cancer tissues tested. IHC was performed on tumor tissue sections using a monoclonal antibody raised against CCK2R. The staining intensity and coverage score was multiplied to obtain the total staining score and plotted (error bars represent standard error of the mean).

Total Staining Score (n in each group)	0	1	2	3	4	6	9
Liver	1	1	3	10	3	15	13
Lung	4	0	1	37	0	55	5
GIST - primary	0	4	9	17	9	20	8
GIST - metastases	0	2	3	1	0	2	4
Pancreas	0	0	0	2	0	15	38
Thyroid	0	0	1	12	0	11	2

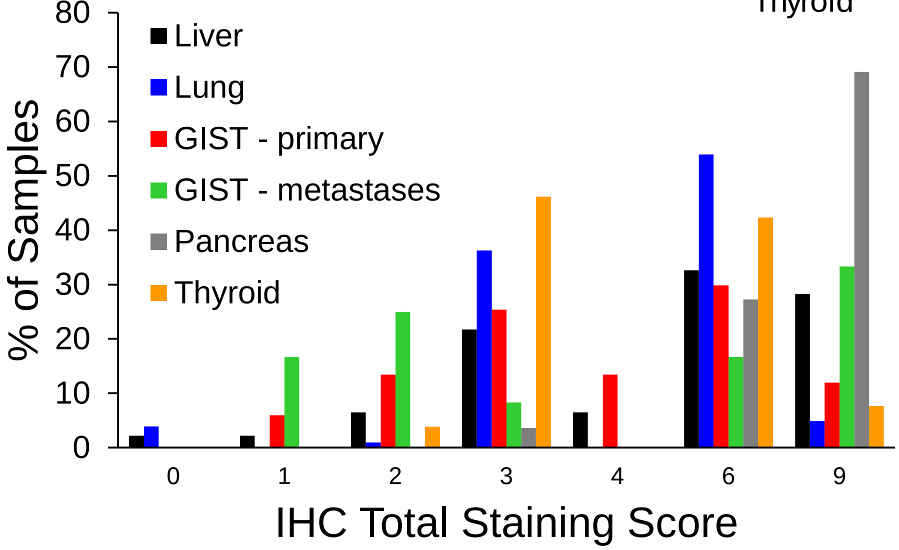

Total Staining Score (% in each group)	0	1	2	3	4	6	9
Liver	2.17	2.17	6.52	21.74	6.52	32.61	28.26
Lung	3.92	0.00	0.98	36.27	0.00	53.92	4.90
GIST - primary	0.00	5.97	13.43	25.37	13.43	29.85	11.94
GIST - metastases	0.00	16.67	25.00	8.33	0.00	16.67	33.33
Pancreas	0.00	0.00	0.00	3.64	0.00	27.27	69.09
Thyroid	0.00	0.00	3.85	46.15	0.00	42.31	7.69

SI Figure 6. CCK2R Total Staining Score for all cancer tissues tested. IHC was performed on tumor tissue sections using a monoclonal antibody raised against CCK2R. The staining intensity and coverage score was multiplied to obtain the total staining score and plotted.

GIST
primary tumor

GIST primary tumor Images

GIST, pathology total score = 0

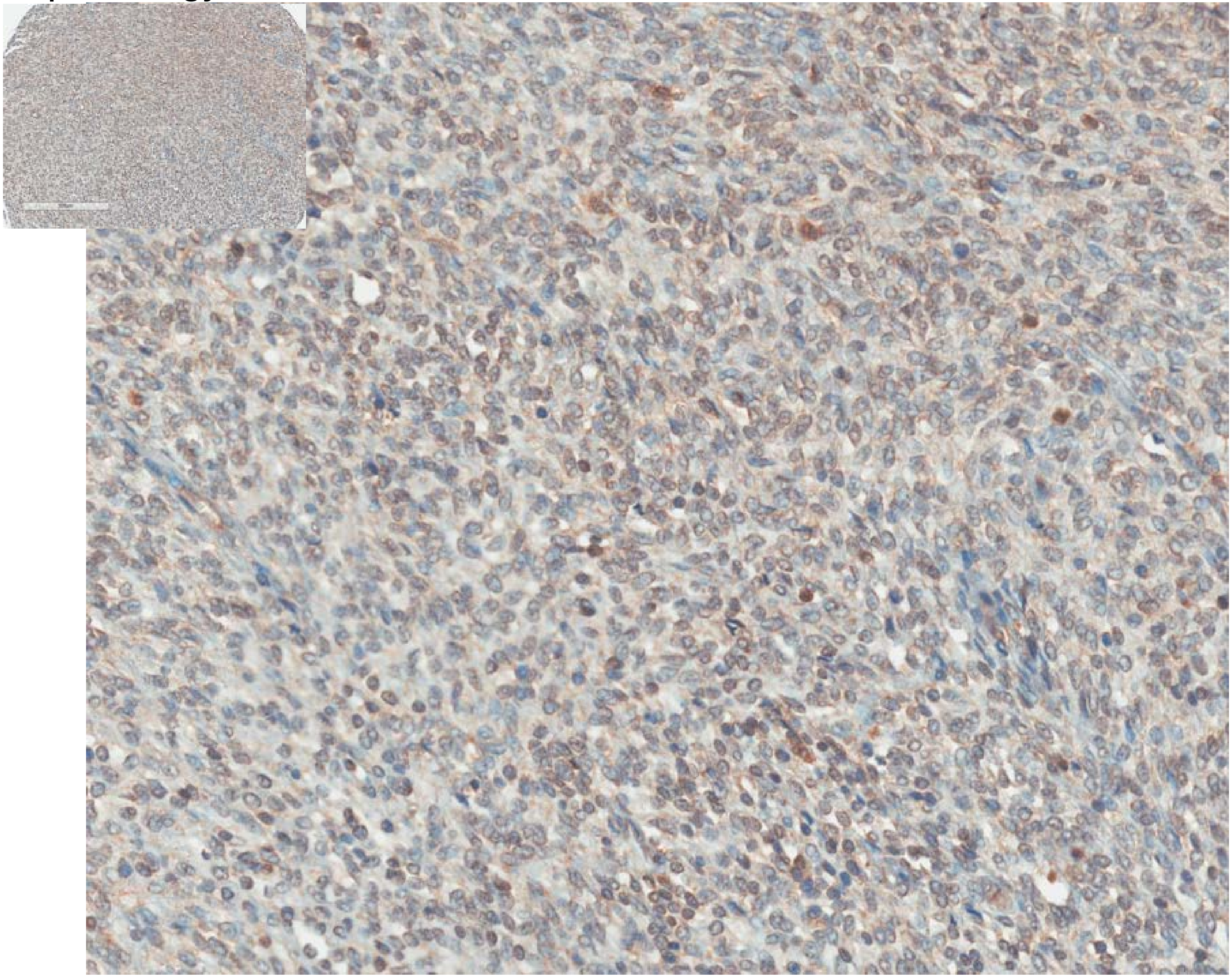

SI Figure 7. Example image of stained tissue from GIST. IHC was performed on tissue sections using a monoclonal antibody raised against CCK2R.

GIST, pathology total score = 1

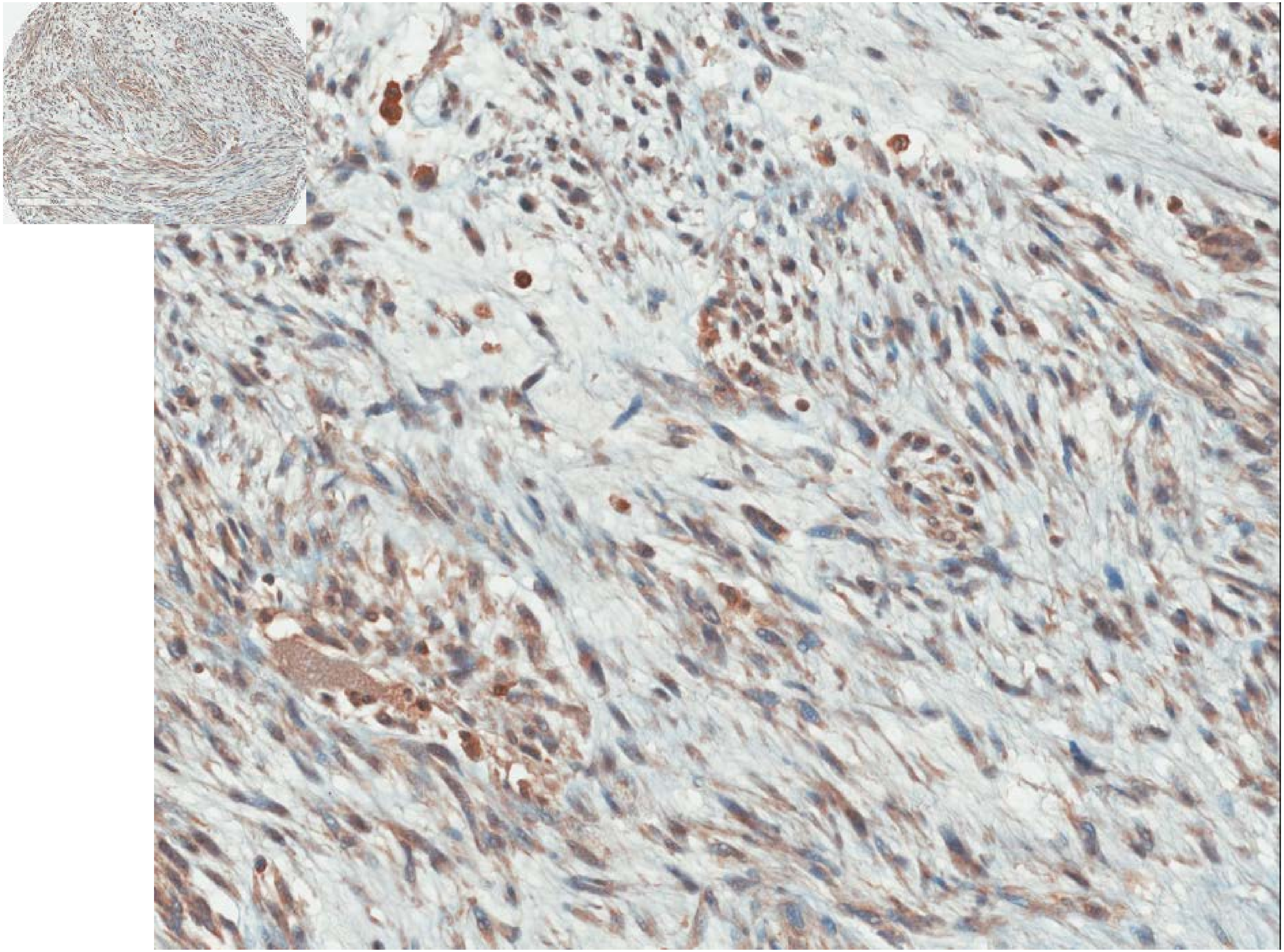

SI Figure 8. Example image of stained tissue from GIST. IHC was performed on tissue sections using a monoclonal antibody raised against CCK2R.

GIST, pathology total score = 2

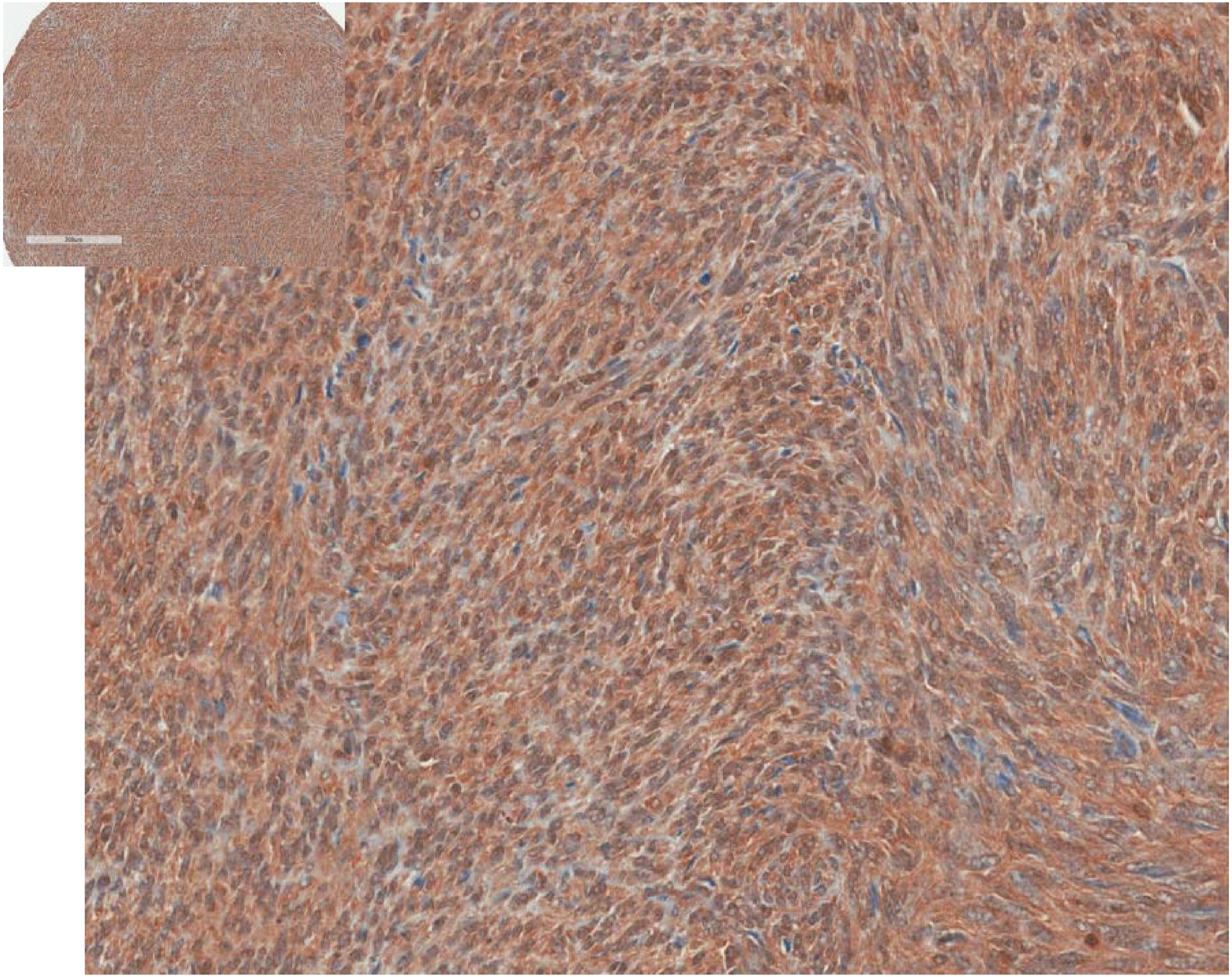

SI Figure 9. Example image of stained tissue from GIST. IHC was performed on tissue sections using a monoclonal antibody raised against CCK2R.

GIST, pathology total score = 3

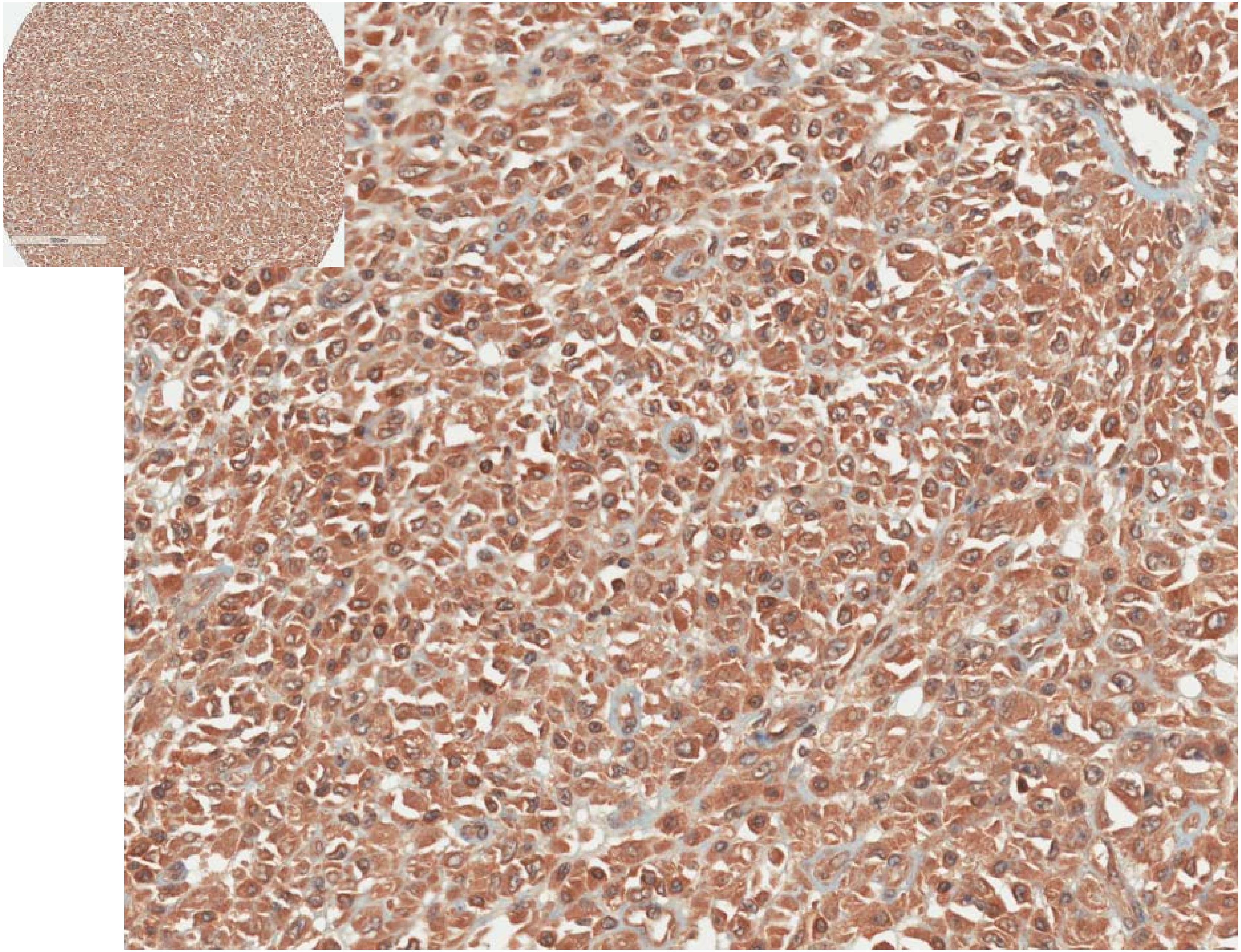

SI Figure 10. Example image of stained tissue from GIST. IHC was performed on tissue sections using a monoclonal antibody raised against CCK2R.

GIST, pathology total score = 4

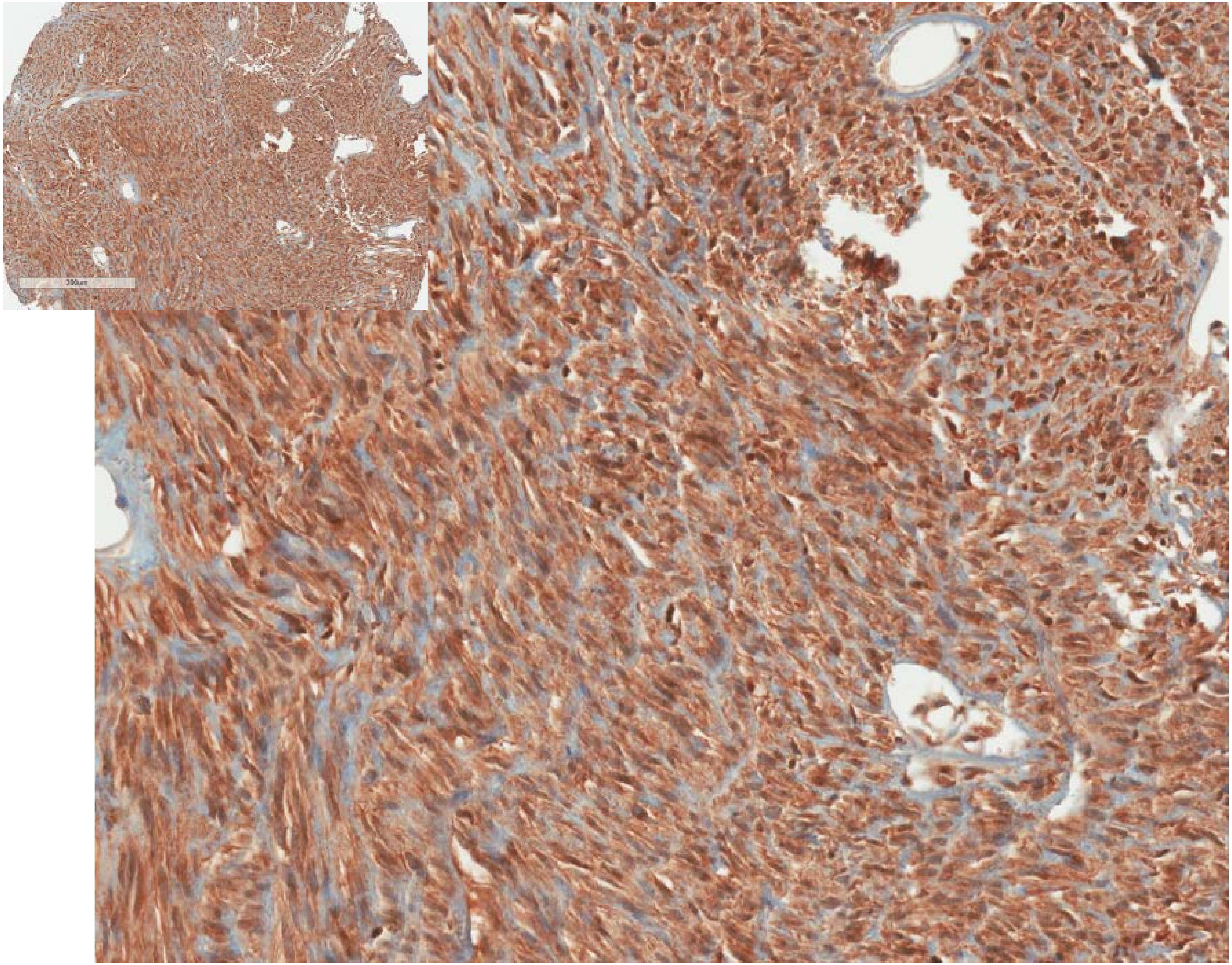

SI Figure 11. Example image of stained tissue from GIST. IHC was performed on tissue sections using a monoclonal antibody raised against CCK2R.

GIST, pathology total score = 6

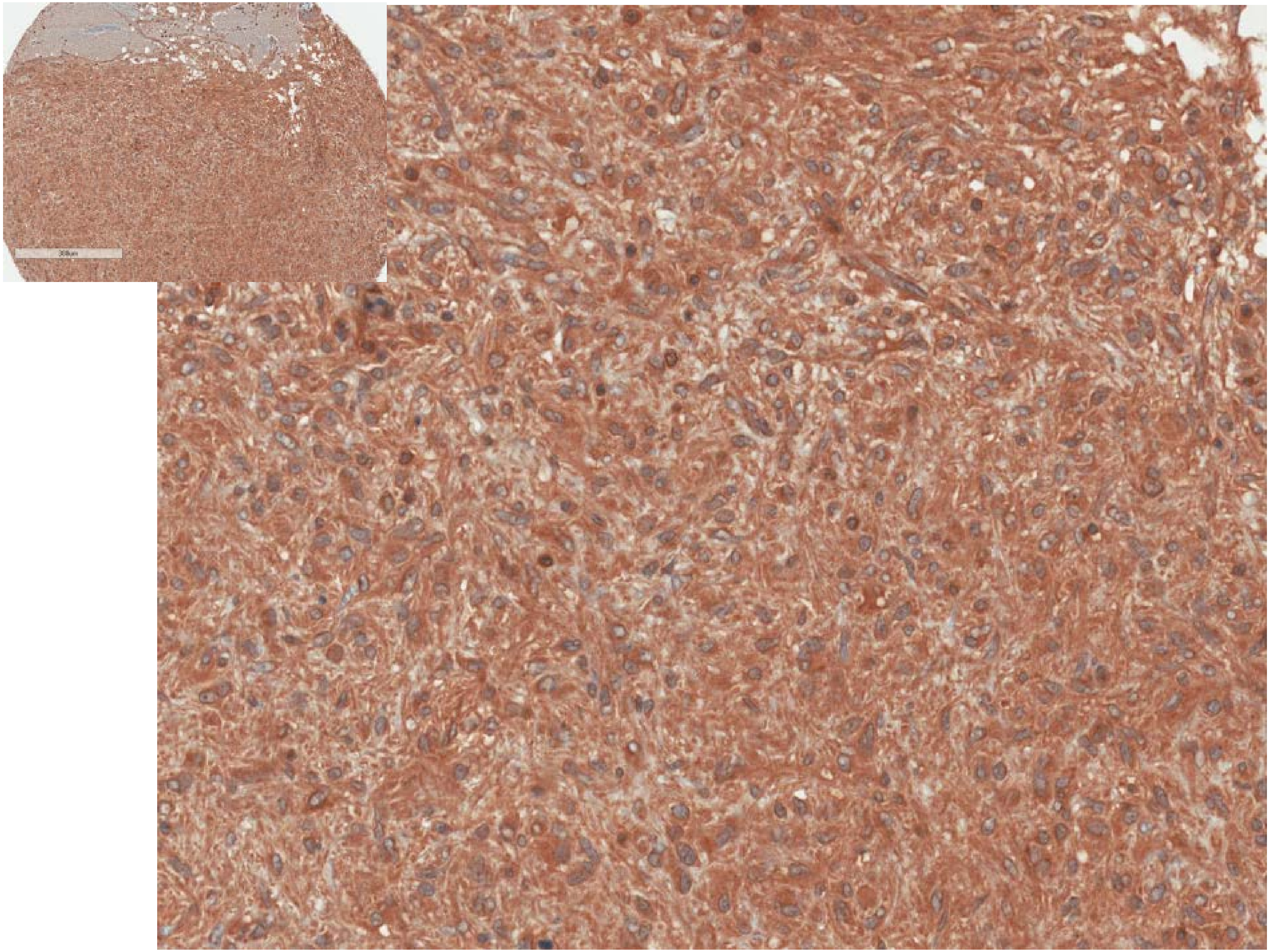

SI Figure 12. Example image of stained tissue from GIST. IHC was performed on tissue sections using a monoclonal antibody raised against CCK2R.

GIST, pathology total score = 9

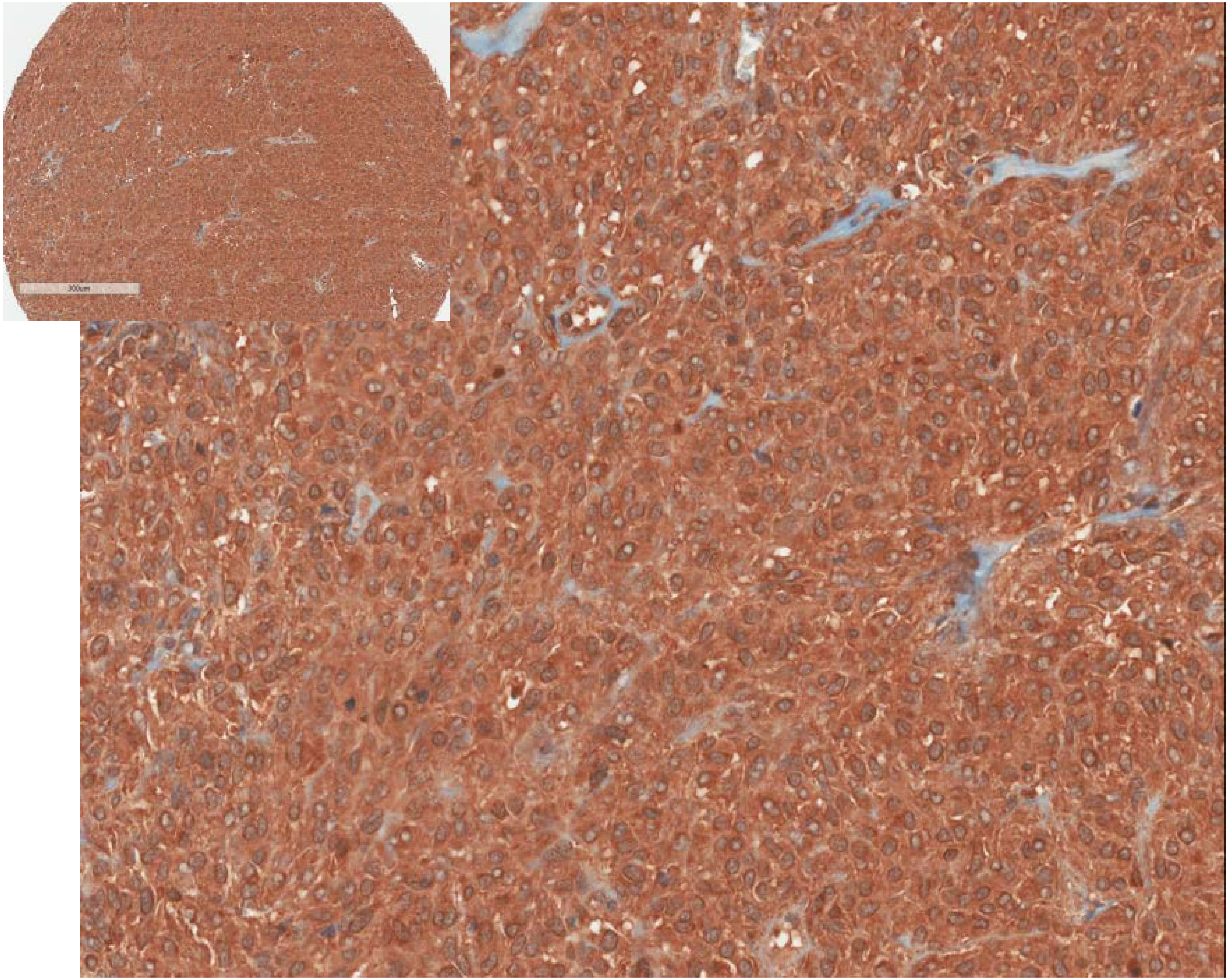

SI Figure 13. Example image of stained tissue from GIST. IHC was performed on tissue sections using a monoclonal antibody raised against CCK2R.

GIST primary tumor
Overall Summary

GIST - primary tumor - Spearman Correlation

	Sex	Age at Diagnosis	Primary Tumor Type	Primary Tumor Site	Stage	Grade	Tumor Size (TNM, T)	Tumor Size (longest dimension)	Lymph Node Involvement (TNM, N)	Metastatic (TNM, M)	Metastatic Site	Survival after Diagnosis	Survival after Stage IV Diagnosis
**Staining Intensity**	N.A.	No 0.425	N.A.	N.A.	N.D.	No 0.860	N.D.	No 0.453	N.D.	N.D.	N.A.	No 0.524	N.D.
**Coverage Score**	N.A.	No 0.263	N.A.	N.A.	N.D.	No 0.860	N.D.	No 0.519	N.D.	N.D.	N.A.	No 0.961	N.D.
**Total Staining Score**	N.A.	No 0.247	N.A.	N.A.	N.D.	No 0.834	N.D.	No 0.357	N.D.	N.D.	N.A.	No 0.597	N.D.

GIST - primary tumor - Spearman Correlation - ANOVA/t-test

	Sex	Age at Diagnosis	Primary Tumor Type	Primary Tumor Site	Stage	Grade	Tumor Size (TNM, T)	Tumor Size (longest dimension)	Lymph Node Involvement (TNM, N)	Metastatic (TNM, M)	Metastatic Site	Survival after Diagnosis	Survival after Stage IV Diagnosis
**Staining Intensity**	No 0.179	No 0.505	N.D.	No 0.645	N.D.	No 0.903	N.D.	No 0.461	N.D.	N.D.	N.D.	No 0.991	N.D.
**Coverage Score**	No 0.355	No 0.385	N.D.	No 0.532	N.D.	No 0.903	N.D.	No 0.938	N.D.	N.D.	N.D.	No 0.859	N.D.
**Total Staining Score**	No 0.117	No 0.701	N.D.	No 0.902	N.D.	No 0.807	N.D.	No 0.823	N.D.	N.D.	N.D.	No 0.506	N.D.

SI Figure 14. Correlation summary of CCK2R in GIST primary tumors. IHC was performed on GIST primary tumor tissue sections using a monoclonal antibody raised against CCK2R. The staining intensity, coverage score and total staining score were compared against available patient data. If appropriate, a spearman analysis was used to determine if any significant correlation exists while a 1-way ANOVA or t-test was used to determine if a significant difference exists between groups. Whether the test was statistically significant and the p-value is listed. N.A. – not applicable (this statistical test was not applicable to this data set). N.D. – not determined (this statistical test could not be performed, generally due to a lack of the number of samples within a group or all data was in a single group).

GIST primary tumor
Staining Intensity

GIST Cancer

	n	Average	St. Dev	St. Error
Staining Intensity	67	1.76	0.70	0.09

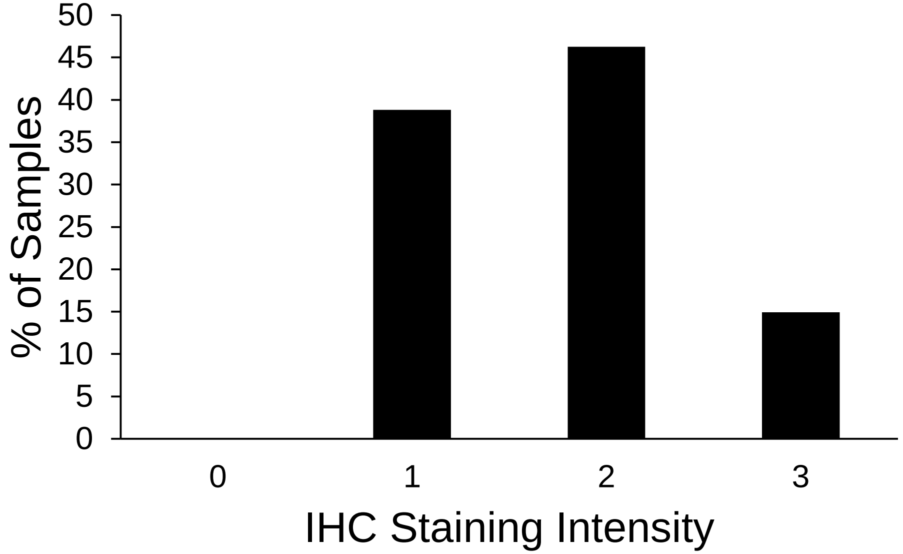

Staining Intensity	0	1	2	3
n	0	26	31	10
%	0.00	38.81	46.27	14.93

Normal Stomach

	n	Average	St. Dev	St. Error
Staining Intensity	3	1.00	0.00	0.00

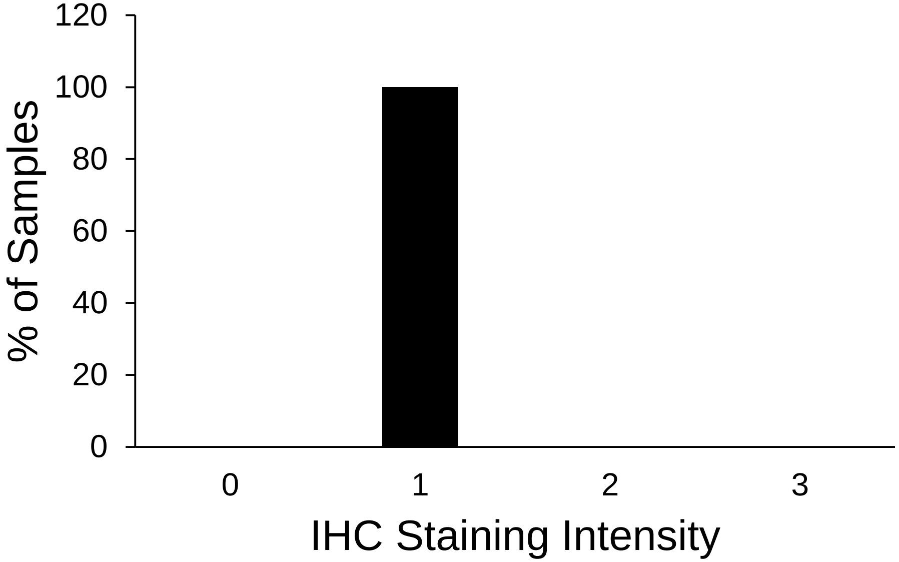

Staining Intensity	0	1	2	3
n	0	3	0	0
%	0.00	100.00	0.00	0.00

SI Figure 15. CCK2R Staining Intensity for cancer and normal tissue from the gastro-intestinal tract and stomach, respectively. IHC was performed on tissue sections using a monoclonal antibody raised against CCK2R. The intensity of staining was graded on a scale of 0 to 3 and plotted.

GIST primary tumor
Staining Intensity Correlations

GIST - primary tumor - Staining Intensity

	Sex	Age at Diagnosis	Primary Tumor Type	Primary Tumor Site	Stage	Grade	Tumor Size (TNM, T)	Tumor Size (longest dimension)	Lymph Node Involvement (TNM, N)	Metastatic (TNM, M)	Metastatic Site	Survival after Diagnosis	Survival after Stage IV Diagnosis
**Spearman Correlation**	N.A.	No 0.425	N.A.	N.A.	N.D.	No 0.860	N.D.	No 0.453	N.D.	N.D.	N.A.	No 0.524	N.D.
**ANOVA/t-test**	No 0.179	No 0.505	N.D.	No 0.645	N.D.	No 0.903	N.D.	No 0.461	N.D.	N.D.	N.D.	No 0.991	N.D.

SI Figure 16. Staining intensity correlation summary of CCK2R in GIST primary tumors. IHC was performed on GIST primary tumor tissue sections using a monoclonal antibody raised against CCK2R. The staining intensity was compared against available patient data. If appropriate, a spearman analysis was used to determine if any significant correlation exists while a 1-way ANOVA or t-test was used to determine if a significant difference exists between groups. Whether the test was statistically significant and the p-value is listed. N.A. – not applicable (this statistical test was not applicable to this data set). N.D. – not determined (this statistical test could not be performed, generally due to a lack of the number of samples within a group or all data was in a single group).

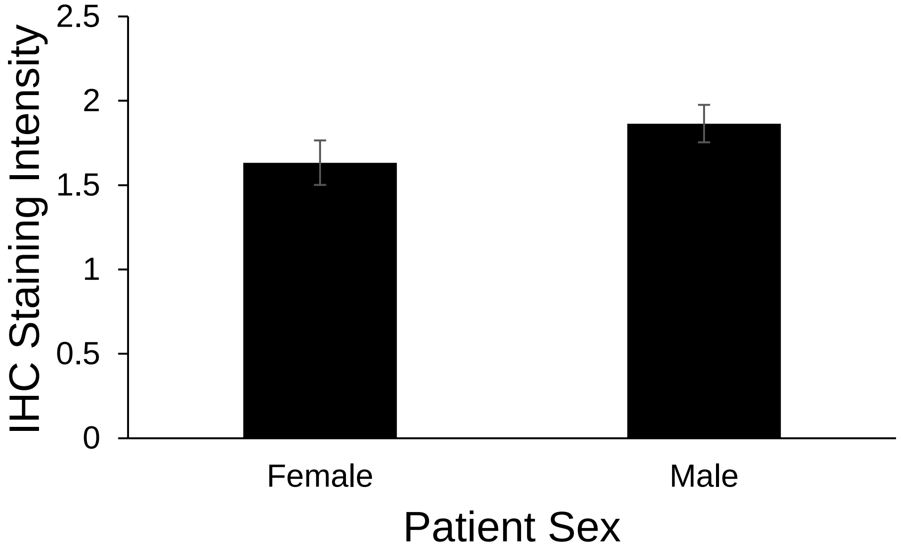

Spearman Analysis	
ρ (rho) =	-
t =	-
p-value =	-

	n	Average	St. Dev	St. Error
Female	30	1.63	0.72	0.13
Male	37	1.86	0.67	0.11

t-test

p-value = 0.1791

SI Figure 17. Correlation analysis of CCK2R staining intensity in GIST primary tumor versus patient sex. IHC was performed on GIST primary tumor tissue sections using a monoclonal antibody raised against CCK2R. The staining intensity was graded on a scale of 0 to 3 and plotted (error bars represent standard error of the mean). A t-test was used to determine if there were any significant differences between groups.

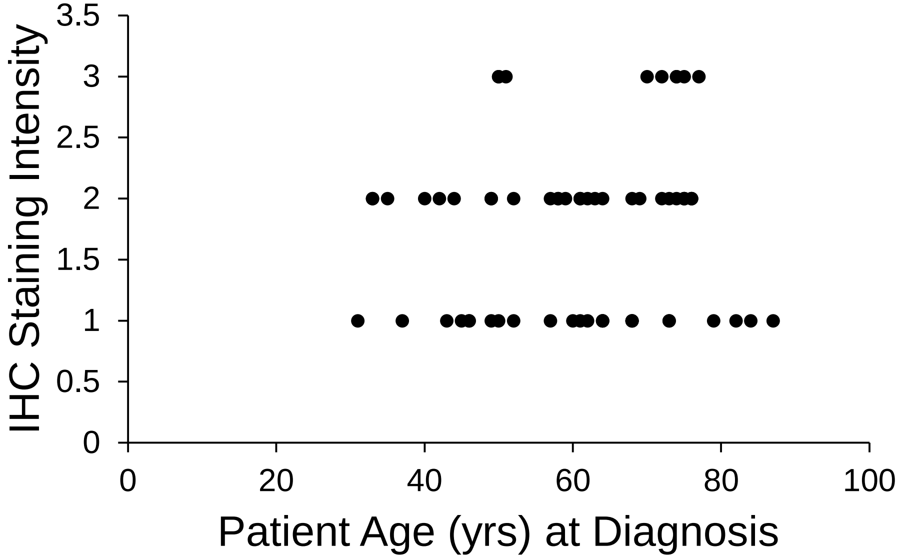

Spearman Analysis	
ρ (rho) =	0.1024
t =	0.8040
p-value =	0.4245

	n	Average	St. Dev	St. Error
0	0	-	-	-
1	25	60.52	14.86	2.97
2	29	59.62	14.03	2.61
3	9	65.89	11.83	3.94

1-Way Anova

	SS	df	MS	F	p
Between	275	2	137.6	0.692	0.505
Within	11,931	60	198.8		
Total	12,206	62			

SI Figure 18. Correlation analysis of CCK2R staining intensity in GIST primary tumor versus patient age at diagnosis. IHC was performed on GIST primary tumor tissue sections using a monoclonal antibody raised against CCK2R. The staining intensity was graded on a scale of 0 to 3 and plotted. A Spearman analysis was used to determine if there was a statistically significant correlation and a 1-way ANOVA was used to determine if there were any significant differences between groups.

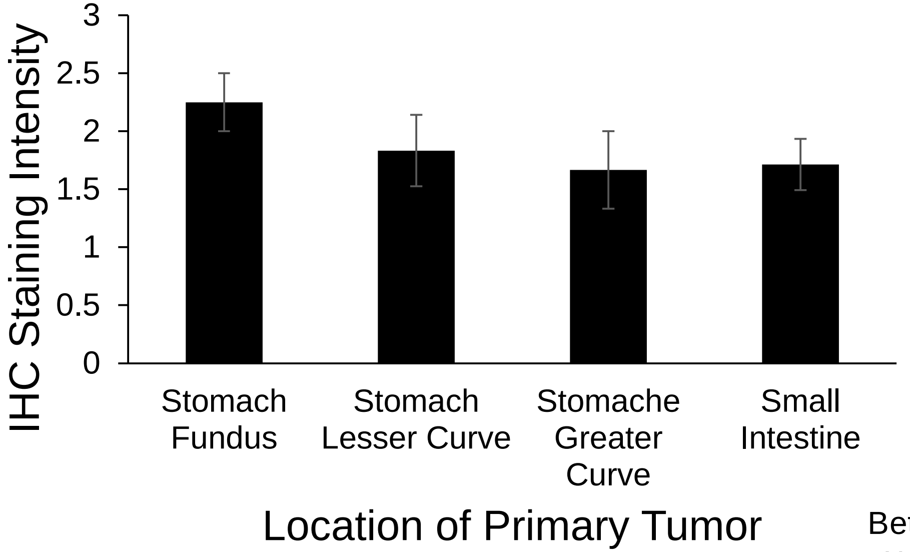

Spearman Analysis

ρ (rho) =	-
t =	-
p-value =	-

	n	Average	St. Dev	St. Error
Stomach Fundus	4	2.25	0.50	0.25
Stomach Lesser Curve	6	1.83	0.75	0.31
Stomache Greater Curve	6	1.67	0.82	0.33
Small Intestine	14	1.71	0.83	0.22

1-Way Anova

	SS	df	MS	F	p
Between	1.03	3	0.343	0.562	0.645
Within	15.88	26	0.611		
Total	16.91	29			

SI Figure 19. Correlation analysis of CCK2R staining intensity in GIST primary tumor versus location of primary tumor. IHC was performed on GIST primary tumor tissue sections using a monoclonal antibody raised against CCK2R. The staining intensity was graded on a scale of 0 to 3 and plotted (error bars represent standard error of the mean). A 1-way ANOVA was used to determine if there were any significant differences between groups.

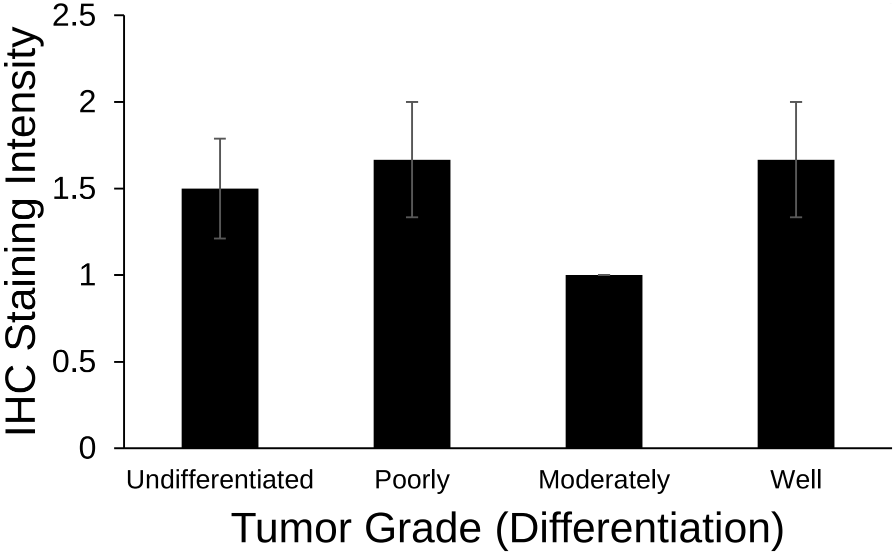

Spearman Analysis

ρ (rho) = 0.0603
t = 0.1811
p-value = 0.8599

	n	Average	St. Dev	St. Error
Undifferentiated	4	1.50	0.58	0.29
Poorly	3	1.67	0.58	0.33
Moderately	1	1.00	-	-
Well	3	1.67	0.58	0.33

1-Way Anova

	SS	df	MS	F	p
Between	0.07	2	0.035	0.103	0.903
Within	2.36	7	0.336		
Total	2.42	9			

SI Figure 20. Correlation analysis of CCK2R staining intensity in GIST primary tumor versus primary tumor grade. IHC was performed on GIST primary tumor tissue sections using a monoclonal antibody raised against CCK2R. The staining intensity was graded on a scale of 0 to 3 and plotted (error bars represent standard error of the mean). A Spearman analysis was used to determine if there was a statistically significant correlation and a 1-way ANOVA was used to determine if there were any significant differences between groups.

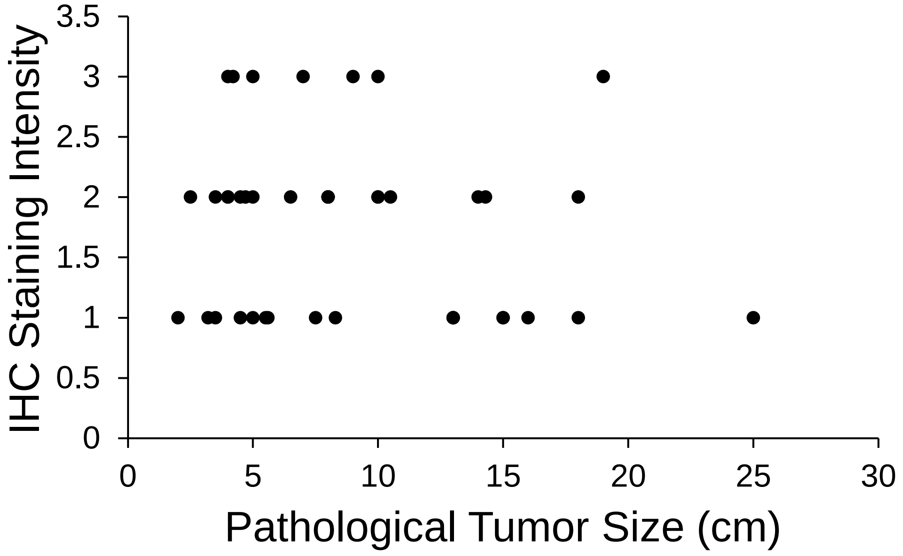

Spearman Analysis

ρ (rho) =	-0.1190
t =	-0.7577
p-value =	0.4529

	n	Average	St. Dev	St. Error
0	0	-	-	-
1	17	10.18	6.41	1.56
2	18	7.97	4.25	1.00
3	7	8.31	5.26	1.99

1-Way Anova

	SS	df	MS	F	p
Between	45.8	2	22.92	0.791	0.461
Within	1,130.5	39	28.99		
Total	1,176.3	41			

SI Figure 21. Correlation analysis of CCK2R staining intensity in GIST primary tumor versus primary tumor size (length of longest side). IHC was performed on GIST primary tumor tissue sections using a monoclonal antibody raised against CCK2R. The staining intensity was graded on a scale of 0 to 3 and plotted. A Spearman analysis was used to determine if there was a statistically significant correlation and a 1-way ANOVA was used to determine if there were any significant differences between groups.

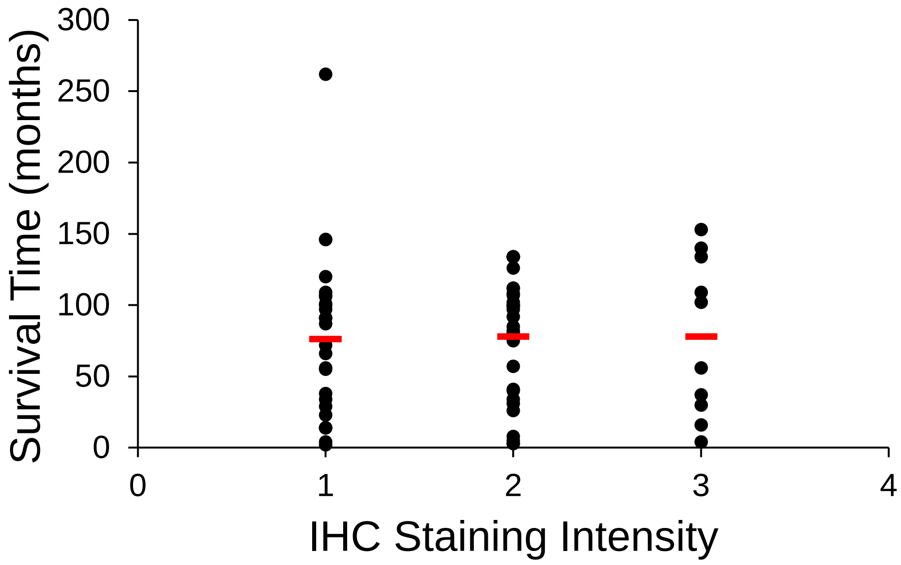

Spearman Analysis		
	ρ (rho) =	0.0793
	t =	0.6411
	p-value =	0.5237

	n	Average	St. Dev	St. Error
0	0	-	-	-
1	26	76.31	57.57	11.29
2	31	77.97	42.07	7.56
3	10	78.10	55.71	17.62

1-Way Anova

	SS	df	MS	F	p
Between	46	2	23	0.009	0.991
Within	163,887	64	2,561		
Total	463,932	66			

SI Figure 22. Correlation analysis of CCK2R staining intensity in GIST primary tumor versus survival time after diagnosis. IHC was performed on GIST primary tumor tissue sections using a monoclonal antibody raised against CCK2R. The staining intensity was graded on a scale of 0 to 3 and plotted (red bars represent population mean). A Spearman analysis was used to determine if there was a statistically significant correlation and a 1-way ANOVA was used to determine if there were any significant differences between groups.

GIST primary tumor
Coverage Score Correlations

GIST Cancer

	n	Average	St. Dev	St. Error
Coverage Score	67	2.52	0.70	0.09

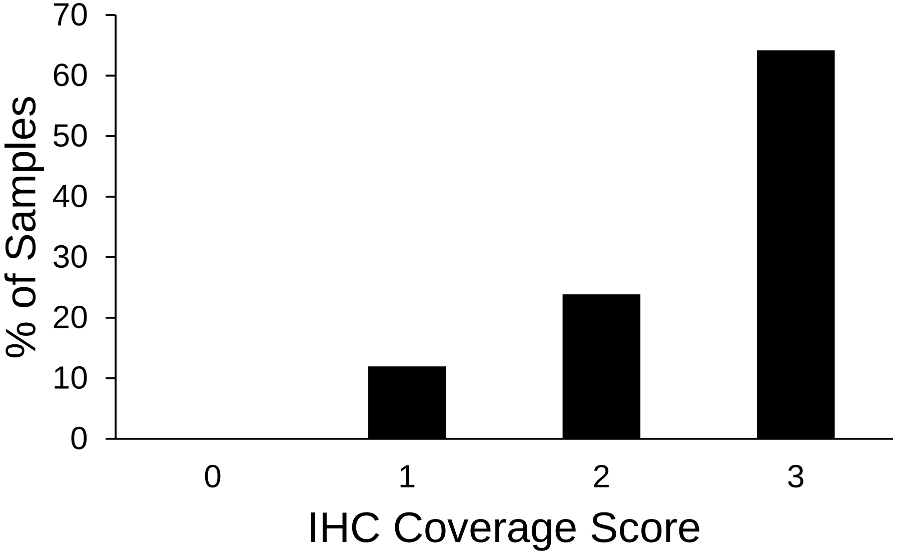

Coverage Score	0	1	2	3
n	0	8	16	43
%	0.00	11.94	23.88	64.18

Normal Stomach

	n	Average	St. Dev	St. Error
Coverage Score	3	2.33	0.58	0.33

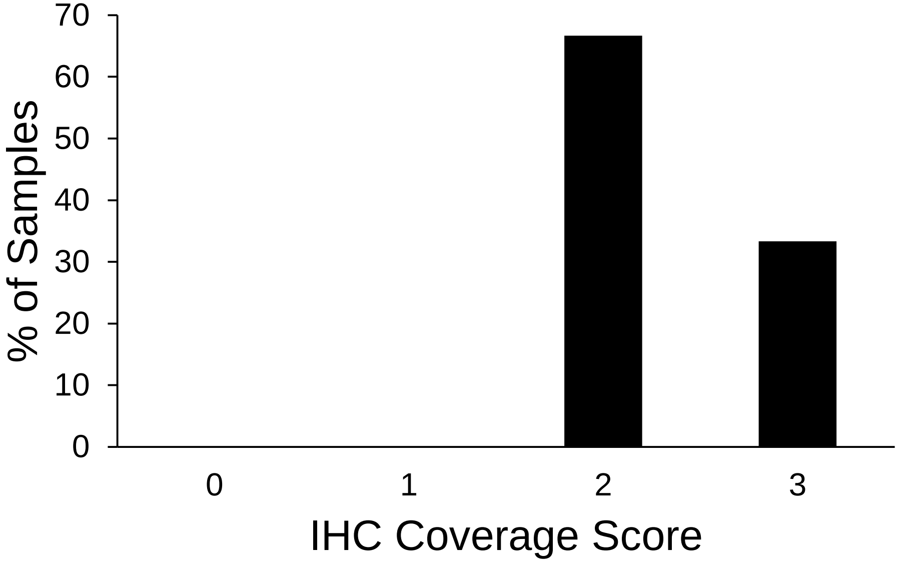

Coverage Score	0	1	2	3
n	0	0	2	1
%	0.00	0.00	66.67	33.33

SI Figure 23. CCK2R Coverage Score for cancer and normal tissue from the gastro-intestinal tract and stomach, respectively. IHC was performed on tissue sections using a monoclonal antibody raised against CCK2R. The area stained (coverage) was graded on a scale of 0 to 3 and plotted.

GIST - primary tumor - Coverage Score

	Sex	Age at Diagnosis	Primary Tumor Type	Primary Tumor Site	Stage	Grade	Tumor Size (TNM, T)	Tumor Size (longest dimension)	Lymph Node Involvement (TNM, N)	Metastatic (TNM, M)	Metastatic Site	Survival after Diagnosis	Survival after Stage IV Diagnosis
**Spearman Correlation**	N.A.	No 0.263	N.A.	N.A.	N.D.	No 0.860	N.D.	No 0.519	N.D.	N.D.	N.A.	No 0.961	N.D.
**ANOVA/t-test**	No 0.355	No 0.385	N.D.	No 0.532	N.D.	No 0.903	N.D.	No 0.938	N.D.	N.D.	N.D.	No 0.859	N.D.

SI Figure 24. Coverage score correlation summary of CCK2R in GIST primary tumors. IHC was performed on GIST primary tumor tissue sections using a monoclonal antibody raised against CCK2R. The coverage score was compared against available patient data. If appropriate, a spearman analysis was used to determine if any significant correlation exists while a 1-way ANOVA or t-test was used to determine if a significant difference exists between groups. Whether the test was statistically significant and the p-value is listed. N.A. – not applicable (this statistical test was not applicable to this data set). N.D. – not determined (this statistical test could not be performed, generally due to a lack of the number of samples within a group or all data was in a single group).

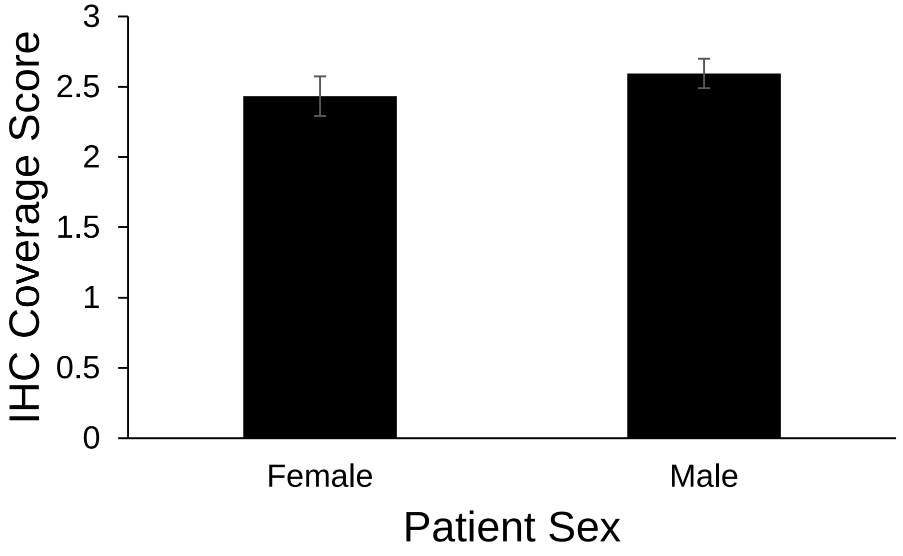

Spearman Analysis ρ (rho) = -
t = -
p-value = -

	n	Average	St. Dev	St. Error
Female	30	2.43	0.77	0.14
Male	37	2.59	0.64	0.11

t-test

p-value = 0.3551

SI Figure 25. Correlation analysis of CCK2R coverage score in GIST primary tumor versus patient sex. IHC was performed on GIST primary tumor tissue sections using a monoclonal antibody raised against CCK2R. The coverage score was graded on a scale of 0 to 3 and plotted (error bars represent standard error of the mean). A t-test was used to determine if there were any significant differences between groups.

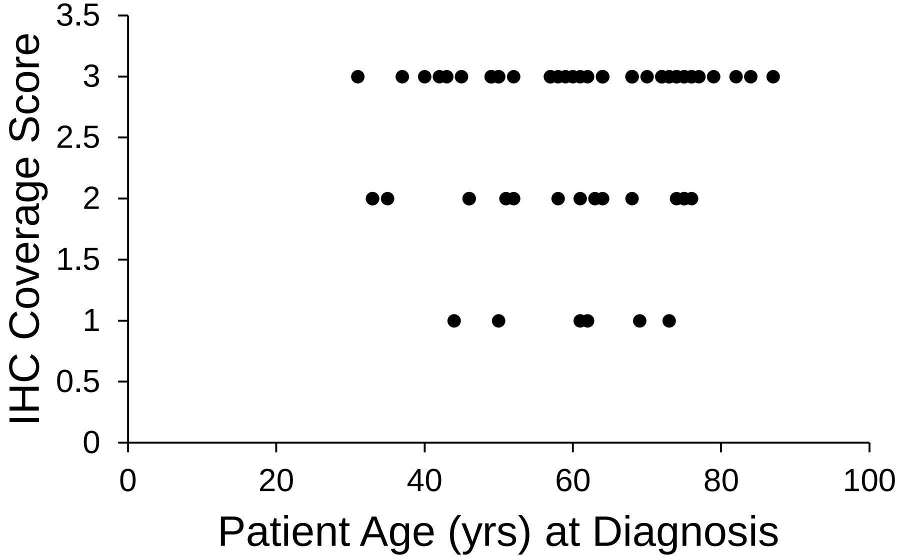

Spearman Analysis		
	ρ (rho) =	0.1430
	t =	1.1288
	p-value =	0.2633

	n	Average	St. Dev	St. Error
0	0	-	-	-
1	6	59.83	11.05	4.51
2	16	56.88	15.19	3.80
3	41	62.59	13.92	2.17

1-Way Anova

	SS	df	MS	F	p
Between	383	2	191.3	0.971	0.385
Within	11,822	60	197.0		
Total	12,205	62			

SI Figure 26. Correlation analysis of CCK2R coverage score in GIST primary tumor versus patient age at diagnosis. IHC was performed on GIST primary tumor tissue sections using a monoclonal antibody raised against CCK2R. The coverage score was graded on a scale of 0 to 3 and plotted. A Spearman analysis was used to determine if there was a statistically significant correlation and a 1-way ANOVA was used to determine if there were any significant differences between groups.

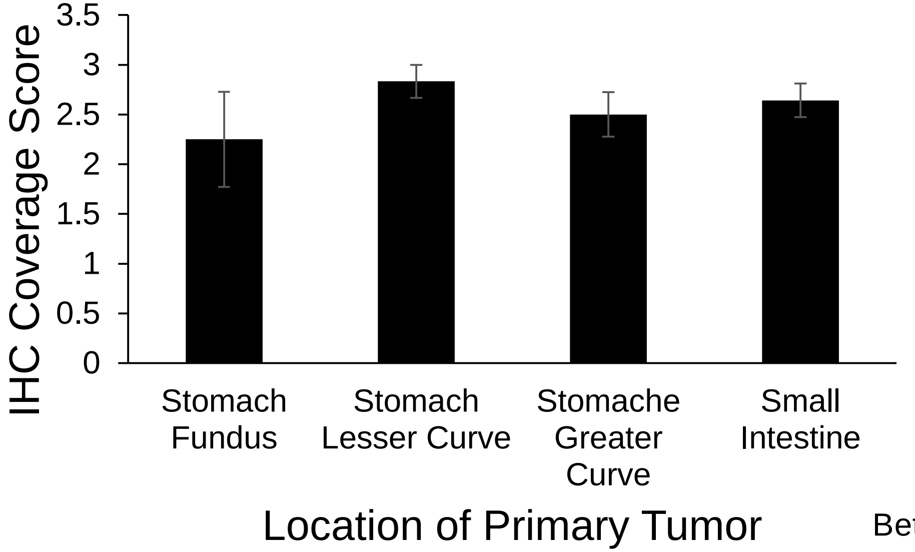

Spearman Analysis

ρ (rho) =	-
t =	-
p-value =	-

	n	Average	St. Dev	St. Error
Stomach Fundus	4	2.25	0.96	0.48
Stomach Lesser Curve	6	2.83	0.41	0.17
Stomache Greater Curve	6	2.50	0.55	0.22
Small Intestine	14	2.64	0.63	0.17

1-Way Anova

	SS	df	MS	F	p
Between	0.89	3	0.297	0.750	0.532
Within	10.28	26	0.395		
Total	11.17	29			

SI Figure 27. Correlation analysis of CCK2R coverage score in GIST primary tumor versus location of primary tumor. IHC was performed on GIST primary tumor tissue sections using a monoclonal antibody raised against CCK2R. The coverage score was graded on a scale of 0 to 3 and plotted (error bars represent standard error of the mean). A 1-way ANOVA was used to determine if there were any significant differences between groups.

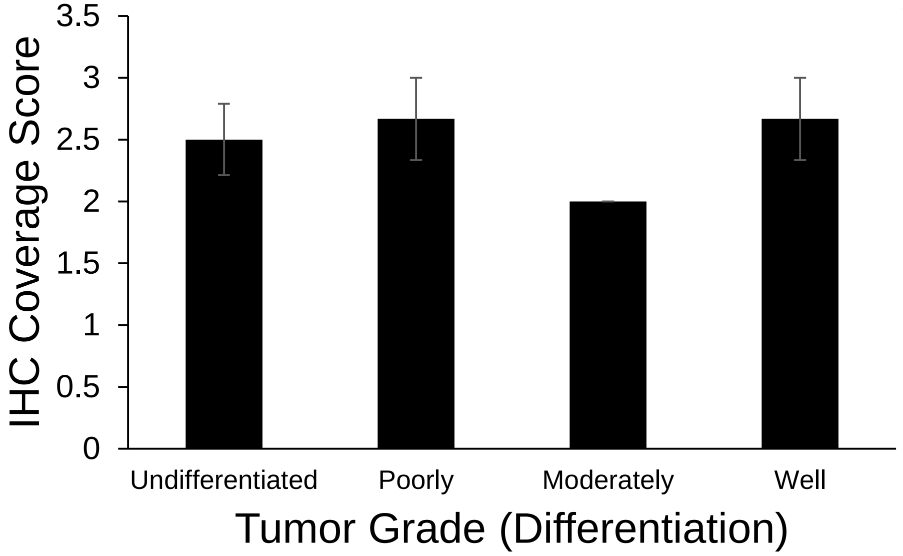

Spearman Analysis

ρ (rho) =	0.0603
t =	0.1811
p-value =	0.8599

	n	Average	St. Dev	St. Error
Undifferentiated	4	2.50	0.58	0.29
Poorly	3	2.67	0.58	0.33
Moderately	1	2.00	-	-
Well	3	2.67	0.58	0.33

1-Way Anova

	SS	df	MS	F	p
Between	0.07	2	0.035	0.103	0.903
Within	2.36	7	0.336		
Total	2.42	9			

SI Figure 28. Correlation analysis of CCK2R coverage score in GIST primary tumor versus primary tumor grade. IHC was performed on GIST primary tumor tissue sections using a monoclonal antibody raised against CCK2R. The coverage score was graded on a scale of 0 to 3 and plotted (error bars represent standard error of the mean). A Spearman analysis was used to determine if there was a statistically significant correlation and a 1-way ANOVA was used to determine if there were any significant differences between groups.

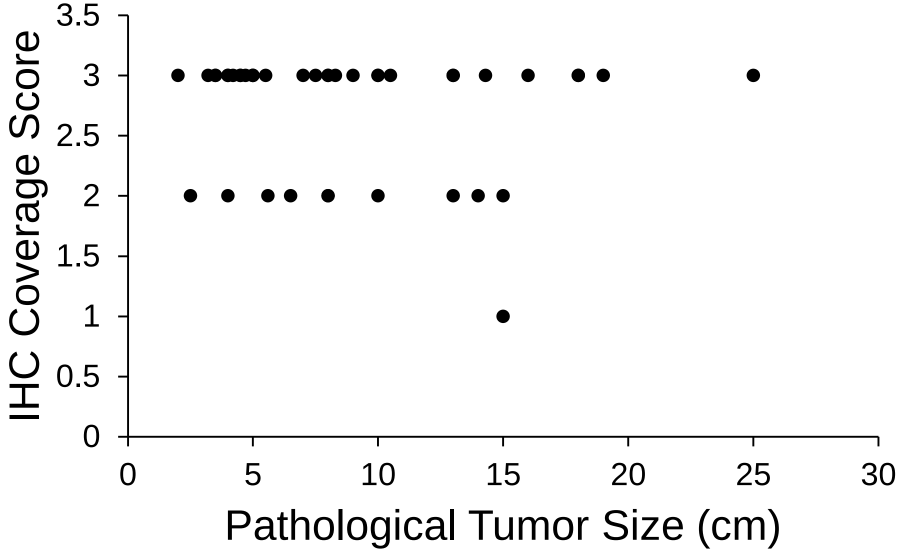

Spearman Analysis

ρ (rho) = -0.1023
t = -0.6506
p-value = 0.5189

	n	Average	St. Dev	St. Error
0	0	-	-	-
1	1	15.00	-	-
2	10	8.66	4.27	1.35
3	31	8.81	5.70	1.02

t-test

p-value = 0.9384

SI Figure 29. Correlation analysis of CCK2R coverage score in GIST primary tumor versus primary tumor size (length of longest side). IHC was performed on GIST primary tumor tissue sections using a monoclonal antibody raised against CCK2R. The coverage score was graded on a scale of 0 to 3 and plotted. A Spearman analysis was used to determine if there was a statistically significant correlation and a t-test was used to determine if there were any significant differences between groups.

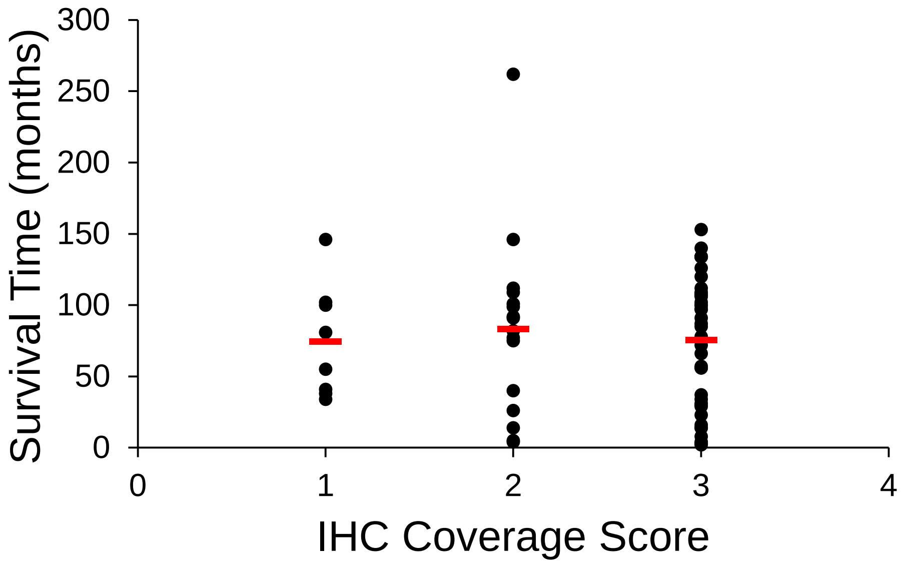

Spearman Analysis

ρ (rho) = 0.0061
t = 0.0491
p-value = 0.9610

	n	Average	St. Dev	St. Error
0	0	-	-	-
1	7	74.63	39.71	15.01
2	16	83.44	63.53	15.88
3	43	75.58	46.70	7.12

1-Way Anova

	SS	df	MS	F	p
Between	780	2	390	0.152	0.859
Within	161,600	63	265		
Total	162,379	65			

SI Figure 30. Correlation analysis of CCK2R coverage score in GIST primary tumor versus survival time after diagnosis. IHC was performed on GIST primary tumor tissue sections using a monoclonal antibody raised against CCK2R. The coverage score was graded on a scale of 0 to 3 and plotted (red bars represent population mean). A Spearman analysis was used to determine if there was a statistically significant correlation and a 1-way ANOVA was used to determine if there were any significant differences between groups.

GIST primary tumor
Total Staining Score Correlations

GIST Cancer

	n	Average	St. Dev	St. Error
Total Score	67	4.49	2.31	0.28

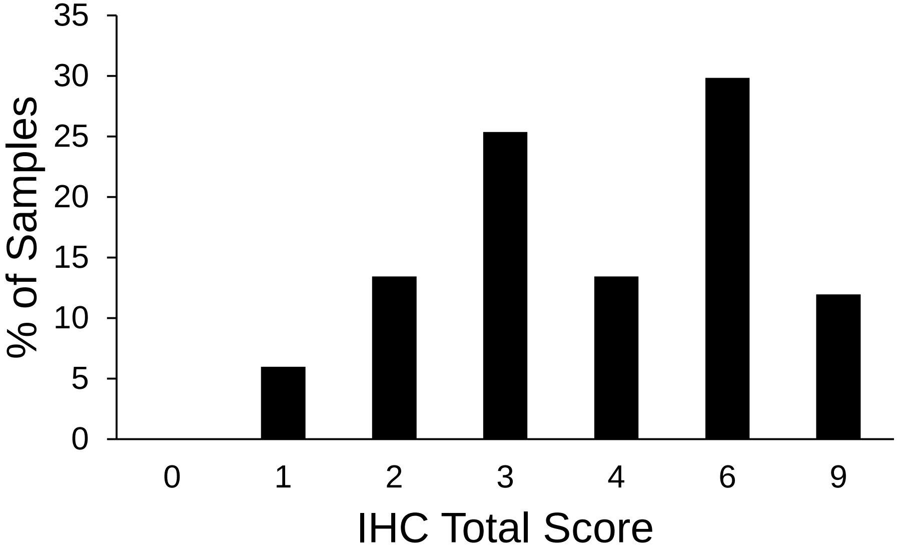

Total Staining Score

	0	1	2	3	4	6	9
n	0	4	9	17	9	20	8
%	0.00	5.97	13.43	25.37	13.43	29.85	11.94

Normal Stomach

	n	Average	St. Dev	St. Error
Total Score	3	2.33	0.58	0.33

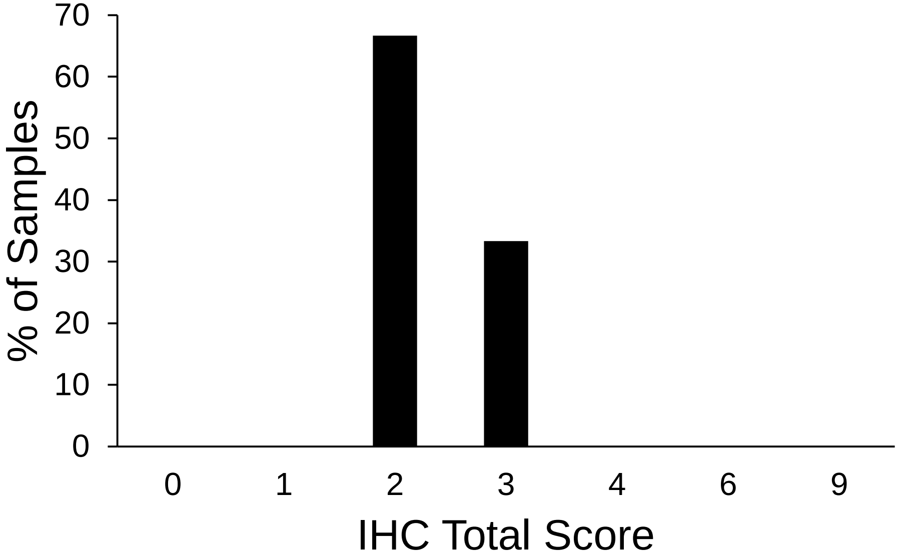

Total Staining Score

	0	1	2	3	4	6	9
n	0	0	2	1	0	0	0
%	0.00	0.00	66.67	33.33	0.00	0.00	0.00

SI Figure 31. CCK2R Total Staining Score for cancer and normal tissue from the gastro-intestinal tract and stomach, respectively. IHC was performed on tissue sections using a monoclonal antibody raised against CCK2R. The staining intensity and coverage score was multiplied to obtain the total staining score.

GIST - primary tumor - Total Staining Score

	Sex	Age at Diagnosis	Primary Tumor Type	Primary Tumor Site	Stage	Grade	Tumor Size (TNM, T)	Tumor Size (longest dimension)	Lymph Node Involvement (TNM, N)	Metastatic (TNM, M)	Metastatic Site	Survival after Diagnosis	Survival after Stage IV Diagnosis
**Spearman Correlation**	N.A.	No<br>0.247	N.A.	N.A.	N.D.	No<br>0.834	N.D.	No<br>0.357	N.D.	N.D.	N.A.	No<br>0.597	N.D.
**ANOVA/t-test**	No<br>0.117	No<br>0.701	N.D.	No<br>0.902	N.D.	No<br>0.807	N.D.	No<br>0.823	N.D.	N.D.	N.D.	No<br>0.506	N.D.

SI Figure 32. Total staining score correlation summary of CCK2R in GIST primary tumors. IHC was performed on GIST primary tumor tissue sections using a monoclonal antibody raised against CCK2R. The total staining score was compared against available patient data. If appropriate, a spearman analysis was used to determine if any significant correlation exists while a 1-way ANOVA or t-test was used to determine if a significant difference exists between groups. Whether the test was statistically significant and the p-value is listed. N.A. – not applicable (this statistical test was not applicable to this data set). N.D. – not determined (this statistical test could not be performed, generally due to a lack of the number of samples within a group or all data was in a single group).

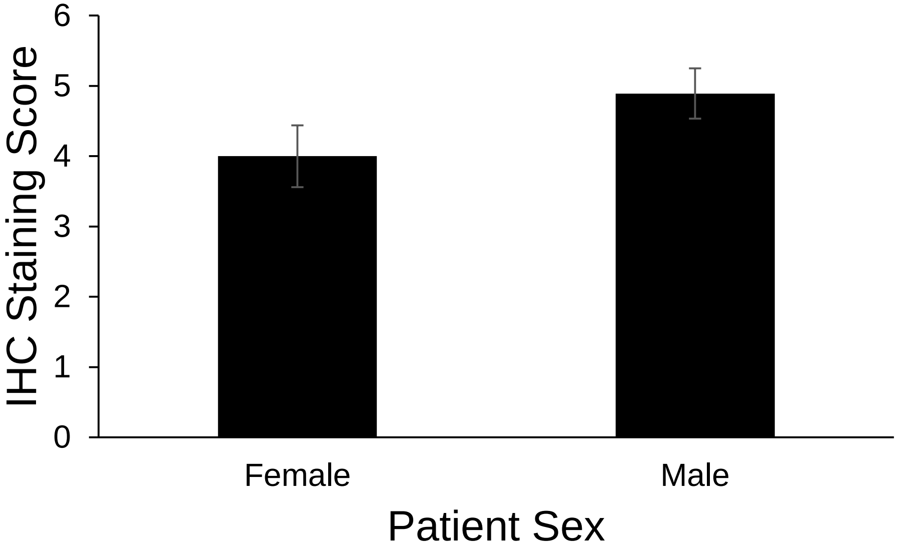

Spearman Analysis

ρ (rho) = -
t = -
p-value = -

	n	Average	St. Dev	St. Error
Female	30	4.00	2.41	0.44
Male	37	4.89	2.18	0.36

t-test

p-value = 0.1171

SI Figure 33. Correlation analysis of CCK2R total staining score in GIST primary tumor versus patient sex. IHC was performed on GIST primary tumor tissue sections using a monoclonal antibody raised against CCK2R. The total staining score was derived by multiplying the staining intensity with the coverage score (error bars represent standard error of the mean). A t-test was used to determine if there were any significant differences between groups.

Spearman Analysis		
	ρ (rho) =	0.1481
	t =	1.1699
	p-value =	0.2465

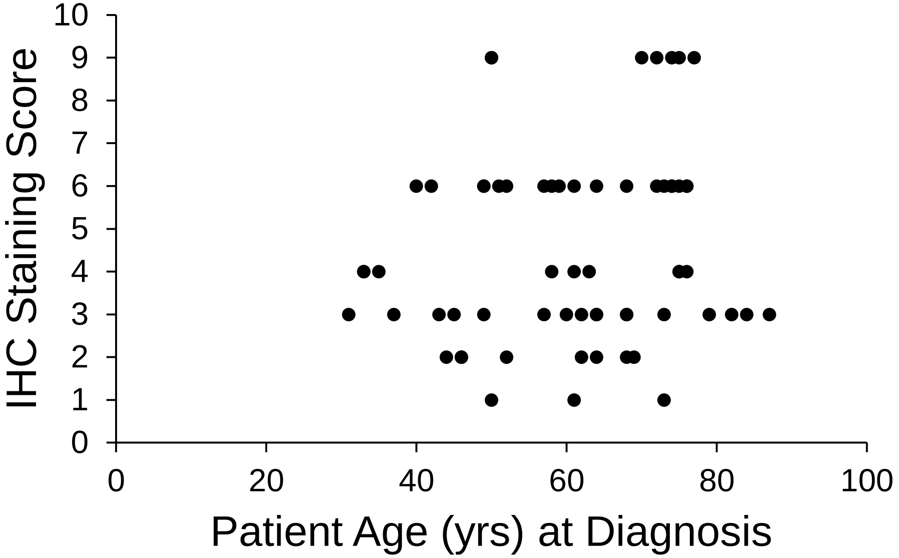

	n	Average	St. Dev	St. Error
0	0	-	-	-
1	3	61.33	11.50	6.64
2	8	56.38	10.50	3.71
3	17	61.94	16.65	4.04
4	9	56.56	18.36	6.12
6	19	61.58	12.02	2.76
9	7	66.86	11.73	4.43

1-Way Anova

	SS	df	MS	F	p
Between	609	5	121.9	0.599	0.701
Within	11,595	57	203.4		
Total	12,204	62			

SI Figure 34. Correlation analysis of CCK2R total staining score in GIST primary tumor versus patient age at diagnosis. IHC was performed on GIST primary tumor tissue sections using a monoclonal antibody raised against CCK2R. The total staining score was derived by multiplying the staining intensity with the coverage score. A Spearman analysis was used to determine if there was a statistically significant correlation and a 1-way ANOVA was used to determine if there were any significant differences between groups.

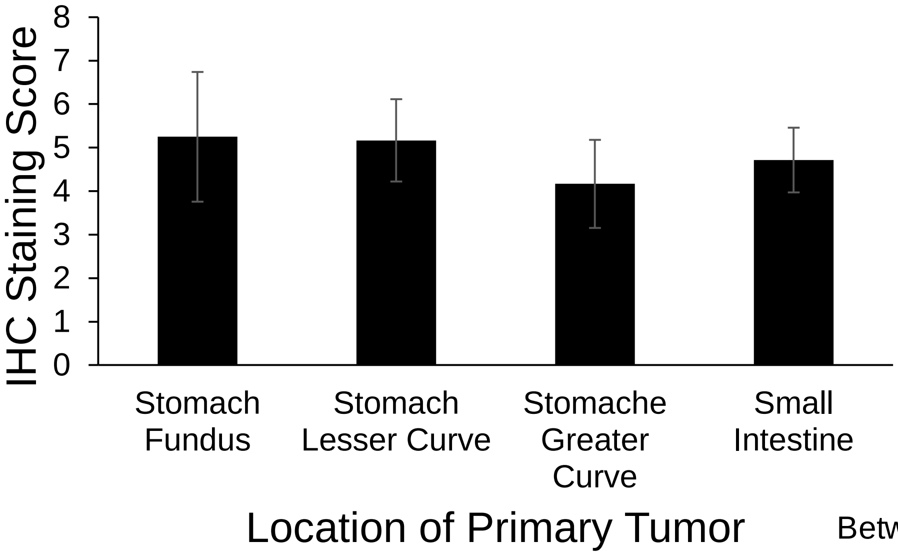

Spearman Analysis

ρ (rho) =	-
t =	-
p-value =	-

	n	Average	St. Dev	St. Error
Stomach Fundus	4	5.25	2.99	1.49
Stomach Lesser Curve	6	5.17	2.32	0.95
Stomache Greater Curve	6	4.17	2.48	1.01
Small Intestine	14	4.71	2.79	0.74

1-Way Anova

	SS	df	MS	F	p
Between	4.09	3	1.364	0.191	0.902
Within	185.68	26	7.141		
Total	189.77	29			

SI Figure 35. Correlation analysis of CCK2R total staining score in GIST primary tumor versus location of primary tumor. IHC was performed on GIST primary tumor tissue sections using a monoclonal antibody raised against CCK2R. The total staining score was derived by multiplying the staining intensity with the coverage score (error bars represent standard error of the mean). A 1-way ANOVA was used to determine if there were any significant differences between groups.

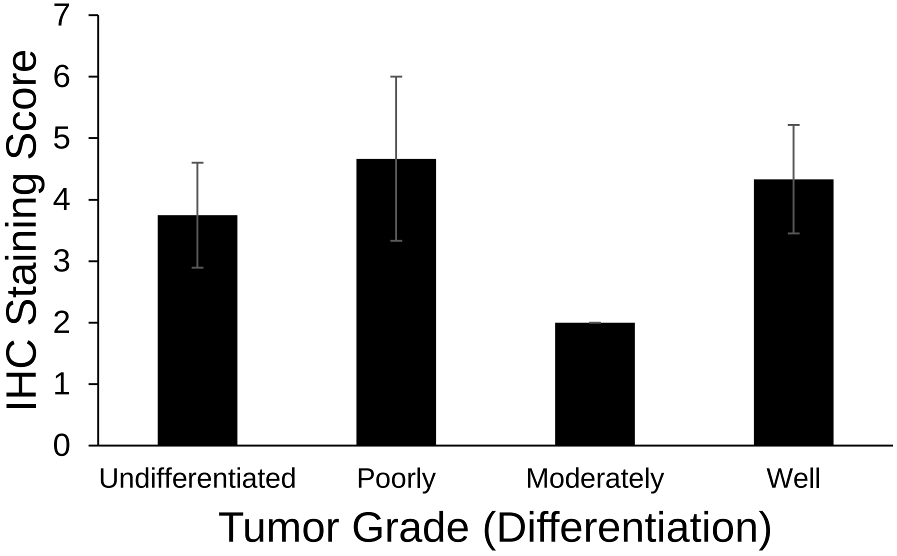

Spearman Analysis

ρ (rho) =	0.0714
t =	0.2148
p-value =	0.8342

	n	Average	St. Dev	St. Error
Undifferentiated	4	3.75	1.71	0.85
Poorly	3	4.67	2.31	1.33
Moderately	1	2.00	-	-
Well	3	4.33	1.53	0.88

1-Way Anova

	SS	df	MS	F	p
Between	1.52	2	0.762	0.221	0.807
Within	24.13	7	3.447		
Total	25.65	9			

SI Figure 36. Correlation analysis of CCK2R total staining score in GIST primary tumor versus primary tumor grade. IHC was performed on GIST primary tumor tissue sections using a monoclonal antibody raised against CCK2R. The total staining score was derived by multiplying the staining intensity with the coverage score (error bars represent standard error of the mean). A Spearman analysis was used to determine if there was a statistically significant correlation and a 1-way ANOVA was used to determine if there were any significant differences between groups.

Spearman Analysis

ρ (rho) =	-0.1458
t =	-0.9322
p-value =	0.3567

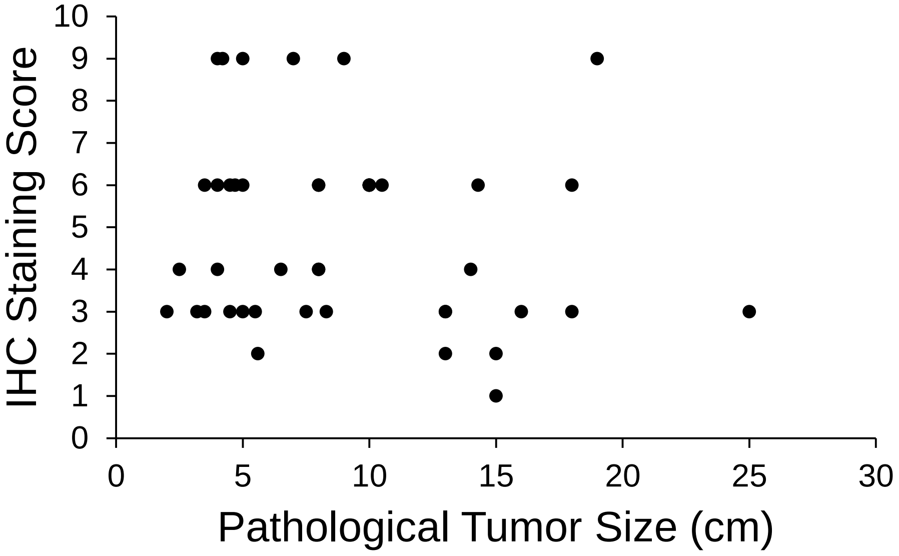

	n	Average	St. Dev	St. Error
0	0	-	-	-
1	1	15.00	-	-
2	3	11.20	4.95	2.86
3	13	9.58	6.94	1.92
4	6	7.17	4.01	1.64
6	13	8.50	4.31	1.20
9	6	8.03	5.70	2.33

1-Way Anova

	SS	df	MS	F	p
Between	45.8	4	11.46	0.378	0.823
Within	1,092.7	36	30.35		
Total	1,138.6	40			

SI Figure 37. Correlation analysis of CCK2R total staining score in GIST primary tumor versus primary tumor size (length of longest side). IHC was performed on GIST primary tumor tissue sections using a monoclonal antibody raised against CCK2R. The total staining score was derived by multiplying the staining intensity with the coverage score. A Spearman analysis was used to determine if there was a statistically significant correlation and a 1-way ANOVA was used to determine if there were any significant differences between groups.

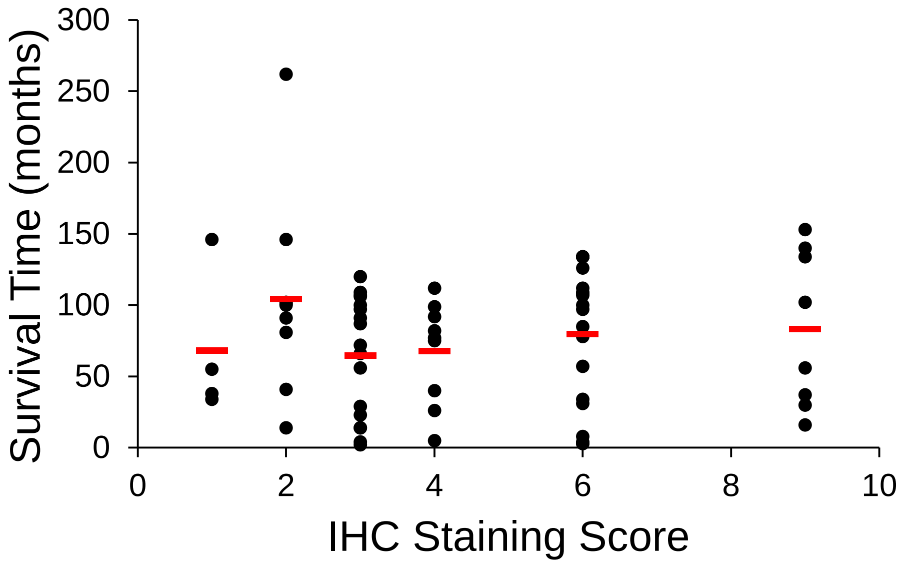

Spearman Analysis	
ρ (rho) =	0.0657
t =	0.5307
p-value =	0.5974

	n	Average	St. Dev	St. Error
0	0	-	-	-
1	4	68.25	52.63	26.31
2	9	104.22	70.29	23.43
3	17	64.53	41.75	10.13
4	9	67.56	35.91	11.97
6	20	79.90	49.06	10.97
9	8	83.50	55.10	19.48

1-Way Anova

	SS	df	MS	F	p
Between	10,918	5	2,184	0.870	0.506
Within	153,023	61	2,509		
Total	163,942	66			

SI Figure 38. Correlation analysis of CCK2R total staining score in GIST primary tumor versus survival time after diagnosis. IHC was performed on GIST primary tumor tissue sections using a monoclonal antibody raised against CCK2R. The total staining score was derived by multiplying the staining intensity with the coverage score (red bars represent population mean). A Spearman analysis was used to determine if there was a statistically significant correlation and a 1-way ANOVA was used to determine if there were any significant differences between groups.

GIST
metastatic tumors

GIST metastatic tumors
Overall Summary

GIST - metastatic tumor - Spearman Correlation

	Sex	Age at Diagnosis	Primary Tumor Type	Primary Tumor Site	Stage	Grade	Tumor Size (TNM, T)	Tumor Size (longest dimension)	Lymph Node Involvement (TNM, N)	Metastatic (TNM, M)	Metastatic Site	Survival after Diagnosis	Survival after Stage IV Diagnosis
**Staining Intensity**	N.A.	No 0.398	N.A.	N.A.	N.D.	N.D.	N.D.	No 0.150	N.D.	N.D.	N.A.	No 0.689	N.D.
**Coverage Score**	N.A.	No 0.724	N.A.	N.A.	N.D.	N.D.	N.D.	No 0.822	N.D.	N.D.	N.A.	No 0.625	N.D.
**Total Staining Score**	N.A.	No 0.418	N.A.	N.A.	N.D.	N.D.	N.D.	No 0.483	N.D.	N.D.	N.A.	No 0.617	N.D.

GIST - metastatic tumor - Spearman Correlation - ANOVA/t-test

	Sex	Age at Diagnosis	Primary Tumor Type	Primary Tumor Site	Stage	Grade	Tumor Size (TNM, T)	Tumor Size (longest dimension)	Lymph Node Involvement (TNM, N)	Metastatic (TNM, M)	Metastatic Site	Survival after Diagnosis	Survival after Stage IV Diagnosis
**Staining Intensity**	No 0.329	No 0.300	N.D.	N.D.	N.D.	N.D.	N.D.	No 0.144	N.D.	N.D.	N.D.	No 0.687	N.D.
**Coverage Score**	No 0.329	No 0.885	N.D.	N.D.	N.D.	N.D.	N.D.	No 0.508	N.D.	N.D.	N.D.	No 0.379	N.D.
**Total Staining Score**	No 0.430	N.D.	N.D.	N.D.	N.D.	N.D.	N.D.	N.D.	N.D.	N.D.	N.D.	N.D.	N.D.

SI Figure 39. Correlation summary of CCK2R in metastases of GIST. IHC was performed on metastases of GIST tissue sections using a monoclonal antibody raised against CCK2R. The staining intensity, coverage score and total staining score were compared against available patient data. If appropriate, a spearman analysis was used to determine if any significant correlation exists while a 1-way ANOVA or t-test was used to determine if a significant difference exists between groups. Whether the test was statistically significant and the p-value is listed. N.A. – not applicable (this statistical test was not applicable to this data set). N.D. – not determined (this statistical test could not be performed, generally due to a lack of the number of samples within a group or all data was in a single group).

GIST metastatic tumors
Staining Intensity

GIST Metastases

	n	Average	St. Dev	St. Error
Staining Intensity	12	1.92	1.00	0.29

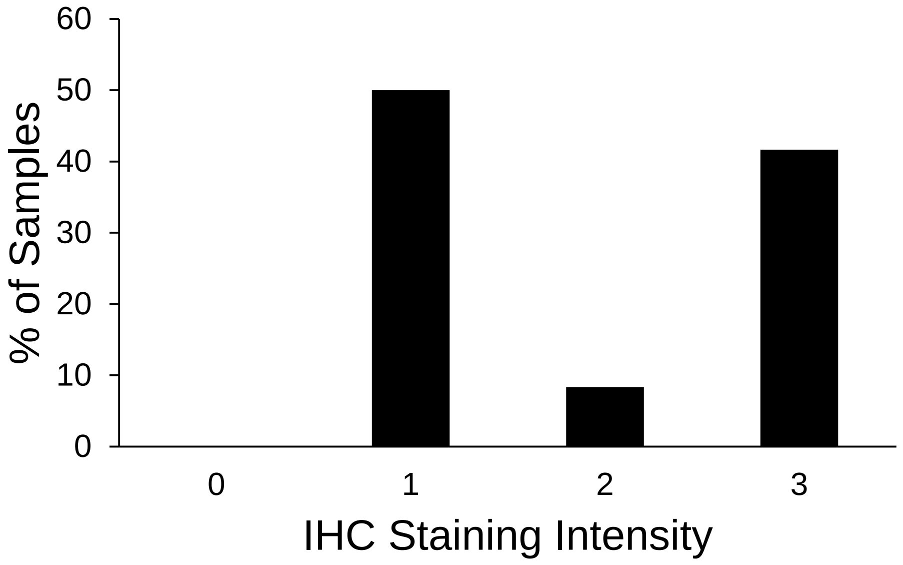

	Staining Intensity			
	0	1	2	3
n	0	6	1	5
%	0.00	50.00	8.33	41.67

SI Figure 40. CCK2R Staining Intensity for metastases from GIST primary tumors. IHC was performed on tissue sections using a monoclonal antibody raised against CCK2R. The intensity of staining was graded on a scale of 0 to 3 and plotted.

GIST - metastases - Staining Intensity

	Sex	Age at Diagnosis	Primary Tumor Type	Primary Tumor Site	Stage	Grade	Tumor Size (TNM, T)	Tumor Size (longest dimension)	Lymph Node Involvement (TNM, N)	Metastatic (TNM, M)	Metastatic Site	Survival after Diagnosis	Survival after Stage IV Diagnosis
**Spearman Correlation**	N.A.	No<br>0.398	N.A.	N.A.	N.D.	N.D.	N.D.	No<br>0.150	N.D.	N.D.	N.A.	No<br>0.689	N.D.
**ANOVA/t-test**	No<br>0.329	No<br>0.300	N.D.	N.D.	N.D.	N.D.	N.D.	No<br>0.144	N.D.	N.D.	N.D.	No<br>0.687	N.D.

SI Figure 41. Staining intensity correlation summary of CCK2R in metastases of GIST. IHC was performed on metastases of GIST tissue sections using a monoclonal antibody raised against CCK2R. The staining intensity was compared against available patient data. If appropriate, a spearman analysis was used to determine if any significant correlation exists while a 1-way ANOVA or t-test was used to determine if a significant difference exists between groups. Whether the test was statistically significant and the p-value is listed. N.A. – not applicable (this statistical test was not applicable to this data set). N.D. – not determined (this statistical test could not be performed, generally due to a lack of the number of samples within a group or all data was in a single group).

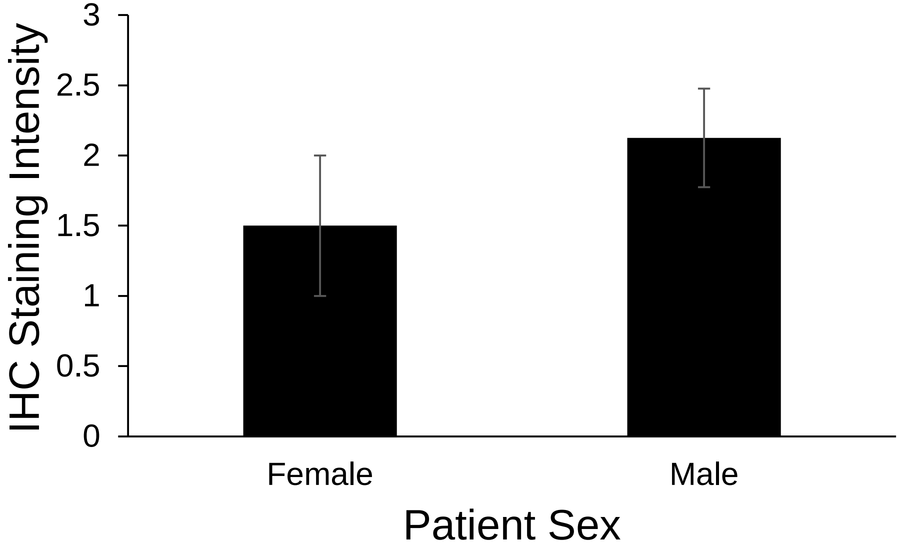

Spearman Analysis		
	ρ (rho) =	-
	t =	-
	p-value =	-

	n	Average	St. Dev	St. Error
Female	4	1.50	1.00	0.50
Male	8	2.13	0.99	0.35

t-test

p-value = 0.329

SI Figure 42. Correlation analysis of CCK2R staining intensity in metastases of GIST versus patient sex. IHC was performed on metastases of GIST tissue sections using a monoclonal antibody raised against CCK2R. The staining intensity was graded on a scale of 0 to 3 and plotted (error bars represent standard error of the mean). A t-test was used to determine if there were any significant differences between groups.

Spearman Analysis

ρ (rho) =	0.2991
t =	0.8865
p-value =	0.3984

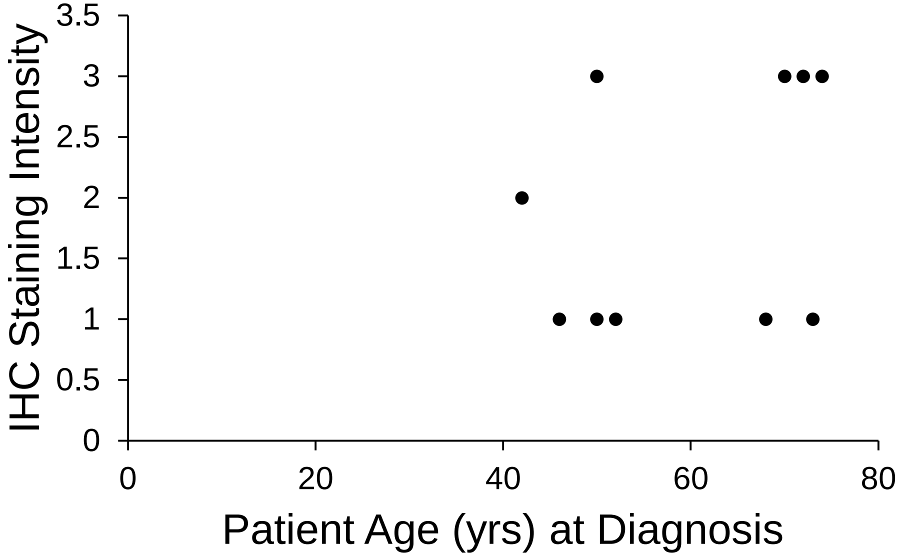

	n	Average	St. Dev	St. Error
0	0	-	-	-
1	5	57.80	11.92	5.33
2	1	42.00	-	-
3	4	66.50	11.12	5.56

t-test

p-value = 0.300

SI Figure 43. Correlation analysis of CCK2R staining intensity in metastases of GIST versus patient age at diagnosis. IHC was performed on metastases of GIST tissue sections using a monoclonal antibody raised against CCK2R. The staining intensity was graded on a scale of 0 to 3 and plotted. A Spearman analysis was used to determine if there was a statistically significant correlation and a t-test was used to determine if there were any significant differences between groups.

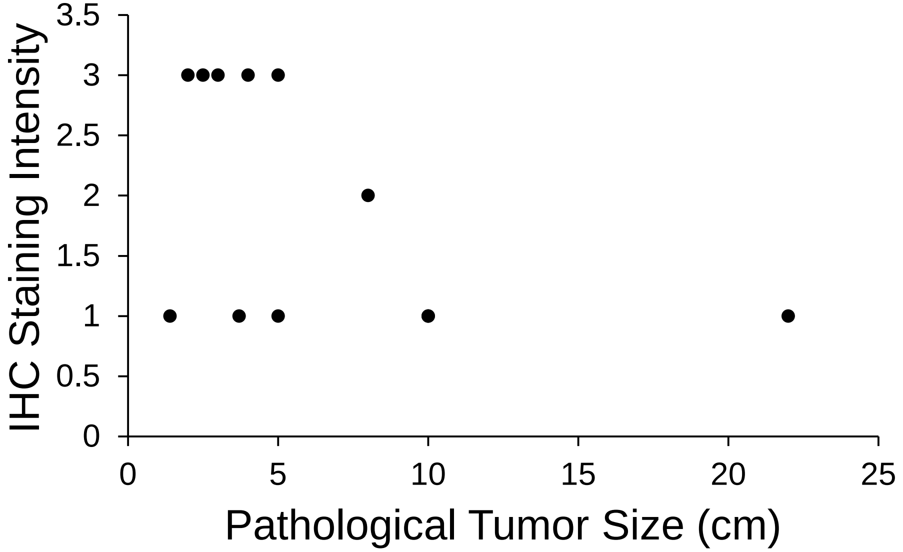

Spearman Analysis

ρ (rho) = -0.4392
t = -1.5461
p-value = 0.1504

	n	Average	St. Dev	St. Error
0	0	-	-	-
1	6	8.68	7.38	3.01
2	1	8.00	-	-
3	5	3.30	1.20	0.54

t-test

p-value = 0.144

SI Figure 44. Correlation analysis of CCK2R staining intensity in metastases of GIST versus size of primary tumor (length of longest side). IHC was performed on metastases of GIST tissue sections using a monoclonal antibody raised against CCK2R. The staining intensity was graded on a scale of 0 to 3 and plotted. A Spearman analysis was used to determine if there was a statistically significant correlation and a t-test was used to determine if there were any significant differences between groups.

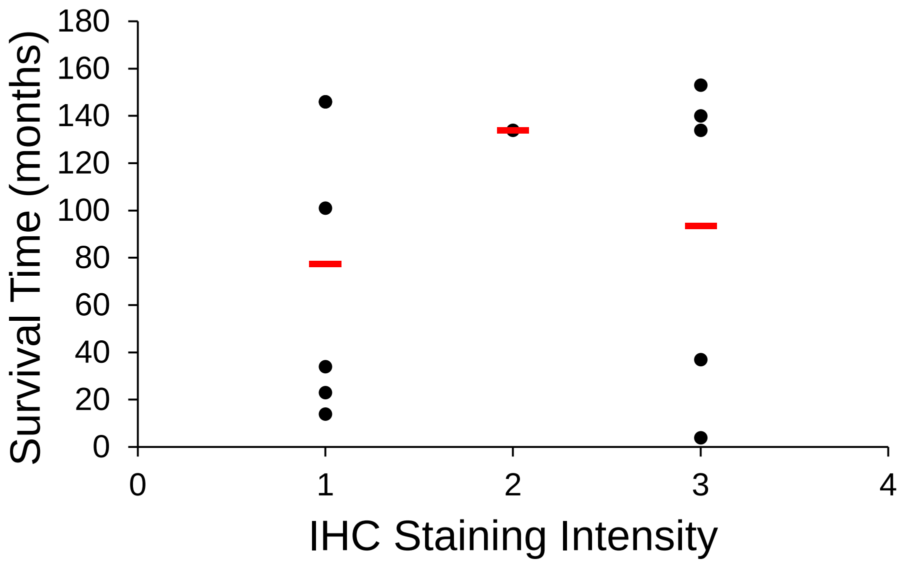

Spearman Analysis

ρ (rho) = 0.1288
t = 0.4109
p-value = 0.6891

	n	Average	St. Dev	St. Error
0	0	-	-	-
1	6	77.33	61.37	25.05
2	1	134.00	-	-
3	5	93.60	68.09	30.45

t-test

p-value = 0.6866

SI Figure 45. Correlation analysis of CCK2R staining intensity in metastases of GIST versus survival time after diagnosis. IHC was performed on metastases of GIST tissue sections using a monoclonal antibody raised against CCK2R. The staining intensity was graded on a scale of 0 to 3 and plotted (red bars represent population mean). A Spearman analysis was used to determine if there was a statistically significant correlation and a t-test was used to determine if there were any significant differences between groups.

GIST metastatic tumors Coverage Score Correlations

GIST Metastases

	n	Average	St. Dev	St. Error
Coverage Score	12	2.33	0.78	0.22

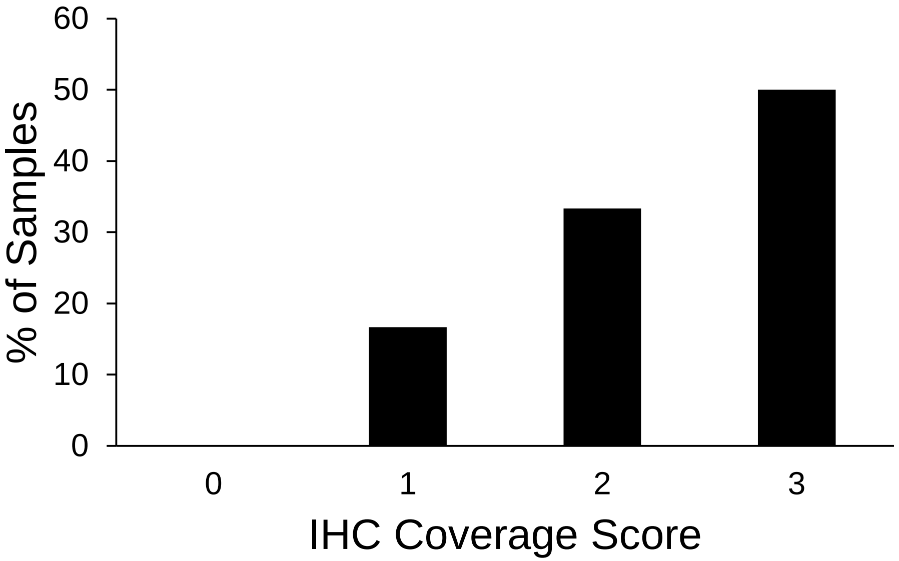

	Coverage Score			
	0	1	2	3
n	0	2	4	6
%	0.00	16.67	33.33	50.00

SI Figure 46. CCK2R Coverage Score for metastases from GIST primary tumors. IHC was performed on tissue sections using a monoclonal antibody raised against CCK2R. The area stained (coverage) was graded on a scale of 0 to 3 and plotted.

GIST - metastases - Coverage Score

	Sex	Age at Diagnosis	Primary Tumor Type	Primary Tumor Site	Stage	Grade	Tumor Size (TNM, T)	Tumor Size (longest dimension)	Lymph Node Involvement (TNM, N)	Metastatic (TNM, M)	Metastatic Site	Survival after Diagnosis	Survival after Stage IV Diagnosis
**Spearman Correlation**	N.A.	No 0.724	N.A.	N.A.	N.D.	N.D.	N.D.	No 0.822	N.D.	N.D.	N.A.	No 0.625	N.D.
**ANOVA/t-test**	No 0.329	No 0.885	N.D.	N.D.	N.D.	N.D.	N.D.	No 0.508	N.D.	N.D.	N.D.	No 0.379	N.D.

SI Figure 47. Coverage score correlation summary of CCK2R in metastases of GIST. IHC was performed on metastases of GIST tissue sections using a monoclonal antibody raised against CCK2R. The coverage score was compared against available patient data. If appropriate, a spearman analysis was used to determine if any significant correlation exists while a 1-way ANOVA or t-test was used to determine if a significant difference exists between groups. N.A. – not applicable (this statistical test was not applicable to this data set). Whether the test was statistically significant and the p-value is listed. N.D. – not determined (this statistical test could not be performed, generally due to a lack of the number of samples within a group or all data was in a single group).

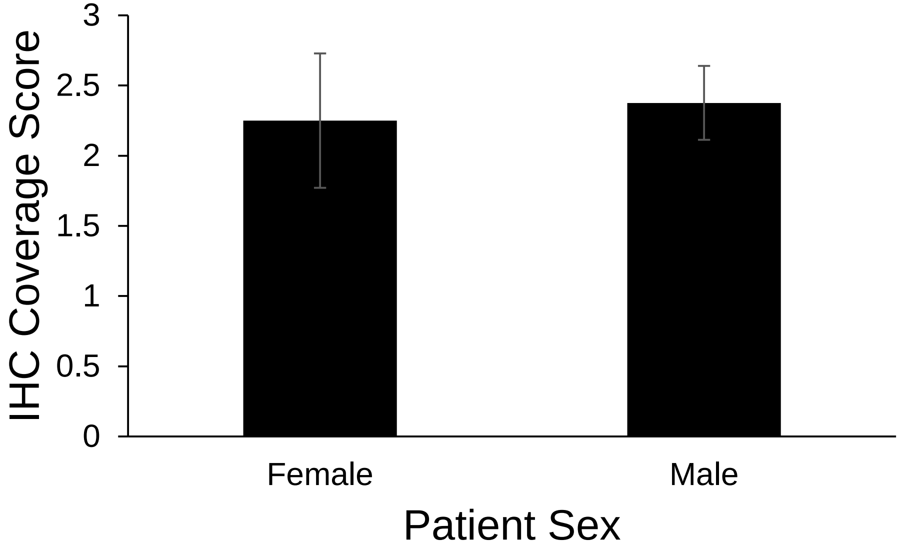

Spearman Analysis		
	ρ (rho) =	-
	t =	-
	p-value =	-

	n	Average	St. Dev	St. Error
Female	4	2.25	0.96	0.48
Male	8	2.38	0.74	0.26

t-test

p-value = 0.329

SI Figure 48. Correlation analysis of CCK2R coverage score in metastases of GIST versus patient sex. IHC was performed on metastases of GIST tissue sections using a monoclonal antibody raised against CCK2R. The coverage score was graded on a scale of 0 to 3 and plotted (error bars represent standard error of the mean). A t-test was used to determine if there were any significant differences between groups.

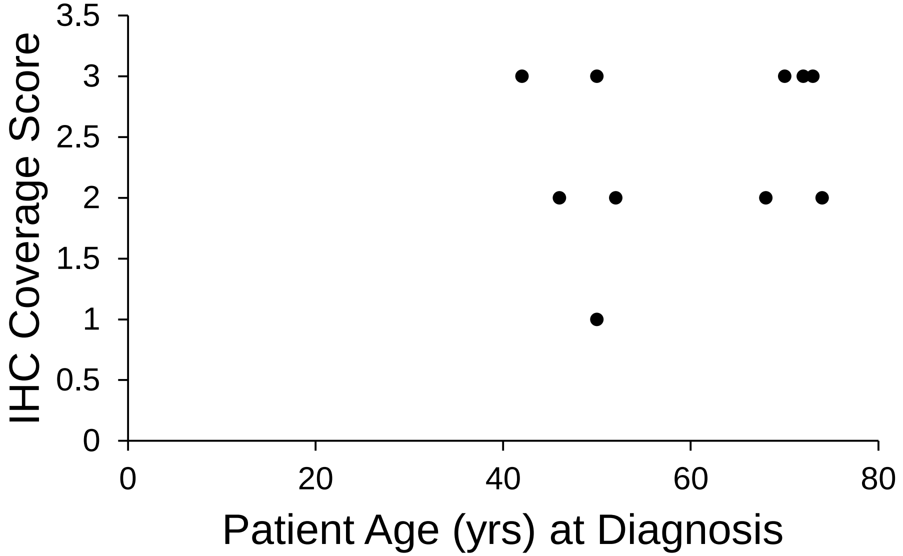

Spearman Analysis

ρ (rho) = 0.1277
t = 0.3641
p-value = 0.7242

	n	Average	St. Dev	St. Error
0	0	-	-	-
1	1	50.00	-	-
2	4	60.00	13.17	6.58
3	5	61.40	14.38	6.43

t-test

p-value = 0.885

SI Figure 49. Correlation analysis of CCK2R coverage score in metastases of GIST versus patient age at diagnosis. IHC was performed on metastases of GIST tissue sections using a monoclonal antibody raised against CCK2R. The coverage score was graded on a scale of 0 to 3 and plotted. A Spearman analysis was used to determine if there was a statistically significant correlation and a t-test was used to determine if there were any significant differences between groups.

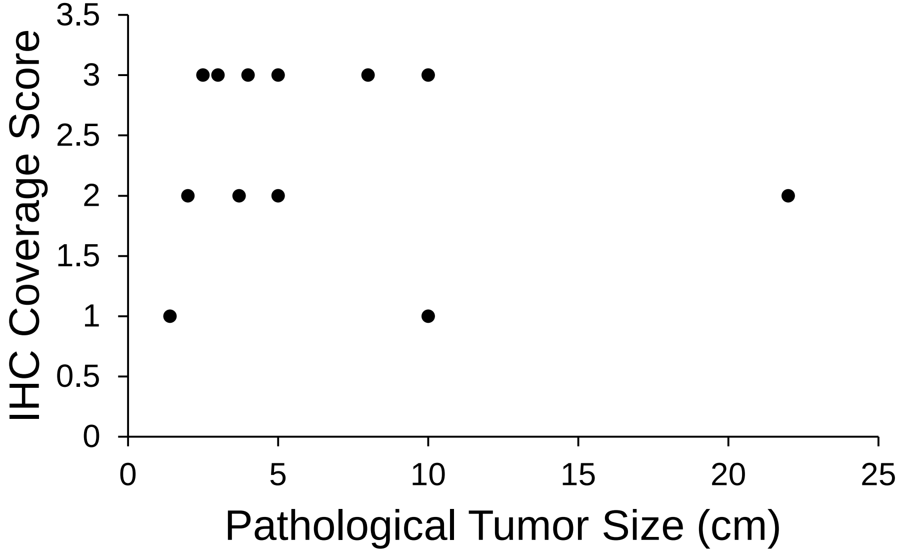

Spearman Analysis

ρ (rho) =	0.0728
t =	0.2308
p-value =	0.8217

	n	Average	St. Dev	St. Error
0	0	-	-	-
1	2	5.70	-	-
2	4	8.18	9.30	4.65
3	6	5.42	2.97	1.21

t-test

p-value = 0.508

SI Figure 50. Correlation analysis of CCK2R coverage score in metastases of GIST versus size of primary tumor (length of longest side). IHC was performed on metastases of GIST tissue sections using a monoclonal antibody raised against CCK2R. The coverage score was graded on a scale of 0 to 3 and plotted. A Spearman analysis was used to determine if there was a statistically significant correlation and a t-test was used to determine if there were any significant differences between groups.

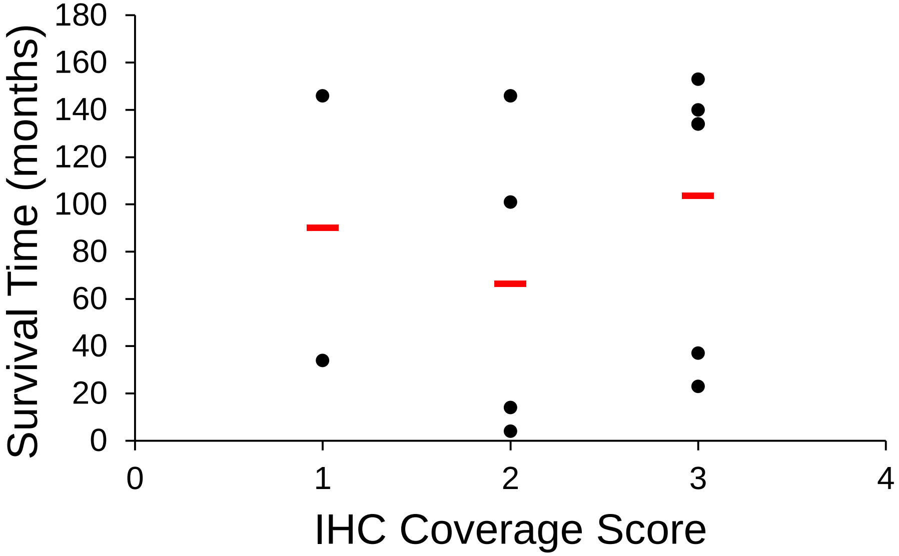

Spearman Analysis

Spearman Analysis	ρ (rho) =	0.1570
	t =	0.5029
	p-value =	0.6250

	n	Average	St. Dev	St. Error
0	0	-	-	-
1	2	90.00	79.20	56.00
2	4	66.25	68.73	34.37
3	6	103.50	57.52	23.48

t-test

p-value = 0.379

SI Figure 51. Correlation analysis of CCK2R coverage score in metastases of GIST versus survival time after diagnosis. IHC was performed on metastases of GIST tissue sections using a monoclonal antibody raised against CCK2R. The coverage score was graded on a scale of 0 to 3 and plotted (red bars represent population mean). A Spearman analysis was used to determine if there was a statistically significant correlation and a t-test was used to determine if there were any significant differences between groups.

GIST metastatic tumors
Total Staining Score Correlations

GIST Metastases

	n	Average	St. Dev	St. Error
Total Score	12	4.92	3.42	0.99

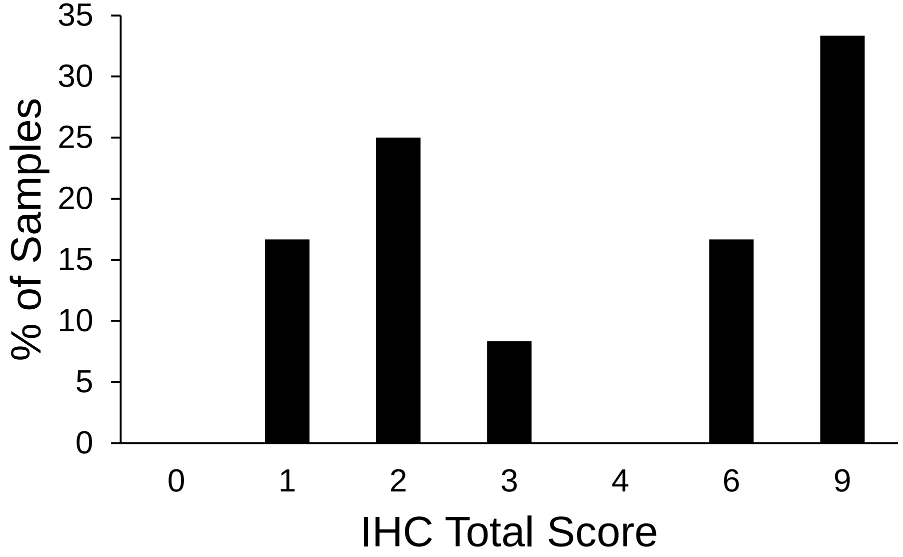

	Total Staining Score						
	0	1	2	3	4	6	9
n	0	2	3	1	0	2	4
%	0.00	16.67	25.00	8.33	0.00	16.67	33.33

SI Figure 52. CCK2R Total Staining Score for metastases from GIST primary tumors. IHC was performed on tissue sections using a monoclonal antibody raised against CCK2R. The staining intensity and coverage score was multiplied to obtain the total staining score.

GIST - metastases - Total Staining Score

	Sex	Age at Diagnosis	Primary Tumor Type	Primary Tumor Site	Stage	Grade	Tumor Size (TNM, T)	Tumor Size (longest dimension)	Lymph Node Involvement (TNM, N)	Metastatic (TNM, M)	Metastatic Site	Survival after Diagnosis	Survival after Stage IV Diagnosis
**Spearman Correlation**	N.A.	No<br>0.418	N.A.	N.A.	N.D.	N.D.	N.D.	No<br>0.483	N.D.	N.D.	N.A.	No<br>0.617	N.D.
**ANOVA/t-test**	No<br>0.43	N.D.	N.D.	N.D.	N.D.	N.D.	N.D.	N.D.	N.D.	N.D.	N.D.	N.D.	N.D.

SI Figure 53. Total staining score correlation summary of CCK2R in metastases of GIST. IHC was performed on metastases of GIST tissue sections using a monoclonal antibody raised against CCK2R. The total staining score was compared against available patient data. If appropriate, a spearman analysis was used to determine if any significant correlation exists while a 1-way ANOVA or t-test was used to determine if a significant difference exists between groups. Whether the test was statistically significant and the p-value is listed. N.A. – not applicable (this statistical test was not applicable to this data set). N.D. – not determined (this statistical test could not be performed, generally due to a lack of the number of samples within a group or all data was in a single group).

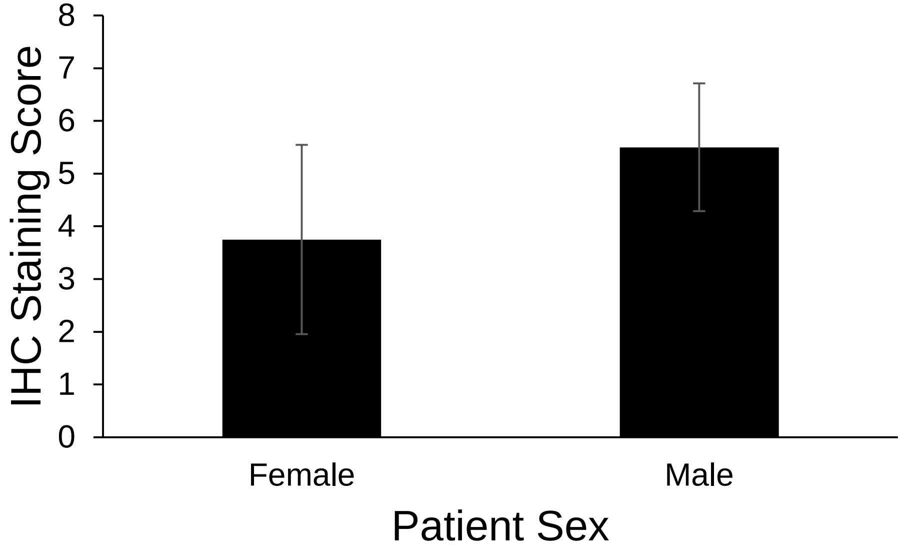

Spearman Analysis		
	ρ (rho) =	-
	t =	-
	p-value =	-

	n	Average	St. Dev	St. Error
Female	4	3.75	3.59	1.80
Male	8	5.50	3.42	1.21

t-test

p-value = 0.430

SI Figure 54. Correlation analysis of CCK2R total staining score in metastases of GIST versus patient sex. IHC was performed on metastases of GIST tissue sections using a monoclonal antibody raised against CCK2R. The total staining score was derived by multiplying the staining intensity with the coverage score (error bars represent standard error of the mean). A t-test was used to determine if there were any significant differences between groups.

Spearman Analysis

ρ (rho) = 0.2876
t = 0.8493
p-value = 0.4177

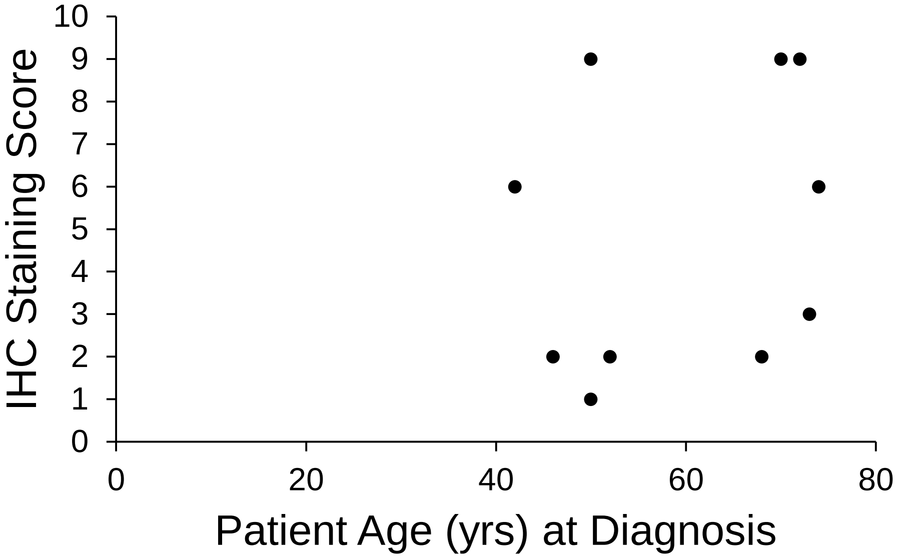

	n	Average	St. Dev	St. Error
0	0	-	-	-
1	1	50.00	-	-
2	3	55.33	11.37	6.57
3	1	73.00	-	-
4	0	-	-	-
6	2	58.00	-	-
9	3	64.00	12.17	7.02

t-test

p-value = -

SI Figure 55. Correlation analysis of CCK2R total staining score in metastases of GIST versus patient age at diagnosis. IHC was performed on metastases of GIST tissue sections using a monoclonal antibody raised against CCK2R. The total staining score was derived by multiplying the staining intensity with the coverage score. A Spearman analysis was used to determine if there was a statistically significant correlation.

Spearman Analysis

ρ (rho) = -0.2239
t = -0.7265
p-value = 0.4827

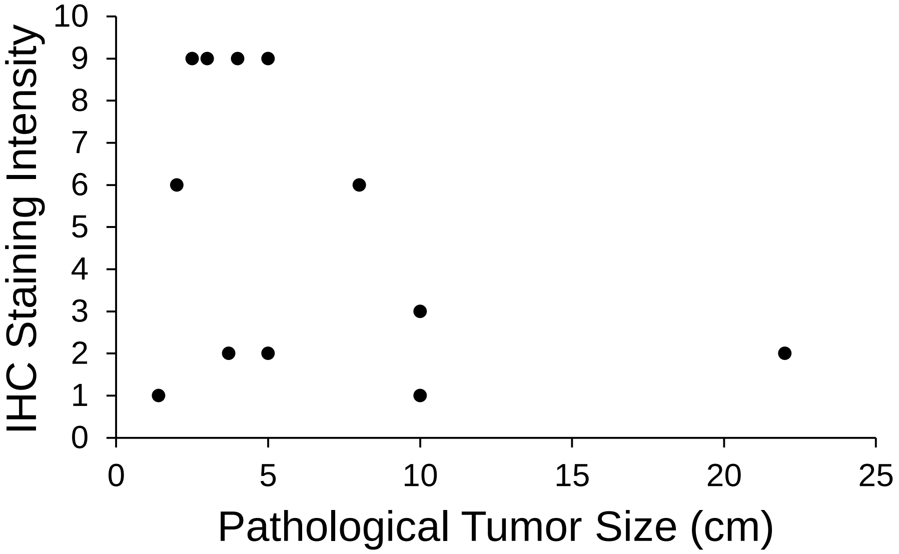

	n	Average	St. Dev	St. Error
0	0	-	-	-
1	2	5.70	-	-
2	3	10.23	10.21	5.90
3	1	10.00	-	-
4	0	-	-	-
6	2	5.00	-	-
9	4	3.63	1.11	0.55

t-test

p-value = -

SI Figure 56. Correlation analysis of CCK2R total staining score in metastases of GIST versus size of primary tumor (length of longest side). IHC was performed on metastases of GIST tissue sections using a monoclonal antibody raised against CCK2R. The total staining score was derived by multiplying the staining intensity with the coverage score. A Spearman analysis was used to determine if there was a statistically significant correlation.

Spearman Analysis

ρ (rho) =	0.1607	
t =	0.5149	
p-value =	0.6168	

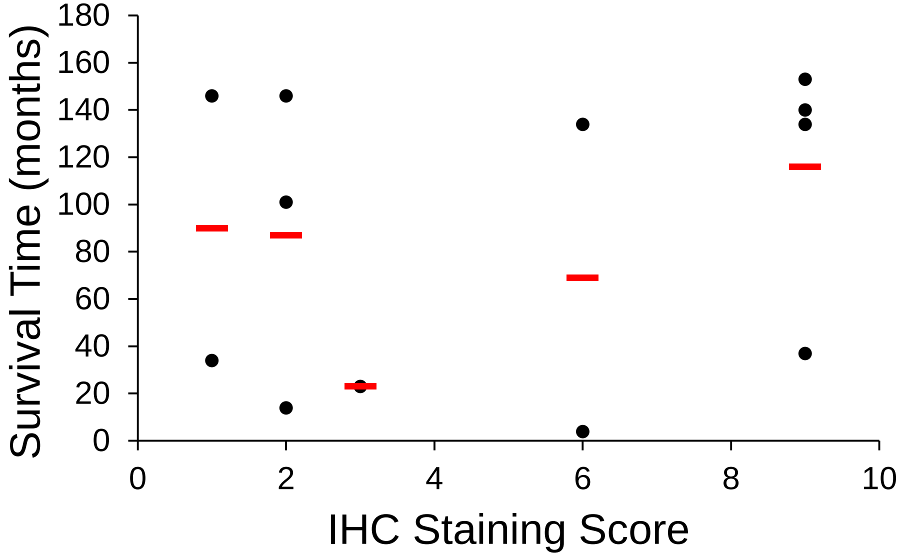

	n	Average	St. Dev	St. Error
0	0	-	-	-
1	2	90.00	-	-
2	3	87.00	67.10	38.74
3	1	23.00	-	-
4	0	-	-	-
6	2	69.00	-	-
9	4	116.00	53.26	26.63

t-test

p-value = -

SI Figure 57. Correlation analysis of CCK2R total staining score in metastases of GIST versus survival time after diagnosis. IHC was performed on metastases of GIST tissue sections using a monoclonal antibody raised against CCK2R. The total staining score was derived by multiplying the staining intensity with the coverage score (red bars represent population mean). A Spearman analysis was used to determine if there was a statistically significant correlation.

Liver Cancer

Liver Cancer Images

HCC, pathology total score = 0

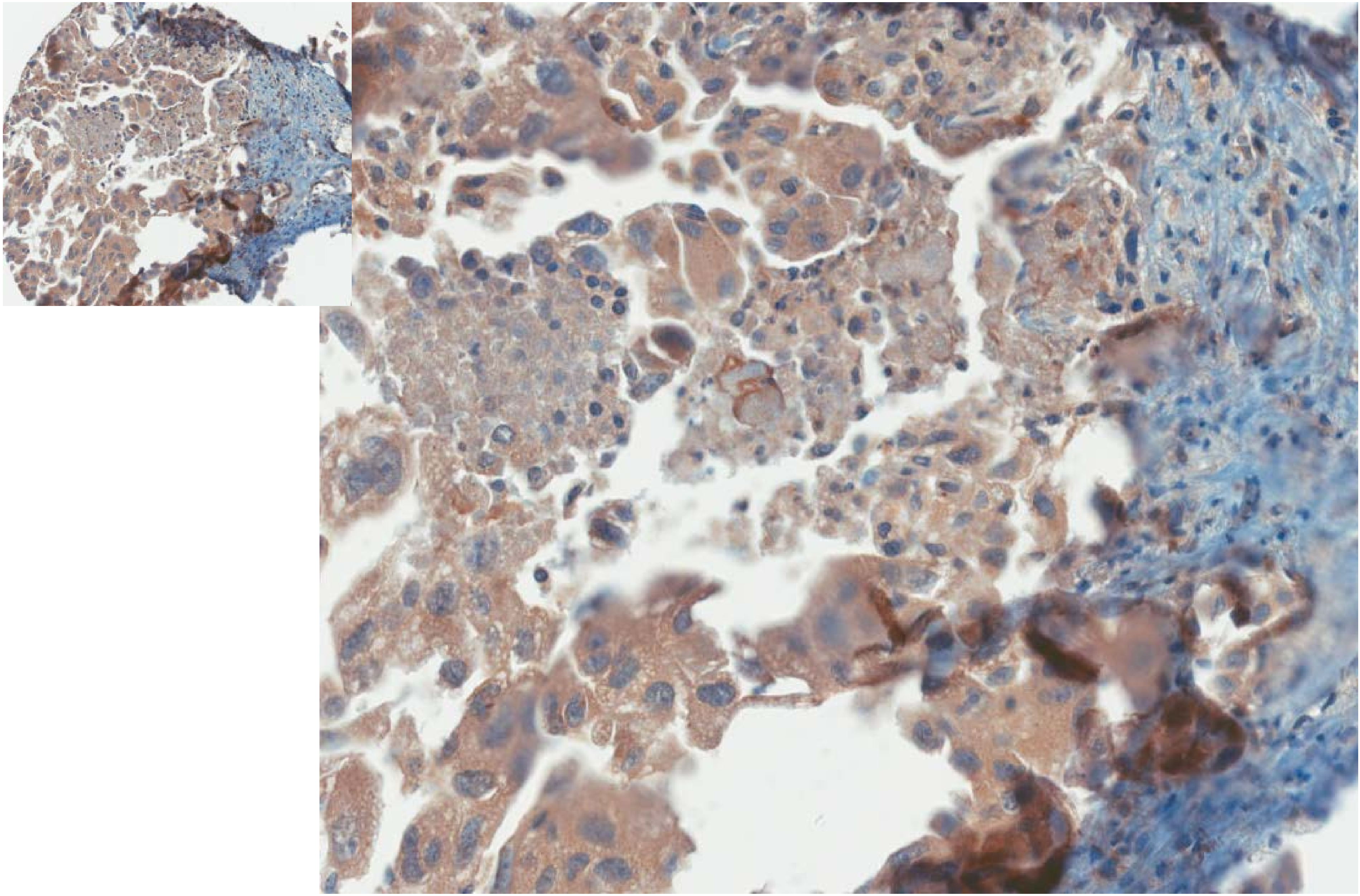

SI Figure 58. Example image of stained tissue from hepatocellular carcinoma. IHC was performed on tissue sections using a monoclonal antibody raised against CCK2R.

HCC, pathology total score = 1

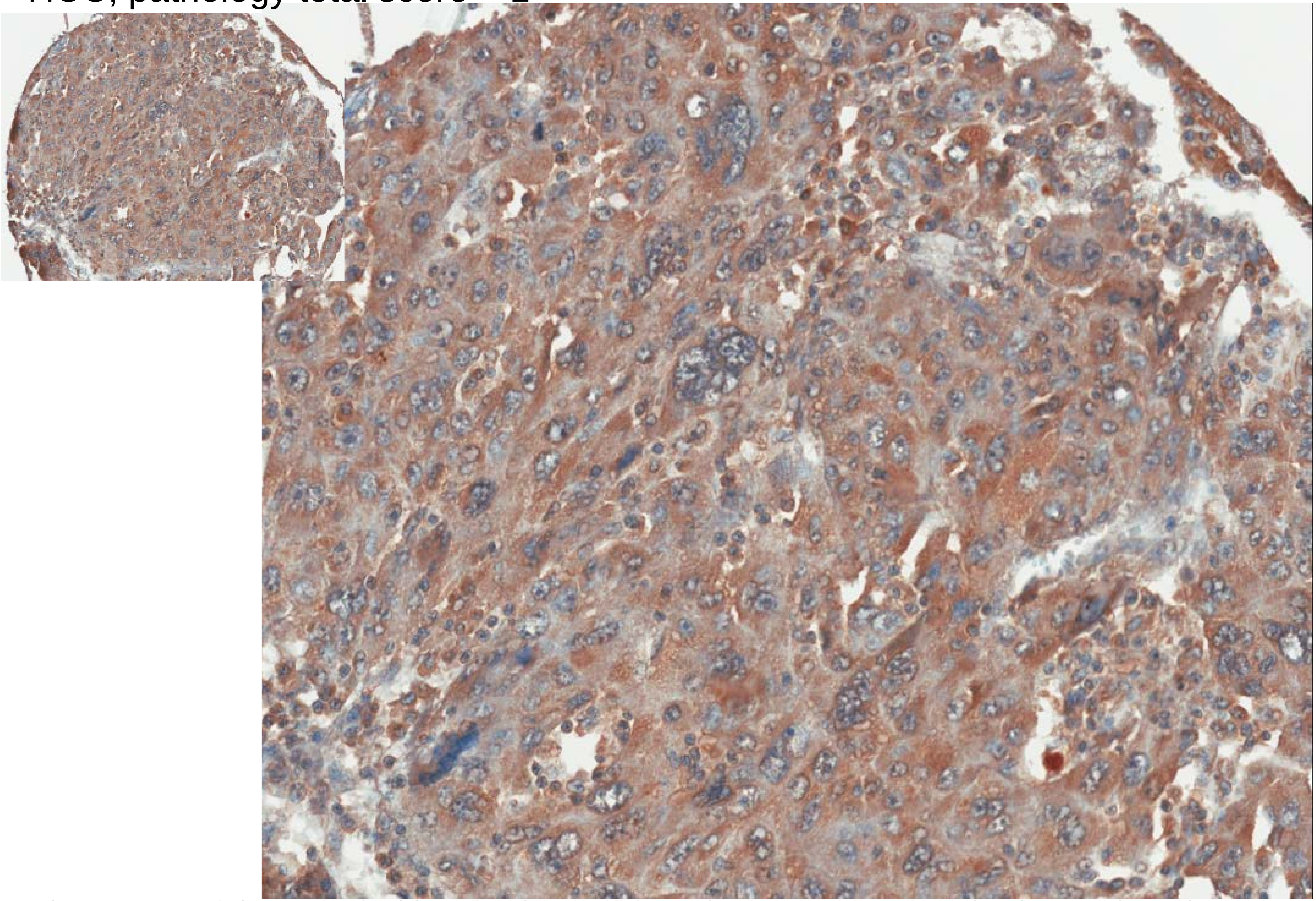

SI Figure 59. Example image of stained tissue from hepatocellular carcinoma. IHC was performed on tissue sections using a monoclonal antibody raised against CCK2R.

HCC, pathology total score = 2

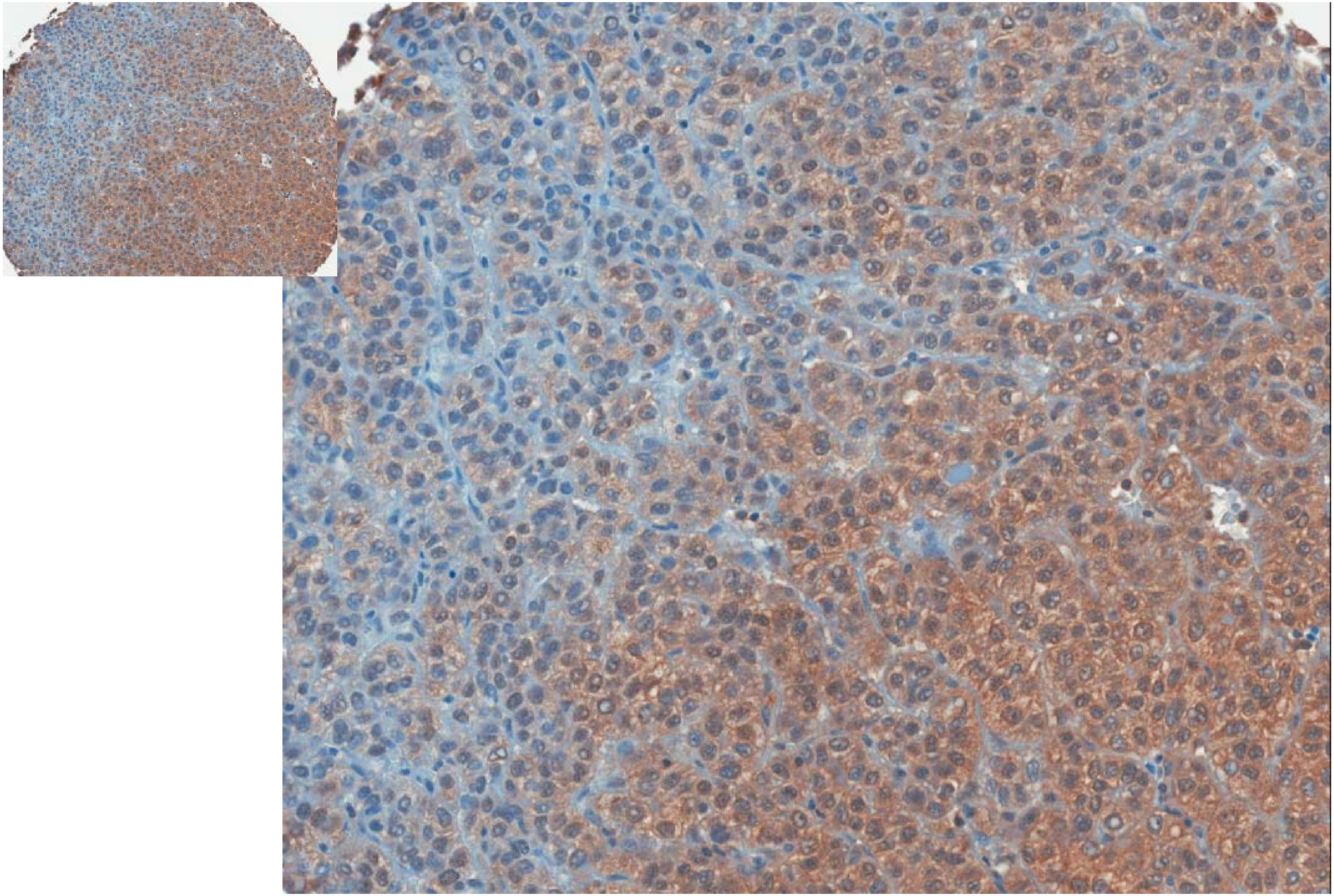

SI Figure 60. Example image of stained tissue from hepatocellular carcinoma. IHC was performed on tissue sections using a monoclonal antibody raised against CCK2R.

HCC, pathology total score = 3

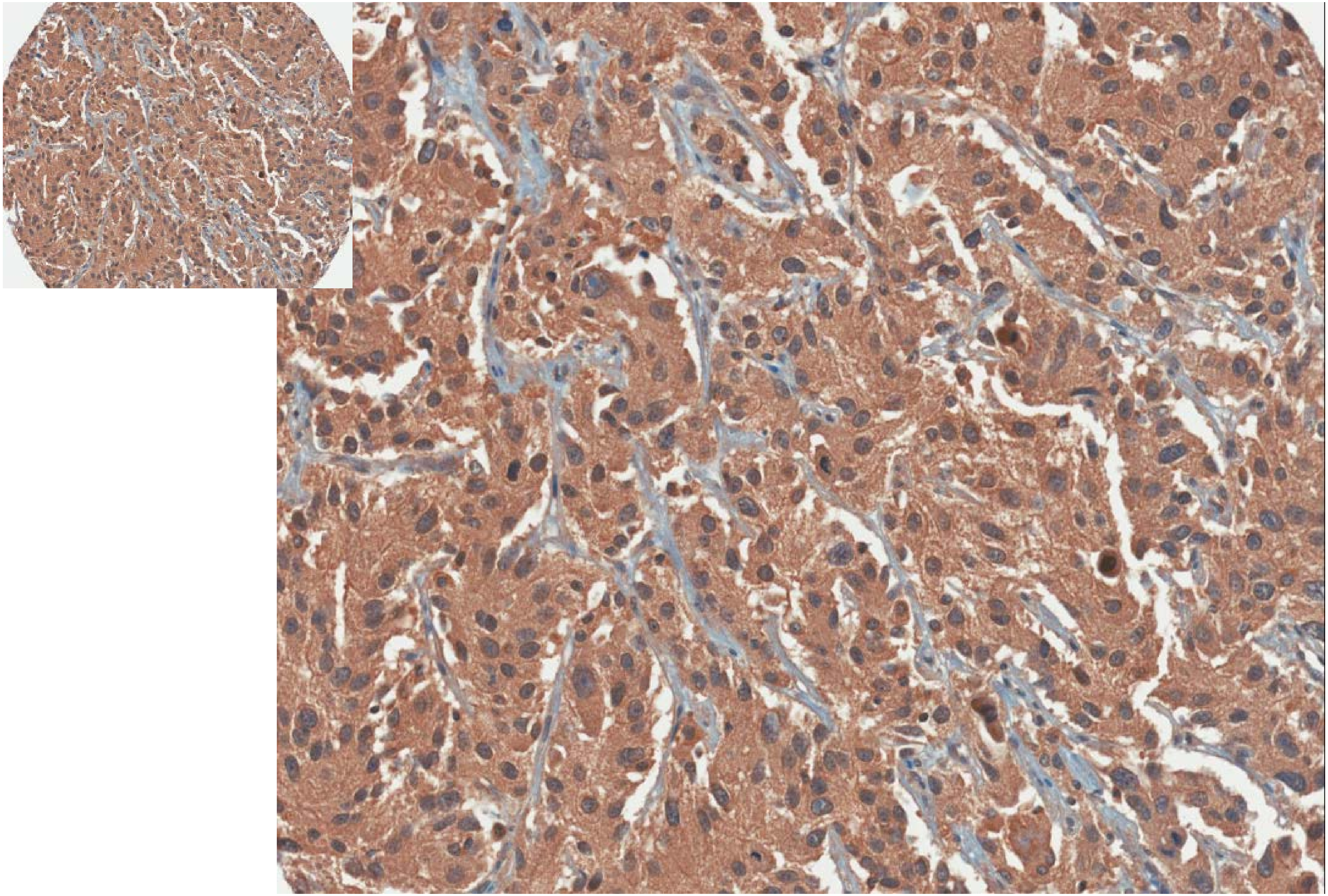

SI Figure 61. Example image of stained tissue from hepatocellular carcinoma. IHC was performed on tissue sections using a monoclonal antibody raised against CCK2R.

HCC, pathology total score = 4

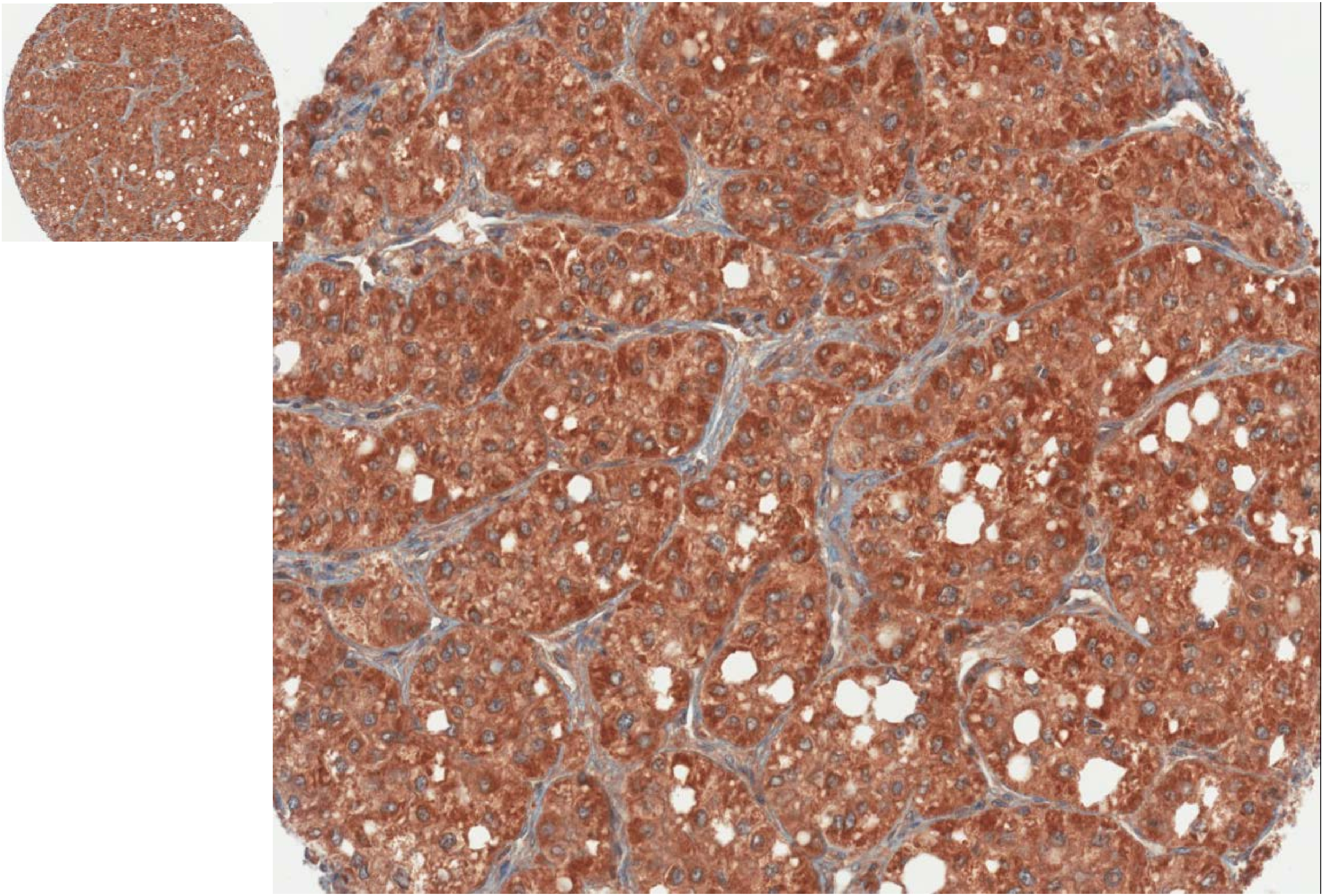

SI Figure 62. Example image of stained tissue from hepatocellular carcinoma. IHC was performed on tissue sections using a monoclonal antibody raised against CCK2R.

HCC, pathology total score = 6

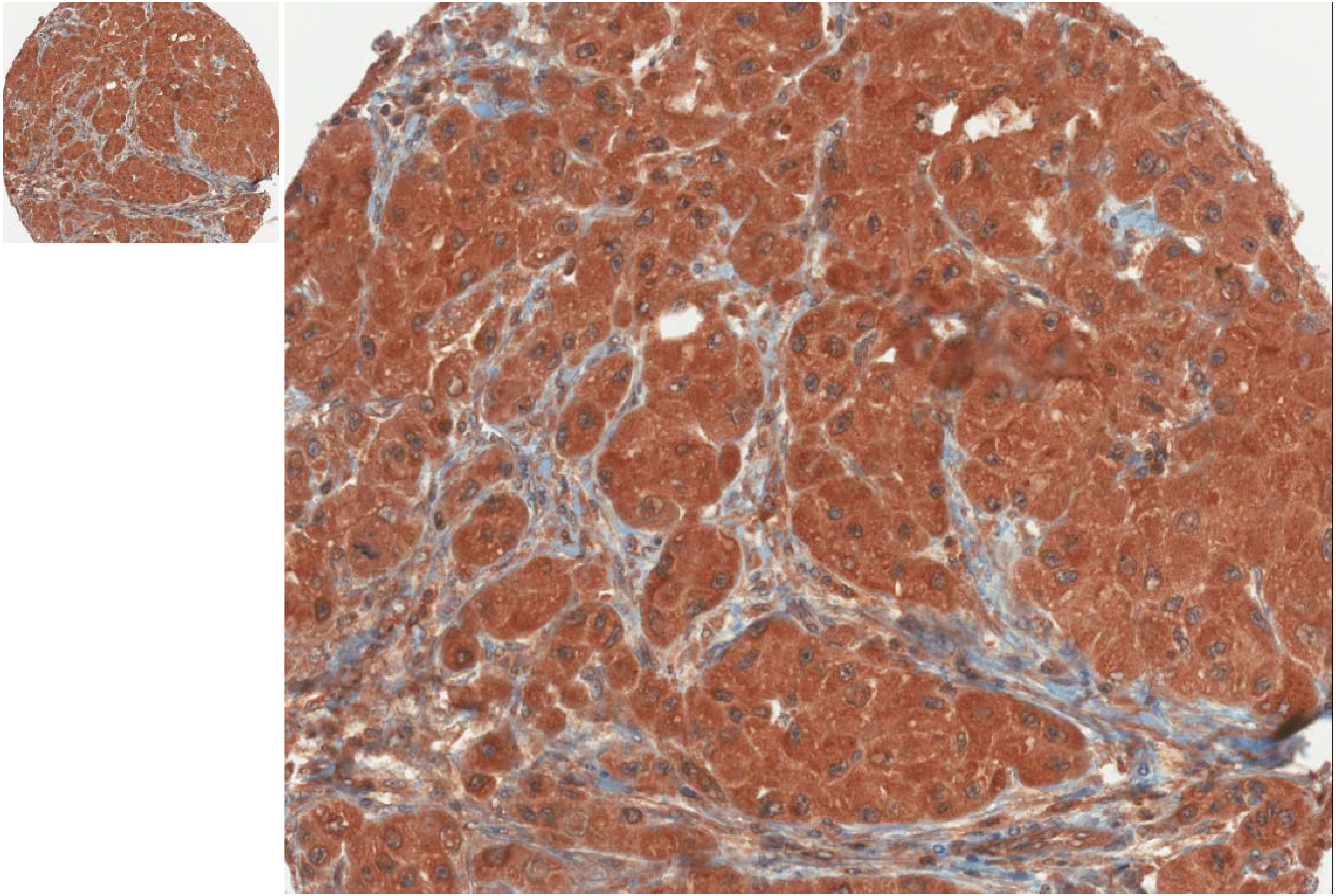

SI Figure 63. Example image of stained tissue from hepatocellular carcinoma. IHC was performed on tissue sections using a monoclonal antibody raised against CCK2R.

HCC, pathology total score = 9

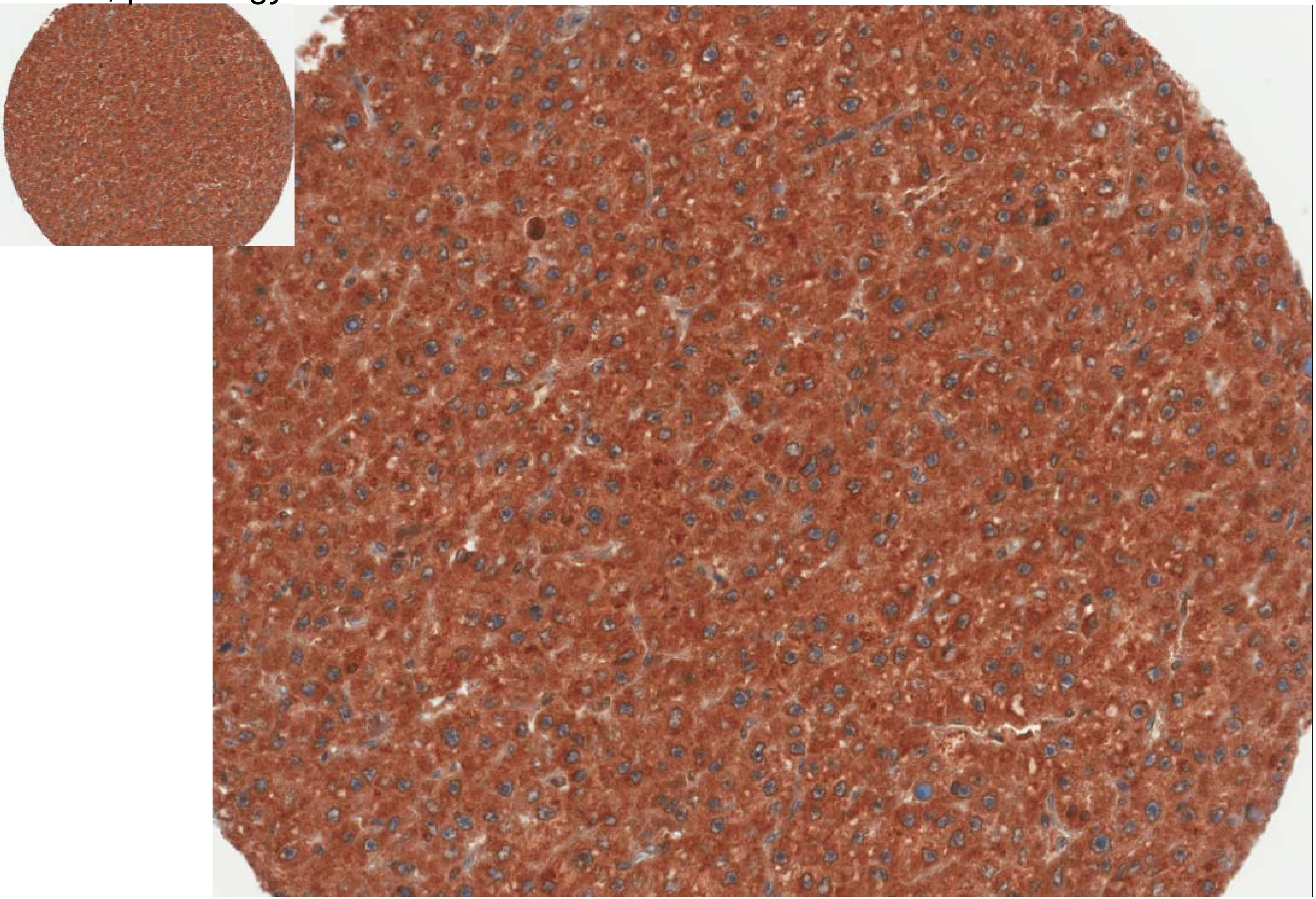

SI Figure 64. Example image of stained tissue from hepatocellular carcinoma. IHC was performed on tissue sections using a monoclonal antibody raised against CCK2R.

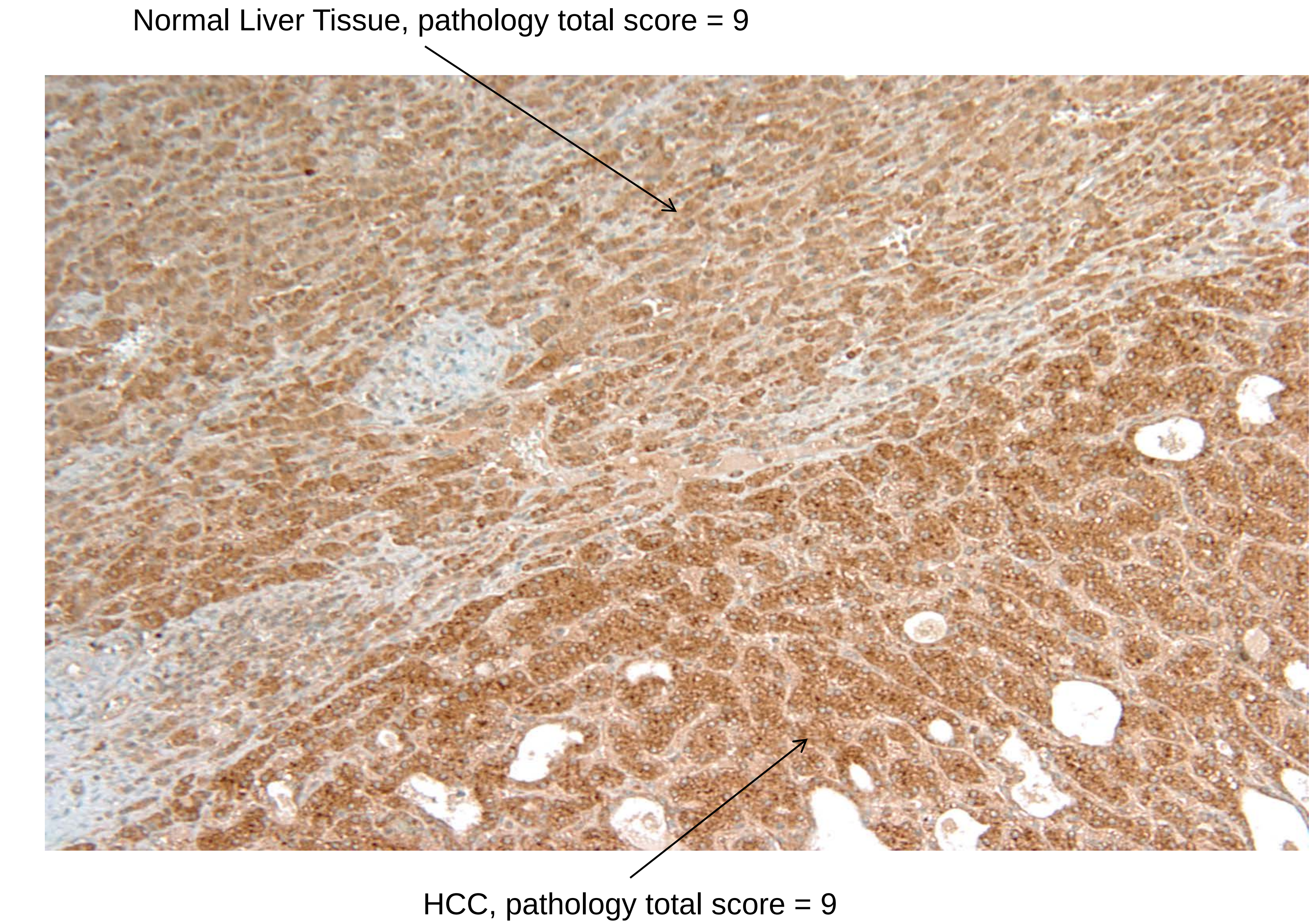

SI Figure 65. Example image of stained tissue from hepatocellular carcinoma including adjacent normal tissue. IHC was performed on tissue sections using a monoclonal antibody raised against CCK2R.

Liver Cancer
Overall Summary

Liver (Heptocellular Carcinoma) - Spearman Correlation

	Sex	Age at Diagnosis	Primary Tumor Type	Primary Tumor Site	Stage	Grade	Tumor Size (TNM, T)	Tumor Size (longest dimension)	Lymph Node Involvement (TNM, N)	Metastatic (TNM, M)	Metastatic Site	Survival after Diagnosis	Survival after Stage IV Diagnosis
**Staining Intensity**	N.A.	No 0.475	N.A.	N.A.	No 0.757	No 0.999	No 0.878	No 0.485	N.D.	N.D.	N.A.	No 0.209	N.D.
**Coverage Score**	N.A.	No 0.630	N.A.	N.A.	No 0.859	No 0.338	No 0.541	No 0.537	N.D.	N.D.	N.A.	No 0.869	N.D.
**Total Staining Score**	N.A.	No 0.564	N.A.	N.A.	No 0.623	No 0.606	No 0.343	No 0.720	N.D.	N.D.	N.A.	No 0.428	N.D.

Liver (Heptocellular Carcinoma) - ANOVA/t-test

	Sex	Age at Diagnosis	Primary Tumor Type	Primary Tumor Site	Stage	Grade	Tumor Size (TNM, T)	Tumor Size (longest dimension)	Lymph Node Involvement (TNM, N)	Metastatic (TNM, M)	Metastatic Site	Survival after Diagnosis	Survival after Stage IV Diagnosis
**Staining Intensity**	No 0.949	No 0.376	N.D.	N.D.	No 0.728	No 0.317	No 0.704	No 0.520	N.D.	N.D.	N.D.	No 0.537	N.D.
**Coverage Score**	No 0.845	No 0.745	N.D.	N.D.	No 0.249	No 0.098	No 0.557	No 0.788	N.D.	N.D.	N.D.	No 0.171	N.D.
**Total Staining Score**	No 0.826	No 0.660	N.D.	N.D.	No 0.991	No 0.265	No 0.114	No 0.892	N.D.	N.D.	N.D.	No 0.989	N.D.

SI Figure 66. Correlation summary of CCK2R in Liver cancer (hepatocellular carcinoma). IHC was performed on liver tumor tissue sections using a monoclonal antibody raised against CCK2R. The staining intensity, coverage score and total staining score were compared against available patient data. If appropriate, a spearman analysis was used to determine if any significant correlation exists while a 1-way ANOVA or t-test was used to determine if a significant difference exists between groups. N.A. – not applicable (this statistical test was not applicable to this data set). Whether the test was statistically significant and the p-value is listed. N.D. – not determined (this statistical test could not be performed, generally due to a lack of the number of samples within a group or all data was in a single group).

Liver Cancer
Staining Intensity

Liver Cancer

	n	Average	St. Dev	St. Error
Staining Intensity	43	1.93	0.86	0.13

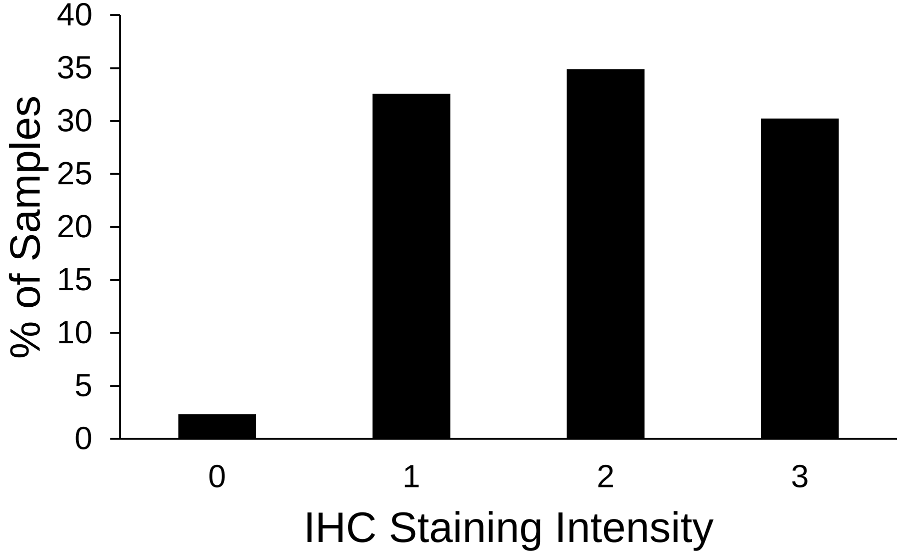

	Staining Intensity			
	0	1	2	3
n	1	14	15	13
%	2.33	32.56	34.88	30.23

Normal Liver

	n	Average	St. Dev	St. Error
Staining Intensity	22	1.95	1.05	0.22

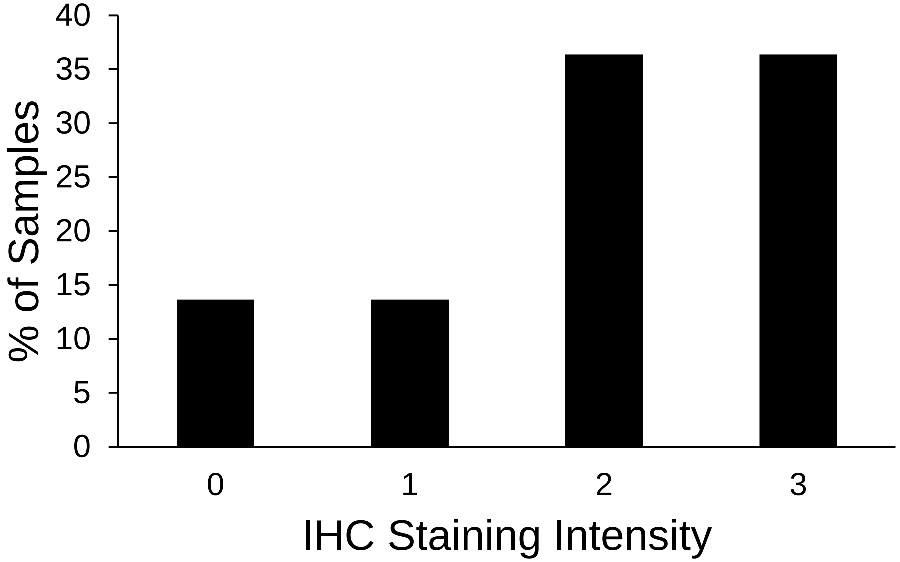

	Staining Intensity			
	0	1	2	3
n	3	3	8	8
%	13.64	13.64	36.36	36.36

SI Figure 67. CCK2R Staining Intensity for cancer and normal tissue from the liver. IHC was performed on tissue sections using a monoclonal antibody raised against CCK2R. The intensity of staining was graded on a scale of 0 to 3 and plotted.

Liver (hepatocellular carcinoma) - Staining Intensity

	Sex	Age at Diagnosis	Primary Tumor Type	Primary Tumor Site	Stage	Grade	Tumor Size (TNM, T)	Tumor Size (longest dimension)	Lymph Node Involvement (TNM, N)	Metastatic (TNM, M)	Metastatic Site	Survival after Diagnosis	Survival after Stage IV Diagnosis
**Spearman Correlation**	N.A.	No 0.475	N.A.	N.A.	No 0.757	No 0.999	No 0.878	No 0.485	N.D.	N.D.	N.A.	No 0.209	N.D.
**ANOVA/t-test**	No 0.949	No 0.376	N.D.	N.D.	No 0.728	No 0.317	No 0.704	No 0.520	N.D.	N.D.	N.D.	No 0.537	N.D.

SI Figure 68. Staining intensity correlation summary of CCK2R in liver cancer (hepatocellular carcinoma). IHC was performed on liver tumor tissue sections using a monoclonal antibody raised against CCK2R. The staining intensity was compared against available patient data. If appropriate, a spearman analysis was used to determine if any significant correlation exists while a 1-way ANOVA or t-test was used to determine if a significant difference exists between groups. N.A. – not applicable (this statistical test was not applicable to this data set). Whether the test was statistically significant and the p-value is listed. N.D. – not determined (this statistical test could not be performed, generally due to a lack of the number of samples within a group or all data was in a single group).

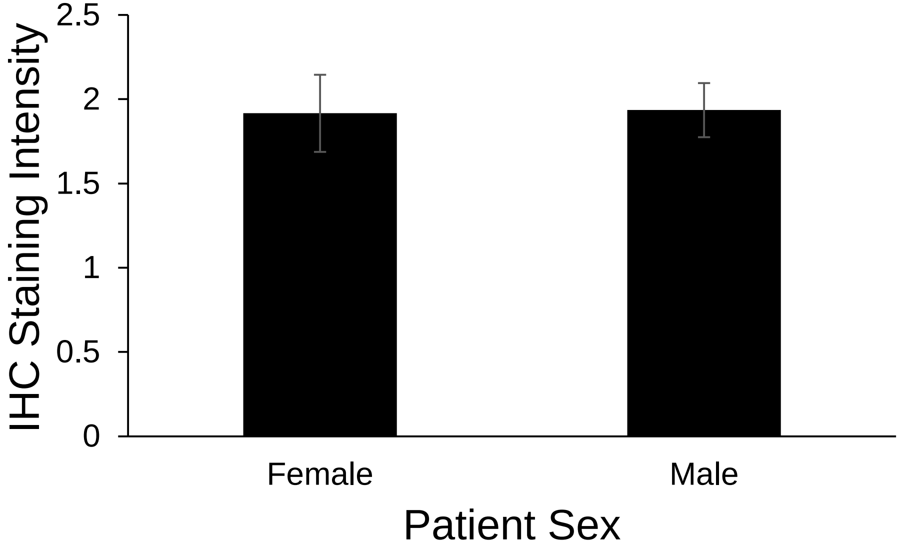

Spearman Analysis

ρ (rho) =	-
t =	-
p-value =	-

	n	Average	St. Dev	St. Error
Female	12	1.92	0.79	0.23
Male	31	1.94	0.89	0.16

t-test

p-value = 0.9494

SI Figure 69. Correlation analysis of CCK2R staining intensity in liver cancer (hepatocellular carcinoma) versus patient sex. IHC was performed on liver cancer (hepatocellular carcinoma) tissue sections using a monoclonal antibody raised against CCK2R. The staining intensity was graded on a scale of 0 to 3 and plotted (error bars represent standard error of the mean). A t-test was used to determine if there were any significant differences between groups.

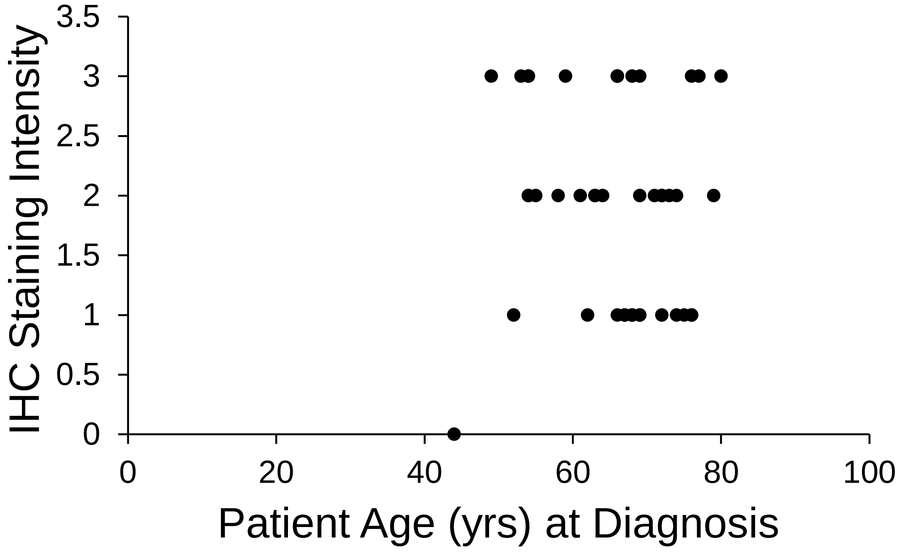

Spearman Analysis

ρ (rho) =	-0.1118
t =	-0.7203
p-value =	0.4753

	n	Average	St. Dev	St. Error
0	1	44.00	-	-
1	14	68.64	6.34	1.70
2	15	65.47	8.03	2.07
3	13	64.38	9.96	2.76

1-Way Anova

	SS	df	MS	F	p
Between	134	2	67.2	1.002	0.376
Within	2,616	39	67.1		
Total	2,750	41			

SI Figure 70. Correlation analysis of CCK2R staining intensity in liver cancer (hepatocellular carcinoma) versus patient age at diagnosis. IHC was performed on liver cancer (hepatocellular carcinoma) tissue sections using a monoclonal antibody raised against CCK2R. The staining intensity was graded on a scale of 0 to 3 and plotted. A Spearman analysis was used to determine if there was a statistically significant correlation and a 1-way ANOVA was used to determine if there were any significant differences between groups.

Spearman Analysis		
	ρ (rho) =	-0.0504
	t =	-0.3111
	p-value =	0.7573

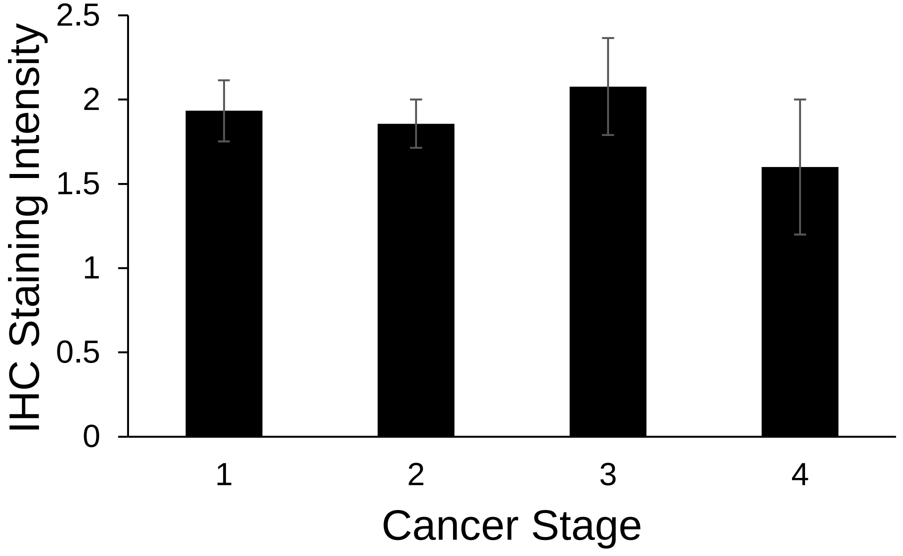

	n	Average	St. Dev	St. Error
1	15	1.93	0.70	0.18
2	7	1.86	0.38	0.14
3	13	2.08	1.04	0.29
4	5	1.60	0.89	0.40

1-Way Anova

	SS	df	MS	F	p
Between	0.87	3	0.29	0.437	0.728
Within	23.87	36	0.66		
Total	24.74	39			

SI Figure 71. Correlation analysis of CCK2R staining intensity in liver cancer (hepatocellular carcinoma) versus cancer stage. IHC was performed on liver cancer (hepatocellular carcinoma) tissue sections using a monoclonal antibody raised against CCK2R. The staining intensity was graded on a scale of 0 to 3 and plotted (error bars represent standard error of the mean). A Spearman analysis was used to determine if there was a statistically significant correlation and a 1-way ANOVA was used to determine if there were any significant differences between groups.

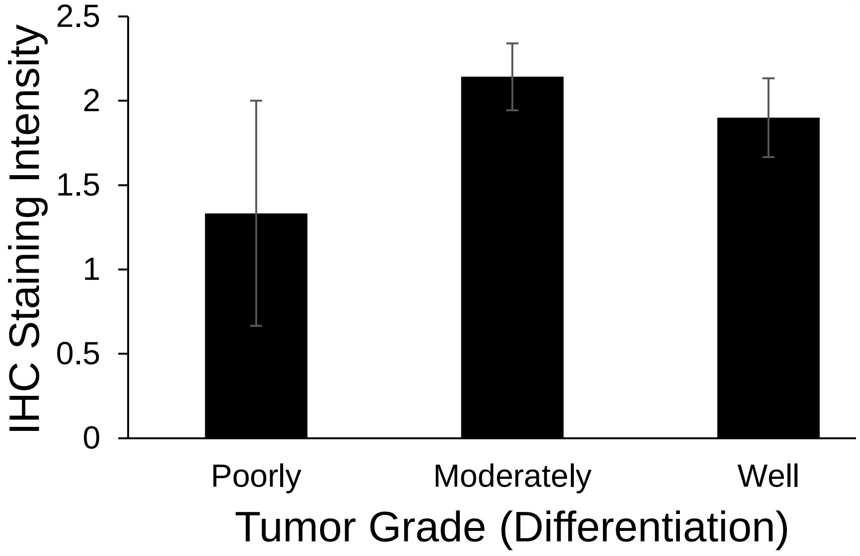

Spearman Analysis

ρ (rho) =	0.0003
t =	0.0016
p-value =	0.9987

	n	Average	St. Dev	St. Error
Poorly Differentiated	3	1.33	1.15	0.67
Moderately Differentiated	21	2.14	0.91	0.20
Well Differentiated	10	1.90	0.74	0.23

1-Way Anova

	SS	df	MS	F	p
Between	1.9	2	0.93	1.193	0.317
Within	24.1	31	0.78		
Total	26.0	33			

SI Figure 72. Correlation analysis of CCK2R staining intensity in liver cancer (hepatocellular carcinoma) versus primary tumor grade. IHC was performed on liver cancer (hepatocellular carcinoma) tissue sections using a monoclonal antibody raised against CCK2R. The staining intensity was graded on a scale of 0 to 3 and plotted (error bars represent standard error of the mean). A Spearman analysis was used to determine if there was a statistically significant correlation and a 1-way ANOVA was used to determine if there were any significant differences between groups.

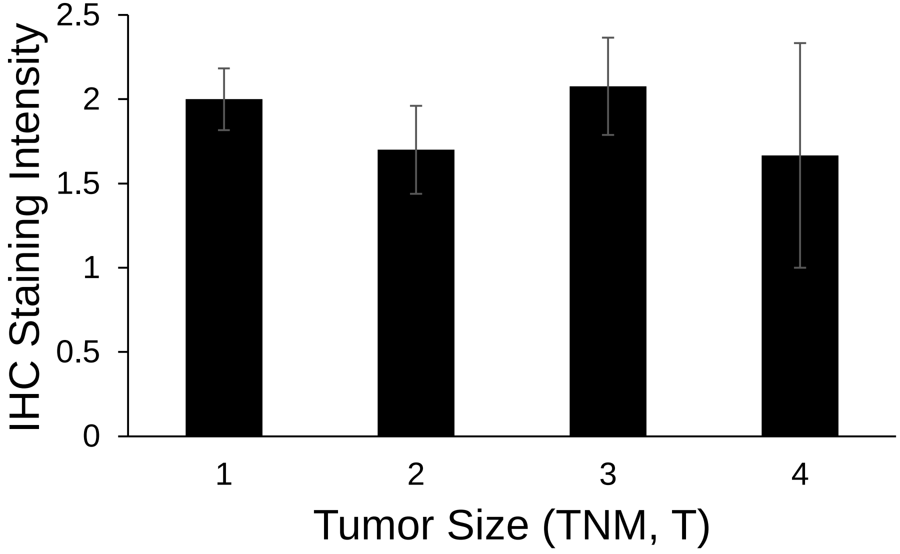

Spearman Analysis

ρ (rho) =	-0.0245
t =	-0.1551
p-value =	0.8775

	n	Average	St. Dev	St. Error
1	16	2.00	0.73	0.18
2	10	1.70	0.82	0.26
3	13	2.08	1.04	0.29
4	3	1.67	1.15	0.67

1-Way Anova

	SS	df	MS	F	p
Between	1.10	3	0.368	0.471	0.704
Within	29.67	38	0.781		
Total	30.77	41			

SI Figure 73. Correlation analysis of CCK2R staining intensity in liver cancer (hepatocellular carcinoma) versus primary tumor size. IHC was performed on liver cancer (hepatocellular carcinoma) tissue sections using a monoclonal antibody raised against CCK2R. The staining intensity was graded on a scale of 0 to 3 and plotted (error bars represent standard error of the mean). A Spearman analysis was used to determine if there was a statistically significant correlation and a 1-way ANOVA was used to determine if there were any significant differences between groups.

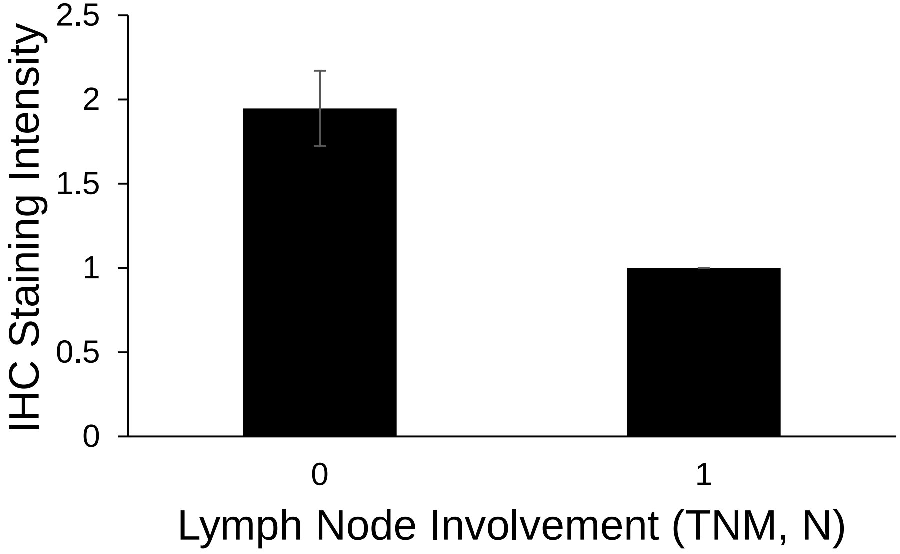

Spearman Analysis ρ (rho) = -

t = -

p-value = -

	n	Average	St. Dev	St. Error
0	38	1.95	0.87	0.22
1	1	1.00	-	-

t-test

p-value = -

SI Figure 74. Correlation analysis of CCK2R staining intensity in liver cancer (hepatocellular carcinoma) versus lymph node involvement. IHC was performed on liver cancer (hepatocellular carcinoma) tissue sections using a monoclonal antibody raised against CCK2R. The staining intensity was graded on a scale of 0 to 3 and plotted (error bars represent standard error of the mean). No statistical tests could be performed.

Spearman Analysis		
	ρ (rho) =	-0.1221
	t =	-0.7067
	p-value =	0.4846

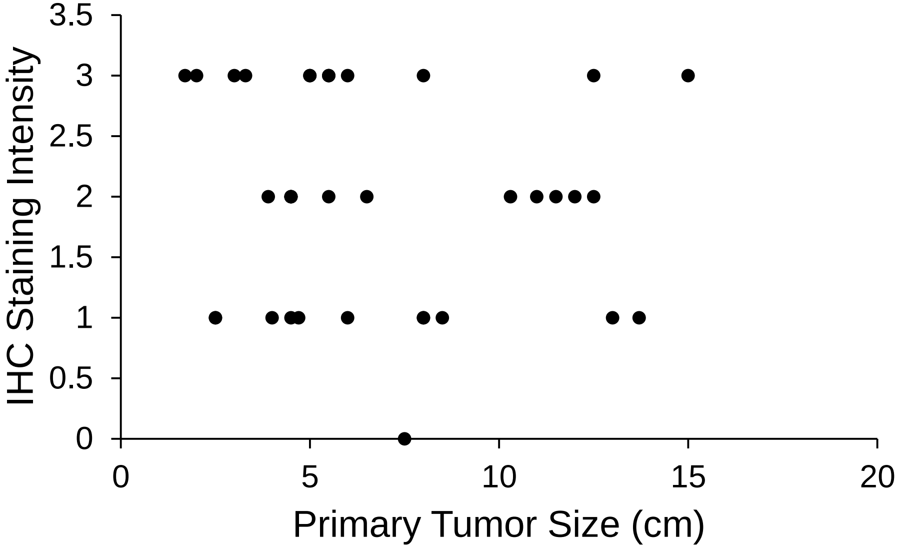

	n	Average	St. Dev	St. Error
0	1	7.50	-	-
1	11	6.85	3.83	1.16
2	11	7.88	3.53	1.06
3	12	6.04	4.05	1.17

1-Way Anova

	SS	df	MS	F	p
Between	19.5	2	9.73	0.668	0.520
Within	451.7	39	14.57		
Total	471.2	41			

SI Figure 75. Correlation analysis of CCK2R staining intensity in liver cancer (hepatocellular carcinoma) versus size of primary tumor (length of longest side). IHC was performed on liver cancer (hepatocellular carcinoma) tissue sections using a monoclonal antibody raised against CCK2R. The staining intensity was graded on a scale of 0 to 3 and plotted. A Spearman analysis was used to determine if there was a statistically significant correlation and a 1-way ANOVA was used to determine if there were any significant differences between groups.

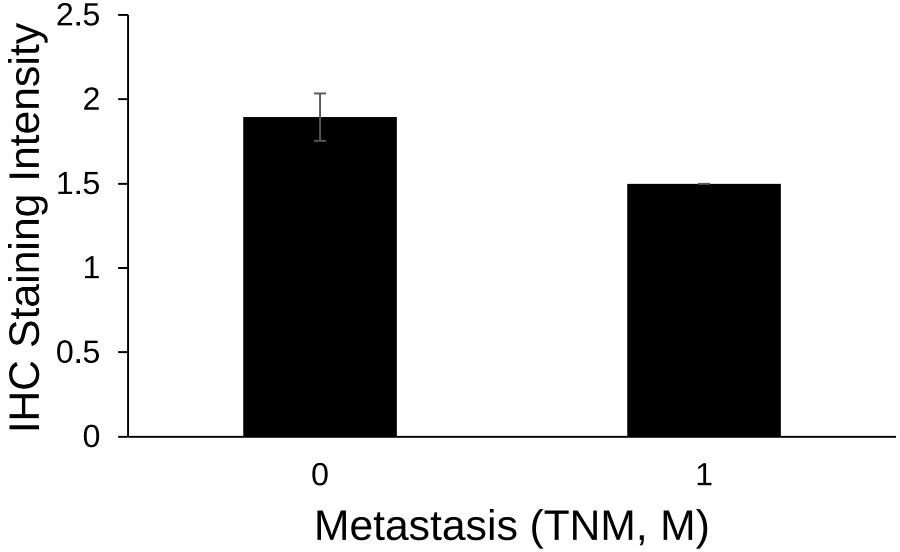

Spearman Analysis

ρ (rho) =	-
t =	-
p-value =	-

	n	Average	St. Dev	St. Error
0	38	1.89	0.86	0.14
1	2	1.50	-	-

t-test

p-value = -

SI Figure 76. Correlation analysis of CCK2R staining intensity in liver cancer (hepatocellular carcinoma) versus metastases. IHC was performed on liver cancer (hepatocellular carcinoma) tissue sections using a monoclonal antibody raised against CCK2R. The staining intensity was graded on a scale of 0 to 3 and plotted (error bars represent standard error of the mean). No statistical tests could be performed.

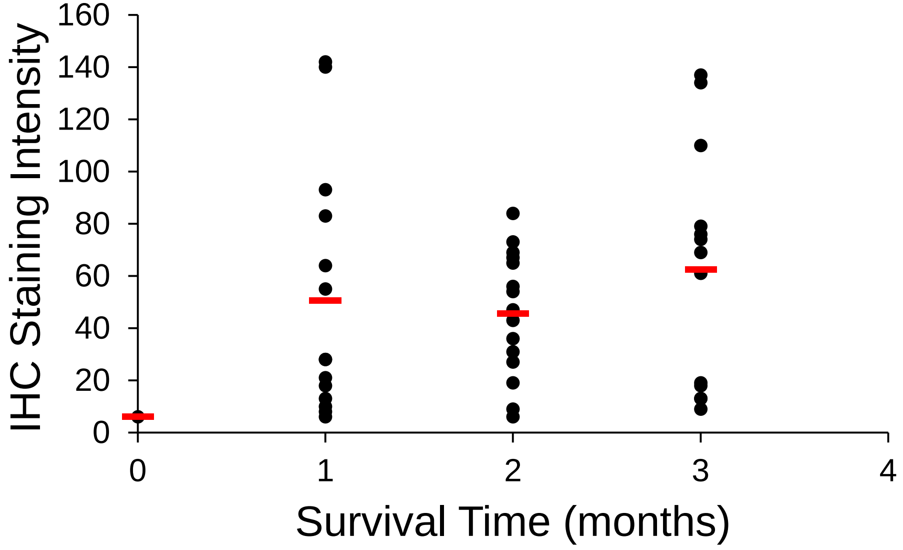

Spearman Analysis		
	ρ (rho) =	0.1954
	t =	1.2759
	p-value =	0.2090

	n	Average	St. Dev	St. Error
0	1	6.00	-	-
1	14	50.64	47.41	12.67
2	15	45.73	24.00	6.20
3	13	62.46	45.81	12.71

1-Way Anova

	SS	df	MS	F	p
Between	2,025	2	1,013	0.632	0.537
Within	62,467	39	1,602		
Total	64,492	41			

SI Figure 77. Correlation analysis of CCK2R staining intensity in liver cancer (hepatocellular carcinoma) versus survival time after diagnosis. IHC was performed on liver cancer (hepatocellular carcinoma) tissue sections using a monoclonal antibody raised against CCK2R. The staining intensity was graded on a scale of 0 to 3 and plotted (red bars represent population mean). A Spearman analysis was used to determine if there was a statistically significant correlation and a 1-way ANOVA was used to determine if there were any significant differences between groups.

Liver Cancer
Coverage Score Correlations

Liver Cancer

	n	Average	St. Dev	St. Error
Coverage Score	43	2.70	0.64	0.10

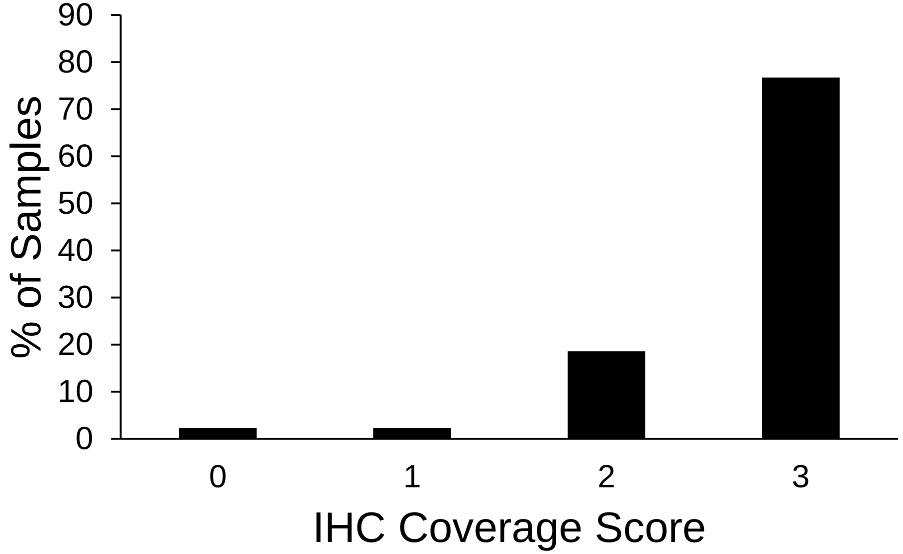

	Coverage Score			
	0	1	2	3
n	1	1	8	33
%	2.33	2.33	18.60	76.74

Normal Liver

	n	Average	St. Dev	St. Error
Coverage Score	22	2.41	1.05	0.22

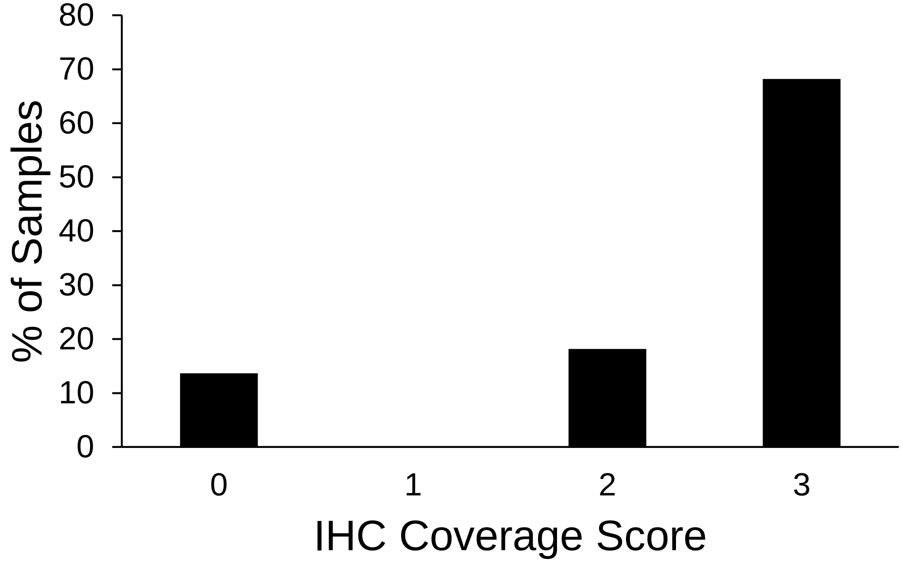

	Coverage Score			
	0	1	2	3
n	3	0	4	15
%	13.64	0.00	18.18	68.18

SI Figure 78. CCK2R Coverage Score for cancer and normal tissue from the liver. IHC was performed on tissue sections using a monoclonal antibody raised against CCK2R. The area stained (coverage) was graded on a scale of 0 to 3 and plotted.

Liver (hepatocellular carcinoma) - Coverage Score

	Sex	Age at Diagnosis	Primary Tumor Type	Primary Tumor Site	Stage	Grade	Tumor Size (TNM, T)	Tumor Size (longest dimension)	Lymph Node Involvement (TNM, N)	Metastatic (TNM, M)	Metastatic Site	Survival after Diagnosis	Survival after Stage IV Diagnosis
**Spearman Correlation**	N.A.	No 0.630	N.A.	N.A.	No 0.859	No 0.338	No 0.541	No 0.537	N.D.	N.D.	N.A.	No 0.869	N.D.
**ANOVA/t-test**	No 0.845	No 0.745	N.D.	N.D.	No 0.249	No 0.098	No 0.557	No 0.788	N.D.	N.D.	N.D.	No 0.171	N.D.

SI Figure 79. Coverage score correlation summary of CCK2R in liver cancer (hepatocellular carcinoma). IHC was performed on liver tumor tissue sections using a monoclonal antibody raised against CCK2R. The coverage score was compared against available patient data. If appropriate, a spearman analysis was used to determine if any significant correlation exists while a 1-way ANOVA or t-test was used to determine if a significant difference exists between groups. N.A. – not applicable (this statistical test was not applicable to this data set). Whether the test was statistically significant and the p-value is listed. N.D. – not determined (this statistical test could not be performed, generally due to a lack of the number of samples within a group or all data was in a single group).

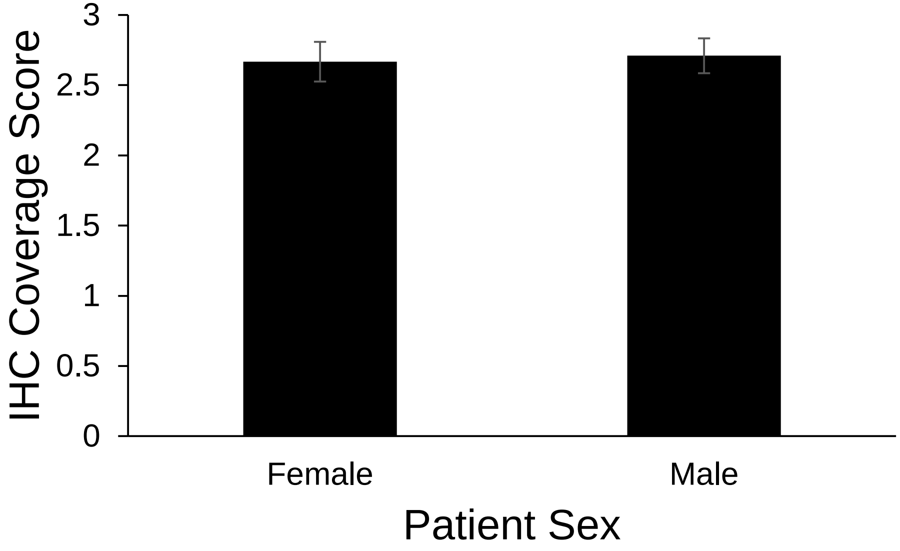

Spearman Analysis

ρ (rho) =	-
t =	-
p-value =	-

	n	Average	St. Dev	St. Error
Female	12	2.67	0.49	0.14
Male	31	2.71	0.69	0.12

t-test

p-value = 0.8454

SI Figure 80. Correlation analysis of CCK2R coverage score in liver cancer (hepatocellular carcinoma) versus patient sex. IHC was performed on liver cancer (hepatocellular carcinoma) tissue sections using a monoclonal antibody raised against CCK2R. The coverage score was graded on a scale of 0 to 3 and plotted (error bars represent standard error of the mean). A t-test was used to determine if there were any significant differences between groups.

Spearman Analysis

ρ (rho) = 0.0755
t = 0.4850
p-value = 0.6302

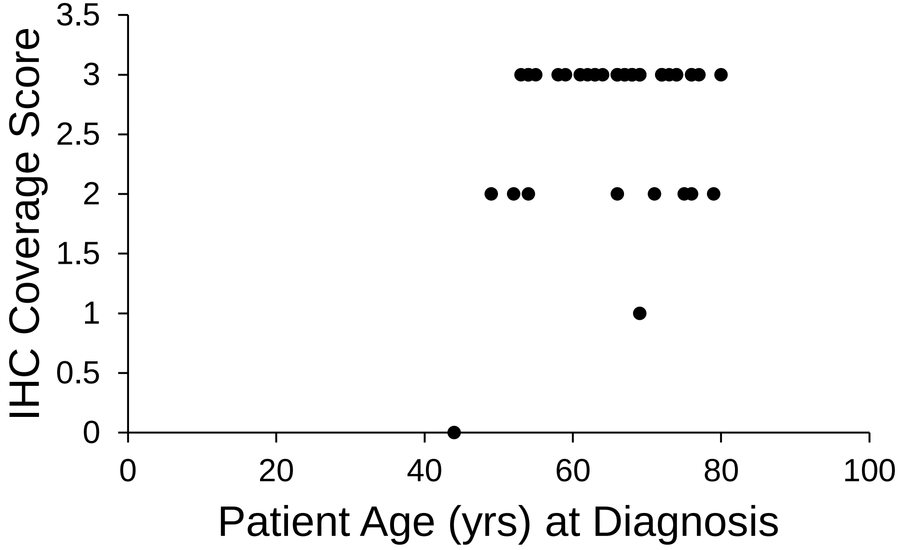

	n	Average	St. Dev	St. Error
0	1	44.00	-	-
1	1	69.00	-	-
2	8	65.25	11.95	4.22
3	33	66.33	7.37	1.28

t-test

p-value = 0.7446

SI Figure 81. Correlation analysis of CCK2R coverage score in liver cancer (hepatocellular carcinoma) versus patient age at diagnosis. IHC was performed on liver cancer (hepatocellular carcinoma) tissue sections using a monoclonal antibody raised against CCK2R. The coverage score was graded on a scale of 0 to 3 and plotted. A Spearman analysis was used to determine if there was a statistically significant correlation and a t-test was used to determine if there were any significant differences between groups.

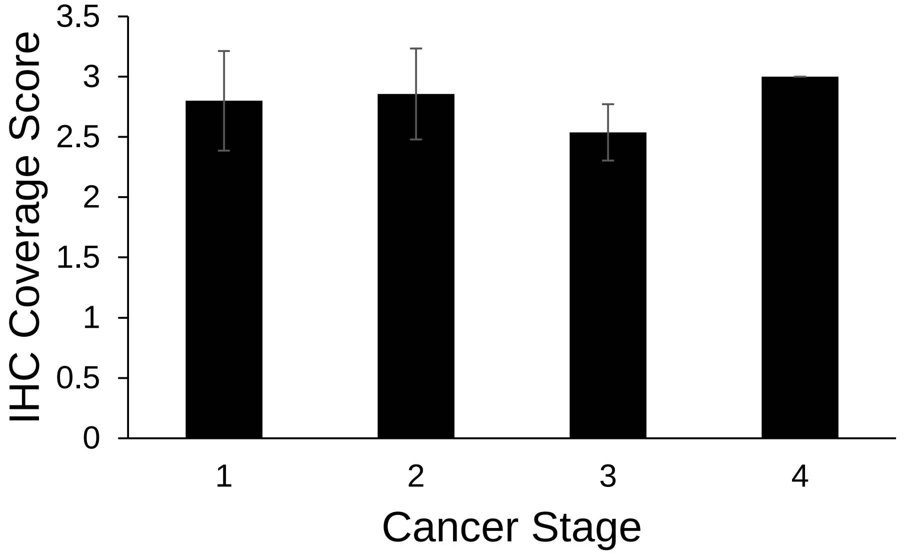

Spearman Analysis

ρ (rho) =	-0.0289
t =	-0.1783
p-value =	0.8594

	n	Average	St. Dev	St. Error
1	15	2.80	0.41	0.41
2	7	2.86	0.38	0.38
3	13	2.54	0.66	0.23
4	5	3.00	0.00	0.00

1-Way Anova

	SS	df	MS	F	p
Between	1.01	3	0.34	1.432	0.249
Within	8.45	36	0.24		
Total	9.45	39			

SI Figure 82. Correlation analysis of CCK2R coverage score in liver cancer (hepatocellular carcinoma) versus cancer stage. IHC was performed on liver cancer (hepatocellular carcinoma) tissue sections using a monoclonal antibody raised against CCK2R. The coverage score was graded on a scale of 0 to 3 and plotted (error bars represent standard error of the mean). A Spearman analysis was used to determine if there was a statistically significant correlation and a 1-way ANOVA was used to determine if there were any significant differences between groups.

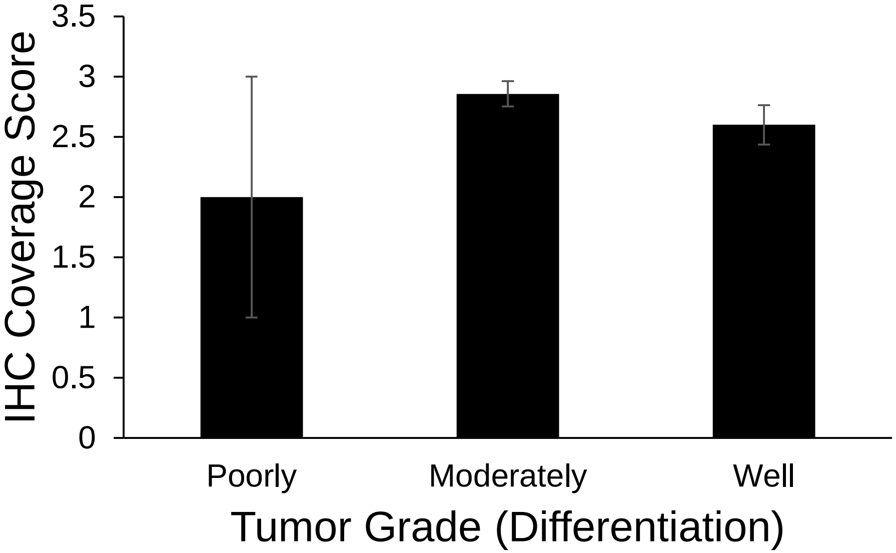

Spearman Analysis

ρ (rho) =	-0.1693
t =	-0.9716
p-value =	0.3383

	n	Average	St. Dev	St. Error
Poorly Differentiated	3	2.00	1.73	1.00
Moderately Differentiated	21	2.86	0.48	0.10
Well Differentiated	10	2.60	0.52	0.16

1-Way Anova

	SS	df	MS	F	p
Between	2.1	2	1.05	2.505	0.098
Within	13.0	31	0.42		
Total	15.1	33			

SI Figure 83. Correlation analysis of CCK2R coverage score in liver cancer (hepatocellular carcinoma) versus primary tumor grade. IHC was performed on liver cancer (hepatocellular carcinoma) tissue sections using a monoclonal antibody raised against CCK2R. The coverage score was graded on a scale of 0 to 3 and plotted (error bars represent standard error of the mean). A Spearman analysis was used to determine if there was a statistically significant correlation and a 1-way ANOVA was used to determine if there were any significant differences between groups.

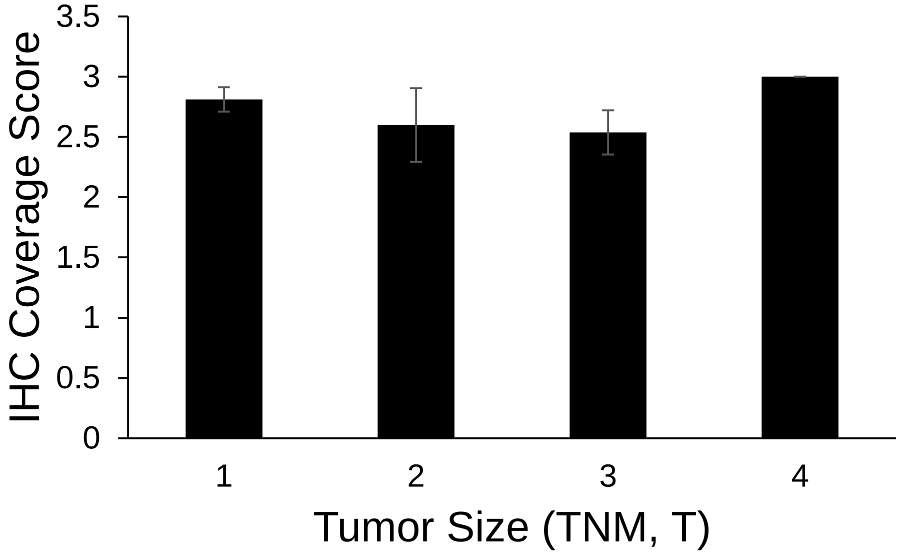

Spearman Analysis

Spearman Analysis	ρ (rho) =	-0.0970
	t =	-0.6161
	p-value =	0.5412

	n	Average	St. Dev	St. Error
1	16	2.81	0.40	0.10
2	10	2.60	0.97	0.31
3	13	2.54	0.66	0.18
4	3	3.00	0.00	0.00

1-Way Anova

	SS	df	MS	F	p
Between	0.89	3	0.297	0.702	0.557
Within	16.10	38	0.424		
Total	16.99	41			

SI Figure 84. Correlation analysis of CCK2R coverage score in liver cancer (hepatocellular carcinoma) versus primary tumor size. IHC was performed on liver cancer (hepatocellular carcinoma) tissue sections using a monoclonal antibody raised against CCK2R. The coverage score was graded on a scale of 0 to 3 and plotted (error bars represent standard error of the mean). A Spearman analysis was used to determine if there was a statistically significant correlation and a 1-way ANOVA was used to determine if there were any significant differences between groups.

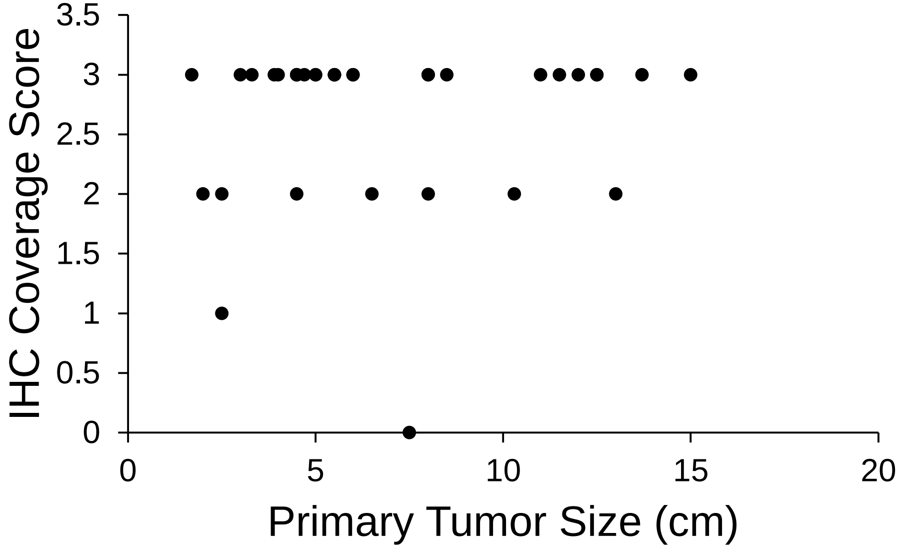

Spearman Analysis		
	ρ (rho) =	0.1080
	t =	0.6239
	p-value =	0.5368

	n	Average	St. Dev	St. Error
0	1	7.50	-	-
1	1	2.50	-	-
2	7	6.69	4.06	1.53
3	26	7.13	3.75	0.74

t-test

p-value = 0.7876

SI Figure 85. Correlation analysis of CCK2R coverage score in liver cancer (hepatocellular carcinoma) versus size of primary tumor (length of longest side). IHC was performed on liver cancer (hepatocellular carcinoma) tissue sections using a monoclonal antibody raised against CCK2R. The coverage score was graded on a scale of 0 to 3 and plotted. A Spearman analysis was used to determine if there was a statistically significant correlation and a t-test was used to determine if there were any significant differences between groups.

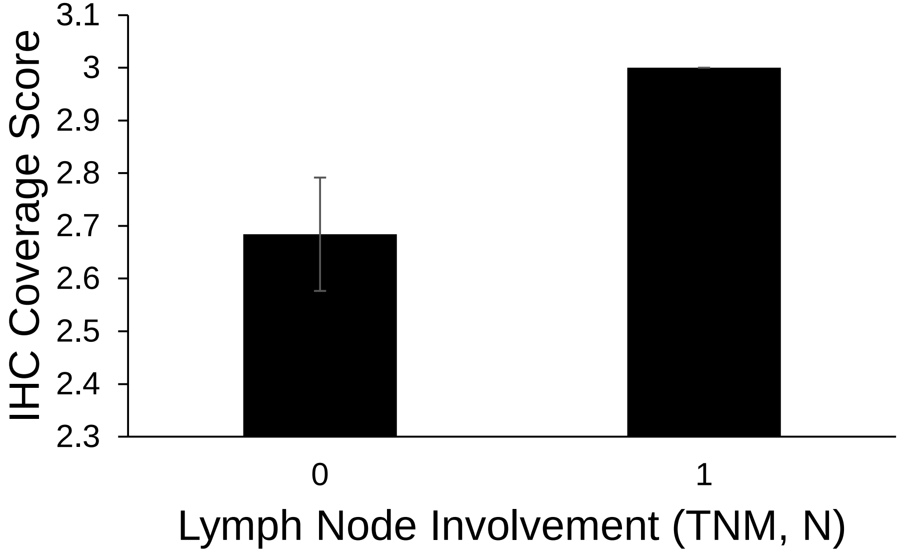

Spearman Analysis

Spearman Analysis	ρ (rho) =	-
	t =	-
	p-value =	-

	n	Average	St. Dev	St. Error
0	38	2.68	0.66	0.11
1	1	3.00	-	-

t-test

p-value = -

SI Figure 86. Correlation analysis of CCK2R coverage score in liver cancer (hepatocellular carcinoma) versus lymph node involvement. IHC was performed on liver cancer (hepatocellular carcinoma) tissue sections using a monoclonal antibody raised against CCK2R. The coverage score was graded on a scale of 0 to 3 and plotted (error bars represent standard error of the mean). No statistical tests could be performed.

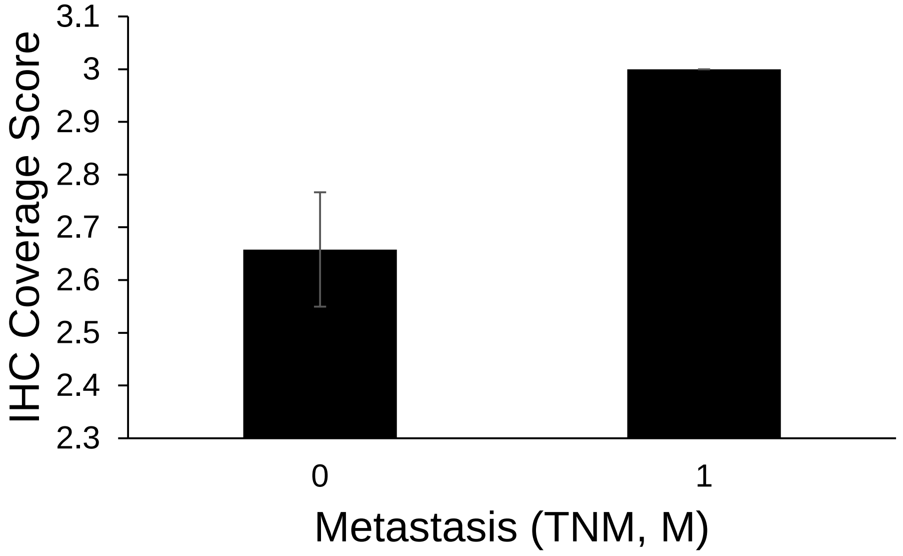

Spearman Analysis

ρ (rho) = -

t = -

p-value = -

	n	Average	St. Dev	St. Error
0	38	2.66	0.67	0.11
1	2	3.00	-	-

t-test

p-value = -

SI Figure 87. Correlation analysis of CCK2R coverage score in liver cancer (hepatocellular carcinoma) versus metastases. IHC was performed on liver cancer (hepatocellular carcinoma) tissue sections using a monoclonal antibody raised against CCK2R. The coverage score was graded on a scale of 0 to 3 and plotted (error bars represent standard error of the mean). No statistical tests could be performed.

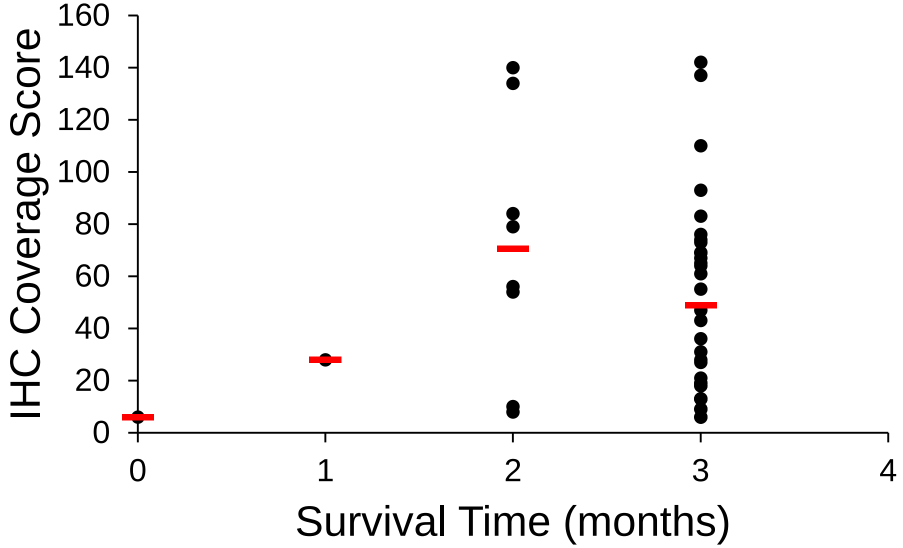

Spearman Analysis

ρ (rho) = -0.0259
t = -0.1657
p-value = 0.8692

	n	Average	St. Dev	St. Error
0	1	6.00	-	-
1	1	28.00	-	-
2	8	70.63	49.52	14.93
3	33	48.91	36.94	10.66

t-test

p-value = 0.1709

SI Figure 88. Correlation analysis of CCK2R coverage score in liver cancer (hepatocellular carcinoma) versus survival time after diagnosis. IHC was performed on liver cancer (hepatocellular carcinoma) tissue sections using a monoclonal antibody raised against CCK2R. The coverage score was graded on a scale of 0 to 3 and plotted (red bars represent population mean). A Spearman analysis was used to determine if there was a statistically significant correlation and a t-test was used to determine if there were any significant differences between groups.

Liver Cancer
Total Staining Score Correlations

Liver Cancer

	n	Average	St. Dev	St. Error
Total Score	43	5.40	2.65	0.40

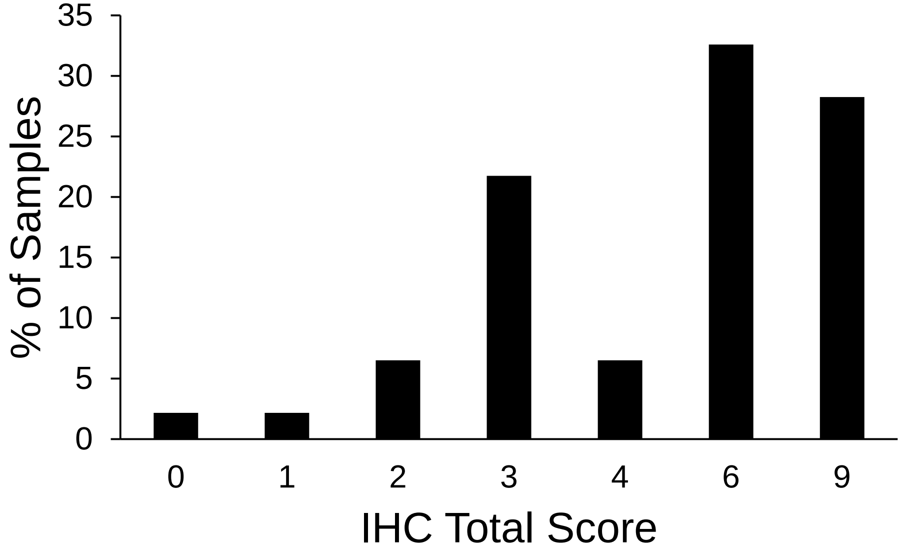

	Total Staining Score						
	0	1	2	3	4	6	9
n	1	1	3	10	3	15	13
%	2.17	2.17	6.52	21.74	6.52	32.61	28.26

Normal Liver

	n	Average	St. Dev	St. Error
Total Score	22	5.50	3.14	0.67

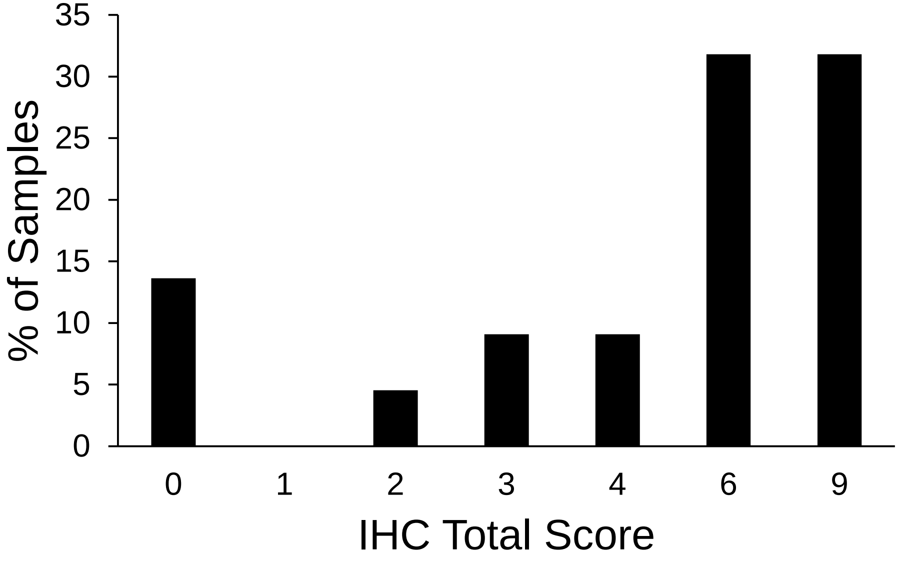

	Total Staining Score						
	0	1	2	3	4	6	9
n	3	0	1	2	2	7	7
%	13.64	0.00	4.55	9.09	9.09	31.82	31.82

SI Figure 89. CCK2R Total Staining Score for cancer and normal tissue from the liver. IHC was performed on tissue sections using a monoclonal antibody raised against CCK2R. The staining intensity and coverage score was multiplied to obtain the total staining score.

Liver (hepatocellular carcinoma) - Total Staining Score

	Sex	Age at Diagnosis	Primary Tumor Type	Primary Tumor Site	Stage	Grade	Tumor Size (TNM, T)	Tumor Size (longest dimension)	Lymph Node Involvement (TNM, N)	Metastatic (TNM, M)	Metastatic Site	Survival after Diagnosis	Survival after Stage IV Diagnosis
**Spearman Correlation**	N.A.	No 0.564	N.A.	N.A.	No 0.623	No 0.606	No 0.343	No 0.720	N.D.	N.D.	N.A.	No 0.428	N.D.
**ANOVA/t-test**	No 0.826	No 0.660	N.D.	N.D.	No 0.991	No 0.265	No 0.114	No 0.892	N.D.	N.D.	N.D.	No 0.989	N.D.

SI Figure 90. Total staining score correlation summary of CCK2R in liver cancer (hepatocellular carcinoma). IHC was performed on liver tumor tissue sections using a monoclonal antibody raised against CCK2R. The total staining score was compared against available patient data. If appropriate, a spearman analysis was used to determine if any significant correlation exists while a 1-way ANOVA or t-test was used to determine if a significant difference exists between groups. N.A. – not applicable (this statistical test was not applicable to this data set). Whether the test was statistically significant and the p-value is listed. N.D. – not determined (this statistical test could not be performed, generally due to a lack of the number of samples within a group or all data was in a single group).

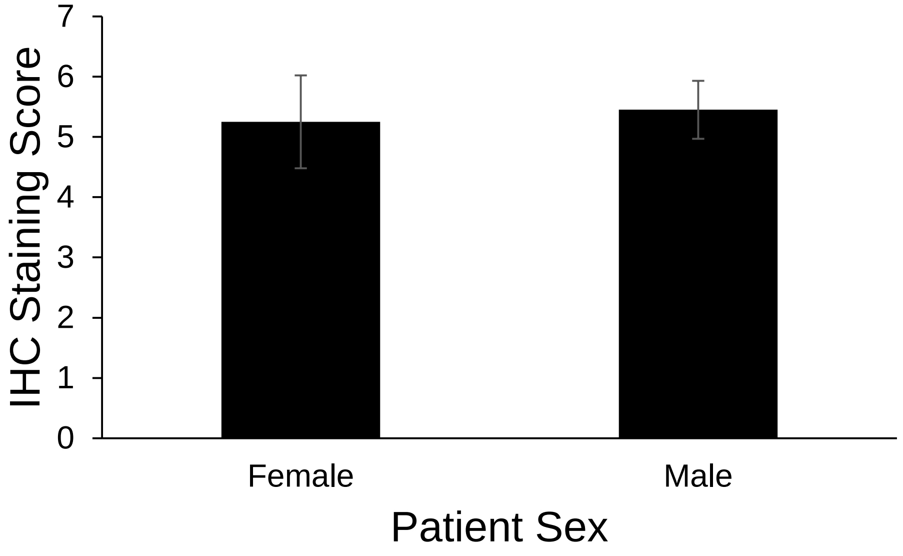

Spearman Analysis

ρ (rho) =	-
t =	-
p-value =	-

	n	Average	St. Dev	St. Error
Female	12	5.25	2.67	0.77
Male	31	5.45	2.68	0.48

t-test

p-value = 0.8258

SI Figure 91. Correlation analysis of CCK2R total staining score in liver cancer (hepatocellular carcinoma) versus patient sex. IHC was performed on liver cancer (hepatocellular carcinoma) tissue sections using a monoclonal antibody raised against CCK2R. The total staining score was derived by multiplying the staining intensity with the coverage score (error bars represent standard error of the mean). A t-test was used to determine if there were any significant differences between groups.

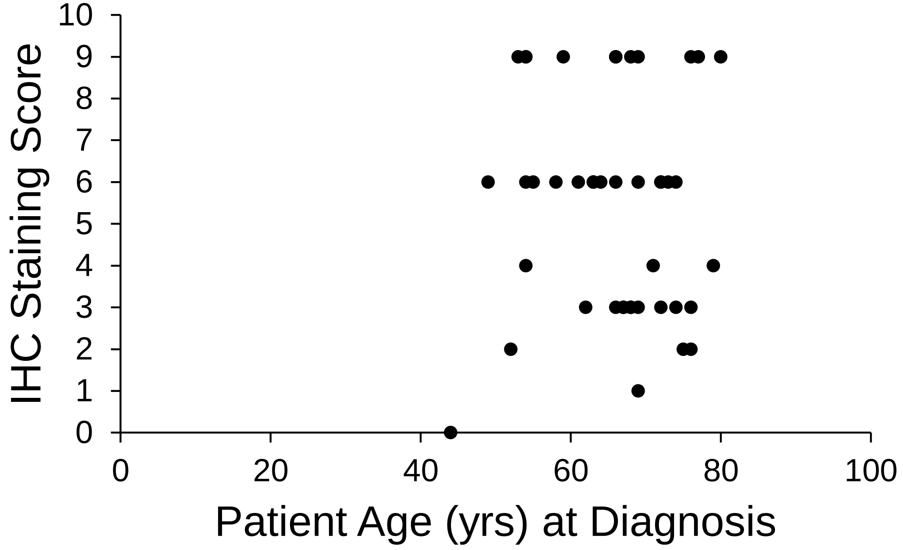

Spearman Analysis

ρ (rho) =	-0.0906
t =	-0.5823
p-value =	0.5635

	n	Average	St. Dev	St. Error
0	1	44.00	-	-
1	1	69.00	-	-
2	3	67.67	13.58	7.84
3	10	68.90	4.09	1.29
4	3	68.00	12.77	7.37
6	14	63.79	7.80	2.08
9	11	65.64	9.67	2.91

1-Way Anova

	SS	df	MS	F	p
Between	174	4	43.4	0.608	0.660
Within	2,572	36	71.4		
Total	2,745	40			

SI Figure 92. Correlation analysis of CCK2R total staining score in liver cancer (hepatocellular carcinoma) versus patient age at diagnosis. IHC was performed on liver cancer (hepatocellular carcinoma) tissue sections using a monoclonal antibody raised against CCK2R. The total staining score was derived by multiplying the staining intensity with the coverage score. A Spearman analysis was used to determine if there was a statistically significant correlation and a 1-way ANOVA was used to determine if there were any significant differences between groups.

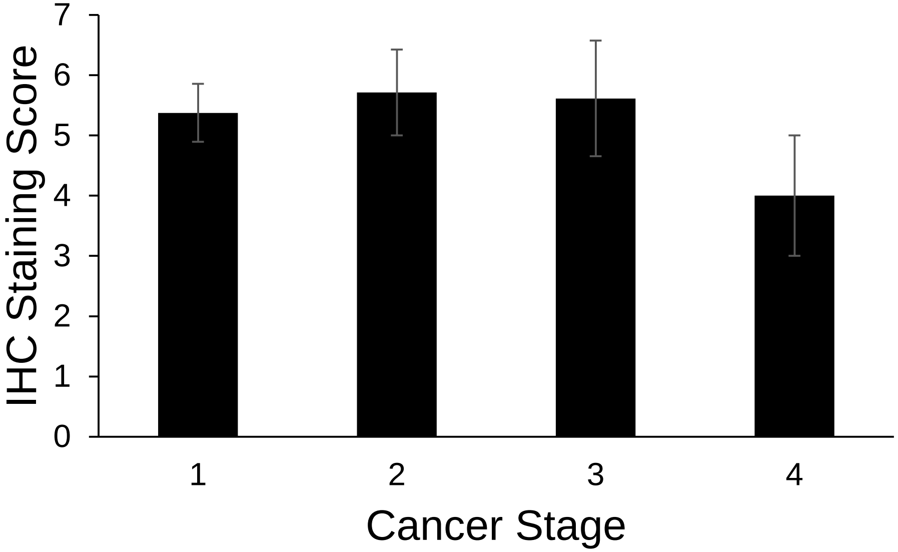

Spearman Analysis		
	ρ (rho) =	-0.0811
	t =	-0.4951
	p-value =	0.6234

	n	Average	St. Dev	St. Error
1	16	5.38	1.93	0.48
2	7	5.71	1.89	0.71
3	13	5.62	3.45	0.96
4	3	4.00	1.73	1.00

1-Way Anova

	SS	df	MS	F	p
Between	0.7	3	0.24	0.034	0.991
Within	243.9	35	6.97		
Total	244.7	38			

SI Figure 93. Correlation analysis of CCK2R total staining score in liver cancer (hepatocellular carcinoma) versus cancer stage. IHC was performed on liver cancer (hepatocellular carcinoma) tissue sections using a monoclonal antibody raised against CCK2R. The total staining score was derived by multiplying the staining intensity with the coverage score (error bars represent standard error of the mean). A Spearman analysis was used to determine if there was a statistically significant correlation and a 1-way ANOVA was used to determine if there were any significant differences between groups.

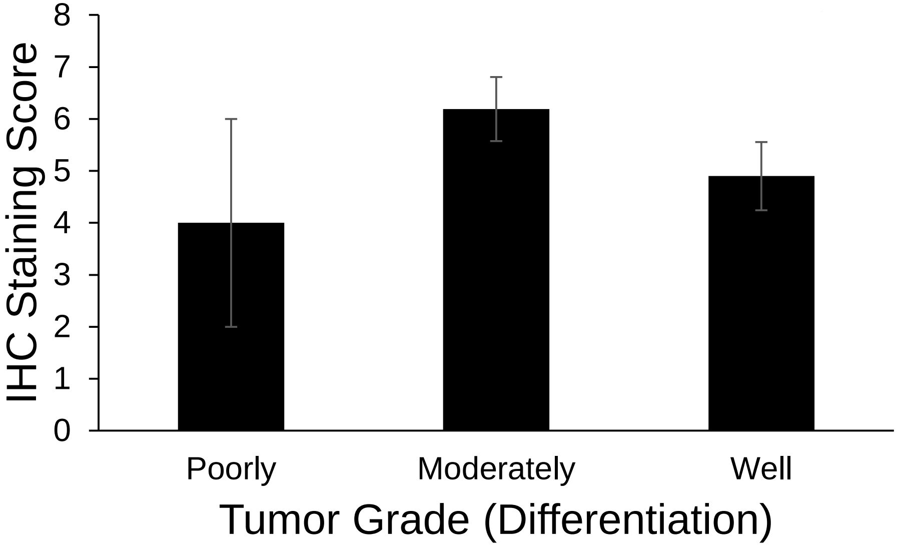

Spearman Analysis

ρ (rho) =	-0.0918
t =	-0.5214
p-value =	0.6056

	n	Average	St. Dev	St. Error
Poorly Differentiated	3	4.00	3.46	2.00
Moderately Differentiated	21	6.19	2.82	0.62
Well Differentiated	10	4.90	2.08	0.66

1-Way Anova

	SS	df	MS	F	p
Between	19.9	2	9.94	1.388	0.265
Within	221.9	31	7.16		
Total	241.8	33			

SI Figure 94. Correlation analysis of CCK2R total staining score in liver cancer (hepatocellular carcinoma) versus primary tumor grade. IHC was performed on liver cancer (hepatocellular carcinoma) tissue sections using a monoclonal antibody raised against CCK2R. The total staining score was derived by multiplying the staining intensity with the coverage score (error bars represent standard error of the mean). A Spearman analysis was used to determine if there was a statistically significant correlation and a 1-way ANOVA was used to determine if there were any significant differences between groups.

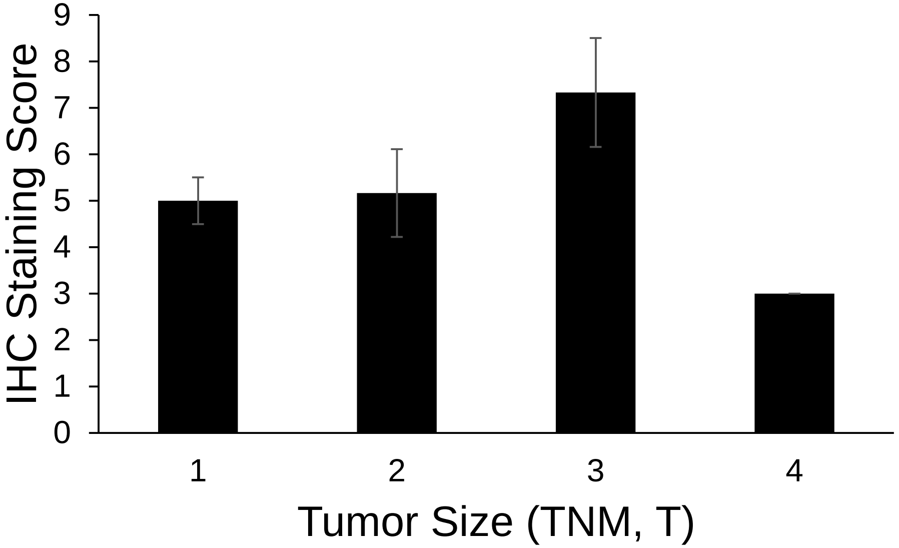

Spearman Analysis		
	ρ (rho) =	0.1934
	t =	0.9657
	p-value =	0.3434

	n	Average	St. Dev	St. Error
1	13	5.00	1.83	0.51
2	6	5.17	2.32	0.95
3	6	7.33	2.88	1.17
4	1	3.00	-	-

1-Way Anova

	SS	df	MS	F	p
Between	23.7	2	11.87	2.406	0.114
Within	108.6	22	4.94		
Total	132.3	24			

SI Figure 95. Correlation analysis of CCK2R total staining score in liver cancer (hepatocellular carcinoma) versus primary tumor size. IHC was performed on liver cancer (hepatocellular carcinoma) tissue sections using a monoclonal antibody raised against CCK2R. The total staining score was derived by multiplying the staining intensity with the coverage score (error bars represent standard error of the mean). A Spearman analysis was used to determine if there was a statistically significant correlation and a 1-way ANOVA was used to determine if there were any significant differences between groups.

Spearman Analysis

ρ (rho) =	-0.0629
t =	-0.3620
p-value =	0.7196

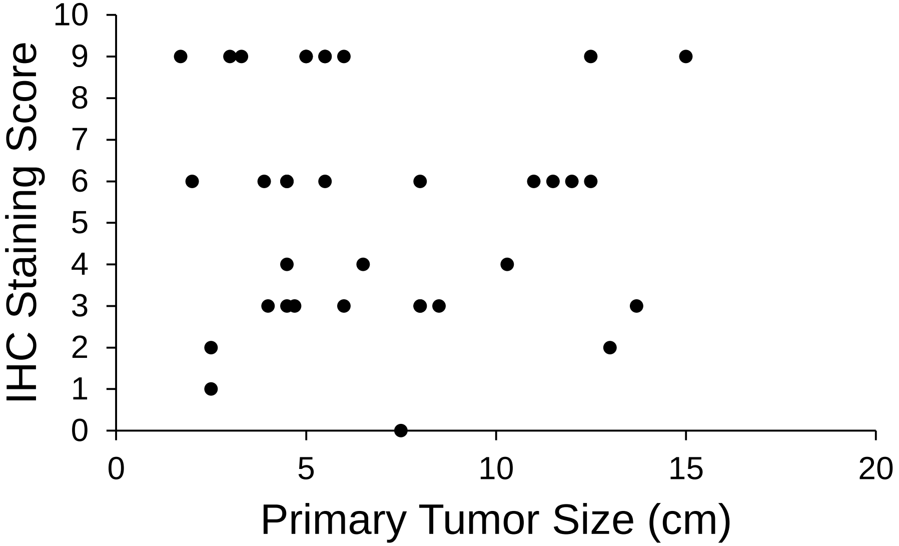

	n	Average	St. Dev	St. Error
0	1	7.50	-	-
1	1	2.50	-	-
2	2	7.75	-	-
3	8	7.18	3.17	1.12
4	3	7.10	2.95	1.70
6	10	7.54	3.93	1.24
9	10	6.25	4.22	1.33

1-Way Anova

	SS	df	MS	F	p
Between	8.8	3	2.94	0.205	0.892
Within	387.0	27	14.33		
Total	395.9	30			

SI Figure 96. Correlation analysis of CCK2R total staining score in liver cancer (hepatocellular carcinoma) versus size of primary tumor (length of longest side). IHC was performed on liver cancer (hepatocellular carcinoma) tissue sections using a monoclonal antibody raised against CCK2R. The total staining score was derived by multiplying the staining intensity with the coverage score. A Spearman analysis was used to determine if there was a statistically significant correlation and a 1-way ANOVA was used to determine if there were any significant differences between groups.

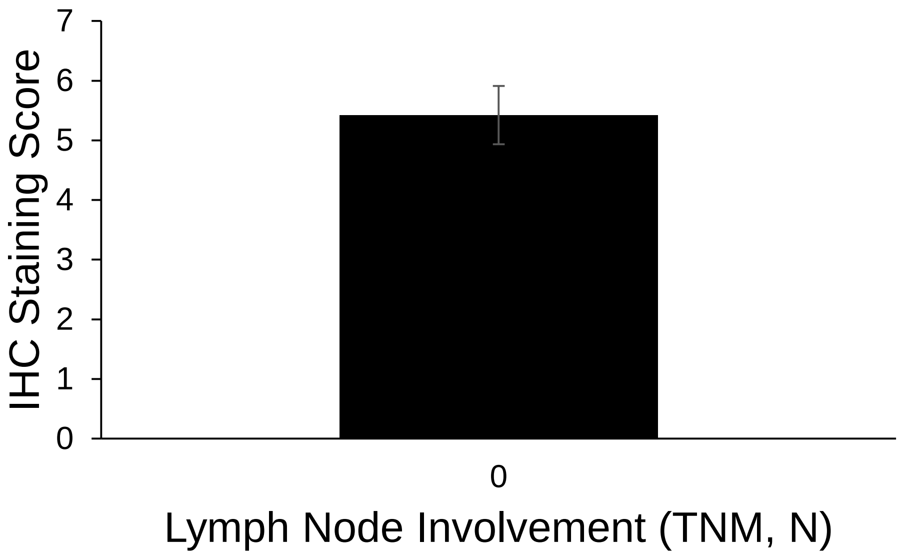

Spearman Analysis

ρ (rho) = -

t = -

p-value = -

	n	Average	St. Dev	St. Error
0	26	5.42	2.50	0.49

t-test

p-value = -

SI Figure 97. Correlation analysis of CCK2R total staining score in liver cancer (hepatocellular carcinoma) versus lymph node involvement. IHC was performed on liver cancer (hepatocellular carcinoma) tissue sections using a monoclonal antibody raised against CCK2R. The total staining score was derived by multiplying the staining intensity with the coverage score (error bars represent standard error of the mean). No statistical tests could be performed.

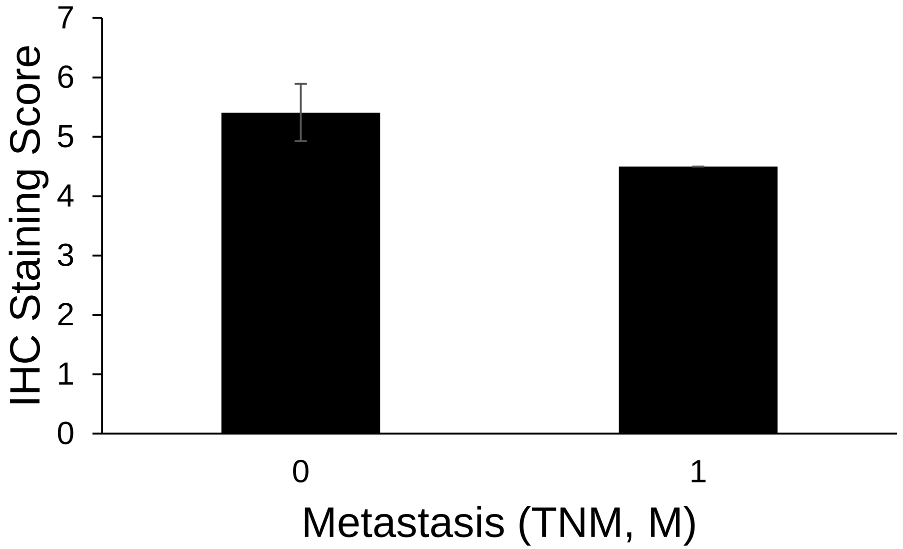

Spearman Analysis		
	ρ (rho) =	-
	t =	-
	p-value =	-

	n	Average	St. Dev	St. Error
0	27	5.41	2.50	0.48
1	2	4.50	-	-

t-test

p-value = -

SI Figure 98. Correlation analysis of CCK2R total staining score in liver cancer (hepatocellular carcinoma) versus metastases. IHC was performed on liver cancer (hepatocellular carcinoma) tissue sections using a monoclonal antibody raised against CCK2R. The total staining score was derived by multiplying the staining intensity with the coverage score (error bars represent standard error of the mean). No statistical tests could be performed.

Spearman Analysis		
	ρ (rho) =	0.1240
	t =	0.7999
	p-value =	0.4283

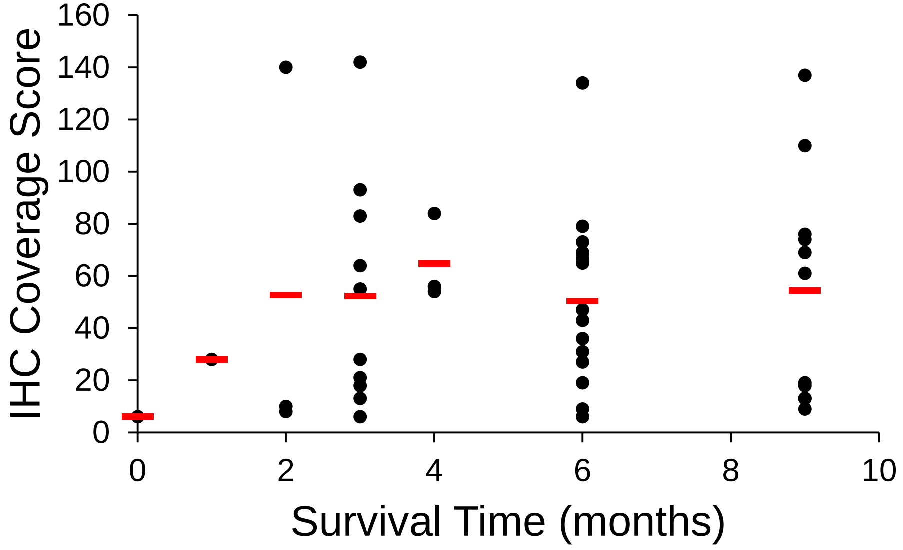

	n	Average	St. Dev	St. Error
0	1	6.00	-	-
1	1	28.00	-	-
2	3	52.67	75.64	43.67
3	10	52.30	43.75	13.83
4	3	64.67	16.77	9.68
6	14	50.36	33.99	9.08
9	11	54.45	43.69	13.17

1-Way Anova

	SS	df	MS	F	p
Between	534	4	133	0.076	0.989
Within	63,339	36	1,759		
Total	63,873	40			

SI Figure 99. Correlation analysis of CCK2R total staining score in liver cancer (hepatocellular carcinoma) versus survival time after diagnosis. IHC was performed on liver cancer (hepatocellular carcinoma) tissue sections using a monoclonal antibody raised against CCK2R. The total staining score was derived by multiplying the staining intensity with the coverage score (red bars represent population mean). A Spearman analysis was used to determine if there was a statistically significant correlation and a 1-way ANOVA was used to determine if there were any significant differences between groups.

Lung Cancer

Lung Cancer Images

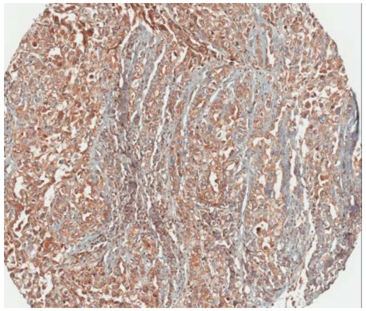
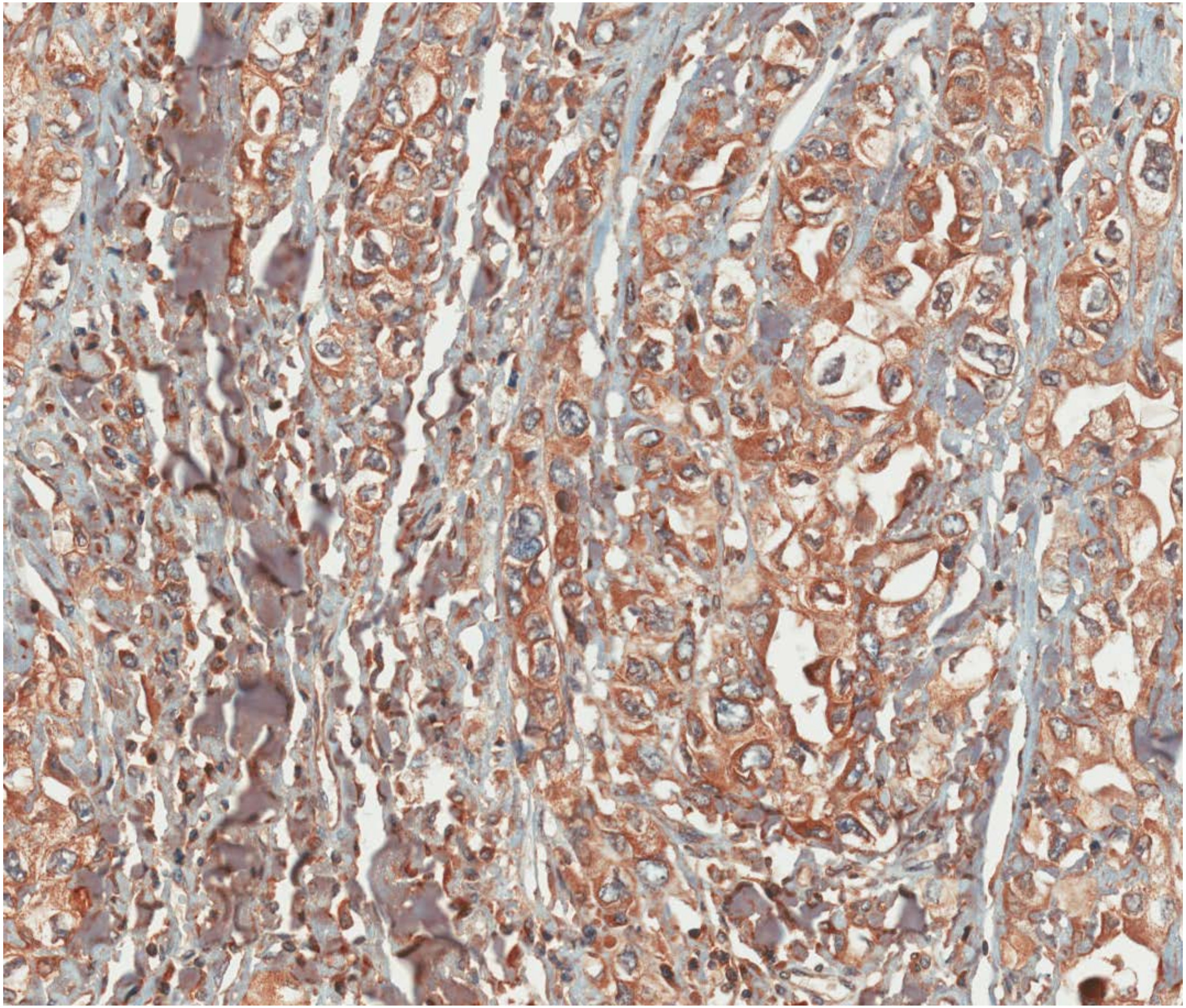

SI Figure 100. Example image of stained tissue from lung cancer. IHC was performed on tissue sections using a monoclonal antibody raised against CCK2R.

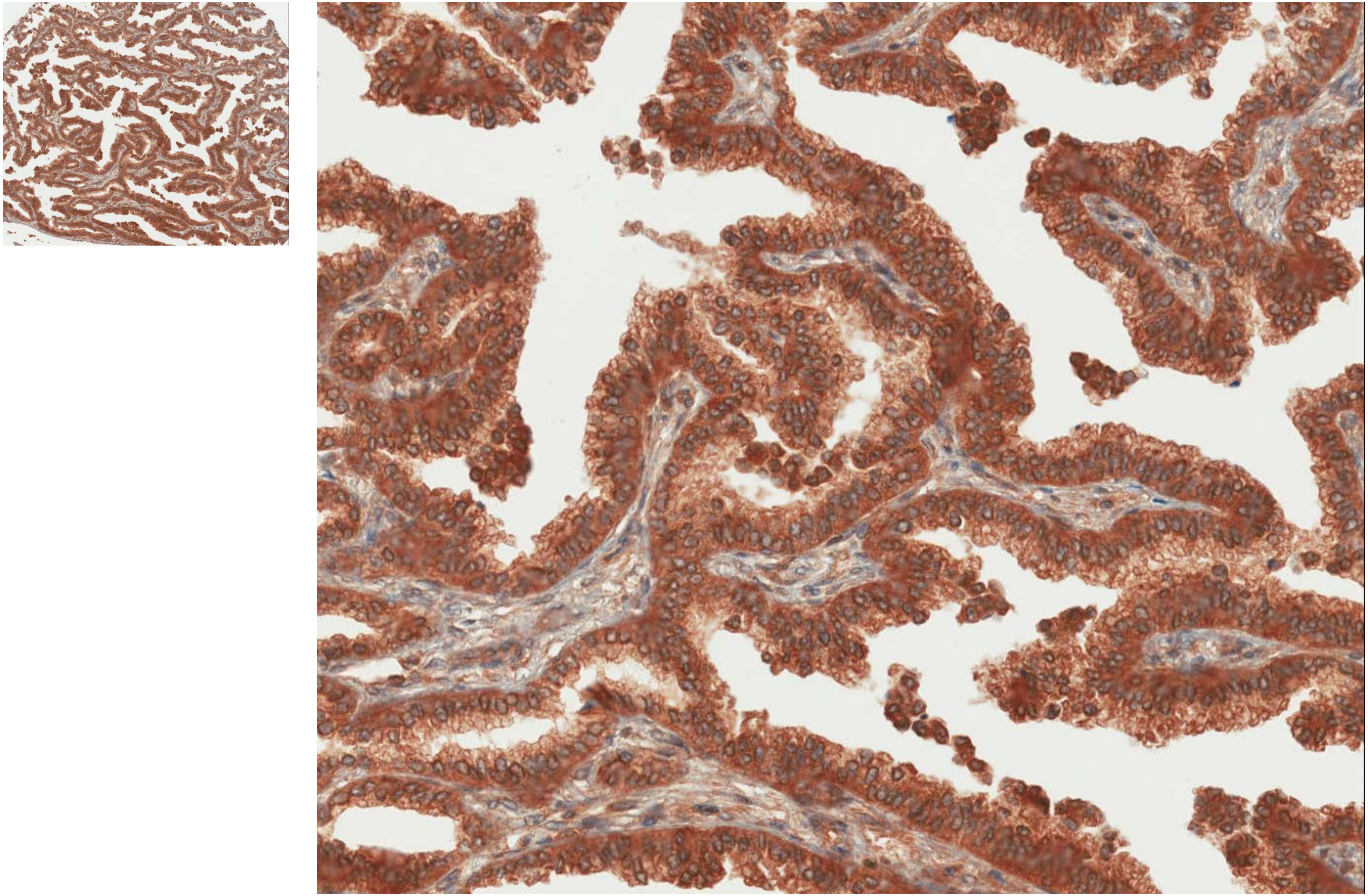

SI Figure 101. Example image of stained tissue from lung cancer. IHC was performed on tissue sections using a monoclonal antibody raised against CCK2R.

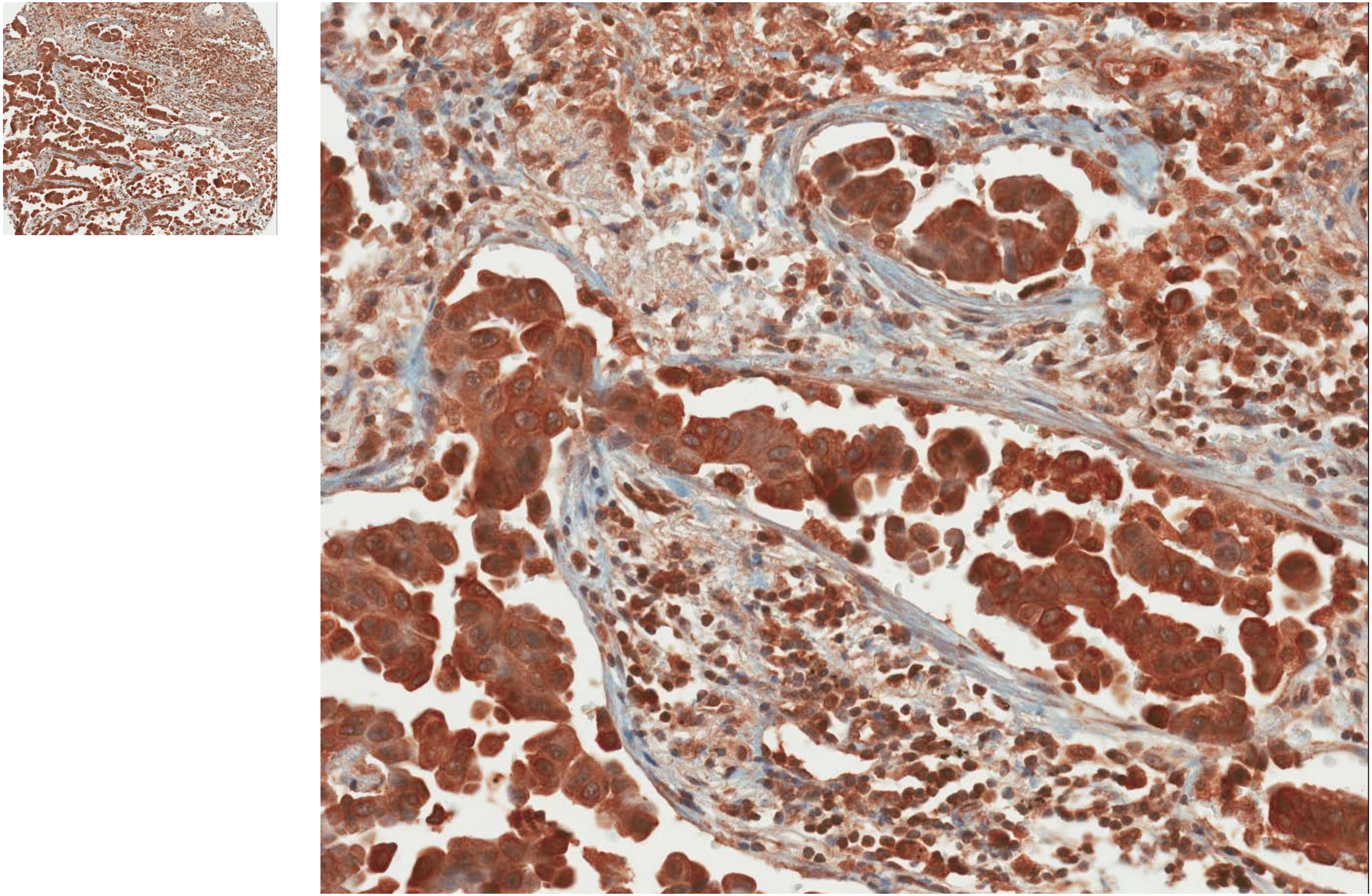

SI Figure 102. Example image of stained tissue from lung cancer. IHC was performed on tissue sections using a monoclonal antibody raised against CCK2R.

Lung Cancer
Overall Summary

Lung - Spearman Correlation

	Sex	Age at Diagnosis	Primary Tumor Type	Primary Tumor Site	Stage	Grade	Tumor Size (TNM, T)	Tumor Size (longest dimension)	Lymph Node Involvement (TNM, N)	Metastatic (TNM, M)	Metastatic Site	Survival after Diagnosis	Survival after Stage IV Diagnosis
**Staining Intensity**	N.A.	No 0.685	N.A.	N.A.	No 0.440	No 0.942	No 0.435	No 0.573	No 0.188	No 0.556	N.A.	No 0.6679	No 0.449
**Coverage Score**	N.A.	No 0.734	N.A.	N.A.	**Yes 0.014**	No 0.341	No 0.348	No 0.8542	No 0.080	No 0.330	N.A.	No 0.855	No 0.300
**Total Staining Score**	N.A.	No 0.732	N.A.	N.A.	No 0.509	No 0.990	No 0.452	No 0.546	No 0.171	No 0.558	N.A.	No 0.663	No 0.418

Lung - Spearman Correlation - ANOVA/t-test

	Sex	Age at Diagnosis	Primary Tumor Type	Primary Tumor Site	Stage	Grade	Tumor Size (TNM, T)	Tumor Size (longest dimension)	Lymph Node Involvement (TNM, N)	Metastatic (TNM, M)	Metastatic Site	Survival after Diagnosis	Survival after Stage IV Diagnosis
**Staining Intensity**	No 0.931	No 0.797	No 0.868	No 0.510	No 0.805	No 0.865	No 0.409	No 0.457	No 0.257	No 0.899	No 0.053	No 0.966	No 0.821
**Coverage Score**	No 0.130	No 0.473	No 0.136	No 0.531	No 0.067	No 0.226	No 0.625	No 0.391	No 0.230	No 0.215	No 1.000	No 0.826	No 0.4123
**Total Staining Score**	No 0.978	No 0.786	No 0.364	No 0.511	No 0.838	No 0.855	No 0.444	No 0.465	No 0.238	No 0.929	**Yes 0.012**	No 0.966	No 0.845

SI Figure 103. Correlation summary of CCK2R in lung cancer. IHC was performed on lung tumor tissue sections using a monoclonal antibody raised against CCK2R. The staining intensity, coverage score and total staining score were compared against available patient data. If appropriate, a spearman analysis was used to determine if any significant correlation exists while a 1-way ANOVA or t-test was used to determine if a significant difference exists between groups. N.A. – not applicable (this statistical test was not applicable to this data set). Whether the test was statistically significant and the p-value is listed. N.D. – not determined (this statistical test could not be performed, generally due to a lack of the number of samples within a group or all data was in a single group).

Lung Caner
Staining Intensity

Lung Cancer

	n	Average	St. Dev	St. Error
Staining Intensity	102	1.59	0.64	0.06

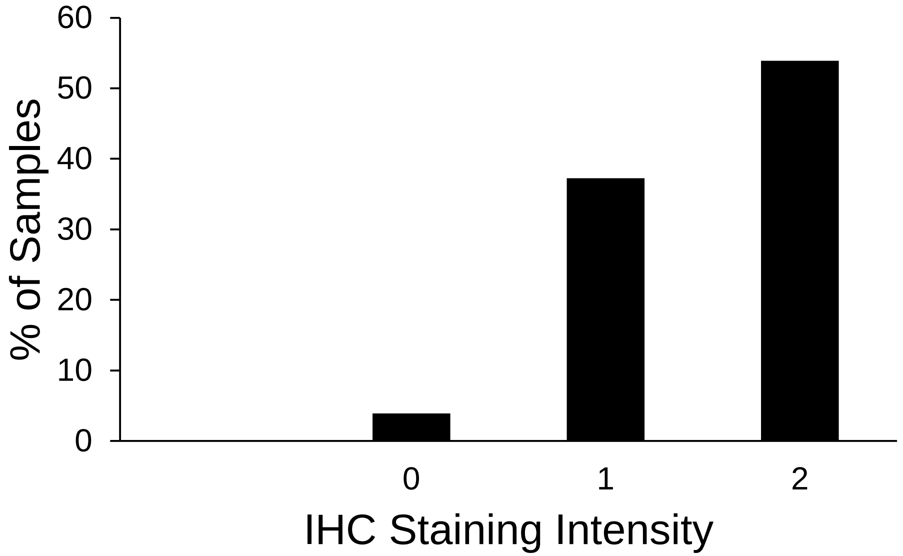

	Staining Intensity			
	0	1	2	3
n	4	38	55	5
%	3.92	37.25	53.92	4.90

Normal Lung

	n	Average	St. Dev	St. Error
Staining Intensity	8	0.38	1.06	0.38

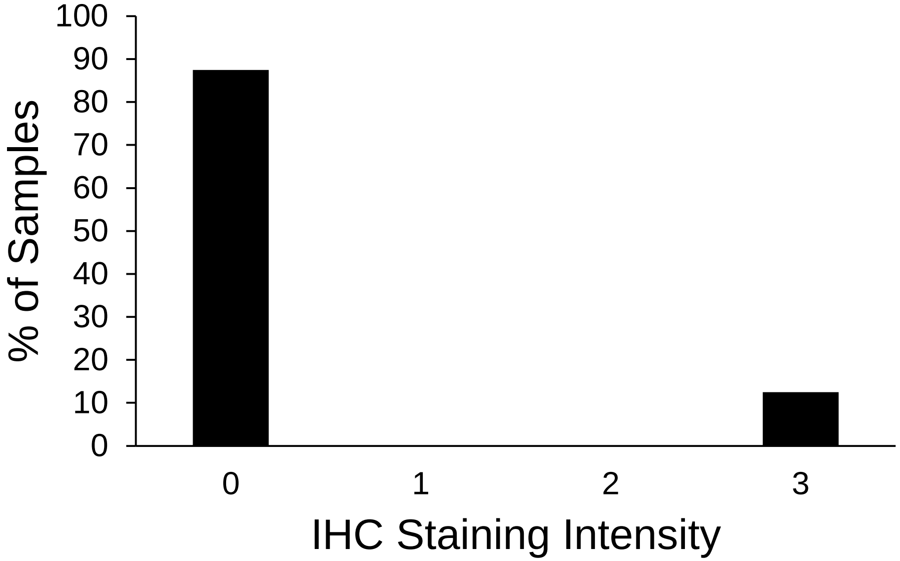

	Staining Intensity			
	0	1	2	3
n	7	0	0	1
%	87.50	0.00	0.00	12.50

SI Figure 104. CCK2R Staining Intensity for cancer and normal tissue from the lung. IHC was performed on tissue sections using a monoclonal antibody raised against CCK2R. The intensity of staining was graded on a scale of 0 to 3 and plotted.

Lung Cancer - Staining Intensity

	Sex	Age at Diagnosis	Primary Tumor Type	Primary Tumor Site	Stage	Grade	Tumor Size (TNM, T)	Tumor Size (longest dimension)	Lymph Node Involvement (TNM, N)	Metastatic (TNM, M)	Metastatic Site	Survival after Diagnosis	Survival after Stage IV Diagnosis
**Spearman Correlation**	N.A.	No 0.685	N.A.	N.A.	No 0.440	No 0.942	No 0.435	No 0.573	No 0.188	No 0.556	N.A.	No 0.6679	No 0.449
**ANOVA/t-test**	No 0.931	No 0.797	No 0.868	No 0.510	No 0.805	No 0.865	No 0.409	No 0.457	No 0.257	No 0.899	No 0.053	No 0.966	No 0.821

SI Figure 105. Staining intensity correlation summary of CCK2R in lung cancer. IHC was performed on lung tumor tissue sections using a monoclonal antibody raised against CCK2R. The staining intensity was compared against available patient data. If appropriate, a spearman analysis was used to determine if any significant correlation exists while a 1-way ANOVA or t-test was used to determine if a significant difference exists between groups. Whether the test was statistically significant and the p-value is listed. N.A. – not applicable (this statistical test was not applicable to this data set). N.D. – not determined (this statistical test could not be performed, generally due to a lack of the number of samples within a group or all data was in a single group).

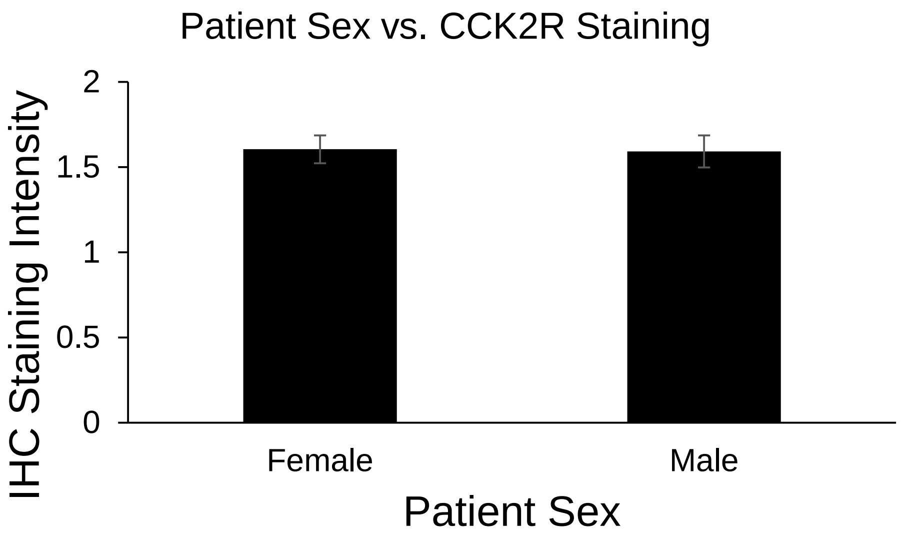

Spearman Analysis		
	ρ (rho) =	-
	t =	-
	p-value =	-

	n	Average	St. Dev	St. Error
Female	43	1.60	0.54	0.08
Male	59	1.59	0.72	0.09

t-test

p-value = 0.9305

SI Figure 106. Correlation analysis of CCK2R staining intensity in lung cancer versus patient sex. IHC was performed on lung cancer tissue sections using a monoclonal antibody raised against CCK2R. The staining intensity was graded on a scale of 0 to 3 and plotted (error bars represent standard error of the mean). A t-test was used to determine if there were any significant differences between groups.

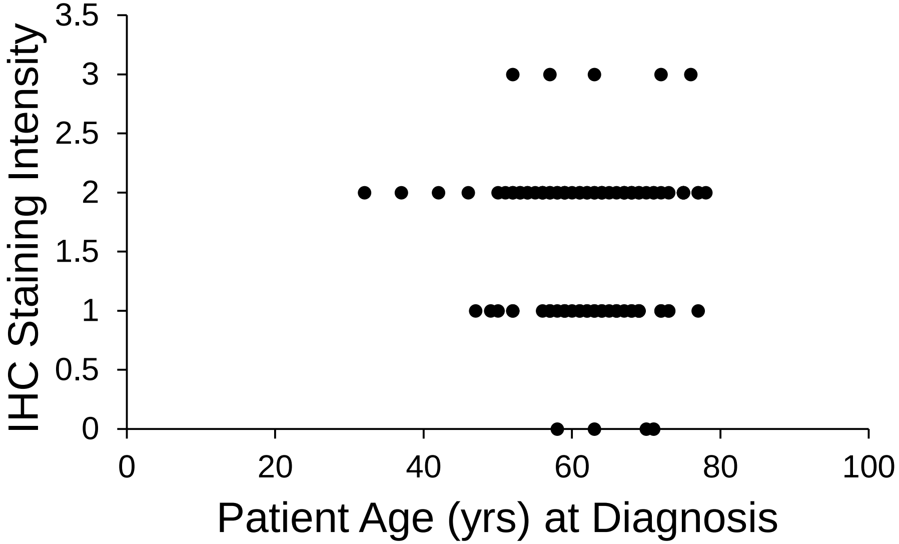

Spearman Analysis

ρ (rho) =	-0.0406
t =	-0.4065
p-value =	0.6853

	n	Average	St. Dev	St. Error
0	4	65.50	6.14	3.07
1	38	62.66	7.18	1.17
2	55	61.69	9.96	1.34
3	5	64.00	10.02	4.48

1-Way Anova

	SS	df	MS	F	p
Between	81	3	27	0.339	0.797
Within	7,779	98	79		
Total	7,860	101			

SI Figure 107. Correlation analysis of CCK2R staining intensity in lung cancer versus patient age at diagnosis. IHC was performed on lung cancer tissue sections using a monoclonal antibody raised against CCK2R. The staining intensity was graded on a scale of 0 to 3 and plotted. A Spearman analysis was used to determine if there was a statistically significant correlation and a 1-way ANOVA was used to determine if there were any significant differences between groups.

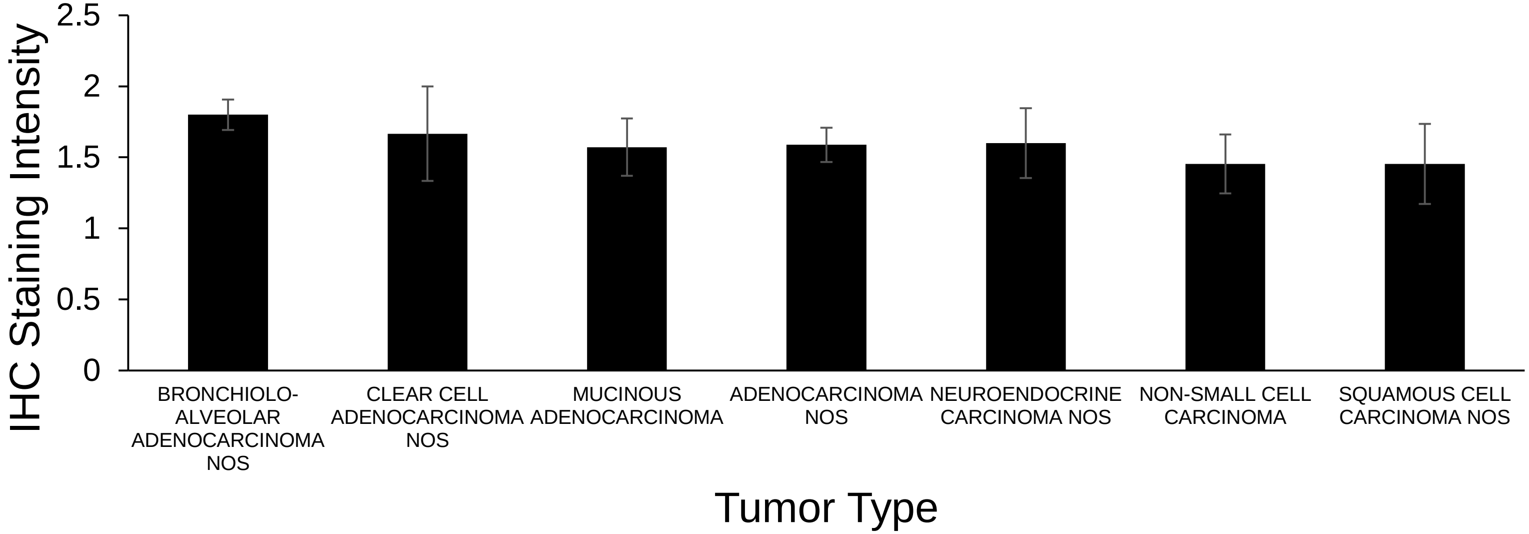

Spearman Analysis

ρ (rho) =	-
t =	-
p-value =	-

	n	Average	St. Dev	St. Error
BRONCHIOLO- ALVEOLAR ADENOCARCINOMA NOS	15	1.80	0.41	0.11
CLEAR CELL ADENOCARCINOMA NOS	3	1.67	0.58	0.33
MUCINOUS ADENOCARCINOMA	7	1.57	0.53	0.20
ADENOCARCINOMA NOS	34	1.59	0.70	0.12
NEUROENDOCRINE CARCINOMA NOS	5	1.60	0.55	0.24
NON-SMALL CELL CARCINOMA	11	1.45	0.69	0.21
SQUAMOUS CELL CARCINOMA NOS	10	1.45	0.93	0.28

1-Way Anova

	SS	df	MS	F	p
Between	1.12	6	0.186	0.413	0.868
Within	35.50	78	0.449		
Total	36.62	84			

SI Figure 108. Correlation analysis of CCK2R staining intensity in lung cancer versus primary tumor type. IHC was performed on lung cancer tissue sections using a monoclonal antibody raised against CCK2R. The staining intensity was graded on a scale of 0 to 3 and plotted (error bars represent standard error of the mean). A 1-way ANOVA was used to determine if there were any significant differences between groups.

Spearman Analysis		
	ρ (rho) =	-
	t =	-
	p-value =	-

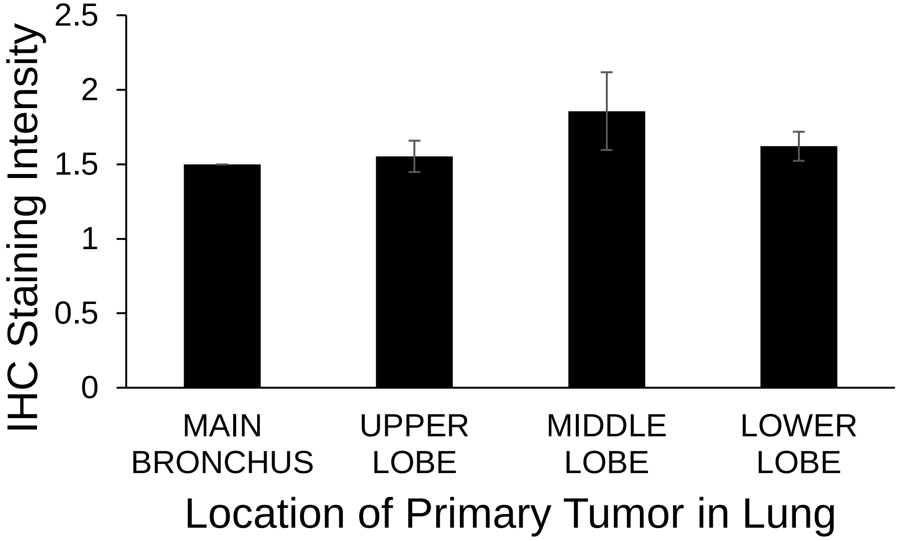

	n	Average	St. Dev	St. Error
MAIN BRONCHUS	2	1.50	-	-
UPPER LOBE	47	1.55	0.72	0.10
MIDDLE LOBE	7	1.86	0.69	0.26
LOWER LOBE	37	1.62	0.59	0.10

1-Way Anova

	SS	df	MS	F	p
Between	0.61	2	0.303	0.679	0.510
Within	39.24	88	0.446		
Total	39.84	90			

SI Figure 109. Correlation analysis of CCK2R staining intensity in lung cancer versus location of primary tumor. IHC was performed on lung cancer tissue sections using a monoclonal antibody raised against CCK2R. The staining intensity was graded on a scale of 0 to 3 and plotted (error bars represent standard error of the mean). A 1-way ANOVA was used to determine if there were any significant differences between groups.

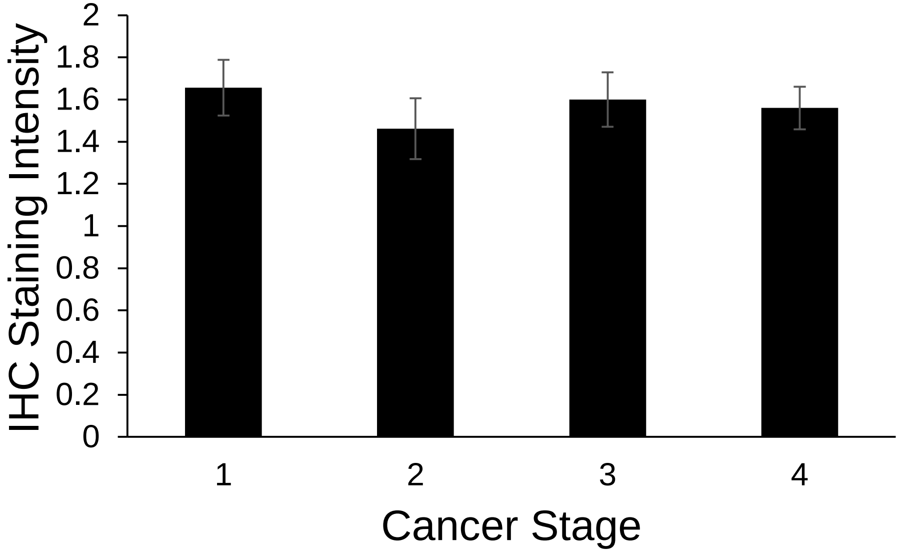

Spearman Analysis		
	ρ (rho) =	-0.0802
	t =	-0.7759
	p-value =	0.4397

	n	Average	St. Dev	St. Error
1	32	1.66	0.75	0.13
2	13	1.46	0.52	0.14
3	25	1.60	0.65	0.13
4	25	1.56	0.51	0.10

1-Way Anova

	SS	df	MS	F	p
Between	0.40	3	0.134	0.329	0.805
Within	37.07	91	0.407		
Total	37.47	94			

SI Figure 110. Correlation analysis of CCK2R staining intensity in lung cancer versus cancer stage. IHC was performed on lung cancer tissue sections using a monoclonal antibody raised against CCK2R. The staining intensity was graded on a scale of 0 to 3 and plotted (error bars represent standard error of the mean). A Spearman analysis was used to determine if there was a statistically significant correlation and a 1-way ANOVA was used to determine if there were any significant differences between groups.

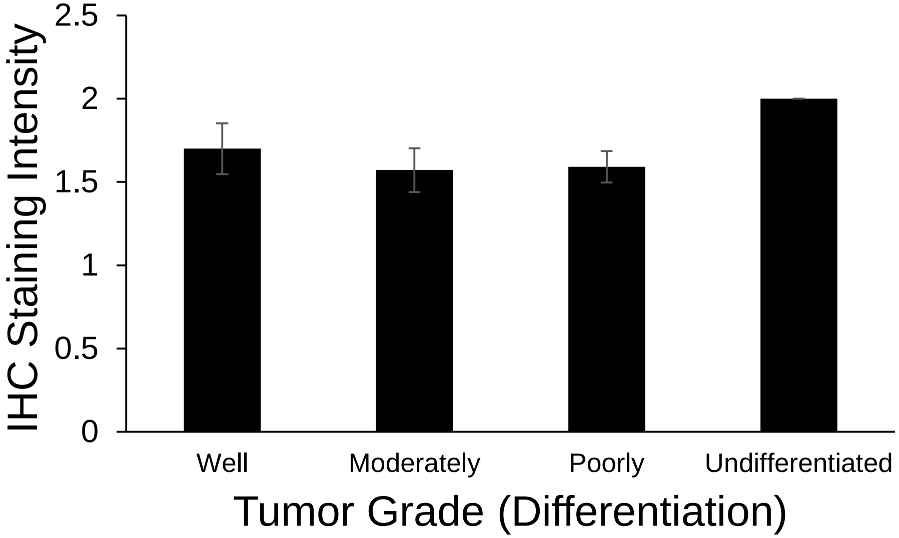

Spearman Analysis	ρ (rho) =	-0.0077
	t =	-0.0727
	p-value =	0.9422

	n	Average	St. Dev	St. Error
Well	10	1.70	0.48	0.15
Moderately	35	1.57	0.78	0.13
Poorly	44	1.59	0.62	0.09
Undifferentiated	2	2.00	-	-

1-Way Anova

	SS	df	MS	F	p
Between	0.13	2	0.067	0.146	0.865
Within	39.29	86	0.457		
Total	39.42	88			

SI Figure 111. Correlation analysis of CCK2R staining intensity in lung cancer versus tumor grade. IHC was performed on lung cancer tissue sections using a monoclonal antibody raised against CCK2R. The staining intensity was graded on a scale of 0 to 3 and plotted (error bars represent standard error of the mean). A Spearman analysis was used to determine if there was a statistically significant correlation and a 1-way ANOVA was used to determine if there were any significant differences between groups.

Spearman Analysis		
	ρ (rho) =	-0.0857
	t =	-0.7841
	p-value =	0.4352

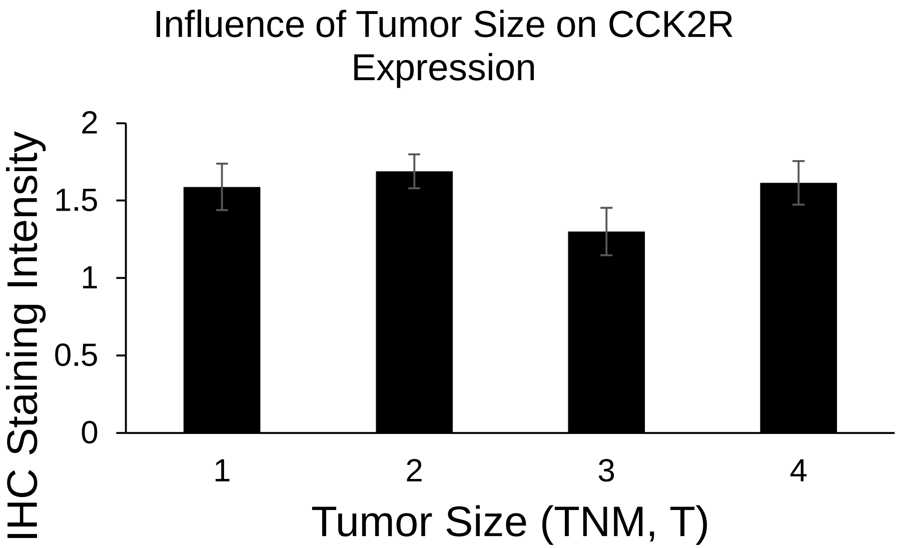

	n	Average	St. Dev	St. Error
1	17	1.59	0.62	0.15
2	45	1.69	0.73	0.11
3	10	1.30	0.48	0.15
4	13	1.62	0.51	0.14

1-Way Anova

	SS	df	MS	F	p
Between	1.26	3	0.419	0.975	0.409
Within	34.79	81	0.430		
Total	36.05	84			

SI Figure 112. Correlation analysis of CCK2R staining intensity in lung cancer versus size of primary tumor. IHC was performed on lung cancer tissue sections using a monoclonal antibody raised against CCK2R. The staining intensity was graded on a scale of 0 to 3 and plotted (error bars represent standard error of the mean). A Spearman analysis was used to determine if there was a statistically significant correlation and a 1-way ANOVA was used to determine if there were any significant differences between groups.

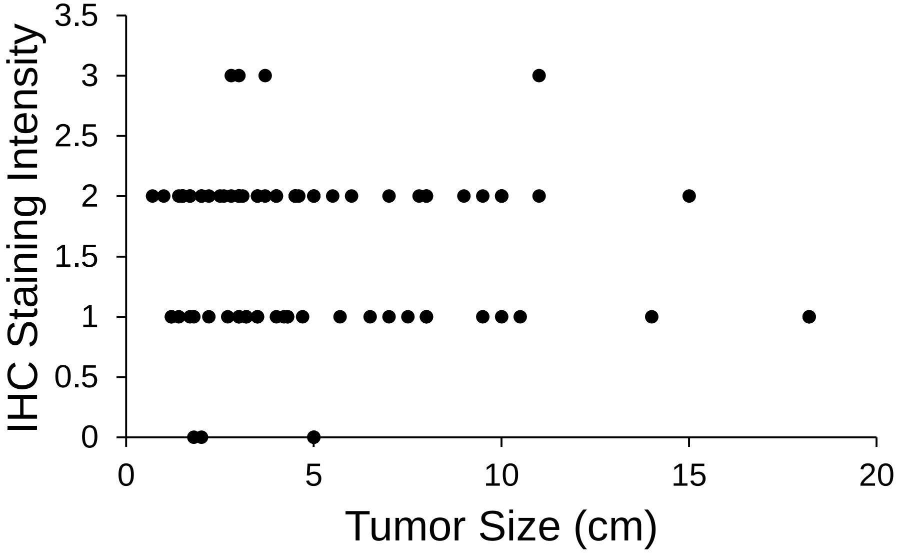

Spearman Analysis		
	ρ (rho) =	-0.0623
	t =	-0.5657
	p-value =	0.5731

	n	Average	St. Dev	St. Error
0	4	3.45	1.79	0.90
1	30	5.87	4.59	0.84
2	45	4.65	3.25	0.48
3	4	5.13	3.94	1.97

1-Way Anova

	SS	df	MS	F	p
Between	38	3	12.545	0.876	0.457
Within	1,132	79	14.328		
Total	1,170	82			

SI Figure 113. Correlation analysis of CCK2R staining intensity in lung cancer versus primary tumor size (length of longest side). IHC was performed on lung cancer tissue sections using a monoclonal antibody raised against CCK2R. The staining intensity was graded on a scale of 0 to 3 and plotted. A Spearman analysis was used to determine if there was a statistically significant correlation and a 1-way ANOVA was used to determine if there were any significant differences between groups.

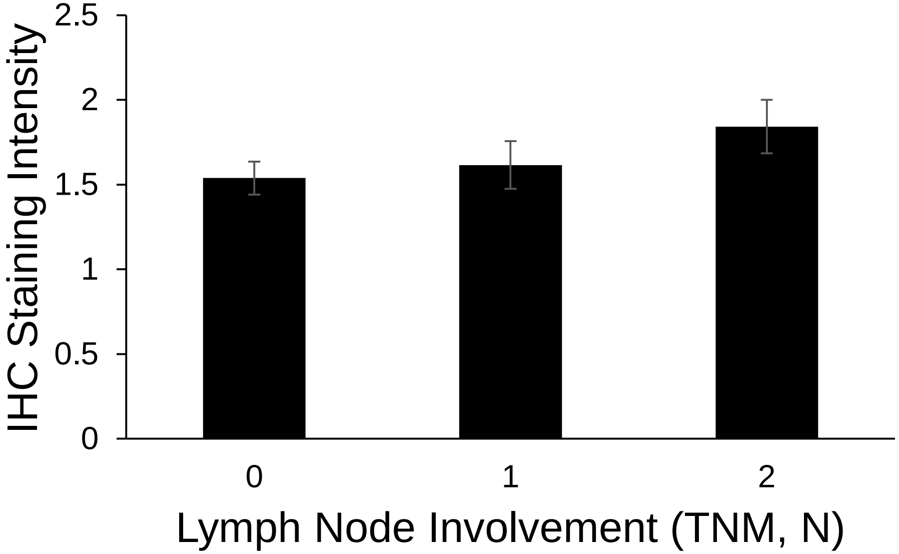

Spearman Analysis

Spearman Analysis	ρ (rho) =	0.1450
	t =	1.3274
	p-value =	0.1880

	n	Average	St. Dev	St. Error
0	52	1.54	0.70	0.10
1	13	1.62	0.51	0.14
2	19	1.84	0.69	0.16

1-Way Anova

	SS	df	MS	F	p
Between	1.25	2	0.626	1.383	0.257
Within	36.68	81	0.453		
Total	37.93	83			

SI Figure 114. Correlation analysis of CCK2R staining intensity in lung cancer versus lymph node involvement. IHC was performed on lung cancer tissue sections using a monoclonal antibody raised against CCK2R. The staining intensity was graded on a scale of 0 to 3 and plotted (error bars represent standard error of the mean). A Spearman analysis was used to determine if there was a statistically significant correlation and a 1-way ANOVA was used to determine if there were any significant differences between groups.

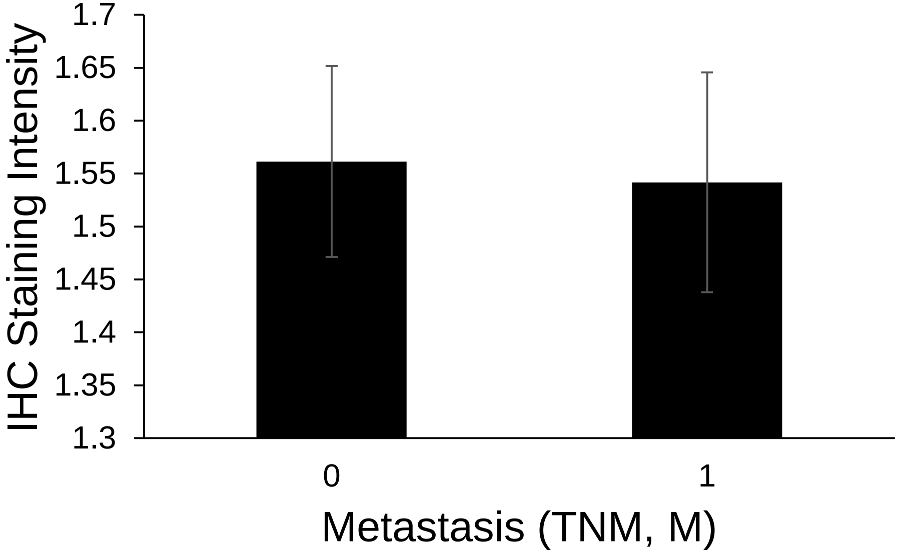

Spearman Analysis

ρ (rho) =	-0.0663
t =	-0.5908
p-value =	0.5563

	n	Average	St. Dev	St. Error
0	57	1.56	0.68	0.09
1	24	1.54	0.51	0.10

t-test

p-value = 0.899

SI Figure 115. Correlation analysis of CCK2R staining intensity in lung cancer versus presence of metastases. IHC was performed on lung cancer tissue sections using a monoclonal antibody raised against CCK2R. The staining intensity was graded on a scale of 0 to 3 and plotted (error bars represent standard error of the mean). A Spearman analysis was used to determine if there was a statistically significant correlation and a t-test was used to determine if there were any significant differences between groups.

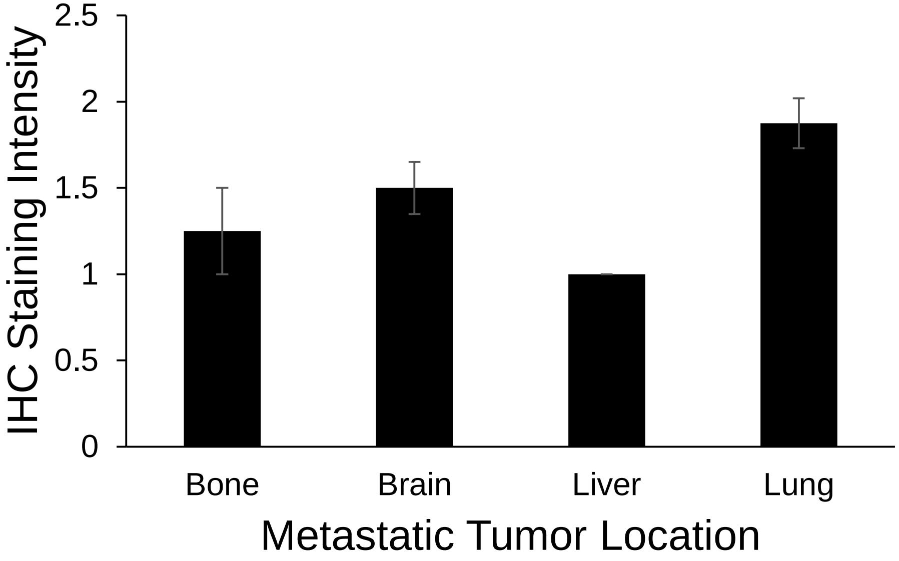

Spearman Analysis		
	ρ (rho) =	-
	t =	-
	p-value =	-

	n	Average	St. Dev	St. Error
Bone	4	1.25	0.50	0.25
Brain	12	1.50	0.52	0.15
Liver	3	1.00	0.00	0.00
Lung	6	1.88	0.35	0.14

1-Way Anova

	SS	df	MS	F	p
Between	1.86	3	0.621	3.009	0.053
Within	4.34	21	0.207		
Total	6.20	24			

SI Figure 116. Correlation analysis of CCK2R staining intensity in lung cancer versus metastatic tumor location. IHC was performed on lung cancer tissue sections using a monoclonal antibody raised against CCK2R. The staining intensity was graded on a scale of 0 to 3 and plotted (error bars represent standard error of the mean). A 1-way ANOVA was used to determine if there were any significant differences between groups.

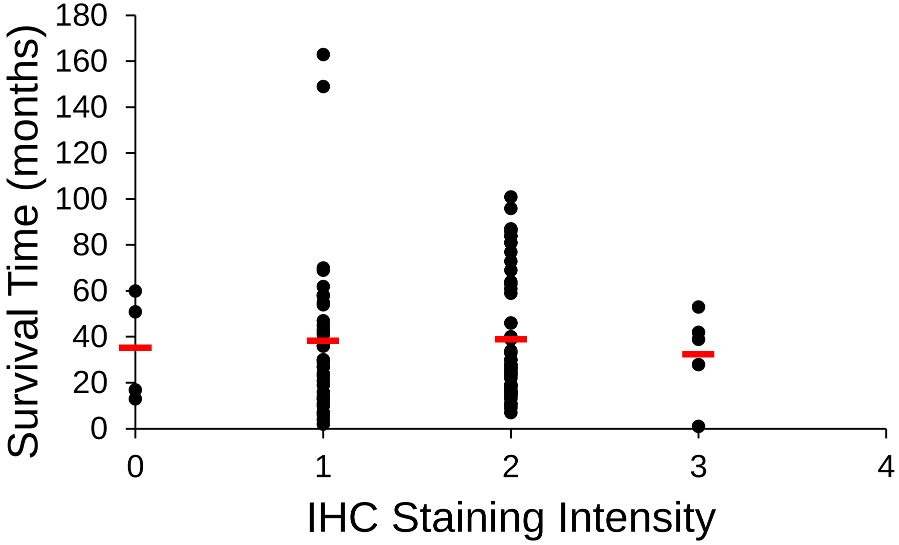

Spearman Analysis

Spearman Analysis	ρ (rho) =	0.0430
	t =	0.4302
	p-value =	0.6679

	n	Average	St. Dev	St. Error
0	4	35.25	23.73	11.86
1	38	38.34	34.16	5.54
2	55	39.11	27.51	3.71
3	5	32.60	19.78	8.85

1-Way Anova

	SS	df	MS	F	p
Between	236	3	79	0.088	0.966
Within	87,297	98	891		
Total	87,533	101			

SI Figure 117. Correlation analysis of CCK2R staining intensity in lung cancer versus survival time after diagnosis. IHC was performed on lung cancer tissue sections using a monoclonal antibody raised against CCK2R. The staining intensity was graded on a scale of 0 to 3 and plotted (red bars represent population mean). A Spearman analysis was used to determine if there was a statistically significant correlation and a 1-way ANOVA was used to determine if there were any significant differences between groups.

Spearman Analysis		
	ρ (rho) =	0.0763
	t =	0.7609
	p-value =	0.4485

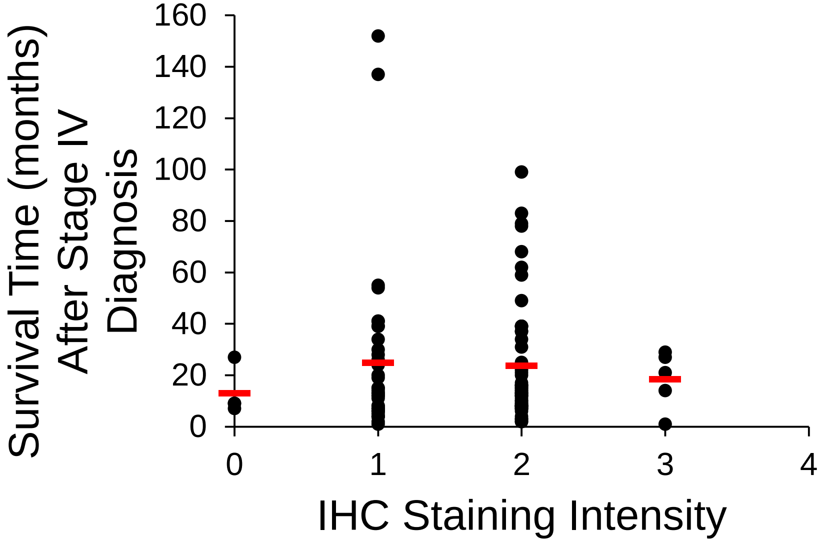

	n	Average	St. Dev	St. Error
0	4	13.00	9.38	4.69
1	38	24.84	32.09	5.21
2	54	23.70	23.33	3.18
3	5	18.40	11.35	5.08

1-Way Anova

	SS	df	MS	F	p
Between	641	3	214	0.306	0.821
Within	67,728	97	698		
Total	68,369	100			

SI Figure 118. Correlation analysis of CCK2R staining intensity in lung cancer versus survival time after stage IV diagnosis. IHC was performed on lung cancer tissue sections using a monoclonal antibody raised against CCK2R. The staining intensity was graded on a scale of 0 to 3 and plotted (red bars represent population mean). A Spearman analysis was used to determine if there was a statistically significant correlation and a 1-way ANOVA was used to determine if there were any significant differences between groups.

Lung Cancer
Coverage Score Correlations

Lung Cancer

	n	Average	St. Dev	St. Error
Coverage Score	102	2.86	0.62	0.06

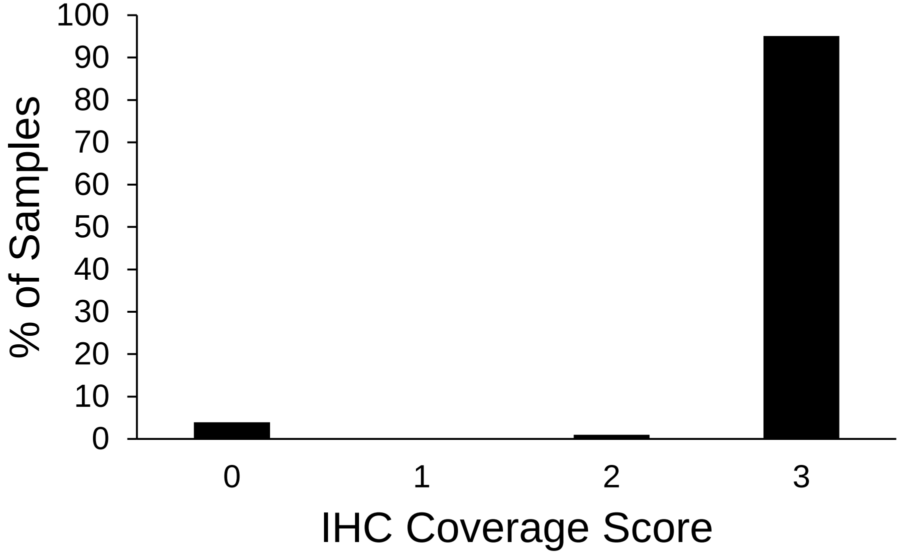

	Coverage Score			
	0	1	2	3
n	4	0	1	97
%	3.92	0.00	0.98	95.10

Normal Lung

	n	Average	St. Dev	St. Error
Coverage Score	8	0.38	1.06	0.38

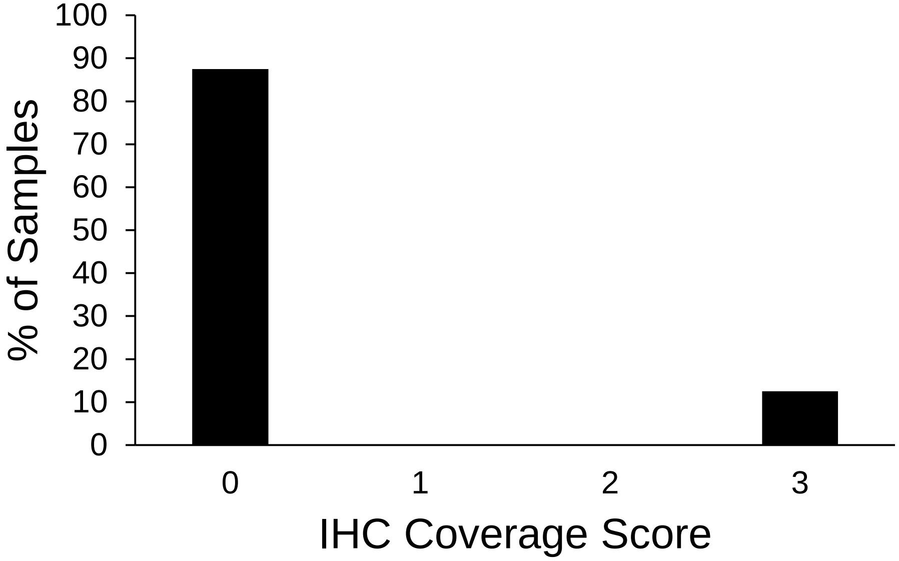

	Coverage Score			
	0	1	2	3
n	7	0	0	1
%	87.50	0.00	0.00	12.50

SI Figure 119. CCK2R Coverage Score for cancer and normal tissue from the lung. IHC was performed on tissue sections using a monoclonal antibody raised against CCK2R. The area stained (coverage) was graded on a scale of 0 to 3 and plotted.

Lung Cancer - Coverage Score

	Sex	Age at Diagnosis	Primary Tumor Type	Primary Tumor Site	Stage	Grade	Tumor Size (TNM, T)	Tumor Size (longest dimension)	Lymph Node Involvement (TNM, N)	Metastatic (TNM, M)	Metastatic Site	Survival after Diagnosis	Survival after Stage IV Diagnosis
**Spearman Correlation**	N.A.	No 0.734	N.A.	N.A.	**Yes 0.014**	No 0.341	No 0.348	No 0.8542	No 0.080	No 0.330	N.A.	No 0.855	No 0.300
**ANOVA/t-test**	No 0.130	No 0.473	No 0.136	No 0.531	No 0.067	No 0.226	No 0.625	No 0.391	No 0.230	No 0.215	No 1.000	No 0.826	No 0.4123

SI Figure 120. Coverage score correlation summary of CCK2R in lung cancer. IHC was performed on lung tumor tissue sections using a monoclonal antibody raised against CCK2R. The coverage score was compared against available patient data. If appropriate, a spearman analysis was used to determine if any significant correlation exists while a 1-way ANOVA or t-test was used to determine if a significant difference exists between groups. N.A. – not applicable (this statistical test was not applicable to this data set). Whether the test was statistically significant and the p-value is listed. N.D. – not determined (this statistical test could not be performed, generally due to a lack of the number of samples within a group or all data was in a single group).

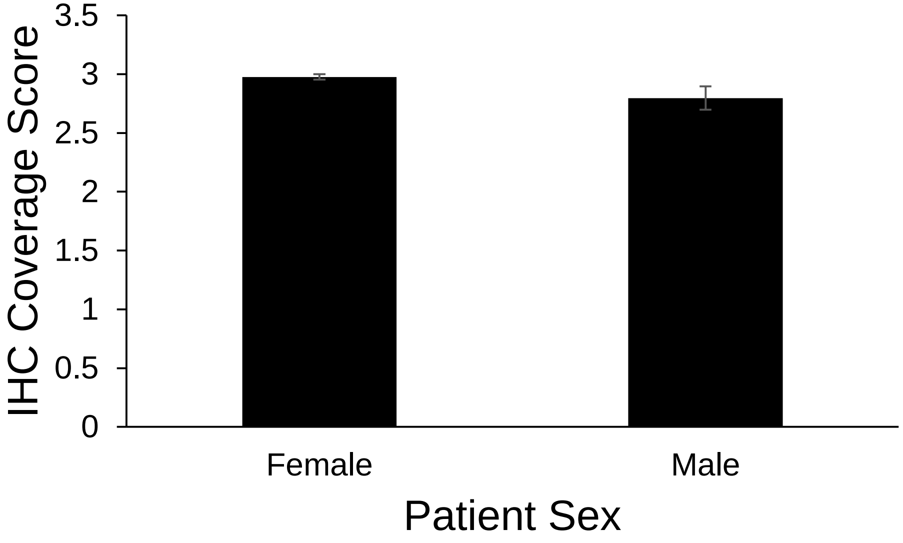

Spearman Analysis

ρ (rho) =	-
t =	-
p-value =	-

	n	Average	St. Dev	St. Error
Female	43	2.98	0.15	0.02
Male	59	2.80	0.76	0.10

t-test

p-value = 0.1295

SI Figure 121. Correlation analysis of CCK2R coverage score in lung cancer versus patient sex. IHC was performed on lung cancer tissue sections using a monoclonal antibody raised against CCK2R. The coverage score was determined by grading the area stained on a scale of 0 to 3 and plotted (error bars represent standard error of the mean). A t-test was used to determine if there were any significant differences between groups.

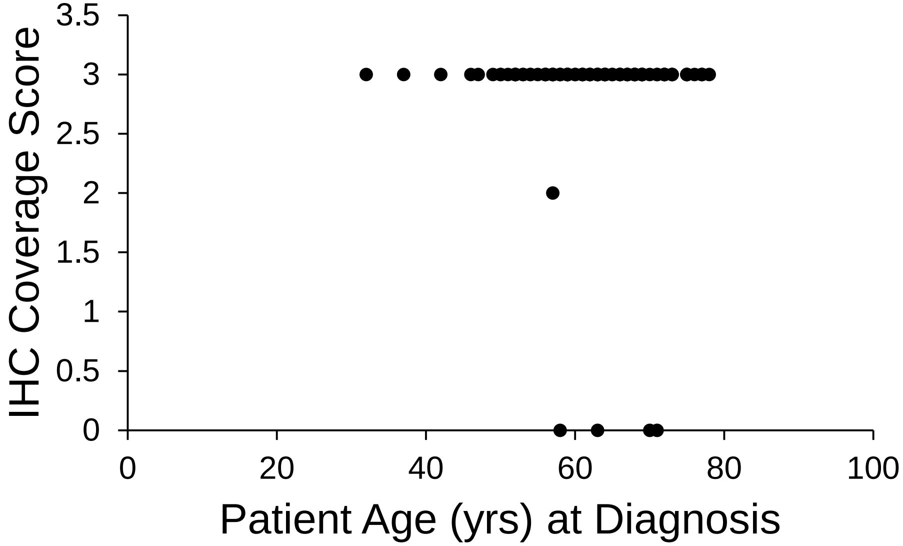

Spearman Analysis

ρ (rho) =	-0.0338	
t =	-0.3402	
p-value =	0.7344	

	n	Average	St. Dev	St. Error
0	4	65.50	6.14	3.07
1	0	-	-	-
2	2	57.00	-	-
3	97	62.24	8.94	0.91

t-test

p-value = 0.4725

SI Figure 122. Correlation analysis of CCK2R coverage score in lung cancer versus patient age at diagnosis. IHC was performed on lung cancer tissue sections using a monoclonal antibody raised against CCK2R. The coverage score was determined by grading the area stained on a scale of 0 to 3 and plotted. A Spearman analysis was used to determine if there was a statistically significant correlation and a t-test was used to determine if there were any significant differences between groups.

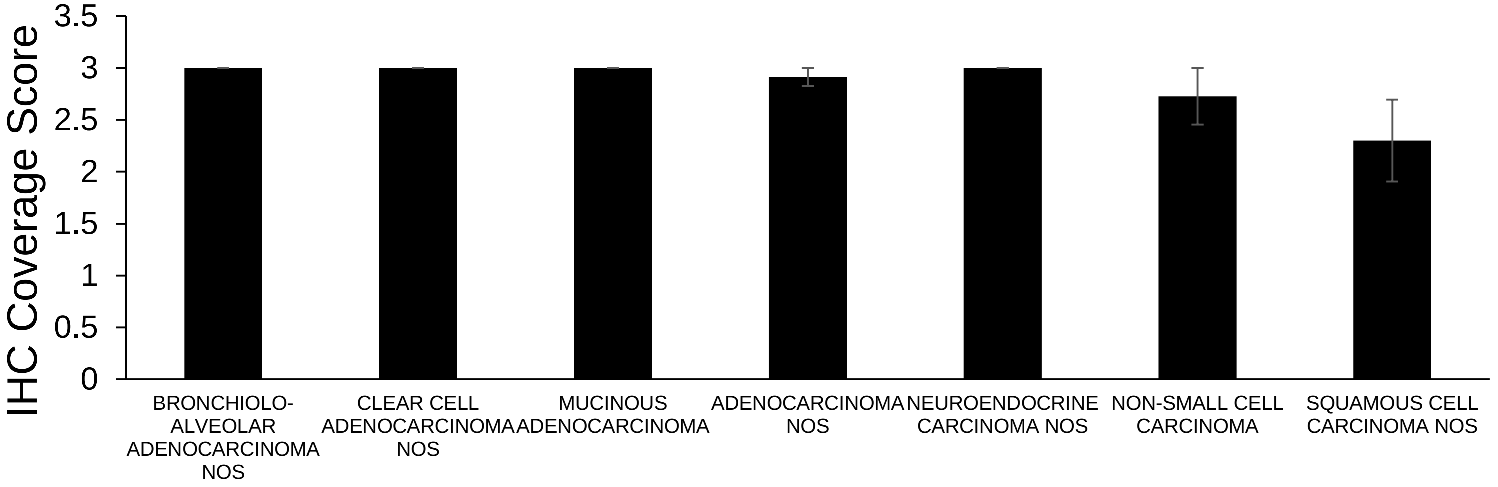

Spearman Analysis

ρ (rho) =	-
t =	-
p-value =	-

	n	Average	St. Dev	St. Error
BRONCHIOLO- ALVEOLAR ADENOCARCINOMA NOS	15	3.00	0.00	0.00
CLEAR CELL ADENOCARCINOMA NOS	3	3.00	0.00	0.00
MUCINOUS ADENOCARCINOMA	7	3.00	0.00	0.00
ADENOCARCINOMA NOS	34	2.91	0.51	0.09
NEUROENDOCRINE CARCINOMA NOS	5	3.00	0.00	0.00
NON-SMALL CELL CARCINOMA	11	2.73	0.90	0.27
SQUAMOUS CELL CARCINOMA NOS	10	2.30	1.25	0.40

1-Way Anova

	SS	df	MS	F	p
Between	3.98	6	0.663	1.683	0.136
Within	30.75	78	0.394		
Total	34.73	84			

SI Figure 123. Correlation analysis of CCK2R coverage score in lung cancer versus primary tumor type. IHC was performed on lung cancer tissue sections using a monoclonal antibody raised against CCK2R. The coverage score was determined by grading the area stained on a scale of 0 to 3 and plotted (error bars represent standard error of the mean). A 1-way ANOVA was used to determine if there were any significant differences between groups.

Spearman Analysis		
	ρ (rho) =	-
	t =	-
	p-value =	-

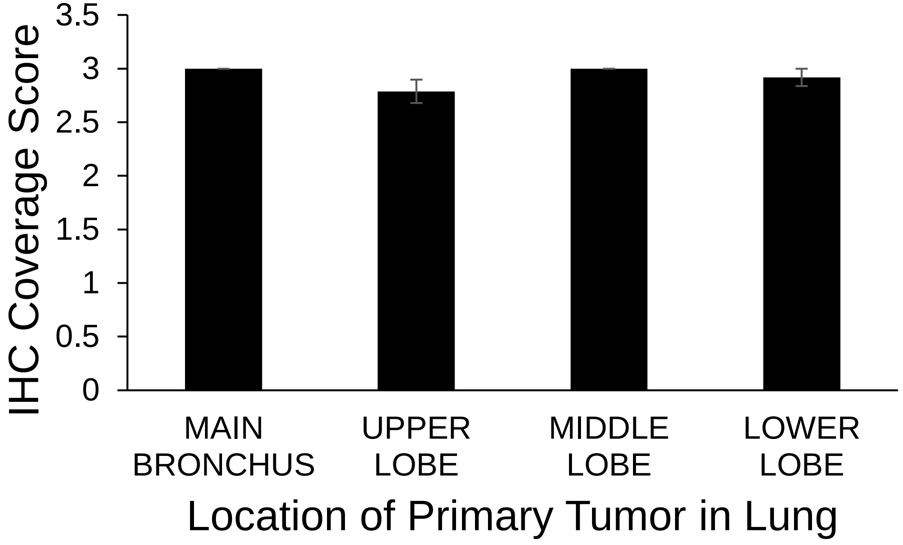

	n	Average	St. Dev	St. Error
MAIN BRONCHUS	2	3.00	-	-
UPPER LOBE	47	2.79	0.75	0.11
MIDDLE LOBE	7	3.00	0.00	0.00
LOWER LOBE	37	2.92	0.49	0.08

1-Way Anova

	SS	df	MS	F	p
Between	0.50	2	0.250	0.638	0.531
Within	34.52	88	0.392		
Total	35.02	90			

SI Figure 124. Correlation analysis of CCK2R coverage score in lung cancer versus location of primary tumor. IHC was performed on lung cancer tissue sections using a monoclonal antibody raised against CCK2R. The coverage score was determined by grading the area stained on a scale of 0 to 3 and plotted (error bars represent standard error of the mean). A 1-way ANOVA was used to determine if there were any significant differences between groups.

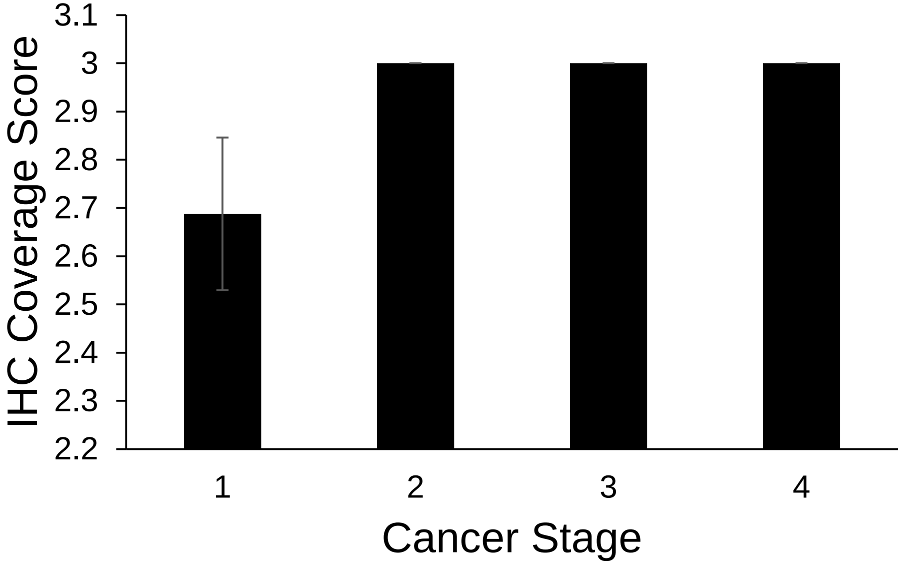

Spearman Analysis

ρ (rho) =	0.2506
t =	2.4969
p-value =	**0.0143**

	n	Average	St. Dev	St. Error
1	32	2.69	0.90	0.16
2	13	3.00	0.00	0.00
3	24	3.00	0.00	0.00
4	26	3.00	0.00	0.00

1-Way Anova

	SS	df	MS	F	p
Between	2.04	3	0.680	2.464	0.067
Within	25.11	91	0.276		
Total	27.15	94			

SI Figure 125. Correlation analysis of CCK2R coverage score in lung cancer versus cancer stage. IHC was performed on lung cancer tissue sections using a monoclonal antibody raised against CCK2R. The coverage score was determined by grading the area stained on a scale of 0 to 3 and plotted (error bars represent standard error of the mean). A Spearman analysis was used to determine if there was a statistically significant correlation and a 1-way ANOVA was used to determine if there were any significant differences between groups.

Spearman Analysis	
ρ (rho) =	0.1009
t =	0.9571
p-value =	0.3411

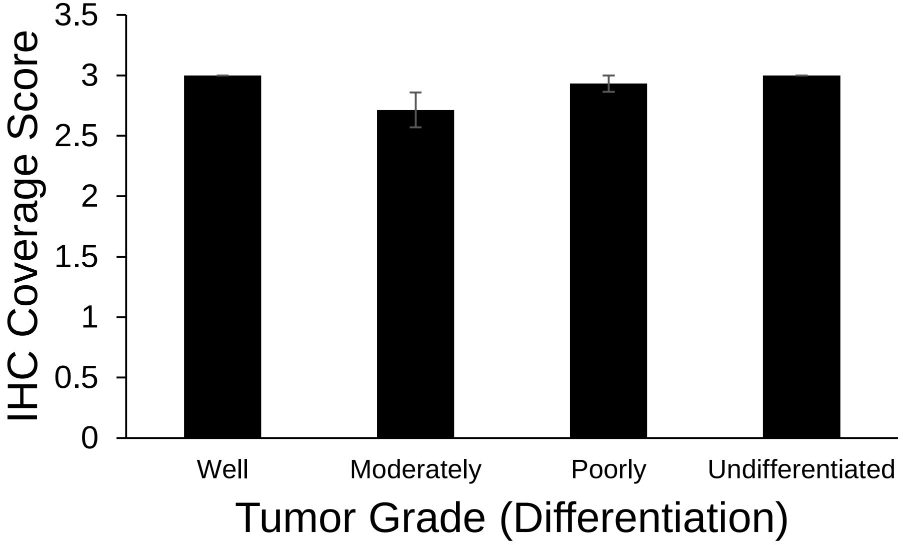

	n	Average	St. Dev	St. Error
Well	10	3.00	0.00	0.00
Moderately	35	2.71	0.86	0.15
Poorly	44	2.93	0.45	0.07
Undifferentiated	2	3.00	-	-

1-Way Anova

	SS	df	MS	F	p
Between	1.19	2	0.596	1.515	0.226
Within	33.85	86	0.394		
Total	35.05	88			

SI Figure 126. Correlation analysis of CCK2R coverage score in lung cancer versus tumor grade. IHC was performed on lung cancer tissue sections using a monoclonal antibody raised against CCK2R. The coverage score was determined by grading the area stained on a scale of 0 to 3 and plotted (error bars represent standard error of the mean). A Spearman analysis was used to determine if there was a statistically significant correlation and a 1-way ANOVA was used to determine if there were any significant differences between groups.

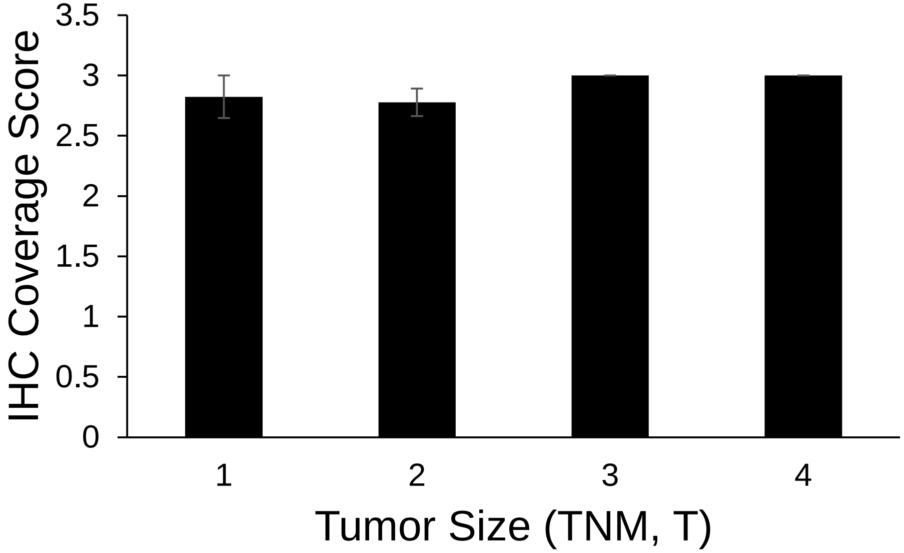

Spearman Analysis		
	ρ (rho) =	0.1032
	t =	0.9448
	p-value =	0.3475

	n	Average	St. Dev	St. Error
1	17	2.82	0.73	0.18
2	45	2.78	0.77	0.11
3	10	3.00	0.00	0.00
4	13	3.00	0.00	0.00

1-Way Anova

	SS	df	MS	F	p
Between	0.75	3	0.251	0.587	0.625
Within	34.61	81	0.427		
Total	35.37	84			

SI Figure 127. Correlation analysis of CCK2R coverage score in lung cancer versus size of primary tumor. IHC was performed on lung cancer tissue sections using a monoclonal antibody raised against CCK2R. The coverage score was determined by grading the area stained on a scale of 0 to 3 and plotted (error bars represent standard error of the mean). A Spearman analysis was used to determine if there was a statistically significant correlation and a 1-way ANOVA was used to determine if there were any significant differences between groups.

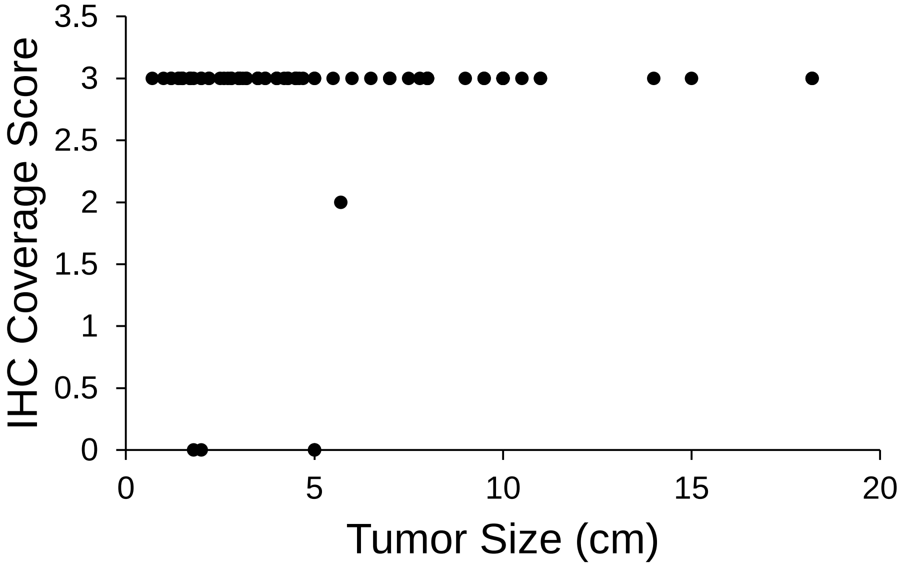

Spearman Analysis

ρ (rho) = 0.0204
t = 0.1844
p-value = 0.8542

	n	Average	St. Dev	St. Error
0	4	3.45	1.79	0.90
1	0	-	-	-
2	1	5.70	-	-
3	78	5.13	3.86	0.44

t-test

p-value = 0.3909

SI Figure 128. Correlation analysis of CCK2R coverage score in lung cancer versus primary tumor size (length of longest side). IHC was performed on lung cancer tissue sections using a monoclonal antibody raised against CCK2R. The coverage score was determined by grading the area stained on a scale of 0 to 3 and plotted. A Spearman analysis was used to determine if there was a statistically significant correlation and a 1-way ANOVA was used to determine if there were any significant differences between groups.

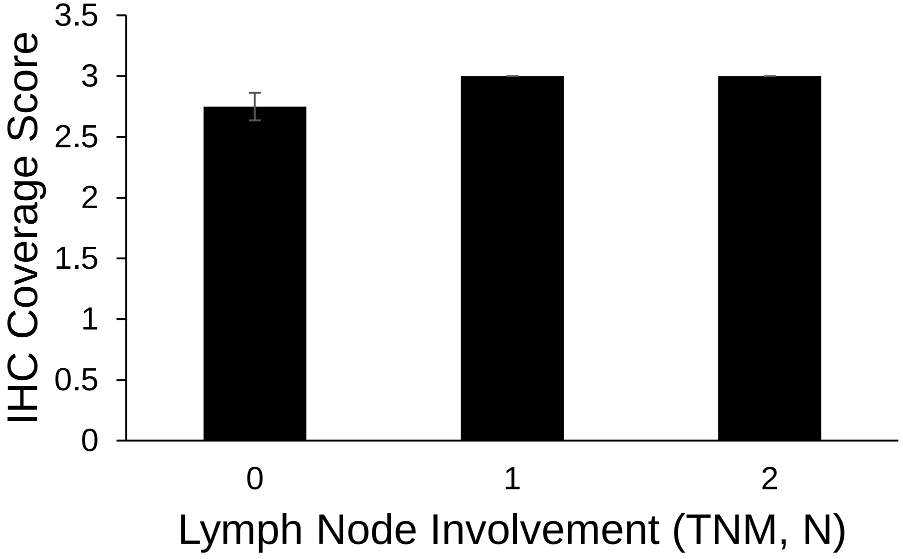

Spearman Analysis

ρ (rho) =	0.1919
t =	1.7710
p-value =	0.0802

	n	Average	St. Dev	St. Error
0	52	2.75	0.81	0.11
1	13	3.00	0.00	0.00
2	19	3.00	0.00	0.00

1-Way Anova

	SS	df	MS	F	p
Between	1.24	2	0.619	1.499	0.230
Within	33.46	81	0.413		
Total	34.70	83			

SI Figure 129. Correlation analysis of CCK2R coverage score in lung cancer versus lymph node involvement. IHC was performed on lung cancer tissue sections using a monoclonal antibody raised against CCK2R. The coverage score was determined by grading the area stained on a scale of 0 to 3 and plotted (error bars represent standard error of the mean). A Spearman analysis was used to determine if there was a statistically significant correlation and a 1-way ANOVA was used to determine if there were any significant differences between groups.

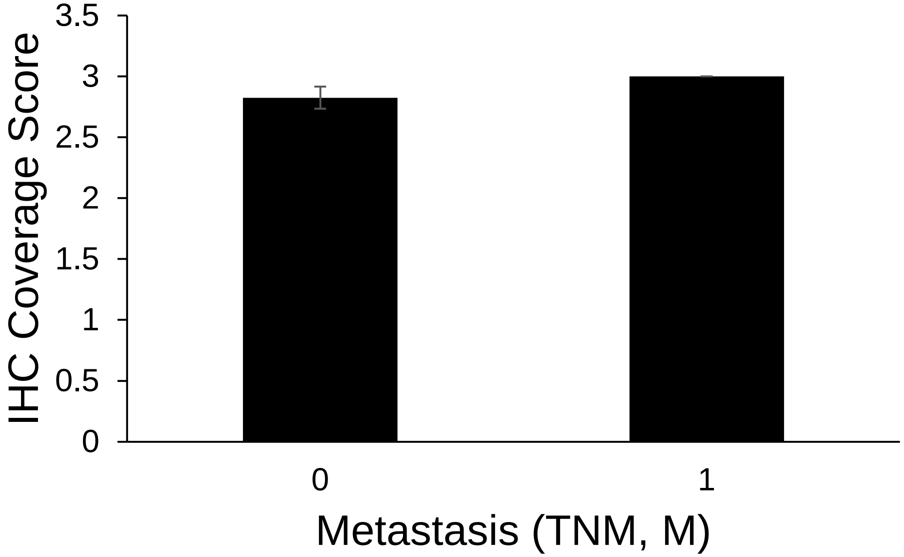

Spearman Analysis

ρ (rho) = 0.1096
t = 0.9797
p-value = 0.3301

	n	Average	St. Dev	St. Error
0	57	2.82	0.68	0.09
1	24	3.00	0.00	0.00

t-test

p-value = 0.215

SI Figure 130. Correlation analysis of CCK2R coverage score in lung cancer versus presence of metastases. IHC was performed on lung cancer tissue sections using a monoclonal antibody raised against CCK2R. The coverage score was determined by grading the area stained on a scale of 0 to 3 and plotted (error bars represent standard error of the mean). A Spearman analysis was used to determine if there was a statistically significant correlation and a t-test was used to determine if there were any significant differences between groups.

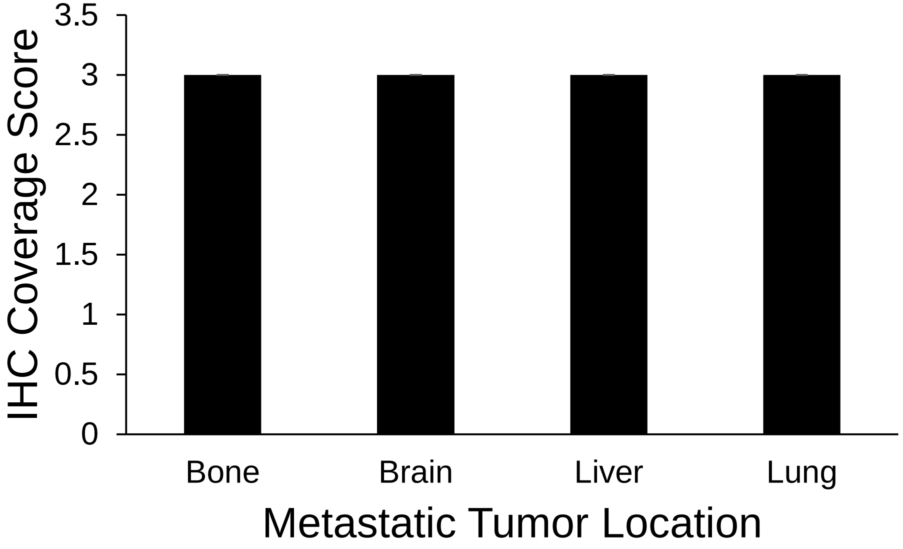

Spearman Analysis	
ρ (rho) =	-
t =	-
p-value =	-

	n	Average	St. Dev	St. Error
Bone	4	3.00	0.00	0.00
Brain	12	3.00	0.00	0.00
Liver	3	3.00	0.00	0.00
Lung	6	3.00	0.00	0.00

1-Way Anova

	SS	df	MS	F	p
Between	0.00	3	0.000	0.000	1.000
Within	0.00	21	0.000		
Total	0.00	24			

SI Figure 131. Correlation analysis of CCK2R coverage score in lung cancer versus metastatic tumor location. IHC was performed on lung cancer tissue sections using a monoclonal antibody raised against CCK2R. The coverage score was determined by grading the area stained on a scale of 0 to 3 and plotted (error bars represent standard error of the mean). A 1-way ANOVA was used to determine if there were any significant differences between groups.

Spearman Analysis

ρ (rho) =	0.0183	
t =	0.1831	
p-value =	0.8551	

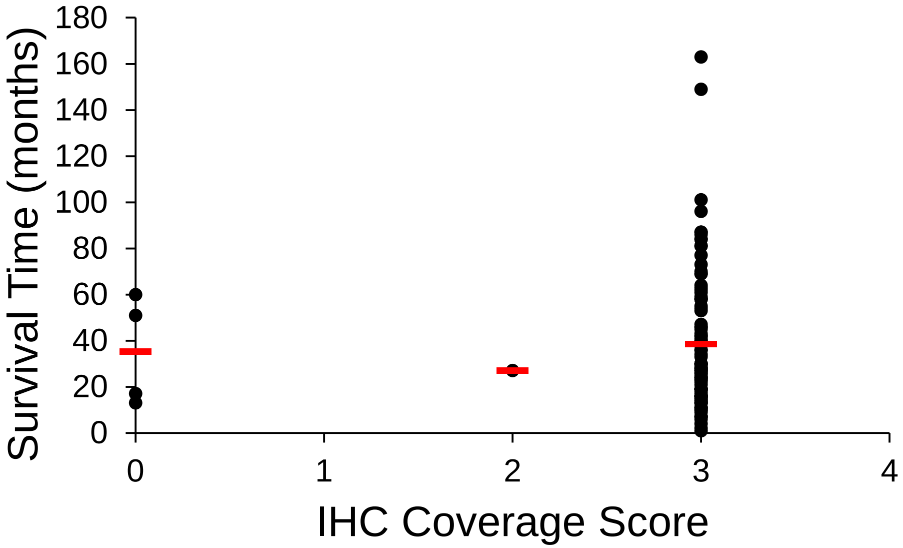

	n	Average	St. Dev	St. Error
0	4	35.25	23.73	11.86
1	0	-	-	-
2	1	27.00	-	-
3	97	38.60	29.88	3.03

t-test

p-value = 0.8256

SI Figure 132. Correlation analysis of CCK2R coverage score in lung cancer versus survival time after diagnosis. IHC was performed on lung cancer tissue sections using a monoclonal antibody raised against CCK2R. The coverage score was determined by grading the area stained on a scale of 0 to 3 and plotted (red bars represent population mean). A Spearman analysis was used to determine if there was a statistically significant correlation and a t-test was used to determine if there were any significant differences between groups.

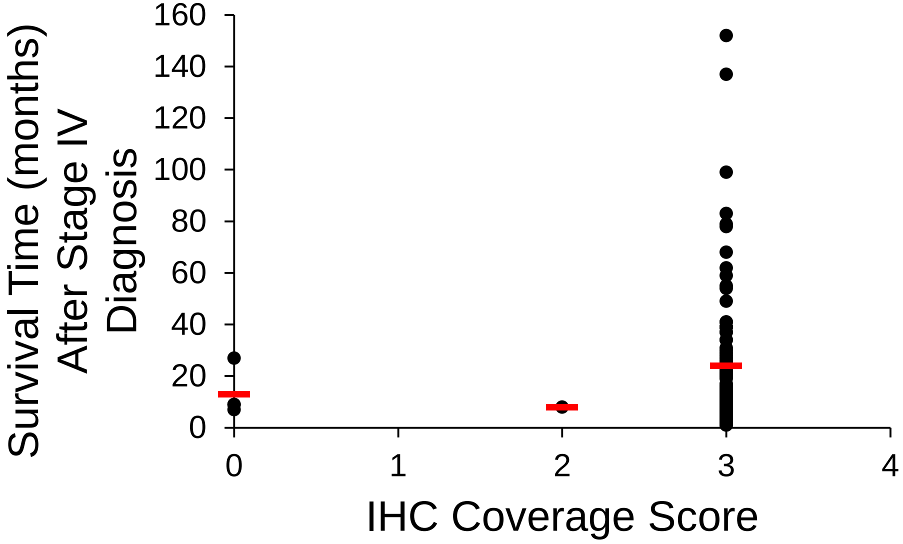

Spearman Analysis

ρ (rho) = 0.1042
t = 1.0421
p-value = 0.2999

	n	Average	St. Dev	St. Error
0	4	13.00	9.38	4.69
1	0	-	-	-
2	1	8.00	-	-
3	96	24.04	26.64	2.72

t-test

p-value = 0.4123

SI Figure 133. Correlation analysis of CCK2R coverage score in lung cancer versus survival time after stage IV diagnosis. IHC was performed on lung cancer tissue sections using a monoclonal antibody raised against CCK2R. The coverage score was determined by grading the area stained on a scale of 0 to 3 and plotted (red bars represent population mean). A Spearman analysis was used to determine if there was a statistically significant correlation and a t-test was used to determine if there were any significant differences between groups.

Lung Cancer
Total Staining Score Correlations

Lung Cancer

	n	Average	St. Dev	St. Error
Total Score	102	4.77	1.96	0.19

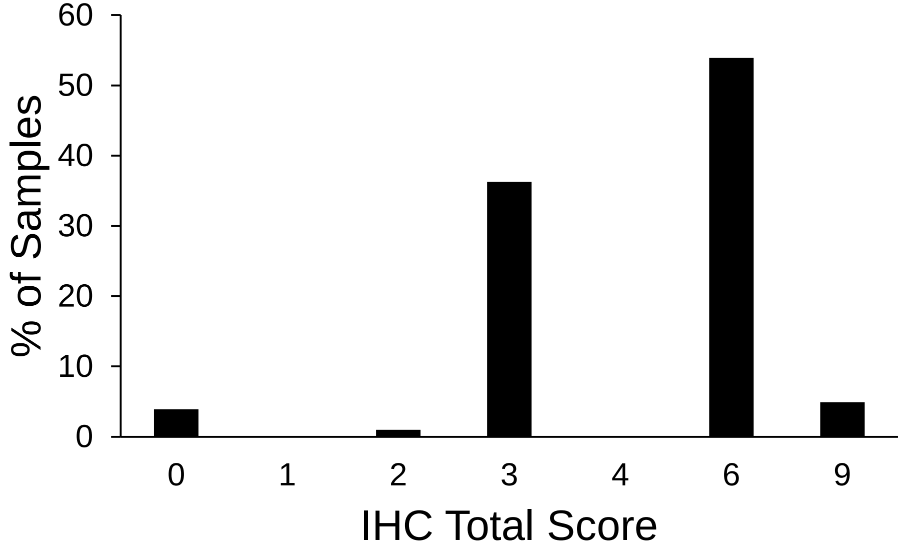

	Total Staining Score					
	0	1	2	3	4	6
n	4	0	1	37	0	55
%	3.92	0.00	0.98	36.27	0.00	53.92

Normal Lung

	n	Average	St. Dev	St. Error
Total Score	8	1.13	3.18	1.13

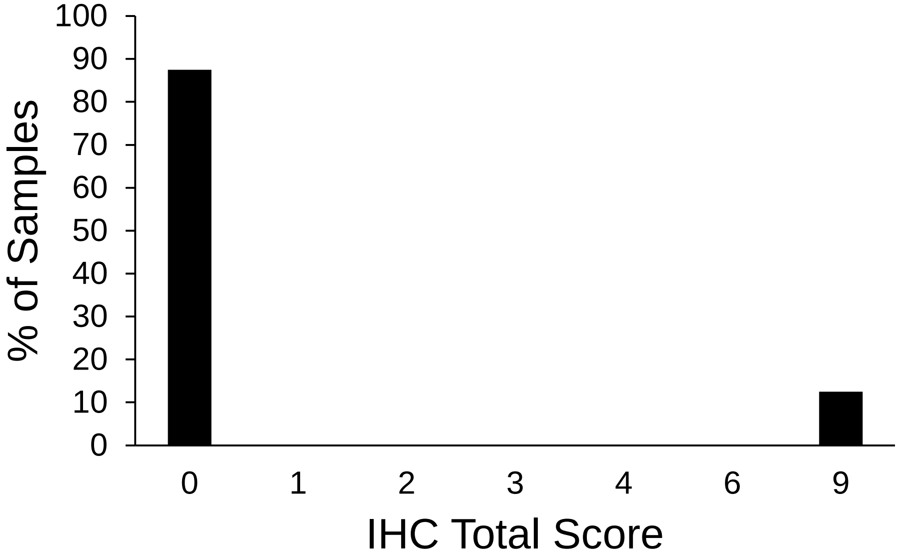

	Total Staining Score						
	0	1	2	3	4	6	9
n	7	0	0	0	0	0	1
%	87.50	0.00	0.00	0.00	0.00	0.00	12.50

SI Figure 134. CCK2R Total Staining Score for cancer and normal tissue from the lung. IHC was performed on tissue sections using a monoclonal antibody raised against CCK2R. The staining intensity and coverage score was multiplied to obtain the total staining score.

Lung Cancer - Total Staining Score

	Sex	Age at Diagnosis	Primary Tumor Type	Primary Tumor Site	Stage	Grade	Tumor Size (TNM, T)	Tumor Size (longest dimension)	Lymph Node Involvement (TNM, N)	Metastatic (TNM, M)	Metastatic Site	Survival after Diagnosis	Survival after Stage IV Diagnosis
**Spearman Correlation**	N.A.	No 0.732	N.A.	N.A.	No 0.509	No 0.990	No 0.452	No 0.546	No 0.171	No 0.558	N.A.	No 0.663	No 0.418
**ANOVA/t-test**	No 0.978	No 0.786	No 0.364	No 0.511	No 0.838	No 0.855	No 0.444	No 0.465	No 0.238	No 0.929	**Yes 0.012**	No 0.966	No 0.845

SI Figure 135. Total staining score correlation summary of CCK2R in lung cancer. IHC was performed on lung tumor tissue sections using a monoclonal antibody raised against CCK2R. The total staining score was compared against available patient data. If appropriate, a spearman analysis was used to determine if any significant correlation exists while a 1-way ANOVA or t-test was used to determine if a significant difference exists between groups. N.A. – not applicable (this statistical test was not applicable to this data set). Whether the test was statistically significant and the p-value is listed. N.D. – not determined (this statistical test could not be performed, generally due to a lack of the number of samples within a group or all data was in a single group).

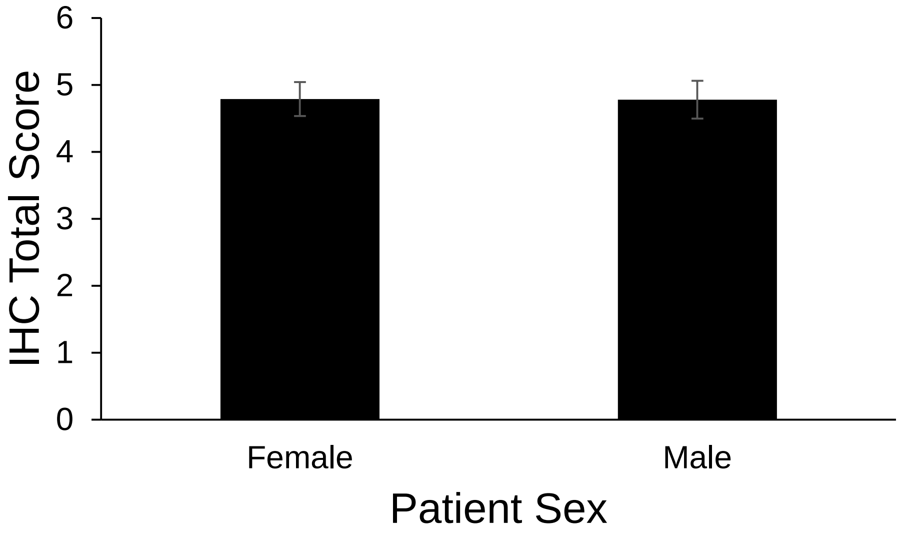

Spearman Analysis	
ρ (rho) =	-
t =	-
p-value =	-

	n	Average	St. Dev	St. Error
Female	43	4.79	1.66	0.25
Male	59	4.78	2.17	0.28

t-test

p-value = 0.9777

SI Figure 136. Correlation analysis of CCK2R total staining score in lung cancer versus patient sex. IHC was performed on lung cancer tissue sections using a monoclonal antibody raised against CCK2R. The total staining score was derived by multiplying the staining intensity with the coverage score and plotted (error bars represent standard error of the mean). A t-test was used to determine if there were any significant differences between groups.

Spearman Analysis		
	ρ (rho) =	-0.0344
	t =	-0.3440
	p-value =	0.7315

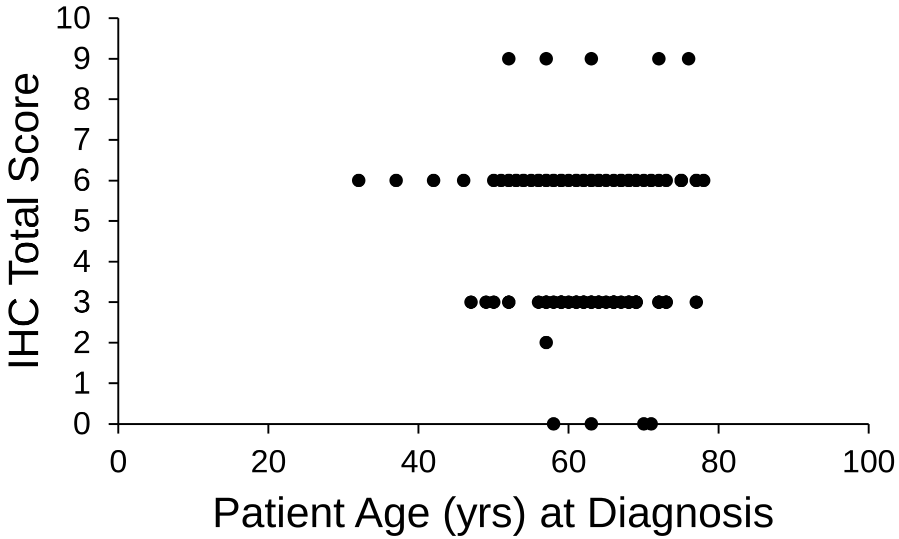

	n	Average	St. Dev	St. Error
0	4	65.50	6.14	3.07
1	0	-	-	-
2	1	57.00	-	-
3	37	62.81	7.22	1.19
4	0	-	-	-
6	55	61.69	9.96	1.34
9	5	64.00	10.02	4.48

1-Way Anova

	SS	df	MS	F	p
Between	85	3	28.4	0.355	0.786
Within	7,748	97	79.9		
Total	7,833	100			

SI Figure 137. Correlation analysis of CCK2R total staining score in lung cancer versus patient age at diagnosis. IHC was performed on lung cancer tissue sections using a monoclonal antibody raised against CCK2R. The total staining score was derived by multiplying the staining intensity with the coverage score and plotted. A Spearman analysis was used to determine if there was a statistically significant correlation and a 1-way ANOVA was used to determine if there were any significant differences between groups.

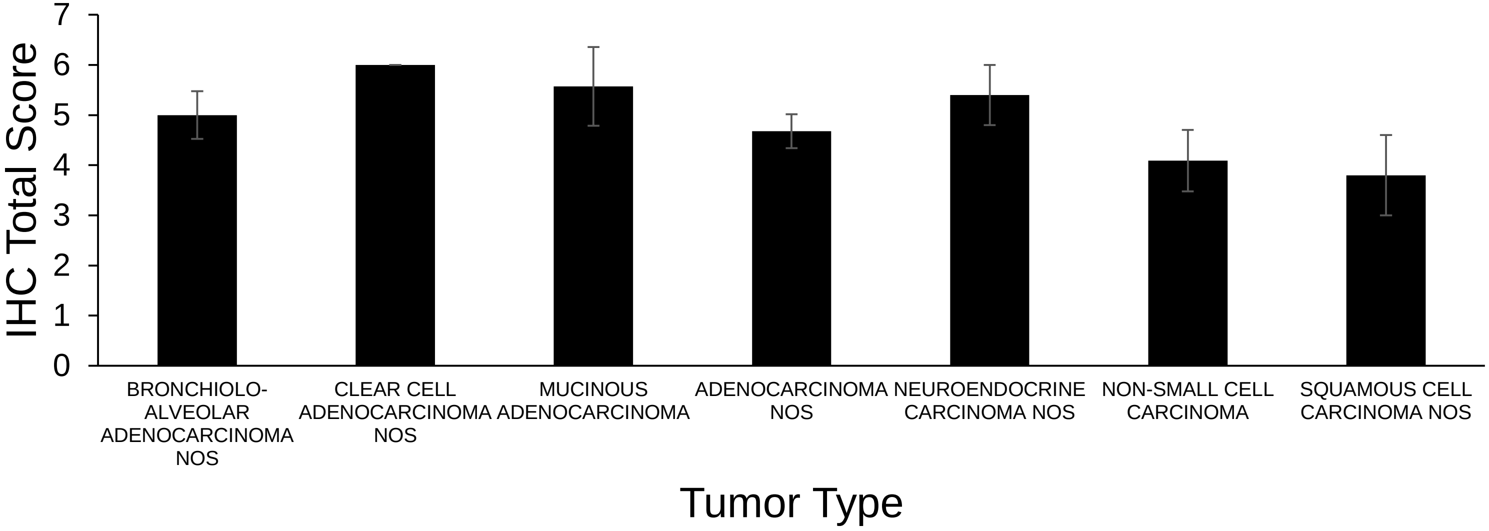

Spearman Analysis

ρ (rho) =	-
t =	-
p-value =	-

	n	Average	St. Dev	St. Error
BRONCHIOLO- ALVEOLAR ADENOCARCINOMA NOS	15	5.00	1.85	0.48
CLEAR CELL ADENOCARCINOMA NOS	3	6.00	0.00	0.00
MUCINOUS ADENOCARCINOMA	7	5.57	2.07	0.78
ADENOCARCINOMA NOS	34	4.68	1.98	0.34
NEUROENDOCRINE CARCINOMA NOS	5	5.40	1.34	0.60
NON-SMALL CELL CARCINOMA	11	4.09	2.02	0.61
SQUAMOUS CELL CARCINOMA NOS	10	3.80	2.53	0.80

1-Way Anova

	SS	df	MS	F	p
Between	26.35	6	4.391	1.110	0.364
Within	308.59	78	3.956		
Total	334.94	84			

SI Figure 138. Correlation analysis of CCK2R total staining score in lung cancer versus primary tumor type. IHC was performed on lung cancer tissue sections using a monoclonal antibody raised against CCK2R. The total staining score was derived by multiplying the staining intensity with the coverage score and plotted (error bars represent standard error of the mean). A 1-way ANOVA was used to determine if there were any significant differences between groups.

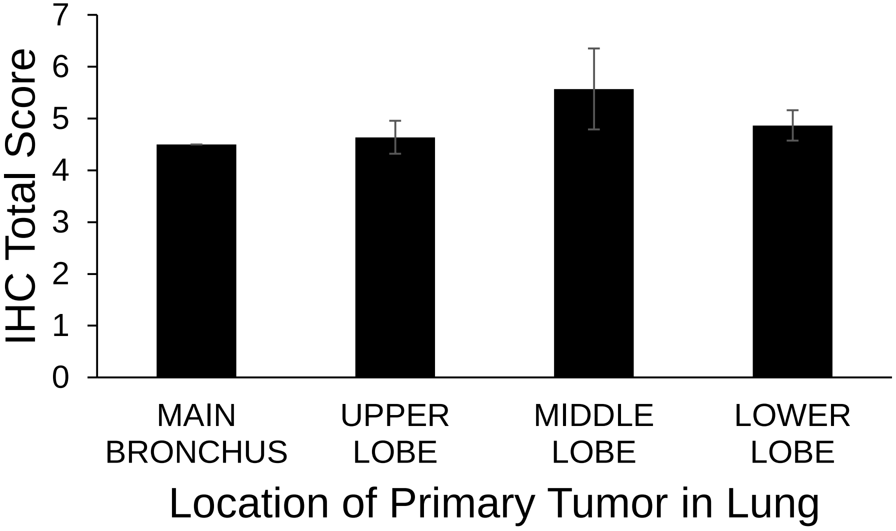

Spearman Analysis		
	ρ (rho) =	-
	t =	-
	p-value =	-

	n	Average	St. Dev	St. Error
MAIN BRONCHUS	2	4.50	-	-
UPPER LOBE	47	4.64	2.17	0.32
MIDDLE LOBE	7	5.57	2.07	0.78
LOWER LOBE	37	4.86	1.78	0.29

1-Way Anova

	SS	df	MS	F	p
Between	5.49	2	2.743	0.677	0.511
Within	356.38	88	4.050		
Total	361.87	90			

SI Figure 139. Correlation analysis of CCK2R total staining score in lung cancer versus location of primary tumor. IHC was performed on lung cancer tissue sections using a monoclonal antibody raised against CCK2R. The total staining score was derived by multiplying the staining intensity with the coverage score and plotted (error bars represent standard error of the mean). A 1-way ANOVA was used to determine if there were any significant differences between groups.

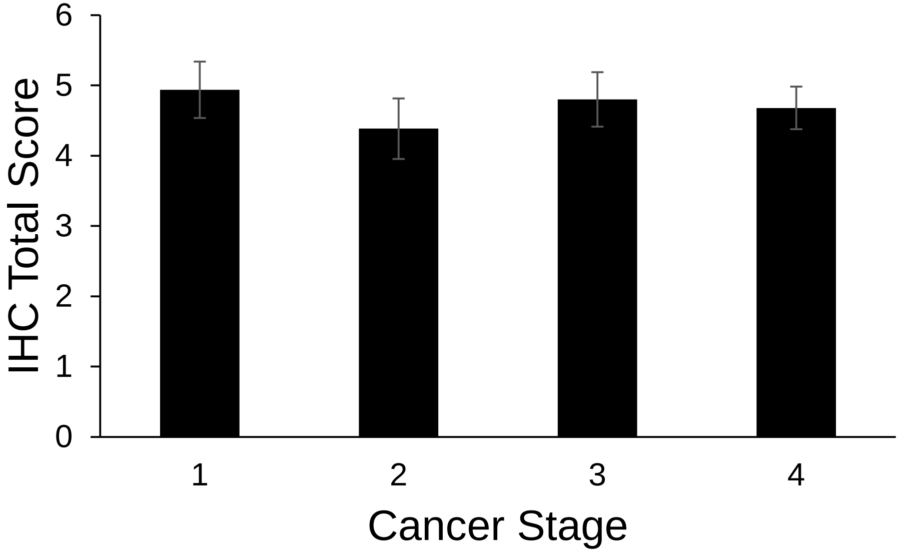

Spearman Analysis

ρ (rho) =	-0.0686
t =	-0.6631
p-value =	0.5089

	n	Average	St. Dev	St. Error
1	32	4.94	2.27	0.40
2	13	4.38	1.56	0.43
3	25	4.80	1.94	0.39
4	25	4.68	1.52	0.30

1-Way Anova

	SS	df	MS	F	p
Between	3.11	3	1.038	0.282	0.838
Within	334.72	91	3.678		
Total	337.83	94			

SI Figure 140. Correlation analysis of CCK2R total staining score in lung cancer versus cancer stage. IHC was performed on lung cancer tissue sections using a monoclonal antibody raised against CCK2R. The total staining score was derived by multiplying the staining intensity with the coverage score and plotted (error bars represent standard error of the mean). A Spearman analysis was used to determine if there was a statistically significant correlation and a 1-way ANOVA was used to determine if there were any significant differences between groups.

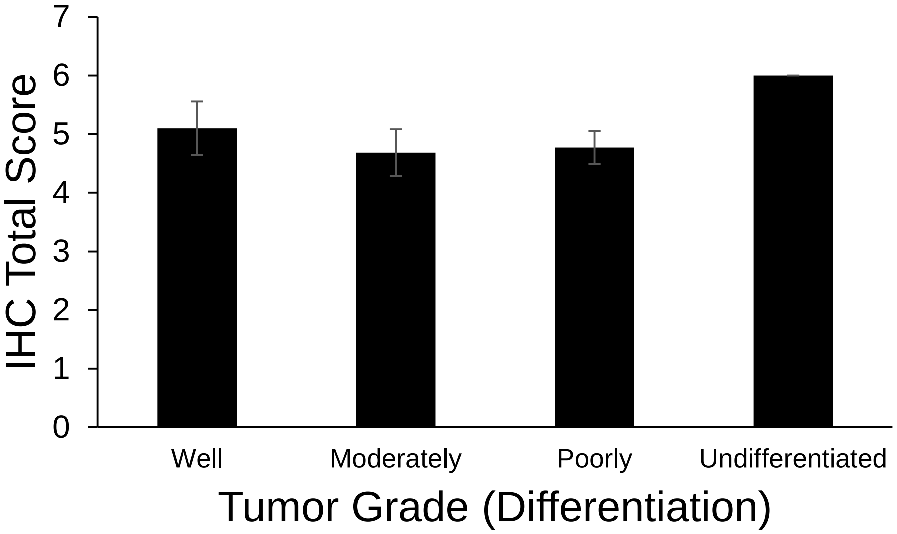

Spearman Analysis

ρ (rho) = -0.0014
t = -0.0131
p-value = 0.9896

	n	Average	St. Dev	St. Error
Well	10	5.10	1.45	0.46
Moderately	35	4.69	2.36	0.40
Poorly	44	4.77	1.87	0.28
Undifferentiated	2	6.00	0.00	0.00

1-Way Anova

	SS	df	MS	F	p
Between	1.31	2	0.655	0.157	0.855
Within	358.66	86	4.170		
Total	359.97	88			

SI Figure 141. Correlation analysis of CCK2R total staining score in lung cancer versus tumor grade. IHC was performed on lung cancer tissue sections using a monoclonal antibody raised against CCK2R. The total staining score was derived by multiplying the staining intensity with the coverage score and plotted (error bars represent standard error of the mean). A Spearman analysis was used to determine if there was a statistically significant correlation and a 1-way ANOVA was used to determine if there were any significant differences between groups.

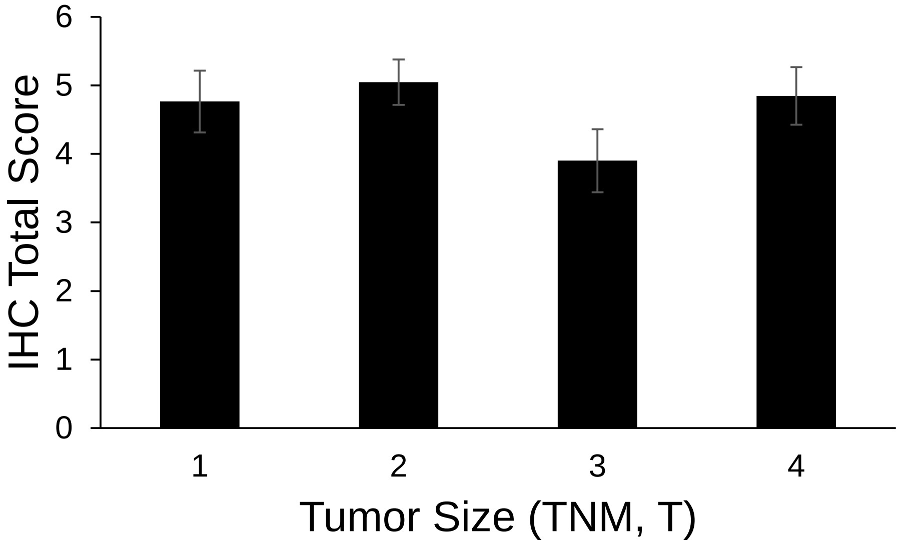

Spearman Analysis

ρ (rho) =	-0.0827
t =	-0.7563
p-value =	0.4516

	n	Average	St. Dev	St. Error
1	17	4.76	1.86	0.45
2	45	5.04	2.23	0.33
3	10	3.90	1.45	0.46
4	13	4.85	1.52	0.42

1-Way Anova

	SS	df	MS	F	p
Between	10.72	3	3.572	0.902	0.444
Within	320.81	81	3.961		
Total	331.52	84			

SI Figure 142. Correlation analysis of CCK2R total staining score in lung cancer versus size of primary tumor. IHC was performed on lung cancer tissue sections using a monoclonal antibody raised against CCK2R. The total staining score was derived by multiplying the staining intensity with the coverage score and plotted (error bars represent standard error of the mean). A Spearman analysis was used to determine if there was a statistically significant correlation and a 1-way ANOVA was used to determine if there were any significant differences between groups.

Spearman Analysis		
	ρ (rho) =	-0.0669
	t =	-0.6069
	p-value =	0.5456

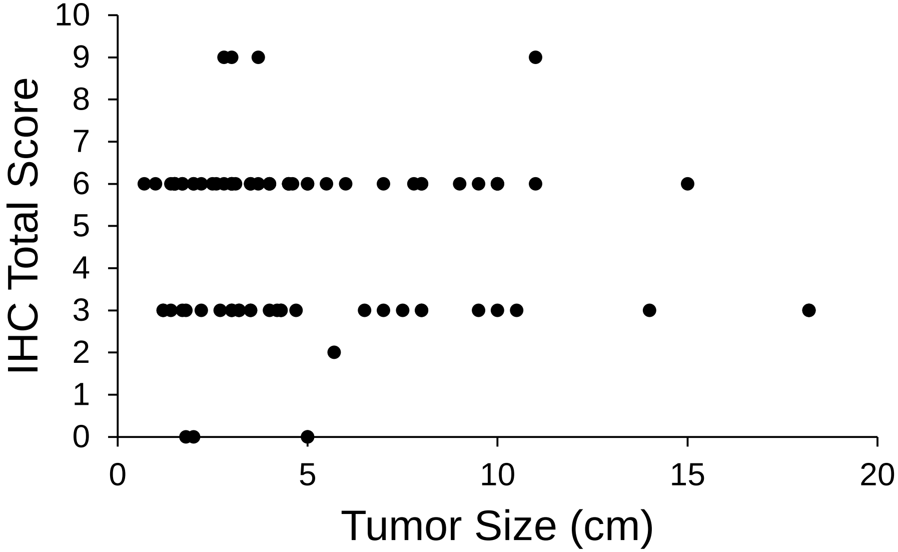

	n	Average	St. Dev	St. Error
0	4	3.45	1.79	0.90
1	0	-	-	-
2	1	5.70	-	-
3	29	5.88	4.67	0.87
4	0	-	-	-
6	45	4.65	3.25	0.48
9	4	5.13	3.94	1.97

1-Way Anova

	SS	df	MS	F	p
Between	37	3	12.481	0.860	0.465
Within	1,132	78	14.507		
Total	1,169	81			

SI Figure 143. Correlation analysis of CCK2R total staining score in lung cancer versus primary tumor size (length of longest side). IHC was performed on lung cancer tissue sections using a monoclonal antibody raised against CCK2R. The total staining score was derived by multiplying the staining intensity with the coverage score and plotted. A Spearman analysis was used to determine if there was a statistically significant correlation and a 1-way ANOVA was used to determine if there were any significant differences between groups.

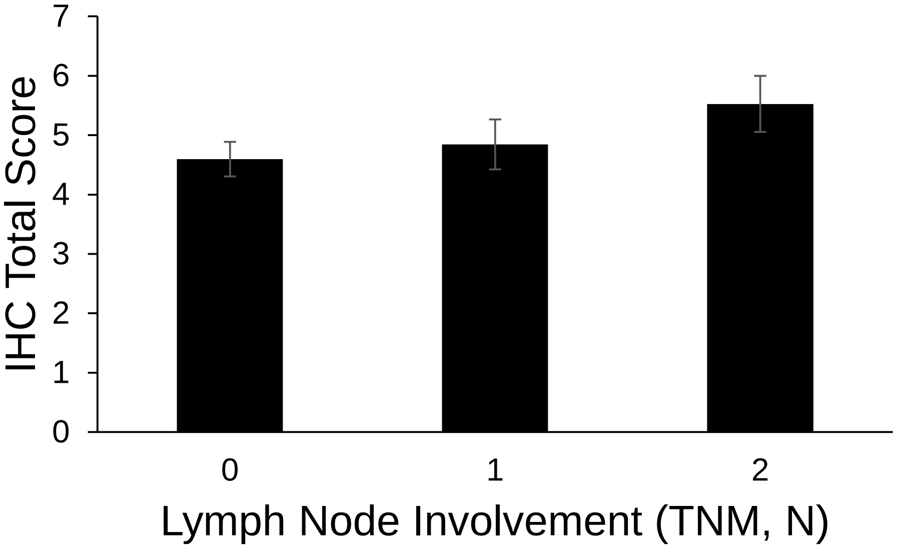

Spearman Analysis

ρ (rho) =	0.1506
t =	1.3795
p-value =	0.1714

	n	Average	St. Dev	St. Error
0	52	4.60	2.12	0.29
1	13	4.85	1.52	0.42
2	19	5.53	2.06	0.47

1-Way Anova

	SS	df	MS	F	p
Between	12.04	2	6.018	1.462	0.238
Within	333.32	81	4.115		
Total	345.36	83			

SI Figure 144. Correlation analysis of CCK2R total staining score in lung cancer versus lymph node involvement. IHC was performed on lung cancer tissue sections using a monoclonal antibody raised against CCK2R. The total staining score was derived by multiplying the staining intensity with the coverage score and plotted (error bars represent standard error of the mean). A Spearman analysis was used to determine if there was a statistically significant correlation and a 1-way ANOVA was used to determine if there were any significant differences between groups.

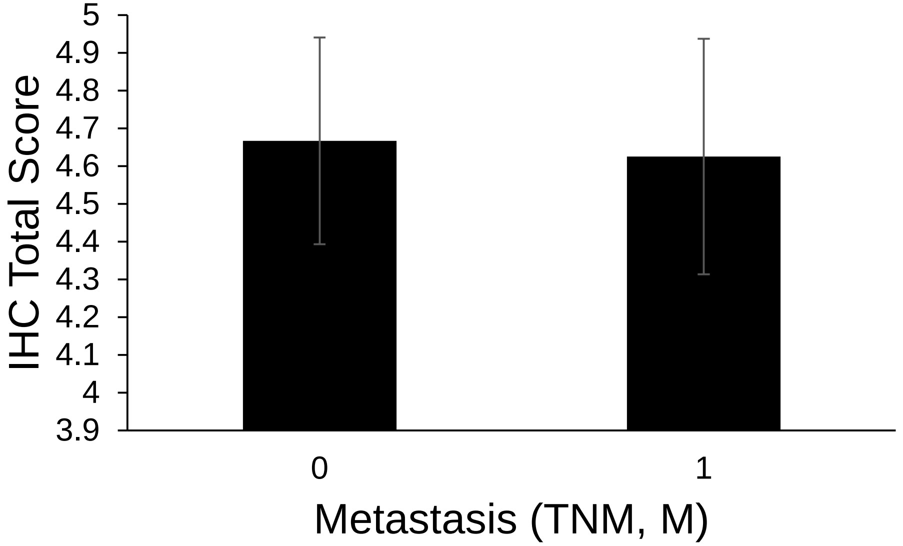

Spearman Analysis

ρ (rho) = -0.0661
t = -0.5885
p-value = 0.5579

	n	Average	St. Dev	St. Error
0	57	4.67	2.06	0.27
1	24	4.63	1.53	0.31

t-test

p-value = 0.929

SI Figure 145. Correlation analysis of CCK2R total staining score in lung cancer versus presence of metastases. IHC was performed on lung cancer tissue sections using a monoclonal antibody raised against CCK2R. The total staining score was derived by multiplying the staining intensity with the coverage score and plotted (error bars represent standard error of the mean). A Spearman analysis was used to determine if there was a statistically significant correlation and a t-test was used to determine if there were any significant differences between groups.

Spearman Analysis		
	ρ (rho) =	-
	t =	-
	p-value =	-

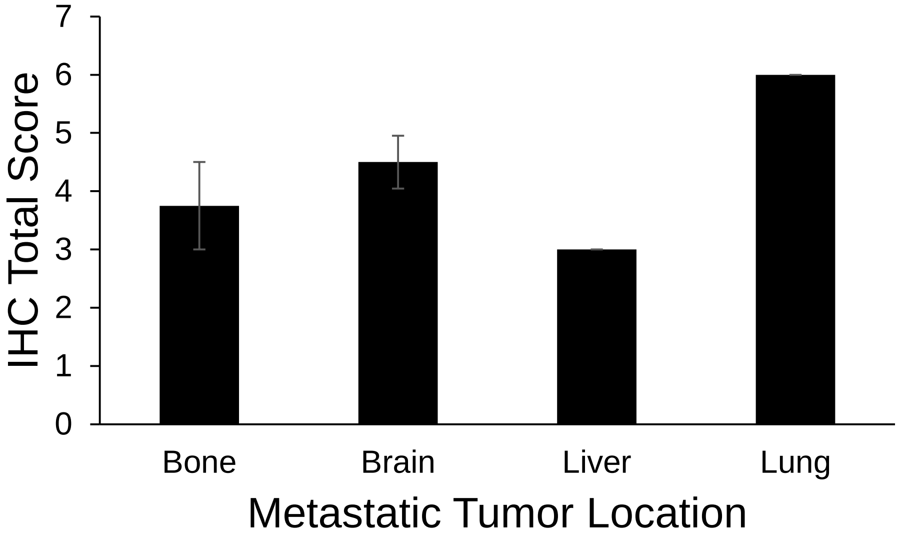

	n	Average	St. Dev	St. Error
Bone	4	3.75	1.50	0.75
Brain	12	4.50	1.57	0.45
Liver	3	3.00	0.00	0.00
Lung	6	6.00	0.00	0.00

1-Way Anova

	SS	df	MS	F	p
Between	22.41	3	7.470	4.632	**0.012**
Within	33.86	21	1.613		
Total	56.27	24			

Tukey-Kramer (p-value)

	Bone	Brain	Liver	Lung
Bone	-	0.7173	0.854	0.051
Brain		-	0.287	0.115
Liver			-	**0.015**
Lung				-

SI Figure 146. Correlation analysis of CCK2R total staining score in lung cancer versus metastatic tumor location. IHC was performed on lung cancer tissue sections using a monoclonal antibody raised against CCK2R. The total staining score was derived by multiplying the staining intensity with the coverage score and plotted (error bars represent standard error of the mean). A 1-way ANOVA followed by a Tukey-Kramer ad hoc analysis was used to determine if there were any significant differences between groups.

Spearman Analysis		
	ρ (rho) =	0.0437
	t =	0.4372
	p-value =	0.6629

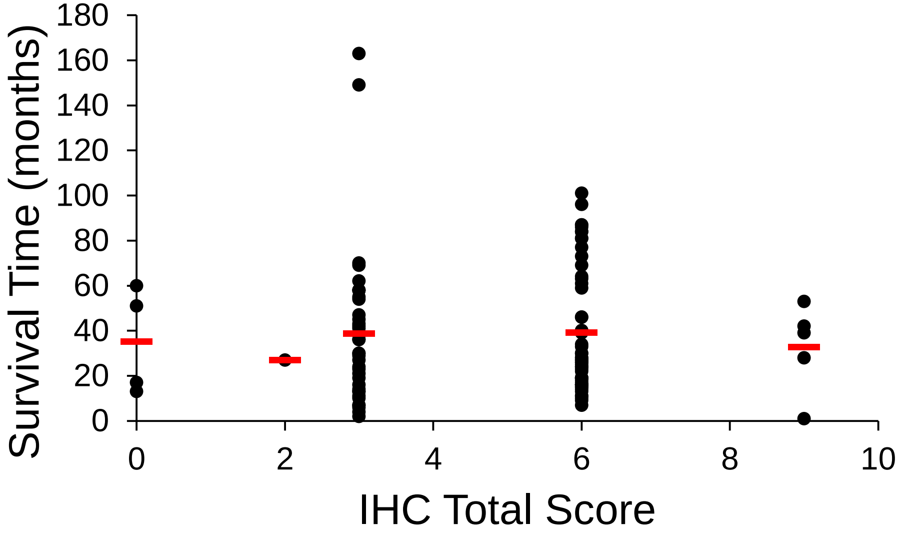

	n	Average	St. Dev	St. Error
0	4	35.25	23.73	11.86
1	0	-	-	-
2	1	27.00	-	-
3	37	38.65	34.58	5.68
4	0	-	-	-
6	55	39.11	27.51	3.71
9	5	32.60	19.78	8.85

1-Way Anova

	SS	df	MS	F	p
Between	237	3	79	0.088	0.966
Within	87,169	97	899		
Total	87,407	100			

SI Figure 147. Correlation analysis of CCK2R total staining score in lung cancer versus survival time after diagnosis. IHC was performed on lung cancer tissue sections using a monoclonal antibody raised against CCK2R. The total staining score was derived by multiplying the staining intensity with the coverage score and plotted (red bars represent population mean). A Spearman analysis was used to determine if there was a statistically significant correlation and a 1-way ANOVA was used to determine if there were any significant differences between groups.

Spearman Analysis		
	ρ (rho) =	0.0815
	t =	0.8139
	p-value =	0.4176

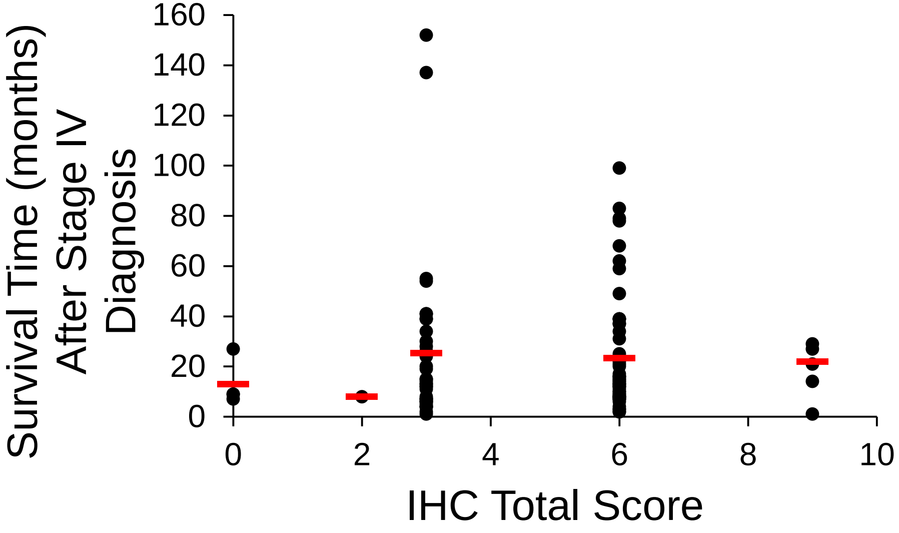

	n	Average	St. Dev	St. Error
0	4	13.00	9.38	4.69
1	0	-	-	-
2	1	8.00	-	-
3	37	25.30	32.41	5.33
4	0	-	-	-
6	53	23.42	23.46	3.22
9	6	21.83	13.18	5.38

1-Way Anova

	SS	df	MS	F	p
Between	577	3	192	0.273	0.845
Within	67,567	96	704		
Total	68,143	99			

SI Figure 148. Correlation analysis of CCK2R total staining score in lung cancer versus survival time after stage IV diagnosis. IHC was performed on lung cancer tissue sections using a monoclonal antibody raised against CCK2R. The total staining score was derived by multiplying the staining intensity with the coverage score and plotted (red bars represent population mean). A Spearman analysis was used to determine if there was a statistically significant correlation and a 1-way ANOVA was used to determine if there were any significant differences between groups.

Pancreatic Cancer

Pancreatic Cancer Images

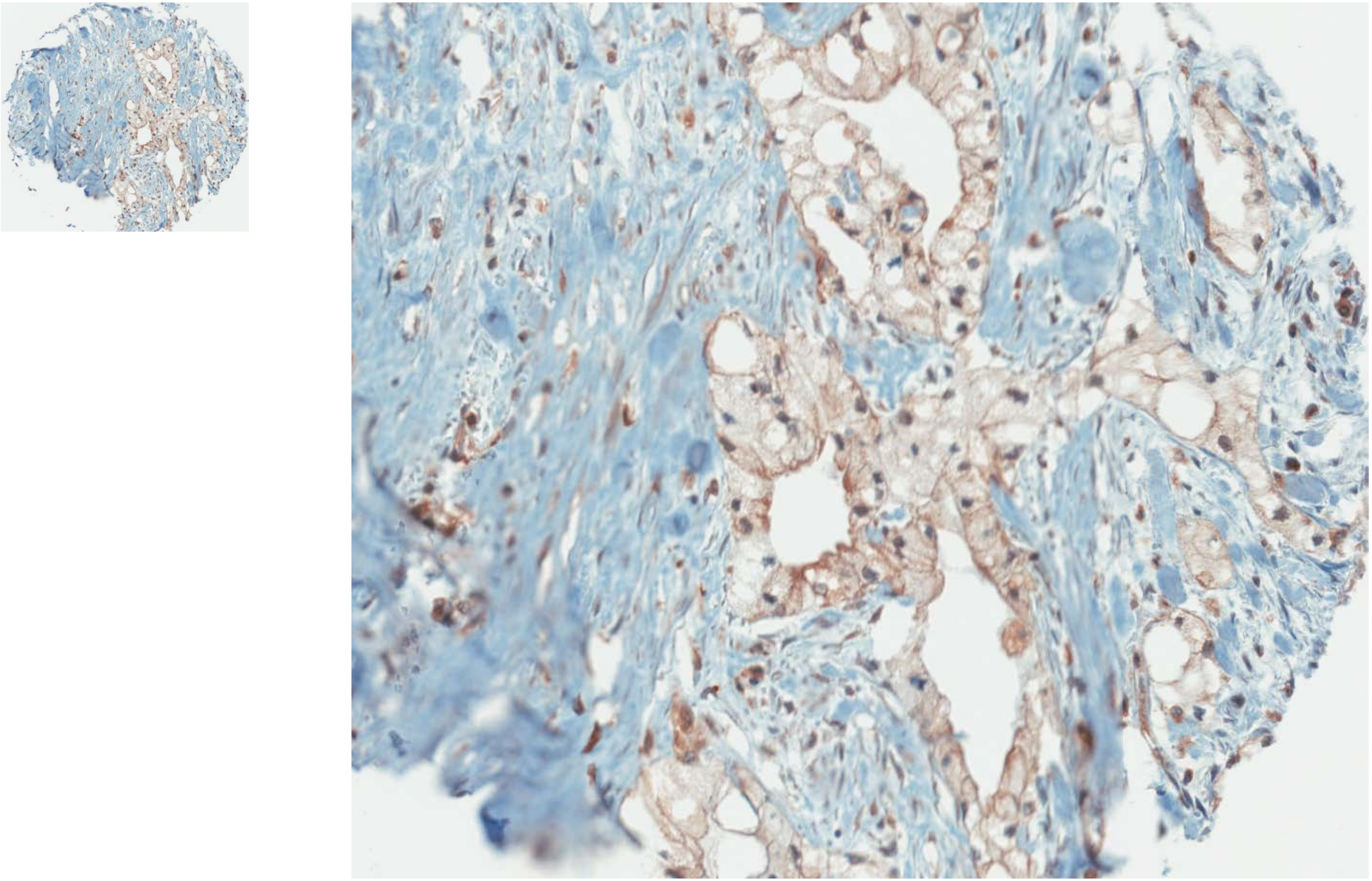

SI Figure 149. Example image of stained tissue from pancreatic cancer. IHC was performed on tissue sections using a monoclonal antibody raised against CCK2R.

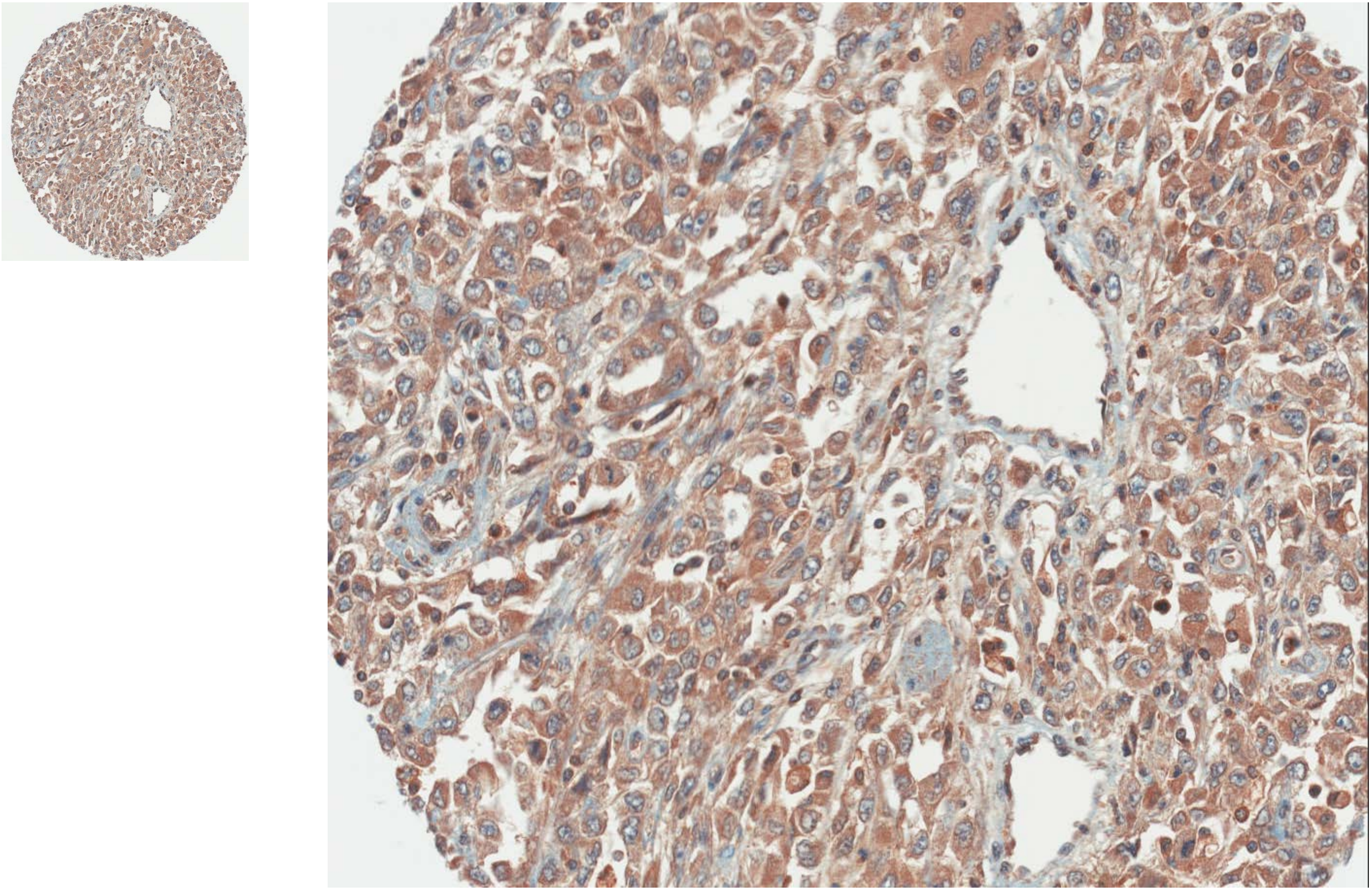

SI Figure 150. Example image of stained tissue from pancreatic cancer. IHC was performed on tissue sections using a monoclonal antibody raised against CCK2R.

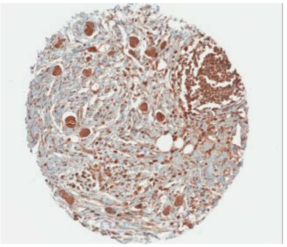

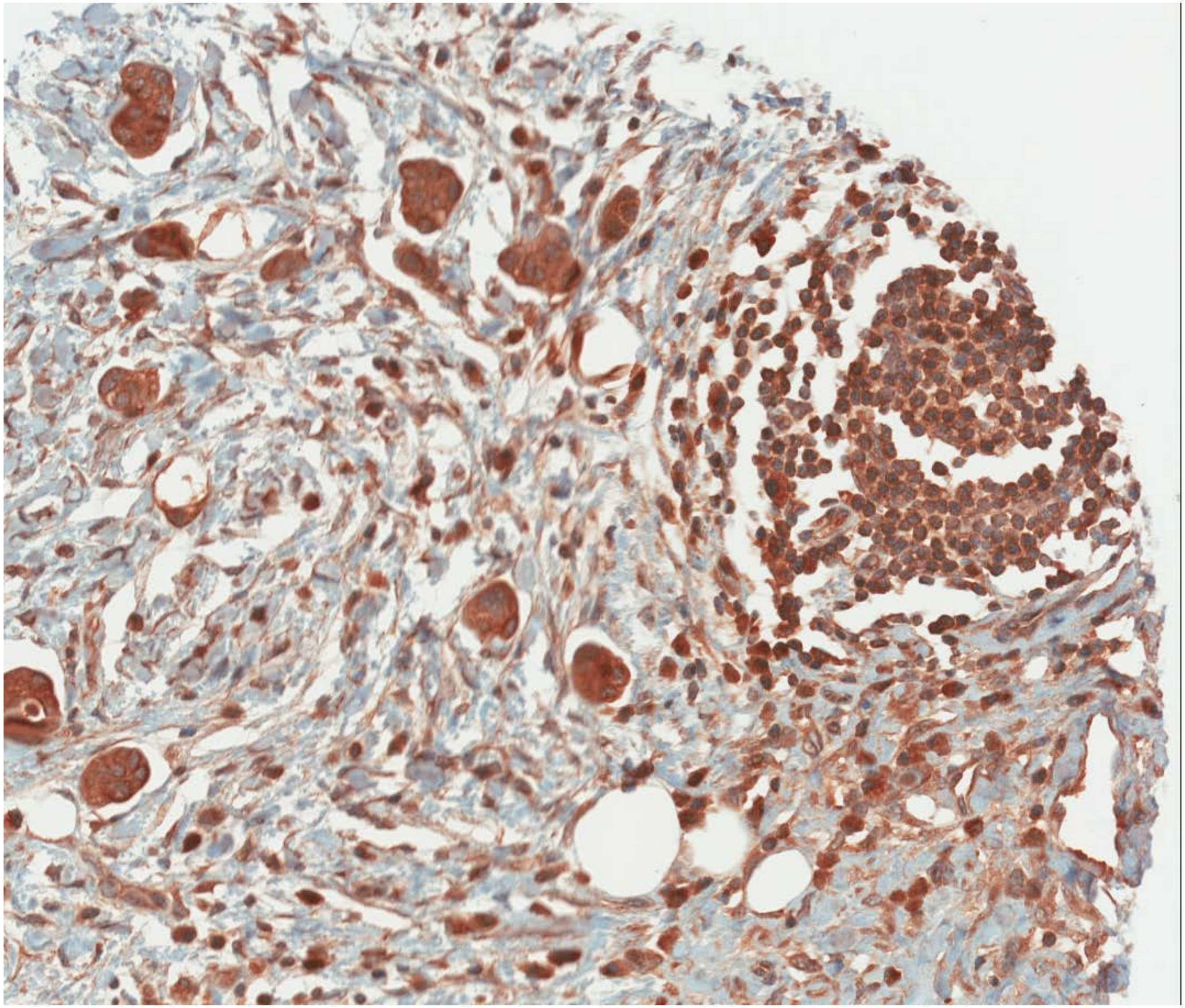

SI Figure 151. Example image of stained tissue from pancreatic cancer. IHC was performed on tissue sections using a monoclonal antibody raised against CCK2R.

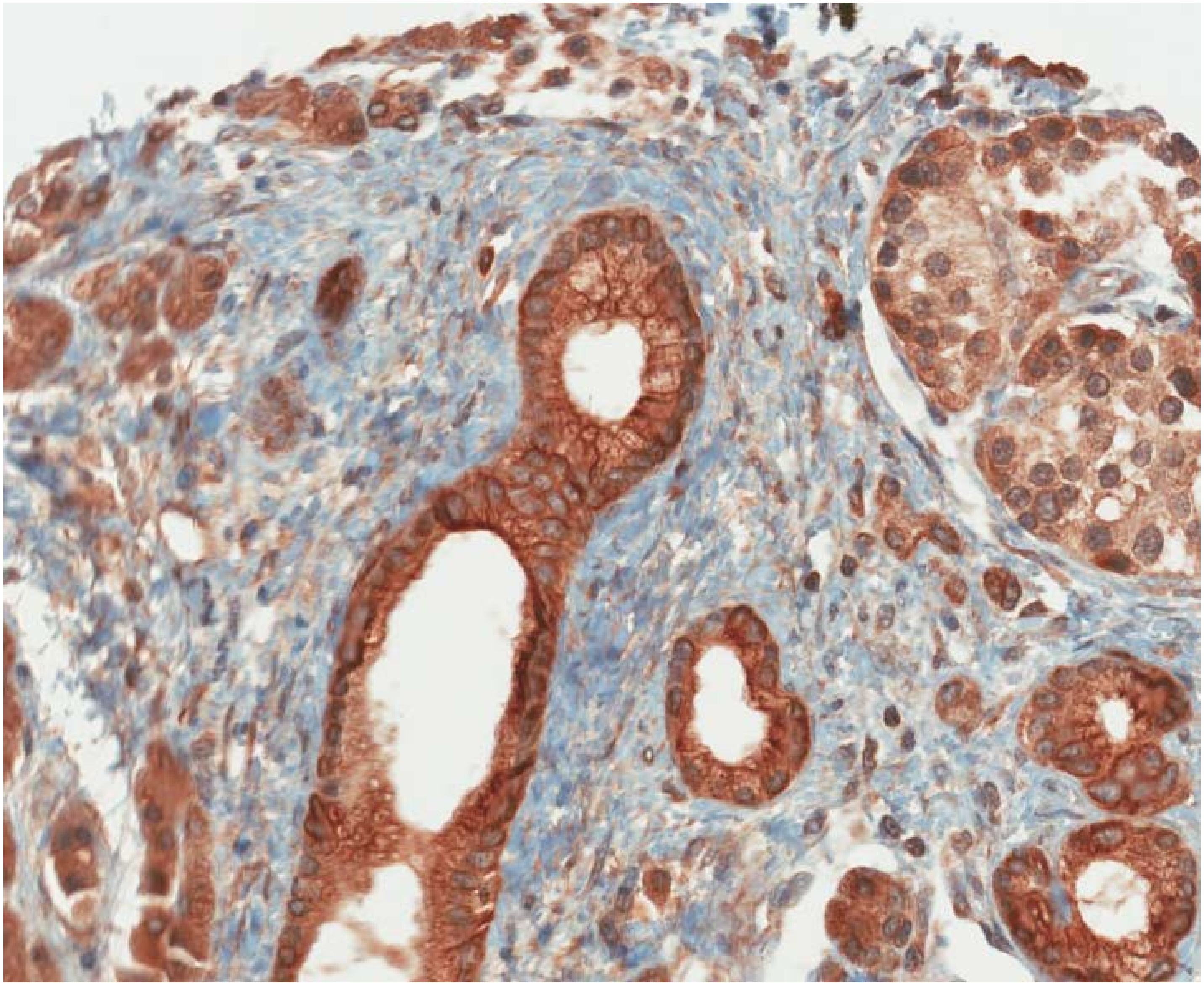

SI Figure 152. Example image of stained tissue from pancreatic cancer (Pancreatic Intraepithelial Neoplasia, PanIN1). IHC was performed on tissue sections using a monoclonal antibody raised against CCK2R.

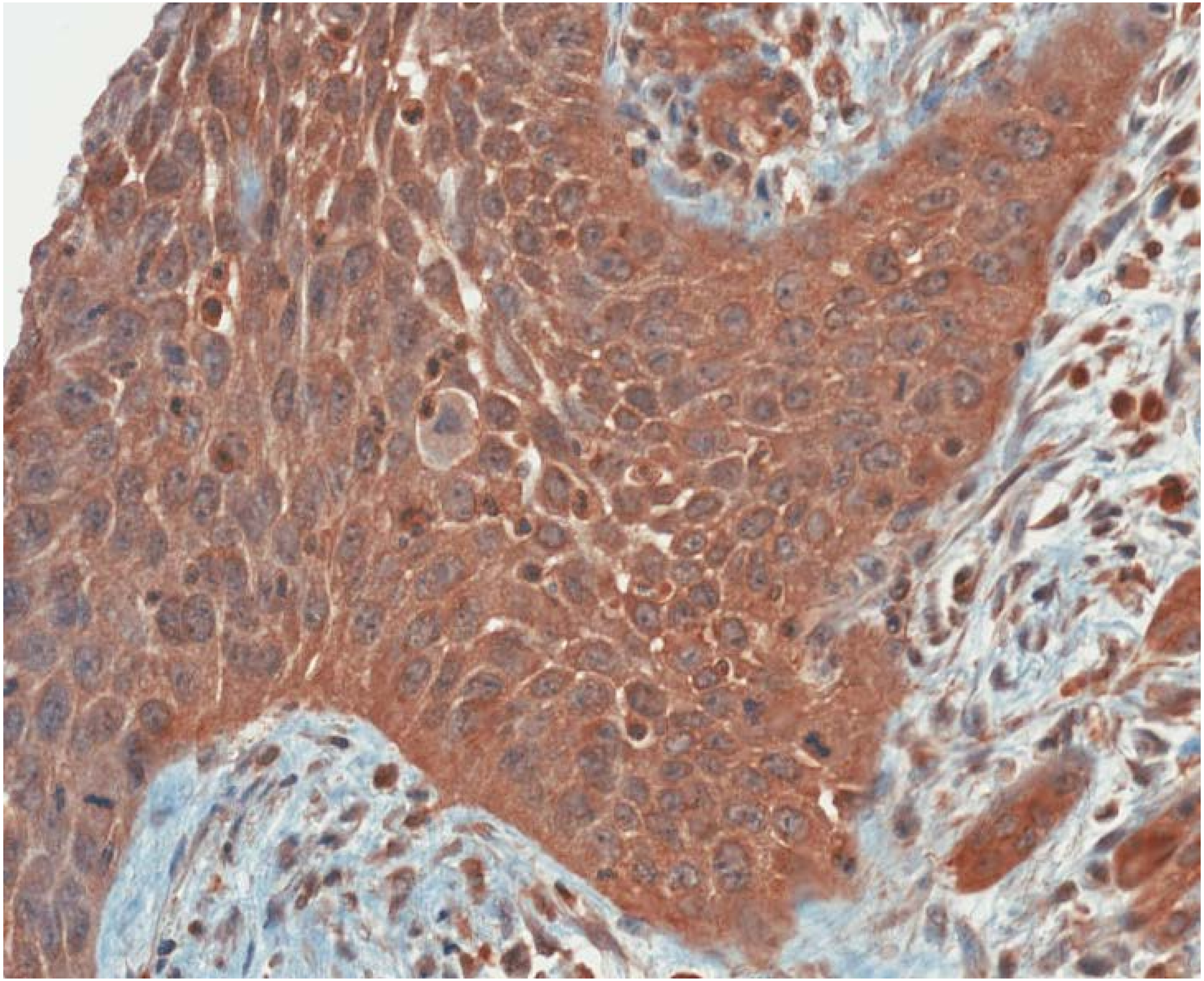

SI Figure 153. Example image of stained tissue from pancreatic cancer. IHC was performed on tissue sections using a monoclonal antibody raised against CCK2R.

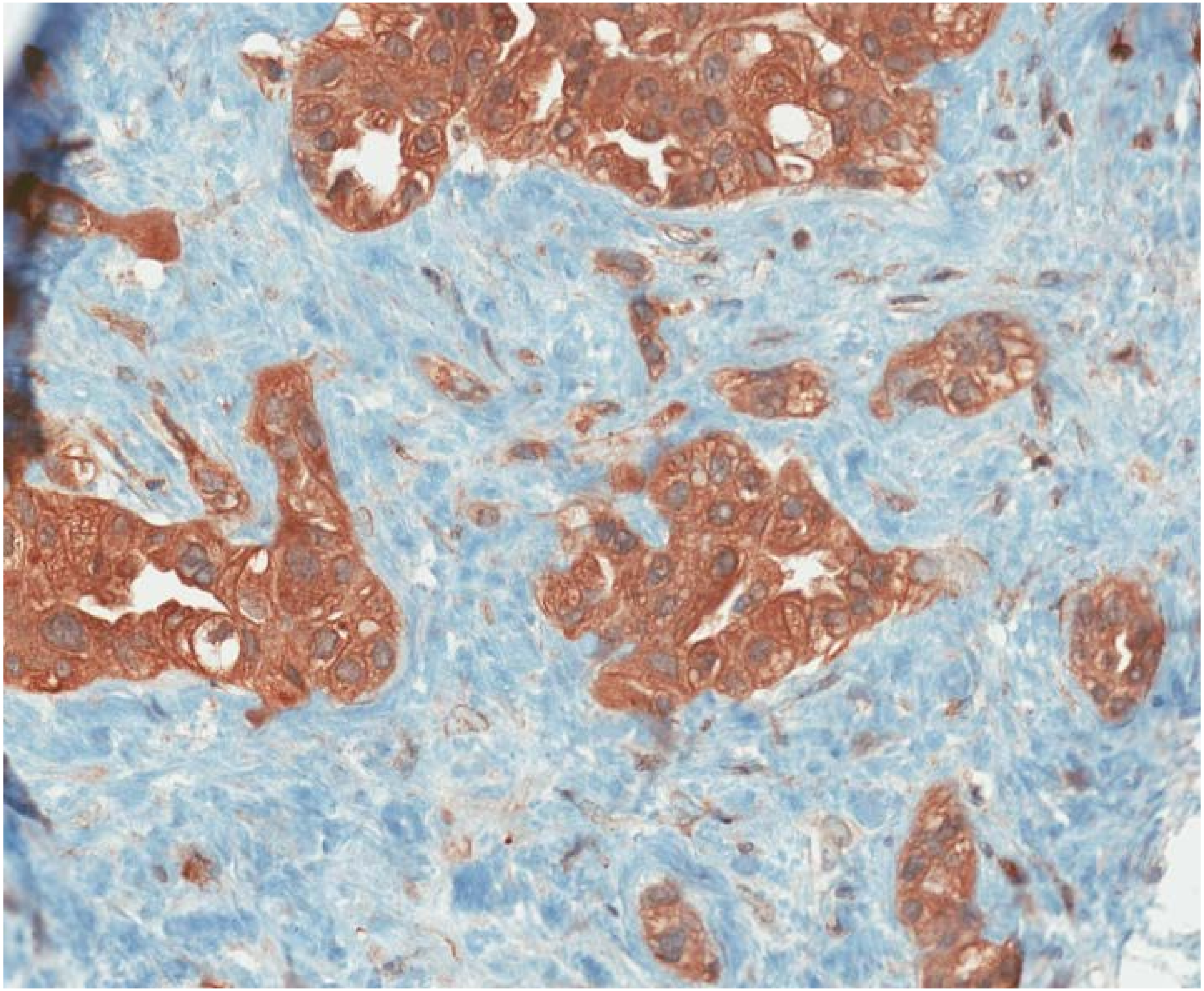

SI Figure 154. Example image of stained tissue from pancreatic cancer (oncocytic carcinoma). IHC was performed on tissue sections using a monoclonal antibody raised against CCK2R.

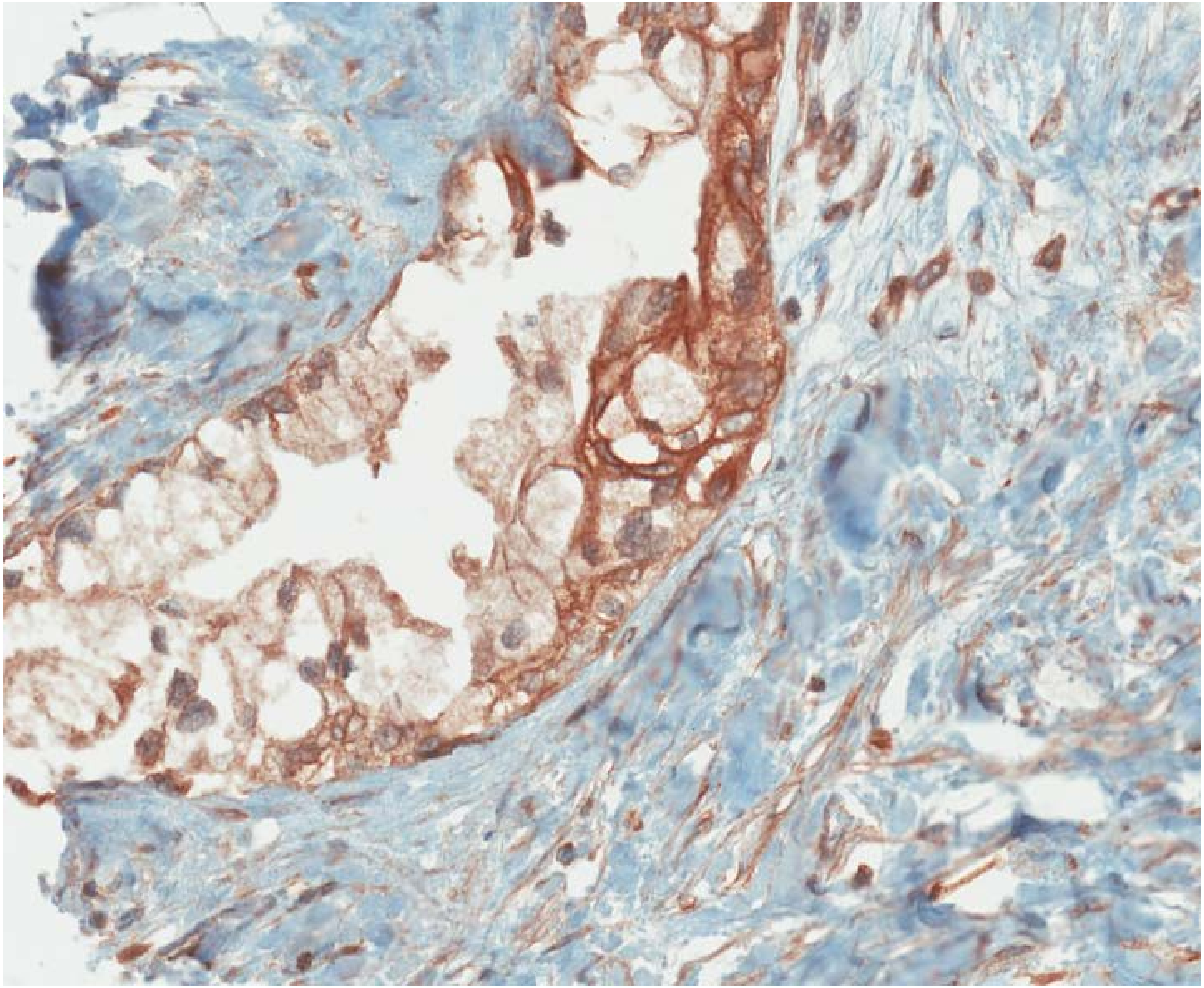

SI Figure 155. Example image of stained tissue from pancreatic cancer (adenocarcinoma). IHC was performed on tissue sections using a monoclonal antibody raised against CCK2R.

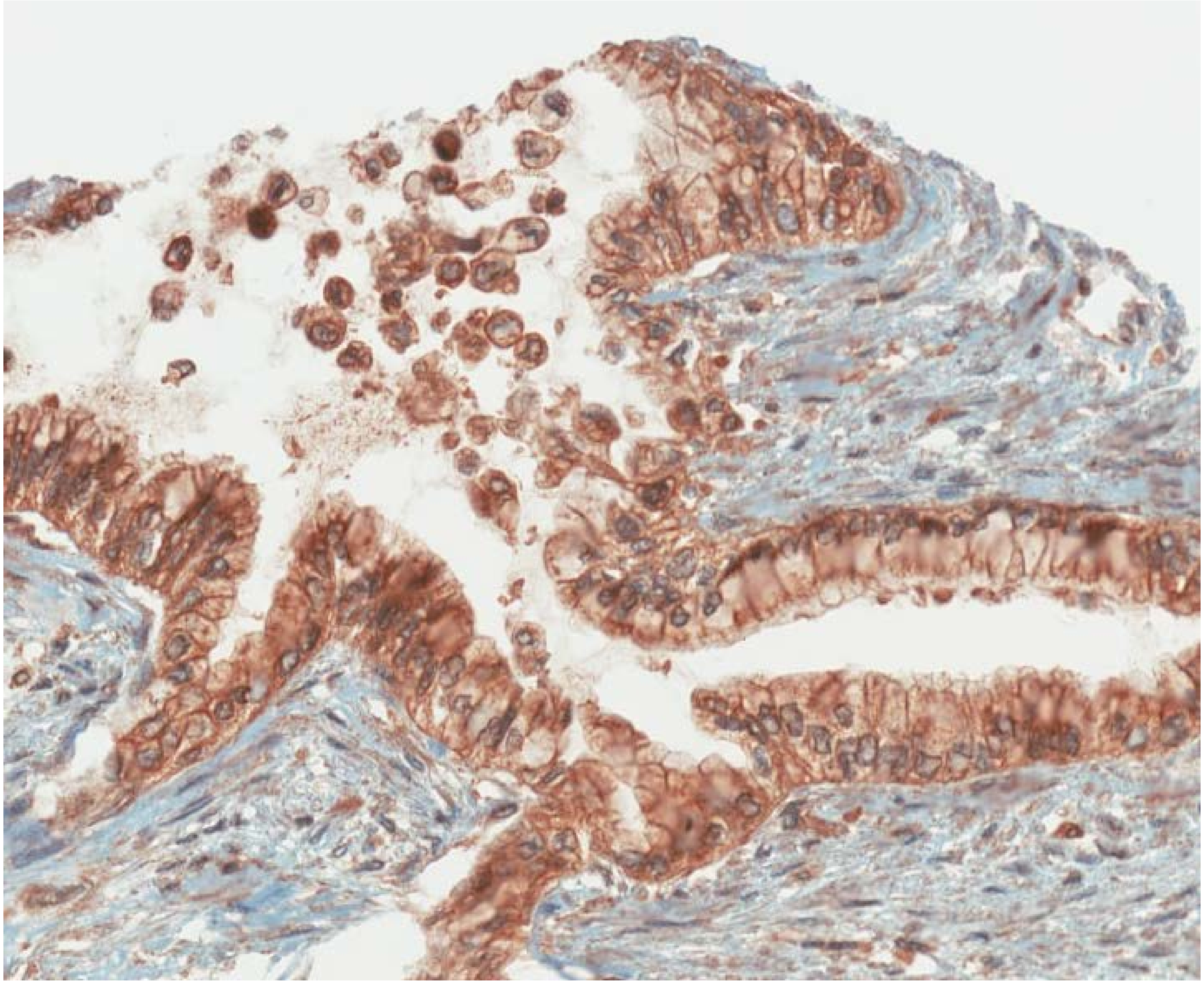

SI Figure 156. Example image of stained tissue from pancreatic cancer (adenocarcinoma). IHC was performed on tissue sections using a monoclonal antibody raised against CCK2R.

Pancreatic Cancer
Overall Summary

Pancreas - Spearman Correlation

	Sex	Age at Diagnosis	Primary Tumor Type	Primary Tumor Site	Stage	Grade	Tumor Size (TNM, T)	Tumor Size (longest dimension)	Lymph Node Involvement (TNM, N)	Metastatic (TNM, M)	Metastatic Site	Survival after Diagnosis	Survival after Stage IV Diagnosis
**Staining Intensity**	N.A.	No 0.719	N.A.	N.A.	No 0.384	No 0.207	**Yes 0.045**	**Yes 0.009**	No 0.809	N.D.	N.A.	No 0.926	N.D.
**Coverage Score**	N.A.	N.D.	N.A.	N.A.	N.D.	N.D.	N.D.	N.D.	N.D.	N.D.	N.A.	N.D.	N.D.
**Total Staining Score**	N.A.	No 0.719	N.A.	N.A.	No 0.415	No 0.2068	**Yes 0.045**	**Yes 0.009**	No 0.809	N.D.	N.A.	No 0.926	N.D.

Pancreas - Spearman Correlation - ANOVA/t-test

	Sex	Age at Diagnosis	Primary Tumor Type	Primary Tumor Site	Stage	Grade	Tumor Size (TNM, T)	Tumor Size (longest dimension)	Lymph Node Involvement (TNM, N)	Metastatic (TNM, M)	Metastatic Site	Survival after Diagnosis	Survival after Stage IV Diagnosis
**Staining Intensity**	No 0.476	No 0.413	No 0.617	No 0.694	**Yes 0.004**	No 0.586	No 0.728	No 0.500	No 0.778	N.D.	N.D.	No 0.713	N.D.
**Coverage Score**	No 1.00	N.D.	No 1.00	No 1.00	N.D.	No 1.00	N.D.	N.D.	No 1.00	N.D.	N.D.	N.D.	N.D.
**Total Staining Score**	No 0.476	N.D.	No 0.617	No 0.685	**Yes 0.003**	No 0.59	N.D.	No 0.500	No 0.775	N.D.	N.D.	N.D.	N.D.

SI Figure 157. Correlation summary of CCK2R in pancreatic cancer. IHC was performed on pancreatic tumor tissue sections using a monoclonal antibody raised against CCK2R. The staining intensity, coverage score and total staining score were compared against available patient data. If appropriate, a spearman analysis was used to determine if any significant correlation exists while a 1-way ANOVA or t-test was used to determine if a significant difference exists between groups. Whether the test was statistically significant and the p-value is listed. N.A. – not applicable (this statistical test was not applicable to this data set). N.D. – not determined (this statistical test could not be performed, generally due to a lack of the number of samples within a group or all data was in a single group).

Pancreatic Cancer
Staining Intensity

Pancreatic Cancer

	n	Average	St. Dev	St. Error
Staining Intensity	55	2.65	0.55	0.07

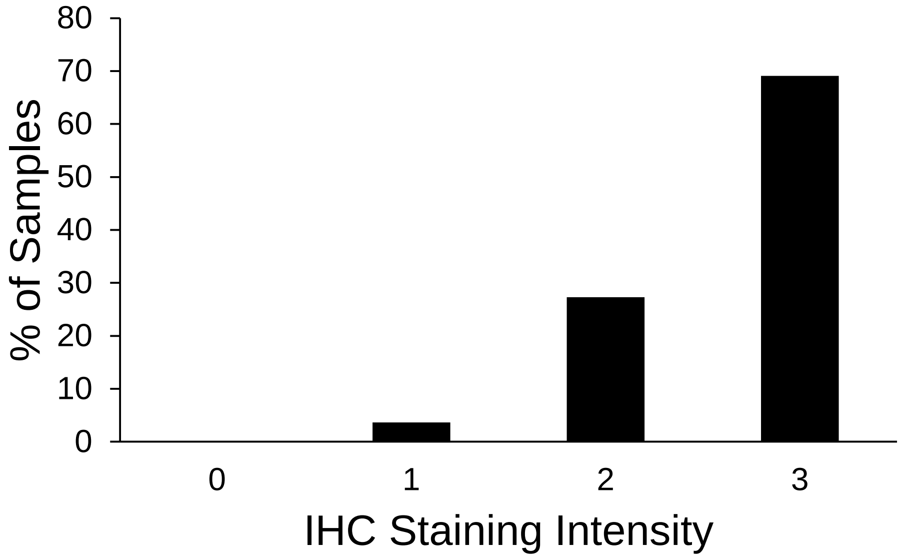

	Staining Intensity			
	0	1	2	3
n	0	2	15	38
%	0.00	3.64	27.27	69.09

Normal Pancreas

	n	Average	St. Dev	St. Error
Staining Intensity	66	2.52	0.66	0.08

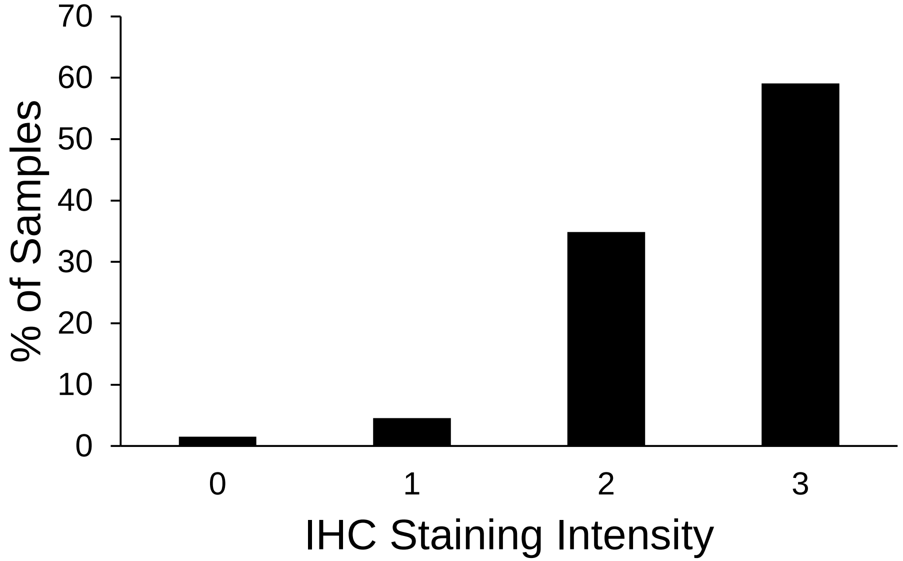

	Staining Intensity			
	0	1	2	3
n	1	3	23	39
%	1.52	4.55	34.85	59.09

SI Figure 158. CCK2R Staining Intensity for cancer and normal tissue from the pancreas. IHC was performed on tissue sections using a monoclonal antibody raised against CCK2R. The intensity of staining was graded on a scale of 0 to 3 and plotted.

Pancreatic Cancer - Staining Intensity

	Sex	Age at Diagnosis	Primary Tumor Type	Primary Tumor Site	Stage	Grade	Tumor Size (TNM, T)	Tumor Size (longest dimension)	Lymph Node Involvement (TNM, N)	Metastatic (TNM, M)	Metastatic Site	Survival after Diagnosis	Survival after Stage IV Diagnosis
**Spearman Correlation**	N.A.	No 0.719	N.A.	N.A.	No 0.384	No 0.207	**Yes 0.045**	**Yes 0.009**	No 0.809	N.D.	N.A.	No 0.926	N.D.
**ANOVA/t-test**	No 0.476	No 0.413	No 0.617	No 0.694	**Yes 0.004**	No 0.586	No 0.728	No 0.500	No 0.778	N.D.	N.D.	No 0.713	N.D.

SI Figure 159. Staining intensity correlation summary of CCK2R in pancreatic cancer. IHC was performed on pancreatic tumor tissue sections using a monoclonal antibody raised against CCK2R. The staining intensity was compared against available patient data. If appropriate, a spearman analysis was used to determine if any significant correlation exists while a 1-way ANOVA or t-test was used to determine if a significant difference exists between groups. Whether the test was statistically significant and the p-value is listed. N.A. – not applicable (this statistical test was not applicable to this data set). N.D. – not determined (this statistical test could not be performed, generally due to a lack of the number of samples within a group or all data was in a single group).

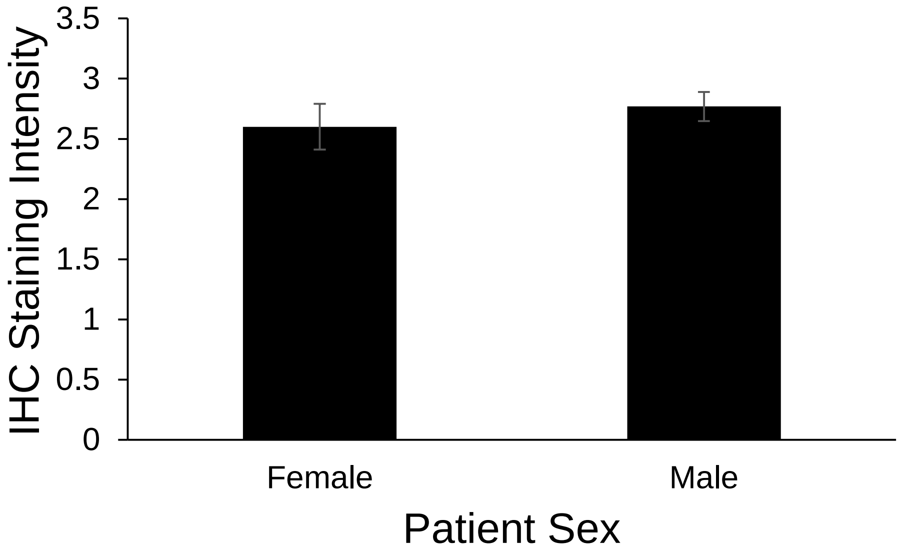

Spearman Analysis	
ρ (rho) =	-
t =	-
p-value =	-

	n	Average	St. Dev	St. Error
Female	15	2.60	0.74	0.19
Male	13	2.77	0.44	0.12

t-test

p-value = 0.476

SI Figure 160. Correlation analysis of CCK2R staining intensity in pancreatic cancer versus patient sex. IHC was performed on pancreatic cancer tissue sections using a monoclonal antibody raised against CCK2R. The staining intensity was graded on a scale of 0 to 3 and plotted (error bars represent standard error of the mean). A t-test was used to determine if there were any significant differences between groups.

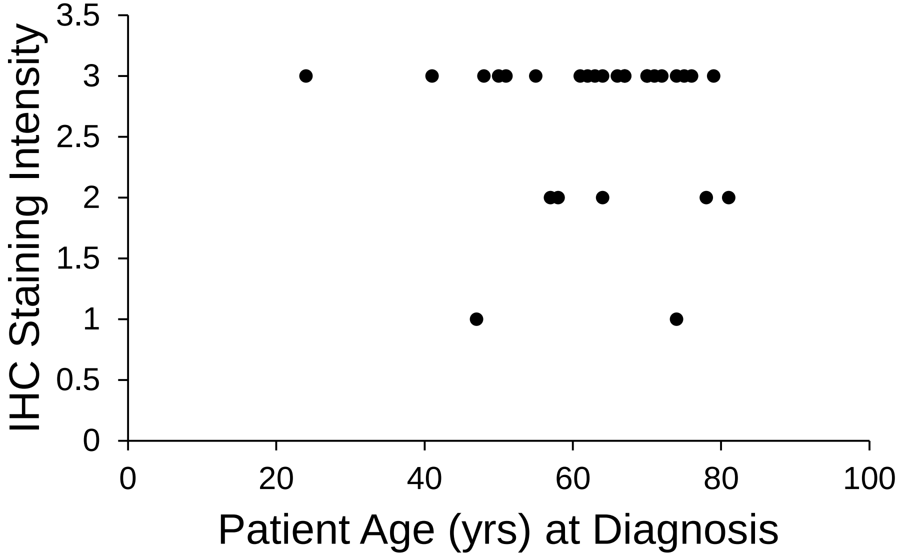

Spearman Analysis

ρ (rho) = -0.0711
t = -0.3634
p-value = 0.7191

	n	Average	St. Dev	St. Error
0	0	-	-	-
1	2	60.50	-	-
2	5	67.60	11.24	5.03
3	21	62.19	13.37	2.92

t-test

p-value = 0.413

SI Figure 161. Correlation analysis of CCK2R staining intensity in pancreatic cancer versus patient age at diagnosis. IHC was performed on pancreatic cancer tissue sections using a monoclonal antibody raised against CCK2R. The staining intensity was graded on a scale of 0 to 3 and plotted. A Spearman analysis was used to determine if there was a statistically significant correlation and a t-test was used to determine if there were any significant differences between groups.

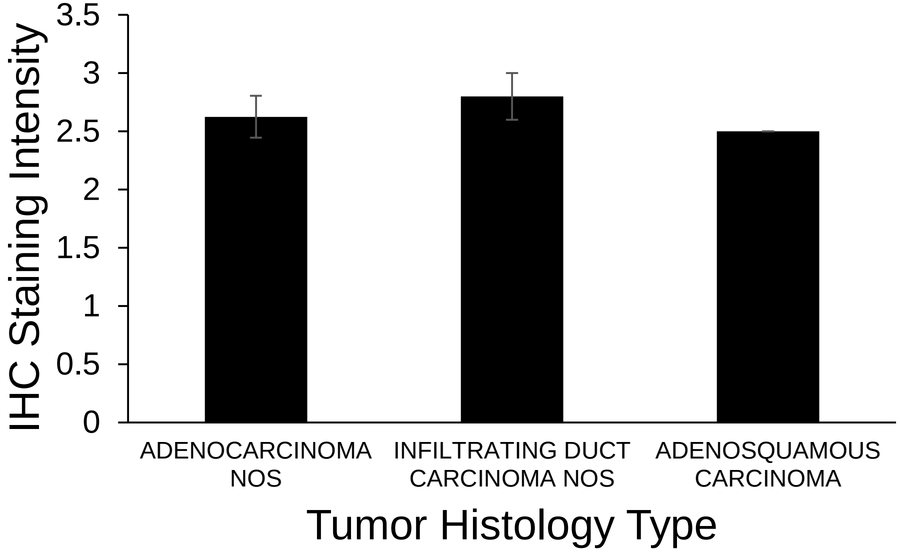

Spearman Analysis		
	ρ (rho) =	-
	t =	-
	p-value =	-

	n	Average	St. Dev	St. Error
ADENOCARCINOMA NOS	16	2.63	0.72	0.18
INFILTRATING DUCT CARCINOMA NOS	5	2.80	0.45	0.20
ADENOSQUAMOUS CARCINOMA	2	2.50	-	-

t-test

p-value = 0.6165

SI Figure 162. Correlation analysis of CCK2R staining intensity in pancreatic cancer versus type of primary tumor. IHC was performed on pancreatic cancer tissue sections using a monoclonal antibody raised against CCK2R. The staining intensity was graded on a scale of 0 to 3 and plotted (error bars represent standard error of the mean). A t-test was used to determine if there were any significant differences between groups.

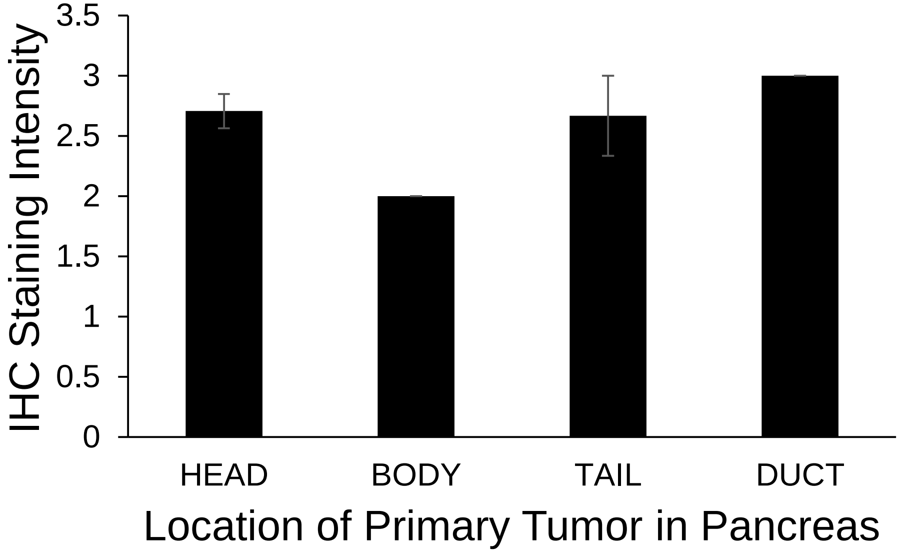

Spearman Analysis		
	ρ (rho) =	-
	t =	-
	p-value =	-

	n	Average	St. Dev	St. Error
HEAD	17	2.71	0.59	0.14
BODY	2	2.00	-	-
TAIL	3	2.67	0.58	0.33
DUCT	3	3	0	0

1-Way Anova

	SS	df	MS	F	p
Between	0.23	2	0.116	0.373	0.694
Within	6.24	20	0.312		
Total	6.48	22			

SI Figure 163. Correlation analysis of CCK2R staining intensity in pancreatic cancer versus location of primary tumor. IHC was performed on pancreatic cancer tissue sections using a monoclonal antibody raised against CCK2R. The staining intensity was graded on a scale of 0 to 3 and plotted (error bars represent standard error of the mean). A 1-way ANOVA was used to determine if there were any significant differences between groups.

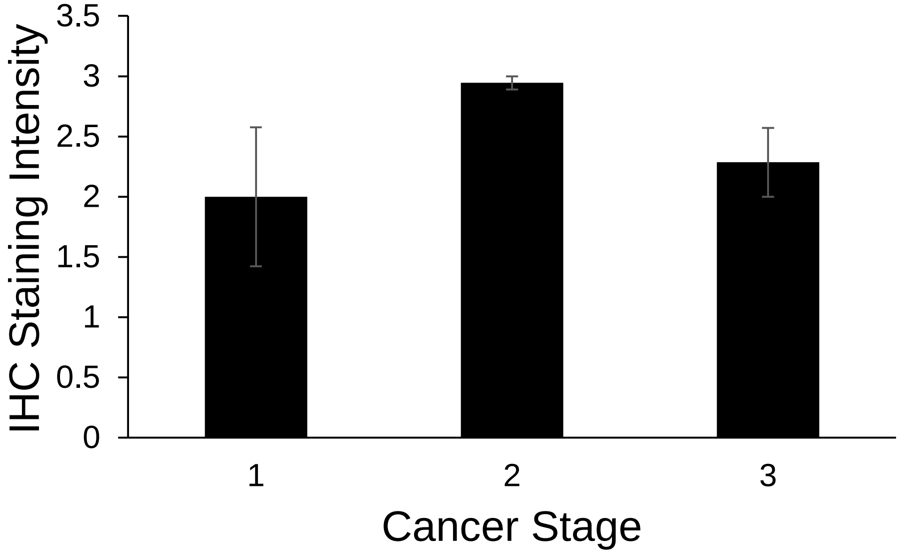

Spearman Analysis

ρ (rho) =	-0.1709
t =	-0.8846
p-value =	0.3842

	n	Average	St. Dev	St. Error
1	3	2.00	1.00	0.58
2	18	2.94	0.24	0.06
3	7	2.29	0.76	0.29
4	0	-	-	-

1-Way Anova

	SS	df	MS	F	p
Between	3.67	2	1.834	7.115	**0.004**
Within	6.45	25	0.258		
Total	10.11	27			

Tukey-Kramer (p-value)

	1	2	3
1	-	**0.0161**	0.683
2		-	**0.019**
3			-

SI Figure 164. Correlation analysis of CCK2R staining intensity in pancreatic cancer versus cancer stage. IHC was performed on pancreatic cancer tissue sections using a monoclonal antibody raised against CCK2R. The staining intensity was graded on a scale of 0 to 3 and plotted (error bars represent standard error of the mean). A Spearman analysis was used to determine if there was a statistically significant correlation and a 1-way ANOVA followed by a Tukey-Kramer ad hoc analysis was used to determine if there were any significant differences between groups.

Spearman Analysis		
	ρ (rho) =	0.2558
	t =	1.2961
	p-value =	0.2068

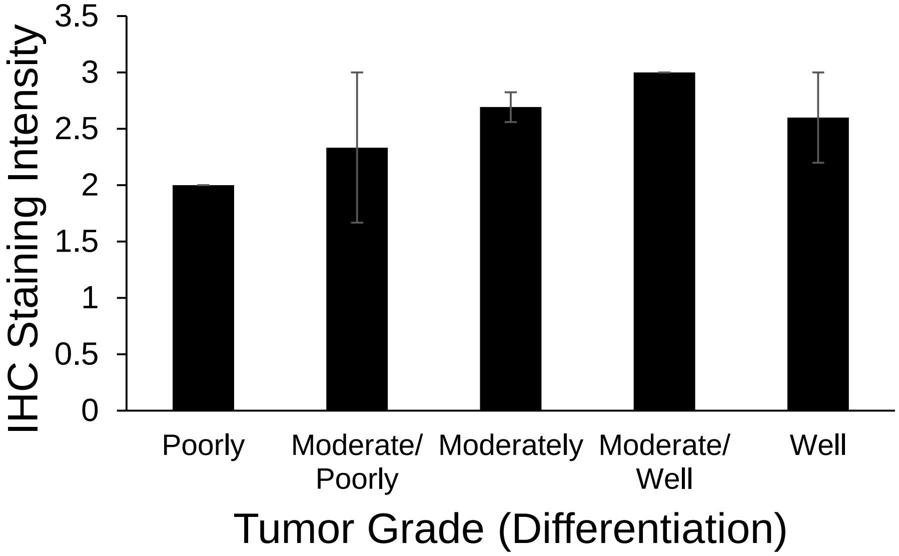

	n	Average	St. Dev	St. Error
Poorly	1	2.00	-	-
Moderate/ Poorly	3	2.33	1.15	0.67
Moderately	13	2.69	0.48	0.13
Moderate/ Well	4	3.00	0.00	0.00
Well	5	2.60	0.89	0.40

1-Way Anova

	SS	df	MS	F	p
Between	0.81	3	0.270	0.659	0.586
Within	8.61	21	0.410		
Total	9.42	24			

SI Figure 165. Correlation analysis of CCK2R staining intensity in pancreatic cancer versus primary tumor grade. IHC was performed on pancreatic cancer tissue sections using a monoclonal antibody raised against CCK2R. The staining intensity was graded on a scale of 0 to 3 and plotted (error bars represent standard error of the mean). A Spearman analysis was used to determine if there was a statistically significant correlation and a 1-way ANOVA was used to determine if there were any significant differences between groups.

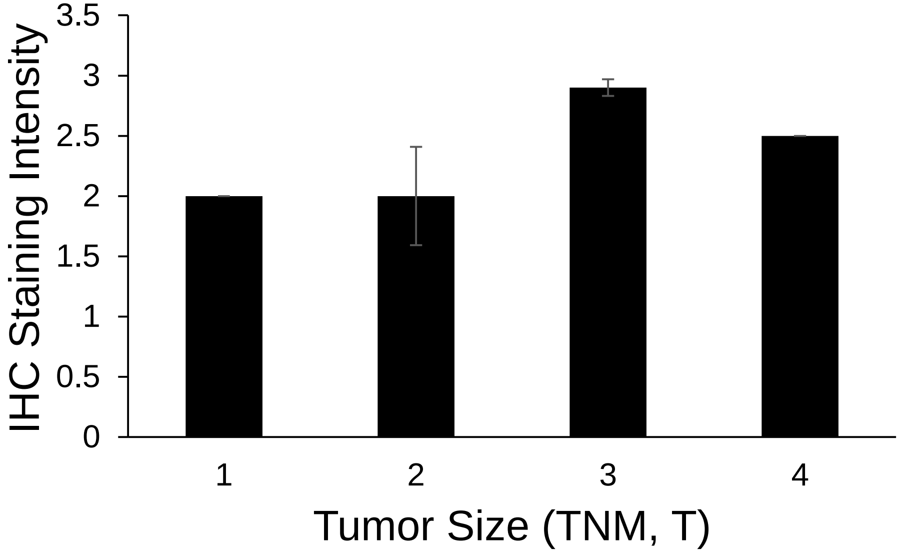

Spearman Analysis

	ρ (rho) =	0.3805
	t =	2.0979
	p-value =	**0.0454**

	n	Average	St. Dev	St. Error
1	2	2.00	-	-
2	4	2.00	0.82	0.41
3	20	2.90	0.31	0.07
4	2	2.50	-	-

t-test

p-value = 0.7282

SI Figure 166. Correlation analysis of CCK2R staining intensity in pancreatic cancer versus primary tumor size. IHC was performed on pancreatic cancer tissue sections using a monoclonal antibody raised against CCK2R. The staining intensity was graded on a scale of 0 to 3 and plotted (error bars represent standard error of the mean). A Spearman analysis was used to determine if there was a statistically significant correlation and a t-test was used to determine if there were any significant differences between groups.

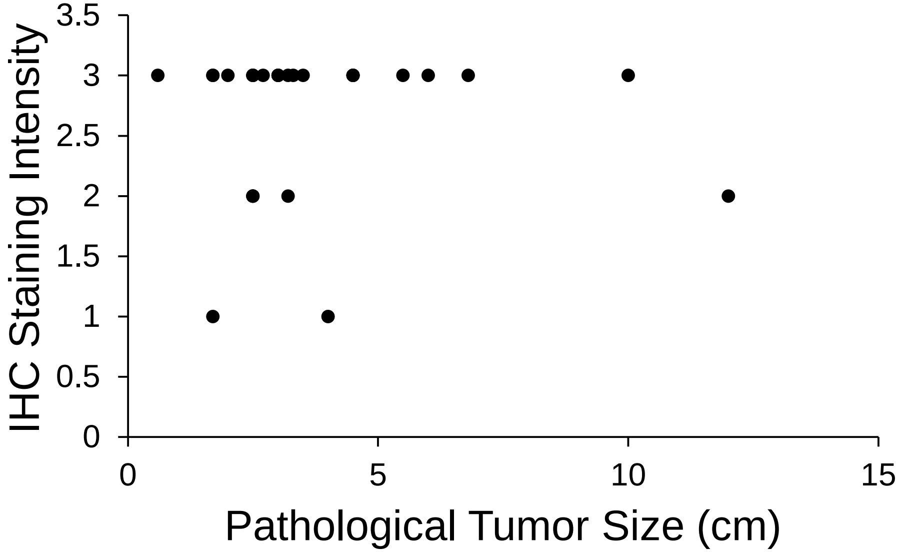

Spearman Analysis		
	ρ (rho) =	0.4831
	t =	2.8138
	p-value =	**0.0090**

	n	Average	St. Dev	St. Error
0	0	-	-	-
1	2	2.85	1.63	1.15
2	5	4.54	4.18	1.87
3	21	3.67	2.09	0.46

t-test

p-value = 0.4997

SI Figure 167. Correlation analysis of CCK2R staining intensity in pancreatic cancer versus size of primary tumor (length of longest side). IHC was performed on pancreatic cancer tissue sections using a monoclonal antibody raised against CCK2R. The staining intensity was graded on a scale of 0 to 3 and plotted. A Spearman analysis was used to determine if there was a statistically significant correlation and a t-test was used to determine if there were any significant differences between groups.

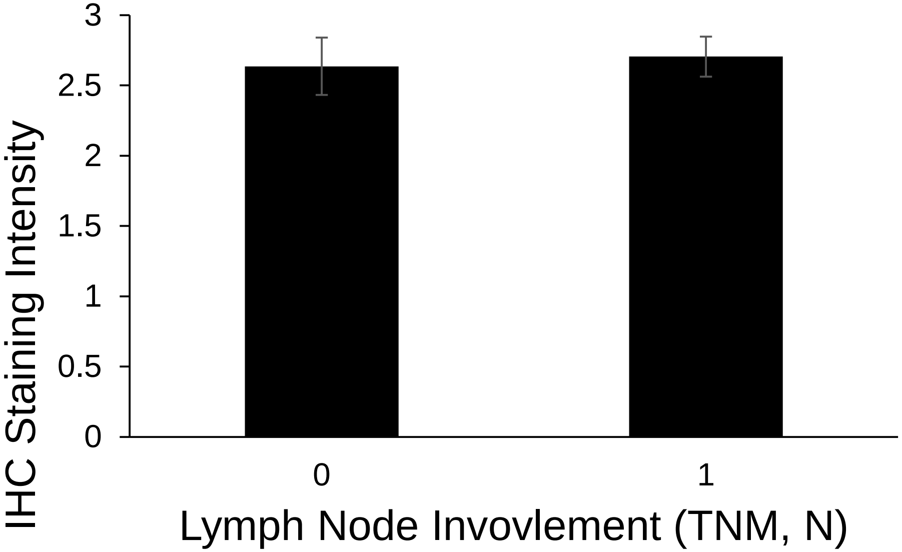

Spearman Analysis

ρ (rho) = 0.0478
t = 0.2442
p-value = 0.8089

	n	Average	St. Dev	St. Error
0	11	2.64	0.67	0.20
1	17	2.71	0.59	0.14

t-test

p-value = 0.7751

SI Figure 168. Correlation analysis of CCK2R staining intensity in pancreatic cancer versus lymph node involvement. IHC was performed on pancreatic cancer tissue sections using a monoclonal antibody raised against CCK2R. The staining intensity was graded on a scale of 0 to 3 and plotted (error bars represent standard error of the mean). A Spearman analysis was used to determine if there was a statistically significant correlation and a t-test was used to determine if there were any significant differences between groups.

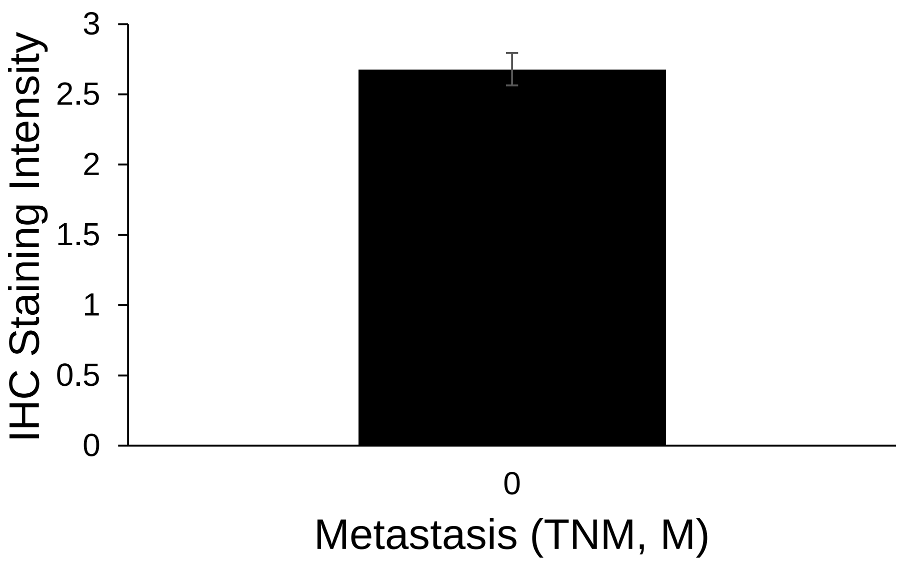

Spearman Analysis	ρ (rho) =	-
	t =	-
	p-value =	-

	n	Average	St. Dev	St. Error
0	28	2.68	0.61	0.12

t-test

p-value = -

SI Figure 169. Correlation analysis of CCK2R staining intensity in pancreatic cancer versus metastases. IHC was performed on pancreatic cancer tissue sections using a monoclonal antibody raised against CCK2R. The staining intensity was graded on a scale of 0 to 3 and plotted (error bars represent standard error of the mean). No statistical tests could be performed.

Spearman Analysis

ρ (rho) =	0.0185
t =	0.0942
p-value =	0.9257

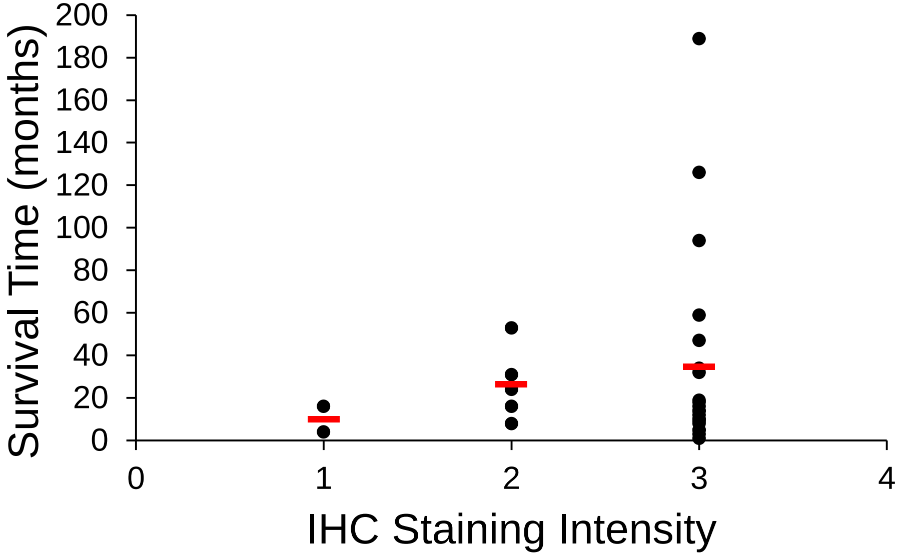

	n	Average	St. Dev	St. Error
0	0	-	-	-
1	2	10.00	-	-
2	5	26.40	17.18	7.69
3	21	34.52	47.52	10.37

t-test

p-value = 0.713

SI Figure 170. Correlation analysis of CCK2R staining intensity in pancreatic cancer versus survival time after diagnosis. IHC was performed on pancreatic cancer tissue sections using a monoclonal antibody raised against CCK2R. The staining intensity was graded on a scale of 0 to 3 and plotted (red bars represent population mean). A Spearman analysis was used to determine if there was a statistically significant correlation and a t-test was used to determine if there were any significant differences between groups.

Pancreatic Cancer
Coverage Score Correlations

Pancreatic Cancer

	n	Average	St. Dev	St. Error
Coverage Score	55	3.00	0.00	0.00

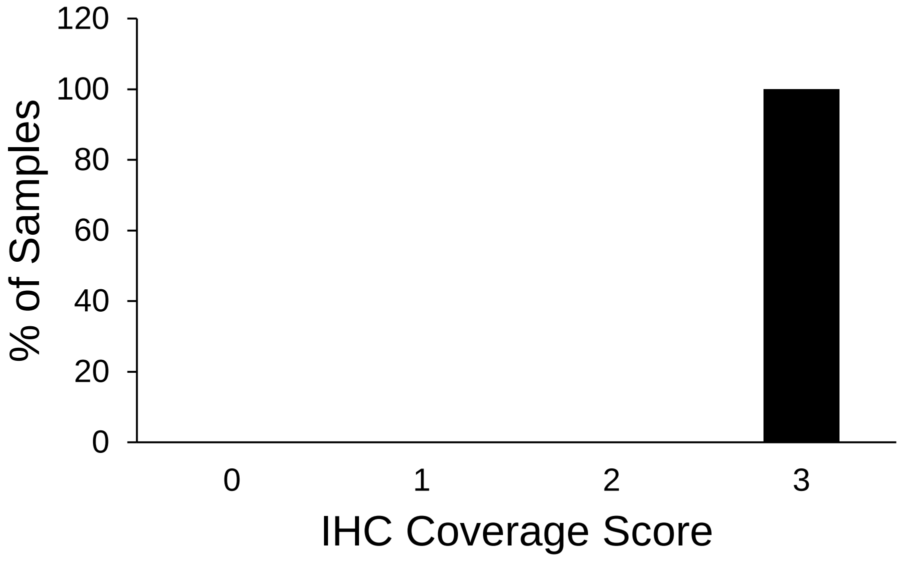

Coverage Score	0	1	2	3
n	0	0	0	55
%	0.00	0.00	0.00	100.00

Normal Pancreas

	n	Average	St. Dev	St. Error
Coverage Score	66	2.95	0.37	0.05

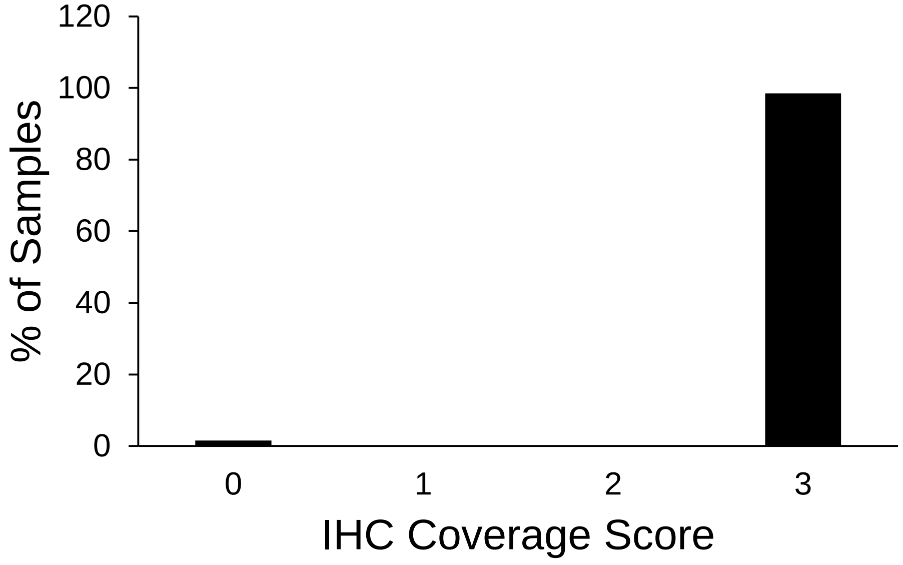

	0	1	2	3
n	1	0	0	65
%	1.52	0.00	0.00	98.48

SI Figure 171. CCK2R Coverage Score for cancer and normal tissue from the pancreas. IHC was performed on tissue sections using a monoclonal antibody raised against CCK2R. The area stained (coverage) was graded on a scale of 0 to 3 and plotted.

Pancreatic Cancer - Coverage Score

	Sex	Age at Diagnosis	Primary Tumor Type	Primary Tumor Site	Stage	Grade	Tumor Size (TNM, T)	Tumor Size (longest dimension)	Lymph Node Involvement (TNM, N)	Metastatic (TNM, M)	Metastatic Site	Survival after Diagnosis	Survival after Stage IV Diagnosis
**Spearman Correlation**	N.A.	N.D.	N.A.	N.A.	N.D.	N.D.	N.D.	N.D.	N.D.	N.D.	N.A.	N.D.	N.D.
**ANOVA/t-test**	No 1.00	N.D.	No 1.00	No 1.00	N.D.	No 1.00	N.D.	N.D.	No 1.00	N.D.	N.D.	N.D.	N.D.

SI Figure 172. Coverage score correlation summary of CCK2R in pancreatic cancer. IHC was performed on pancreatic tumor tissue sections using a monoclonal antibody raised against CCK2R. The coverage score was compared against available patient data. If appropriate, a spearman analysis was used to determine if any significant correlation exists while a 1-way ANOVA or t-test was used to determine if a significant difference exists between groups. Whether the test was statistically significant and the p-value is listed. N.A. – not applicable (this statistical test was not applicable to this data set). N.D. – not determined (this statistical test could not be performed, generally due to a lack of the number of samples within a group or all data was in a single group).

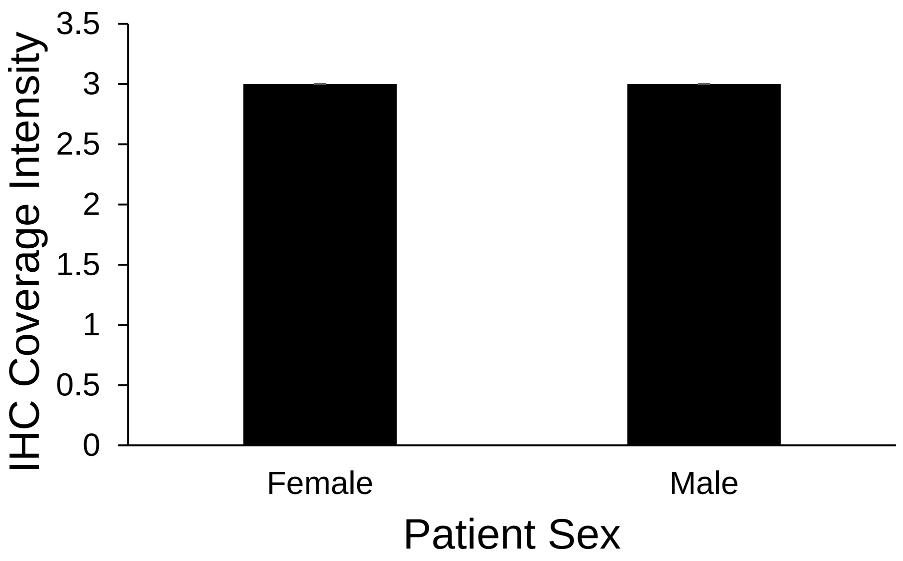

Spearman Analysis	
ρ (rho) =	-
t =	-
p-value =	-

	n	Average	St. Dev	St. Error
Female	15	3.00	0.00	0.00
Male	13	3.00	0.00	0.00

t-test

p-value = 1.00

SI Figure 173. Correlation analysis of CCK2R coverage score in pancreatic cancer versus patient sex. IHC was performed on pancreatic cancer tissue sections using a monoclonal antibody raised against CCK2R. The coverage score was determined by grading the area stained on a scale of 0 to 3 and plotted (error bars represent standard error of the mean). A t-test was used to determine if there were any significant differences between groups.

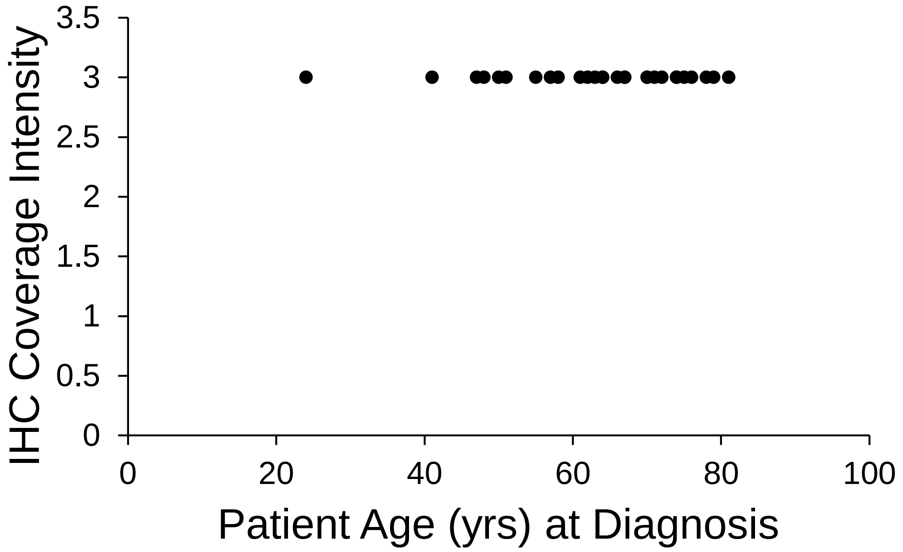

Spearman Analysis

ρ (rho) =	-
t =	-
p-value =	-

	n	Average	St. Dev	St. Error
0	0	-	-	-
1	0	-	-	-
2	0	-	-	-
3	28	63.04	13.02	2.46

1-Way Anova

	SS	df	MS	F	p
Between	-	-	-	-	-
Within	-	-	-		
Total	-	-			

SI Figure 174. Correlation analysis of CCK2R coverage score in pancreatic cancer versus patient age at diagnosis. IHC was performed on pancreatic cancer tissue sections using a monoclonal antibody raised against CCK2R. The coverage score was determined by grading the area stained on a scale of 0 to 3 and plotted. No statistical tests could be performed.

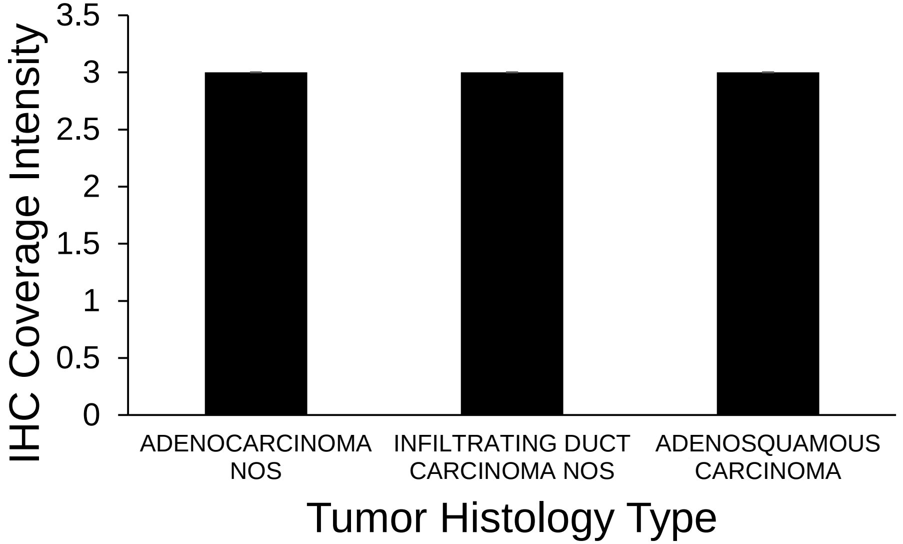

Spearman Analysis		
	ρ (rho) =	-
	t =	-
	p-value =	-

	n	Average	St. Dev	St. Error
ADENOCARCINOMA NOS	16	3.00	0.00	0.00
INFILTRATING DUCT CARCINOMA NOS	5	3.00	0.00	0.00
ADENOSQUAMOUS CARCINOMA	2	3.00	-	-

t-test

p-value = 1.0000

SI Figure 175. Correlation analysis of CCK2R coverage score in pancreatic cancer versus type of primary tumor. IHC was performed on pancreatic cancer tissue sections using a monoclonal antibody raised against CCK2R. The coverage score was determined by grading the area stained on a scale of 0 to 3 and plotted (error bars represent standard error of the mean). A t-test was used to determine if there were any significant differences between groups.

Spearman Analysis		
	ρ (rho) =	-
	t =	-
	p-value =	-

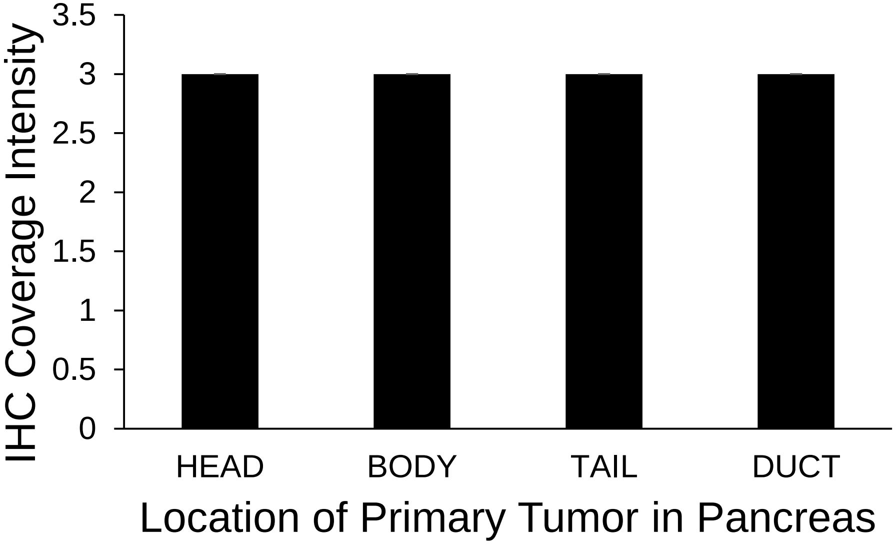

	n	Average	St. Dev	St. Error
HEAD	17	3.00	0.00	0.00
BODY	2	3.00	-	-
TAIL	3	3.00	0.00	0.00
DUCT	3	3.00	0.00	0.00

1-Way Anova

	SS	df	MS	F	p
Between	0.00	2	0.000	0.000	1.000
Within	0.00	20	0.000		
Total	0.00	22			

SI Figure 176. Correlation analysis of CCK2R coverage score in pancreatic cancer versus location of primary tumor. IHC was performed on pancreatic cancer tissue sections using a monoclonal antibody raised against CCK2R. The coverage score was determined by grading the area stained on a scale of 0 to 3 and plotted (error bars represent standard error of the mean). A 1-way ANOVA was used to determine if there were any significant differences between groups.

Spearman Analysis

ρ (rho) =	-
t =	-
p-value =	-

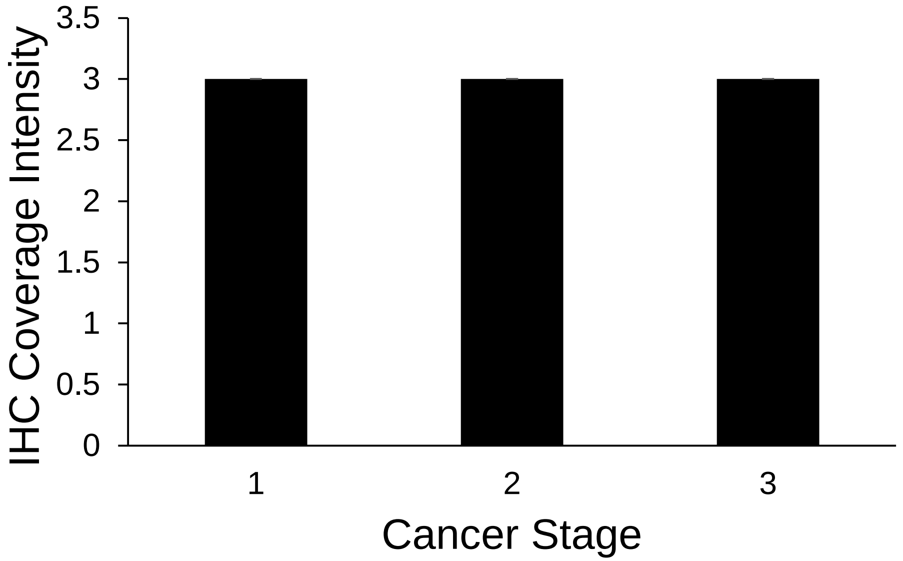

	n	Average	St. Dev	St. Error
1	3	3.00	0.00	0.00
2	18	3.00	0.00	0.00
3	7	3.00	0.00	0.00
4	0	-	-	-

1-Way Anova

	SS	df	MS	F	p
Between	0.00	2	0.000	0.000	1.000
Within	0.00	25	0.000		
Total	0.00	27			

SI Figure 177. Correlation analysis of CCK2R coverage score in pancreatic cancer versus cancer stage. IHC was performed on pancreatic cancer tissue sections using a monoclonal antibody raised against CCK2R. The coverage score was determined by grading the area stained on a scale of 0 to 3 and plotted (error bars represent standard error of the mean). A 1-way ANOVA was used to determine if there were any significant differences between groups.

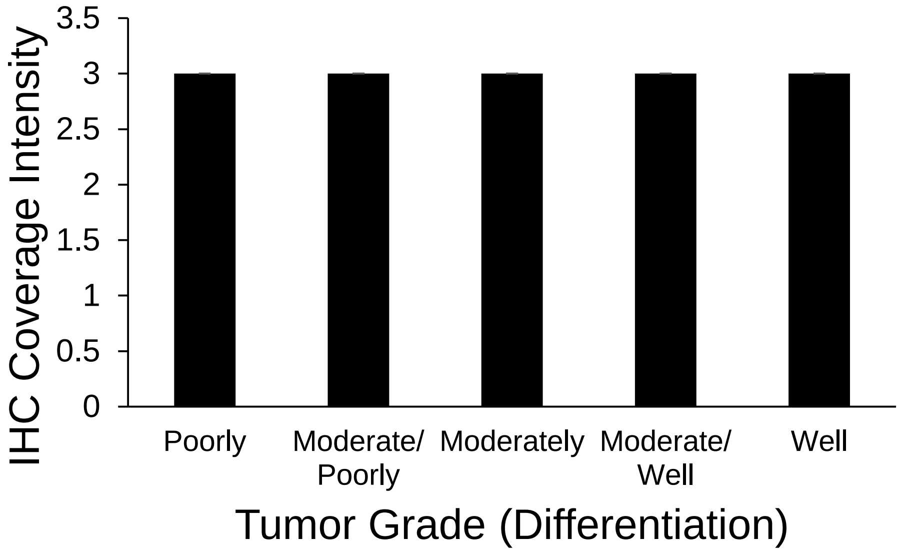

Spearman Analysis	
ρ (rho) =	-
t =	-
p-value =	-

	n	Average	St. Dev	St. Error
Poorly	1	3.00	-	-
Moderate/ Poorly	3	3.00	0.00	0.00
Moderately	13	3.00	0.00	0.00
Moderate/ Well	4	3.00	0.00	0.00
Well	5	3.00	0.00	0.00

1-Way Anova

	SS	df	MS	F	p
Between	0.00	3	0.000	0.000	1.000
Within	0.00	21	0.000		
Total	0.00	24			

SI Figure 178. Correlation analysis of CCK2R coverage score in pancreatic cancer versus primary tumor grade. IHC was performed on pancreatic cancer tissue sections using a monoclonal antibody raised against CCK2R. The coverage score was determined by grading the area stained on a scale of 0 to 3 and plotted (error bars represent standard error of the mean). A 1-way ANOVA was used to determine if there were any significant differences between groups.

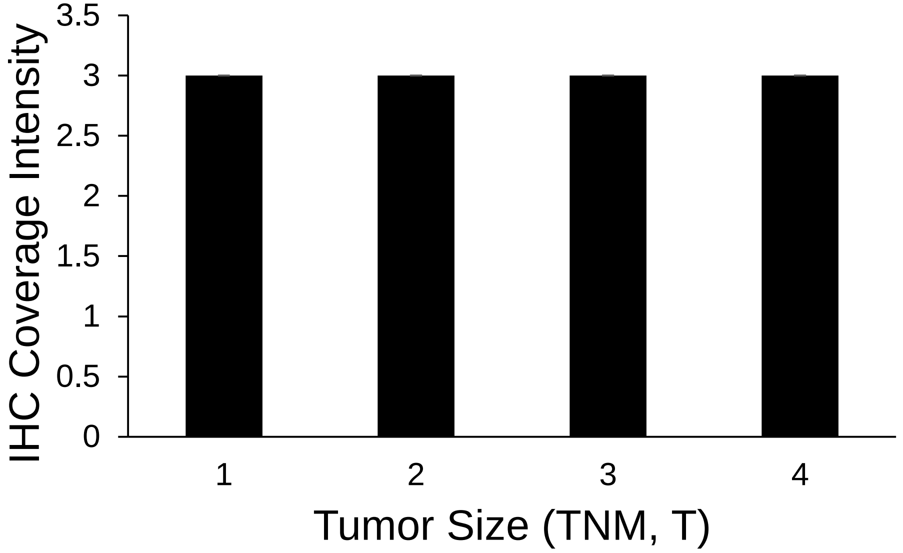

Spearman Analysis		
	ρ (rho) =	-
	t =	-
	p-value =	-

	n	Average	St. Dev	St. Error
1	2	3.00	-	-
2	4	3.00	0.00	0.00
3	20	3.00	0.00	0.00
4	2	3.00	-	-

1-Way Anova

	SS	df	MS	F	p
Between	-	-	-	-	-
Within	-	-	-		
Total	-	-			

SI Figure 179. Correlation analysis of CCK2R coverage score in pancreatic cancer versus primary tumor size. IHC was performed on pancreatic cancer tissue sections using a monoclonal antibody raised against CCK2R. The coverage score was determined by grading the area stained on a scale of 0 to 3 and plotted (error bars represent standard error of the mean). No statistical tests could be performed.

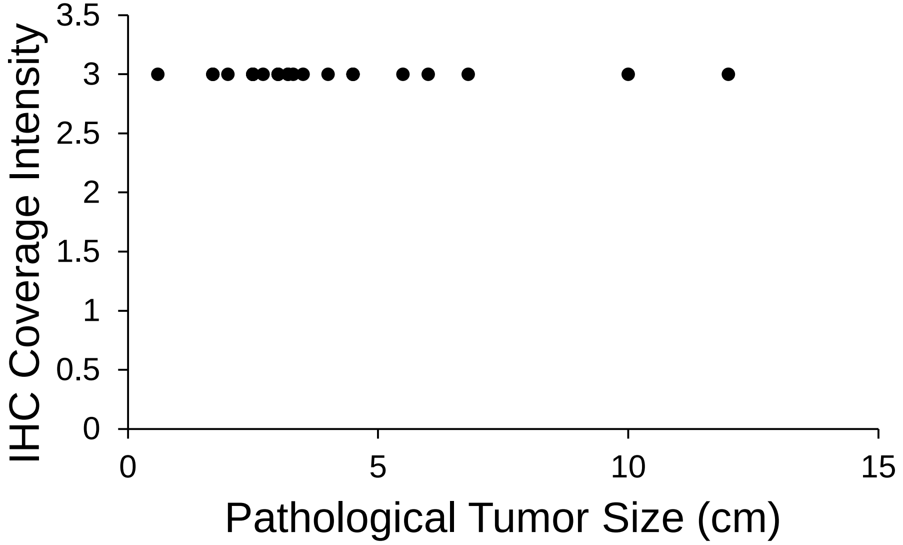

Spearman Analysis		
	ρ (rho) =	-
	t =	-
	p-value =	-

	n	Average	St. Dev	St. Error
0	0	-	-	-
1	0	-	-	-
2	0	-	-	-
3	28	3.76	2.47	0.47

1-Way Anova

	SS	df	MS	F	p
Between	-	-	-	-	-
Within	-	-	-		
Total	-	-			

SI Figure 180. Correlation analysis of CCK2R coverage score in pancreatic cancer versus size of primary tumor (length of longest side). IHC was performed on pancreatic cancer tissue sections using a monoclonal antibody raised against CCK2R. The coverage score was determined by grading the area stained on a scale of 0 to 3 and plotted. No statistical tests could be performed.

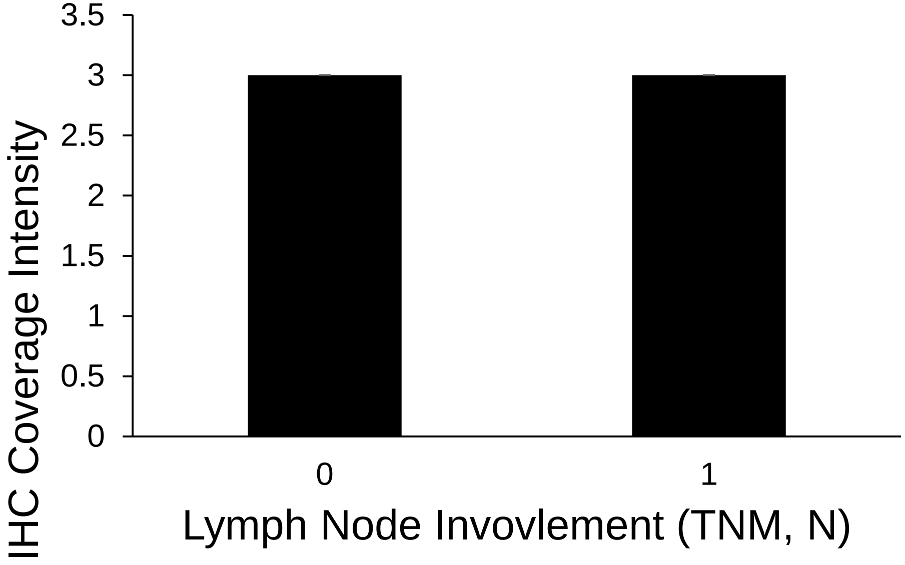

Spearman Analysis ρ (rho) = -
t = -
p-value = -

	n	Average	St. Dev	St. Error
0	11	3.00	0.00	0.00
1	17	3.00	0.00	0.00

t-test

p-value = 1.00

SI Figure 181. Correlation analysis of CCK2R coverage score in pancreatic cancer versus lymph node involvement. IHC was performed on pancreatic cancer tissue sections using a monoclonal antibody raised against CCK2R. The coverage score was determined by grading the area stained on a scale of 0 to 3 and plotted (error bars represent standard error of the mean). A t-test was used to determine if there were any significant differences between groups.

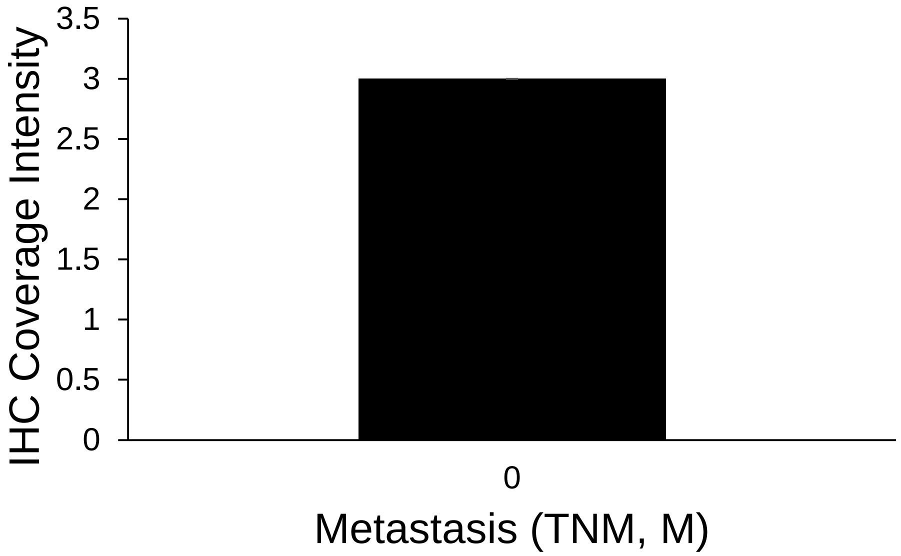

Spearman Analysis ρ (rho) = -

t = -

p-value = -

	n	Average	St. Dev	St. Error
0	28	3.00	0.00	0.00

t-test

p-value = -

SI Figure 182. Correlation analysis of CCK2R coverage score in pancreatic cancer versus metastases. IHC was performed on pancreatic cancer tissue sections using a monoclonal antibody raised against CCK2R. The coverage score was determined by grading the area stained on a scale of 0 to 3 and plotted (error bars represent standard error of the mean). No statistical tests could be performed.

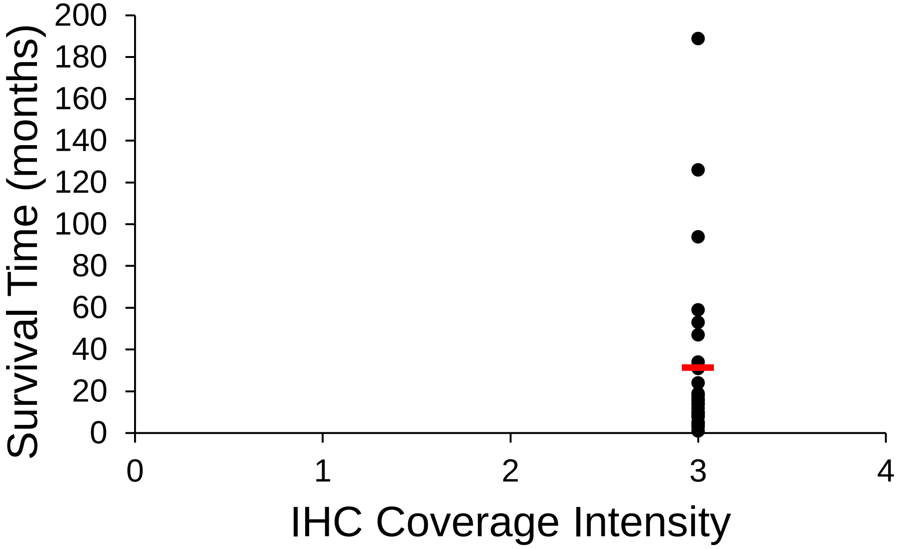

Spearman Analysis		
	ρ (rho) =	-
	t =	-
	p-value =	-

	n	Average	St. Dev	St. Error
0	0	-	-	-
1	0	-	-	-
2	0	-	-	-
3	28	31.32	42.01	7.94

1-Way Anova

	SS	df	MS	F	p
Between	-	-	-	-	-
Within	-	-	-		
Total	-	-			

SI Figure 183. Correlation analysis of CCK2R coverage score in pancreatic cancer versus survival time after diagnosis. IHC was performed on pancreatic cancer tissue sections using a monoclonal antibody raised against CCK2R. The coverage score was determined by grading the area stained on a scale of 0 to 3 and plotted (red bars represent population mean). No statistical tests could be performed.

Pancreatic Cancer
Total Staining Score Correlations

Pancreatic Cancer

	n	Average	St. Dev	St. Error
Total Score	55	7.96	1.66	0.22

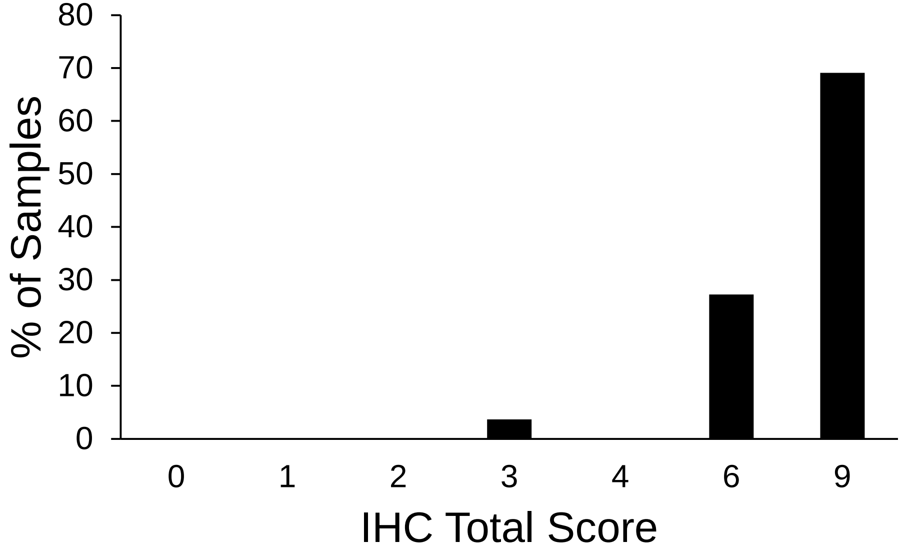

	Total Staining Score						
	0	1	2	3	4	6	9
n	0	0	0	2	0	15	38
%	0.00	0.00	0.00	3.64	0.00	27.27	69.09

Normal Pancreas

	n	Average	St. Dev	St. Error
Total Score	66	7.55	1.99	0.24

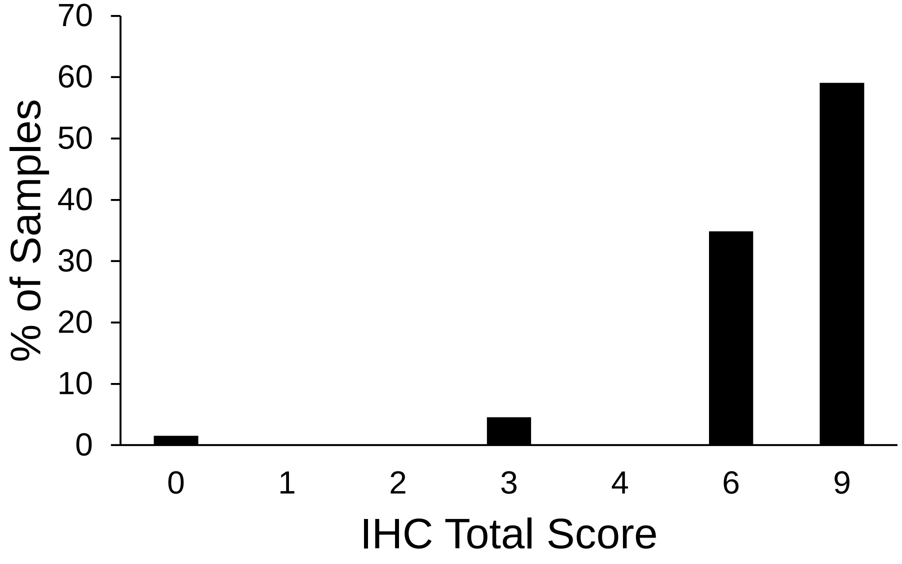

	Total Staining Score						
	0	1	2	3	4	6	9
n	1	0	0	3	0	23	39
%	1.52	0.00	0.00	4.55	0.00	34.85	59.09

SI Figure 184. CCK2R Total Staining Score for cancer and normal tissue from the pancreas. IHC was performed on tissue sections using a monoclonal antibody raised against CCK2R. The staining intensity and coverage score was multiplied to obtain the total staining score.

Pancreatic Cancer - Total Staining Score

	Sex	Age at Diagnosis	Primary Tumor Type	Primary Tumor Site	Stage	Grade	Tumor Size (TNM, T)	Tumor Size (longest dimension)	Lymph Node Involvement (TNM, N)	Metastatic (TNM, M)	Metastatic Site	Survival after Diagnosis	Survival after Stage IV Diagnosis
**Spearman Correlation**	N.A.	No 0.719	N.A.	N.A.	No 0.415	No 0.2068	**Yes 0.045**	**Yes 0.009**	No 0.809	N.D.	N.A.	No 0.926	N.D.
**ANOVA/t-test**	No 0.476	N.D.	No 0.617	No 0.685	**Yes 0.003**	No 0.59	N.D.	No 0.500	No 0.775	N.D.	N.D.	N.D.	N.D.

SI Figure 185. Total staining score correlation summary of CCK2R in pancreatic cancer. IHC was performed on pancreatic tumor tissue sections using a monoclonal antibody raised against CCK2R. The total staining score was compared against available patient data. If appropriate, a spearman analysis was used to determine if any significant correlation exists while a 1-way ANOVA or t-test was used to determine if a significant difference exists between groups. Whether the test was statistically significant and the p-value is listed. N.A. – not applicable (this statistical test was not applicable to this data set). N.D. – not determined (this statistical test could not be performed, generally due to a lack of the number of samples within a group or all data was in a single group).

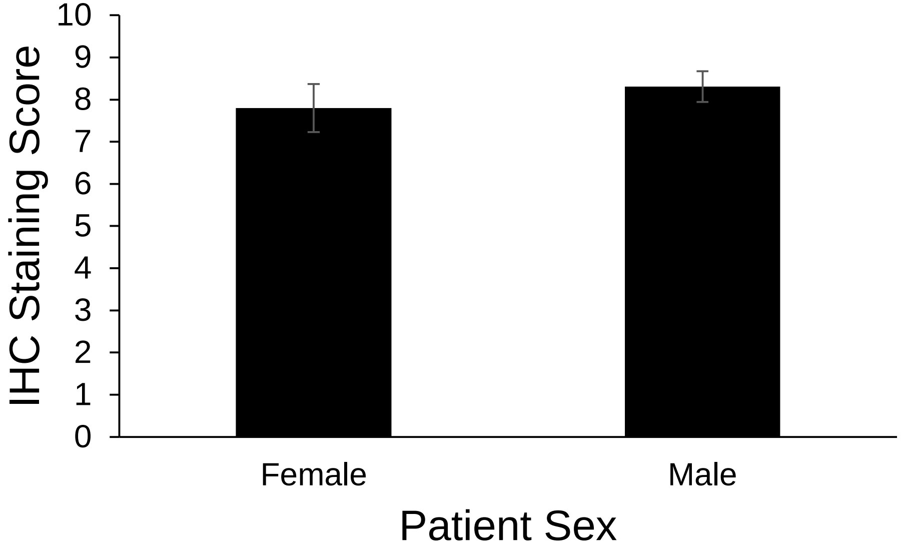

Spearman Analysis

ρ (rho) =	-
t =	-
p-value =	-

	n	Average	St. Dev	St. Error
Female	15	7.80	2.21	0.57
Male	13	8.31	1.32	0.36

t-test

p-value = 0.476

SI Figure 186. Correlation analysis of CCK2R total staining score in pancreatic cancer versus patient sex. IHC was performed on pancreatic cancer tissue sections using a monoclonal antibody raised against CCK2R. The total staining score was derived by multiplying the staining intensity with the coverage score and plotted (error bars represent standard error of the mean). A t-test was used to determine if there were any significant differences between groups.

Spearman Analysis

ρ (rho) =	-0.0711
t =	-0.3634
p-value =	0.7191

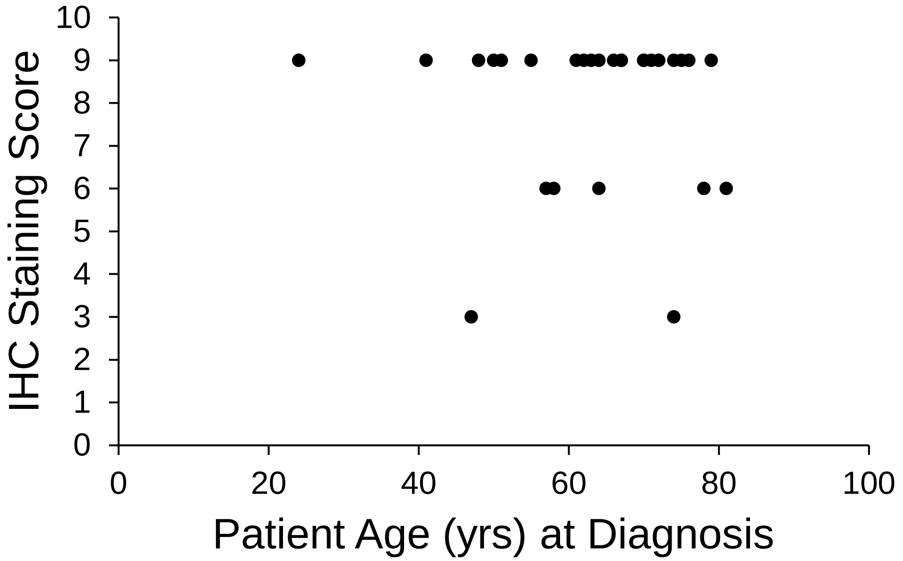

	n	Average	St. Dev	St. Error
0	0	-	-	-
1	0	-	-	-
2	0	-	-	-
3	2	60.50	19.09	13.50
4	0	-	-	-
6	5	67.60	11.24	5.03
9	21	62.19	13.37	2.92

1-Way Anova

	SS	df	MS	F	p
Between	-	-	-	-	-
Within	-	-	-		
Total	-	-			

SI Figure 187. Correlation analysis of CCK2R total staining score in pancreatic cancer versus patient age at diagnosis. IHC was performed on pancreatic cancer tissue sections using a monoclonal antibody raised against CCK2R. The total staining score was derived by multiplying the staining intensity with the coverage score and plotted. A Spearman analysis was used to determine if there was a statistically significant correlation.

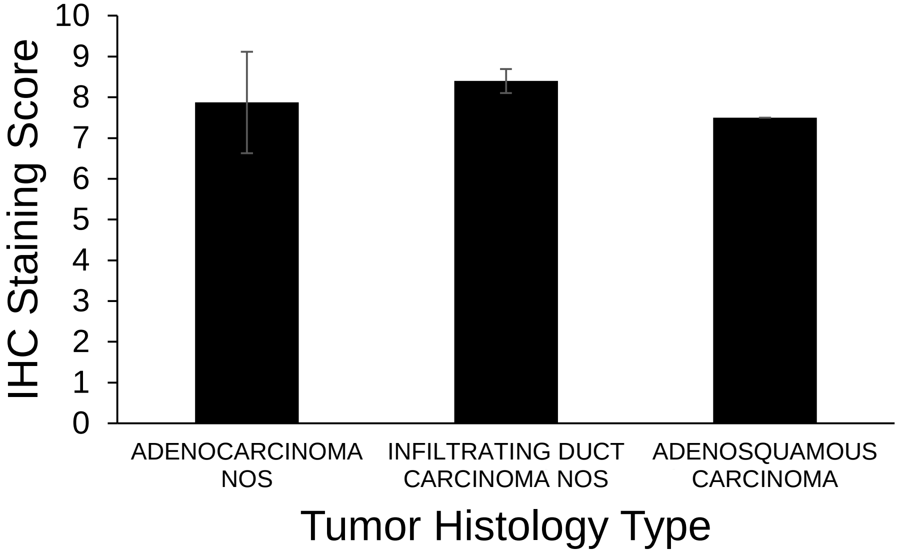

Spearman Analysis

ρ (rho) =	-
t =	-
p-value =	-

	n	Average	St. Dev	St. Error
ADENOCARCINOMA NOS	16	7.88	2.16	1.24
INFILTRATING DUCT CARCINOMA NOS	5	8.40	1.34	0.29
ADENOSQUAMOUS CARCINOMA	2	7.50	-	-

t-test

p-value = 0.6165

SI Figure 188. Correlation analysis of CCK2R total staining score in pancreatic cancer versus type of primary tumor. IHC was performed on pancreatic cancer tissue sections using a monoclonal antibody raised against CCK2R. The total staining score was derived by multiplying the staining intensity with the coverage score and plotted (error bars represent standard error of the mean). A t-test was used to determine if there were any significant differences between groups.

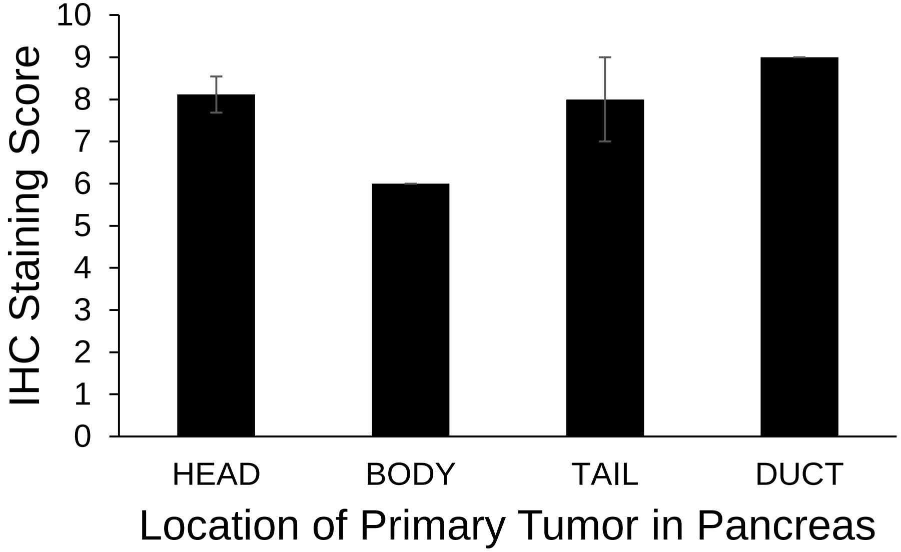

Spearman Analys		
	ρ (rho) =	-
	t =	-
	p-value =	-

	n	Average	St. Dev	St. Error
HEAD	17	8.12	1.76	0.43
BODY	2	6.00	-	-
TAIL	3	8.00	1.73	1.00
DUCT	3	9	0	0

1-Way Anova

	SS	df	MS	F	p
Between	2.14	2	1.070	0.385	0.685
Within	55.55	20	2.777		
Total	57.69	22			

SI Figure 189. Correlation analysis of CCK2R total staining score in pancreatic cancer versus location of primary tumor. IHC was performed on pancreatic cancer tissue sections using a monoclonal antibody raised against CCK2R. The total staining score was derived by multiplying the staining intensity with the coverage score and plotted (error bars represent standard error of the mean). A 1-way ANOVA was used to determine if there were any significant differences between groups.

Spearman Analysis ρ (rho) = -0.1604
t = -0.8287
p-value = 0.4146

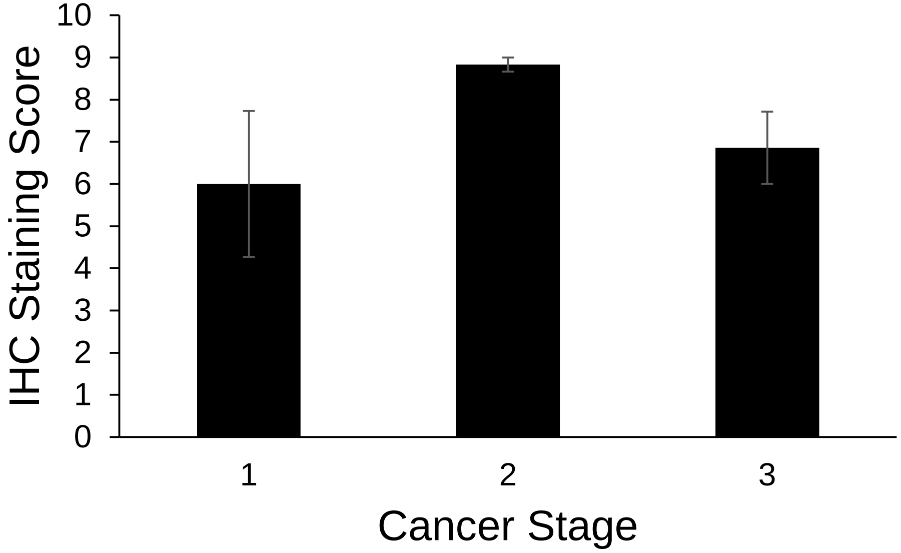

	n	Average	St. Dev	St. Error
1	3	6.00	3.00	1.73
2	18	8.83	0.71	0.17
3	7	6.86	2.27	0.86
4	0	-	-	-

1-Way Anova

	SS	df	MS	F	p
Between	33.46	2	16.73	7.277	**0.003**
Within	57.49	25	2.30		
Total	90.95	27			

Tukey-Kramer (p-value)

	1	2	3
1	-	**0.0161**	0.683
2		-	**0.019**
3			-

SI Figure 190. Correlation analysis of CCK2R total staining score in pancreatic cancer versus cancer stage. IHC was performed on pancreatic cancer tissue sections using a monoclonal antibody raised against CCK2R. The total staining score was derived by multiplying the staining intensity with the coverage score and plotted (error bars represent standard error of the mean). A Spearman analysis was used to determine if there was a statistically significant correlation and a 1-way ANOVA followed by a Tukey-Kramer ad hoc analysis was used to determine if there were any significant differences between groups.

Spearman Analysis		
	ρ (rho) =	0.2558
	t =	1.2961
	p-value =	0.2068

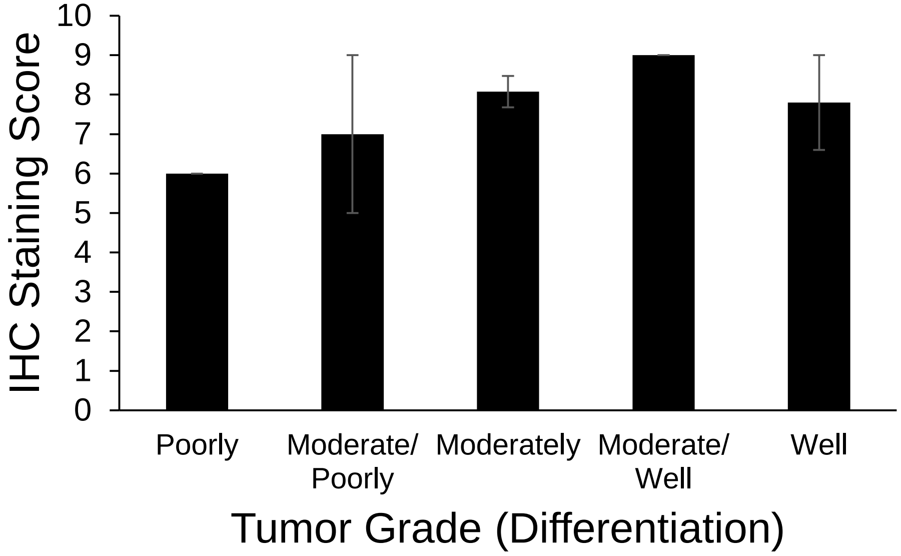

	n	Average	St. Dev	St. Error
Poorly	1	6.00	-	-
Moderate/ Poorly	3	7.00	3.46	2.00
Moderately	13	8.08	1.44	0.40
Moderate/ Well	4	9.00	0.00	0.00
Well	5	7.80	2.68	1.20

1-Way Anova

	SS	df	MS	F	p
Between	7.24	3	2.413	0.653	0.590
Within	77.56	21	3.693		
Total	84.80	24			

SI Figure 191. Correlation analysis of CCK2R total staining score in pancreatic cancer versus primary tumor grade. IHC was performed on pancreatic cancer tissue sections using a monoclonal antibody raised against CCK2R. The total staining score was derived by multiplying the staining intensity with the coverage score and plotted (error bars represent standard error of the mean). A Spearman analysis was used to determine if there was a statistically significant correlation and a 1-way ANOVA was used to determine if there were any significant differences between groups.

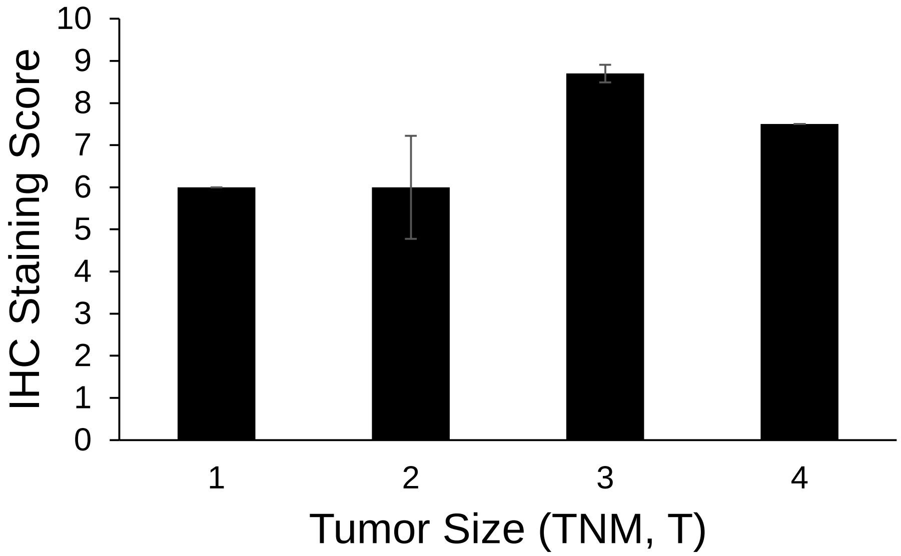

Spearman Analysis		
	ρ (rho) =	0.3805
	t =	2.0979
	p-value =	**0.0454**

	n	Average	St. Dev	St. Error
1	2	6.00	-	-
2	4	6.00	2.45	1.22
3	20	8.70	0.92	0.21
4	2	7.50	-	-

1-Way Anova

	SS	df	MS	F	p
Between	-	-	-	-	-
Within	-	-	-		
Total	-	-			

SI Figure 192. Correlation analysis of CCK2R total staining score in pancreatic cancer versus primary tumor size. IHC was performed on pancreatic cancer tissue sections using a monoclonal antibody raised against CCK2R. The total staining score was derived by multiplying the staining intensity with the coverage score and plotted (error bars represent standard error of the mean). A Spearman analysis was used to determine if there was a statistically significant correlation.

Spearman Analys	ρ (rho) =	0.4831
	t =	2.8138
	p-value =	**0.0090**

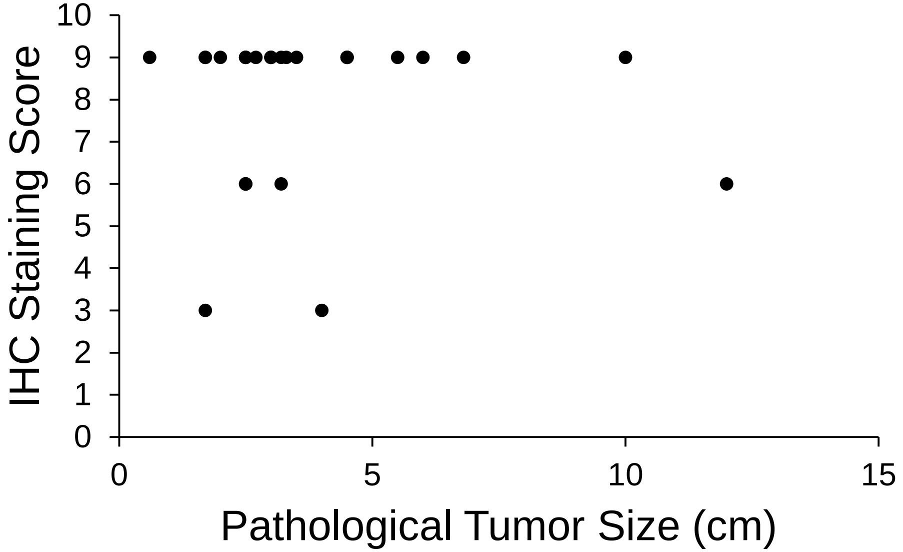

	n	Average	St. Dev	St. Error
0	0	-	-	-
1	0	-	-	-
2	0	-	-	-
3	2	2.85	1.63	1.15
4	0	-	-	-
6	5	4.54	4.18	1.87
9	21	3.67	2.09	0.46

t-test

p-value = 0.4997

SI Figure 193. Correlation analysis of CCK2R total staining score in pancreatic cancer versus size of primary tumor (length of longest side). IHC was performed on pancreatic cancer tissue sections using a monoclonal antibody raised against CCK2R. The total staining score was derived by multiplying the staining intensity with the coverage score and plotted. A Spearman analysis was used to determine if there was a statistically significant correlation and a t-test was used to determine if there were any significant differences between groups.

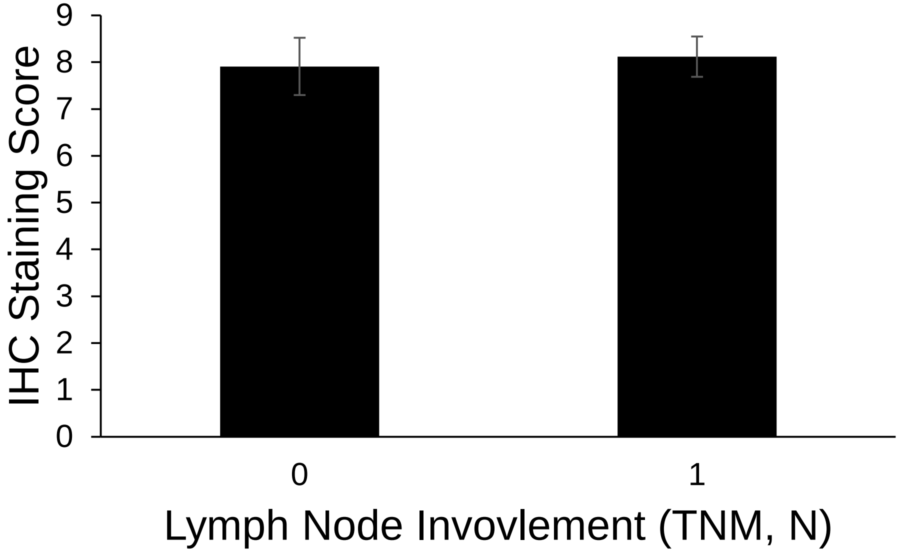

Spearman Analysis

ρ (rho) = 0.0478
t = 0.2442
p-value = 0.8089

	n	Average	St. Dev	St. Error
0	11	7.91	2.02	0.61
1	17	8.12	1.76	0.43

t-test

p-value = 0.7751

SI Figure 194. Correlation analysis of CCK2R total staining score in pancreatic cancer versus lymph node involvement. IHC was performed on pancreatic cancer tissue sections using a monoclonal antibody raised against CCK2R. The total staining score was derived by multiplying the staining intensity with the coverage score and plotted (error bars represent standard error of the mean). A Spearman analysis was used to determine if there was a statistically significant correlation and a t-test was used to determine if there were any significant differences between groups.

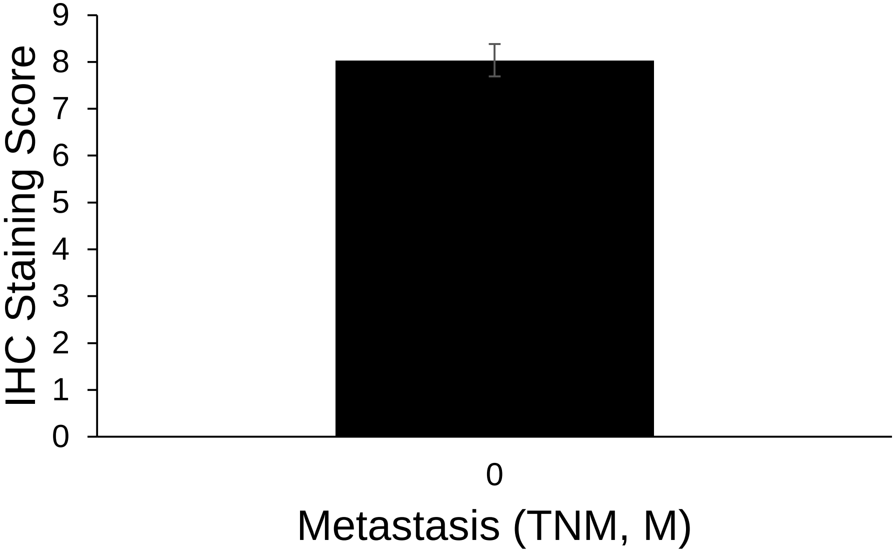

Spearman Analysis ρ (rho) = -
t = -
p-value = -

	n	Average	St. Dev	St. Error
0	28	8.04	1.84	0.35

t-test

p-value = -

SI Figure 195. Correlation analysis of CCK2R total staining score in pancreatic cancer versus metastases. IHC was performed on pancreatic cancer tissue sections using a monoclonal antibody raised against CCK2R. The total staining score was derived by multiplying the staining intensity with the coverage score and plotted (error bars represent standard error of the mean). No statistical tests could be performed.

Spearman Analysis

	ρ (rho) =	0.0185
	t =	0.0942
	p-value =	0.9257

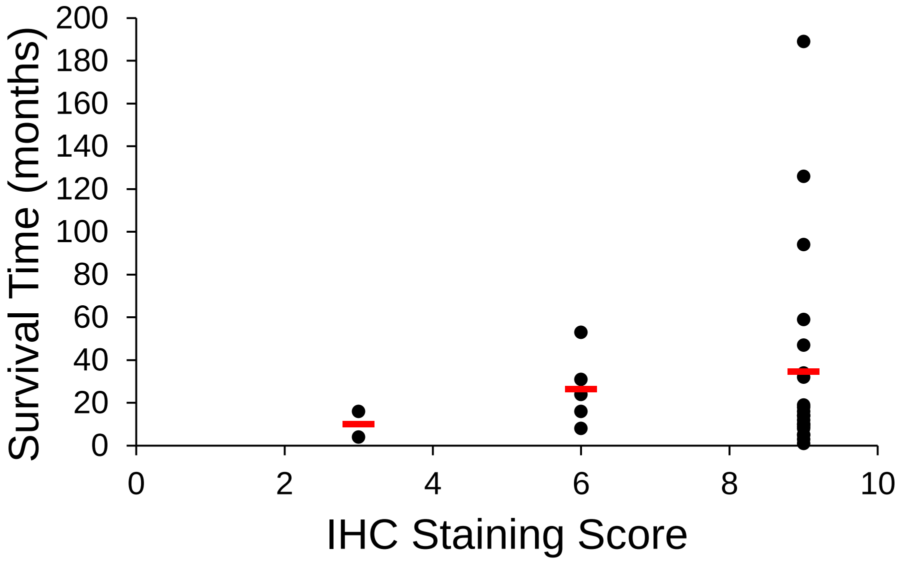

	n	Average	St. Dev	St. Error
0	0	-	-	-
1	0	-	-	-
2	0	-	-	-
3	2	10.00	-	-
4	0	-	-	-
6	5	26.40	17.18	7.69
9	21	34.52	47.52	10.37

1-Way Anova

	SS	df	MS	F	p
Between	-	-	-	-	-
Within	-	-	-		
Total	-	-			

SI Figure 196. Correlation analysis of CCK2R total staining score in pancreatic cancer versus survival time after diagnosis. IHC was performed on pancreatic cancer tissue sections using a monoclonal antibody raised against CCK2R. The total staining score was derived by multiplying the staining intensity with the coverage score and plotted (red bars represent population mean). A Spearman analysis was used to determine if there was a statistically significant correlation.

Thyroid Cancer

Thyroid Cancer Images

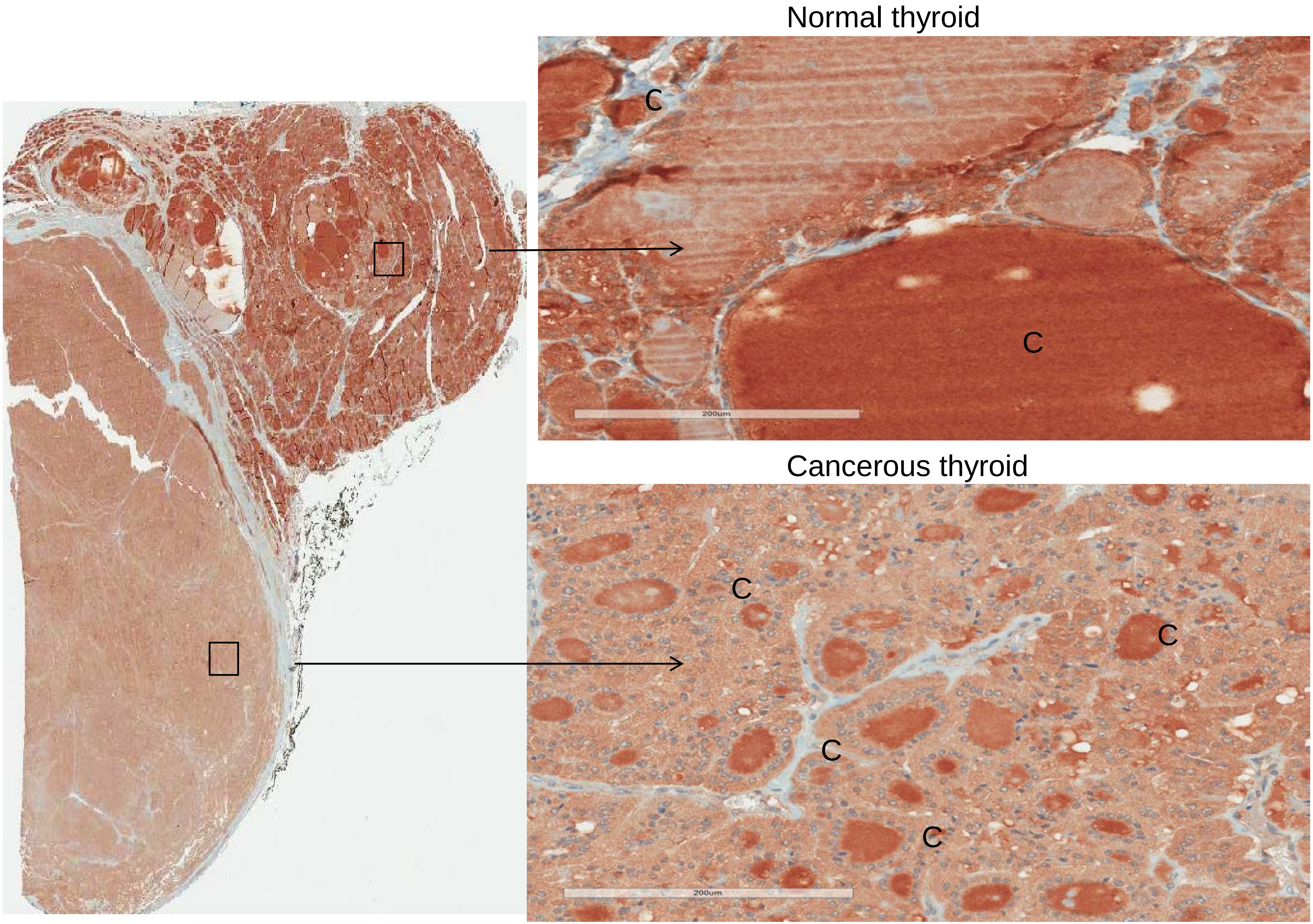

SI Figure 197. Example image of stained tissue from thyroid. IHC was performed on tissue sections using a monoclonal antibody raised against CCK2R. C denotes colloid staining in both normal and cancerous tissue. The staining of colloid did not factor into the scoring.

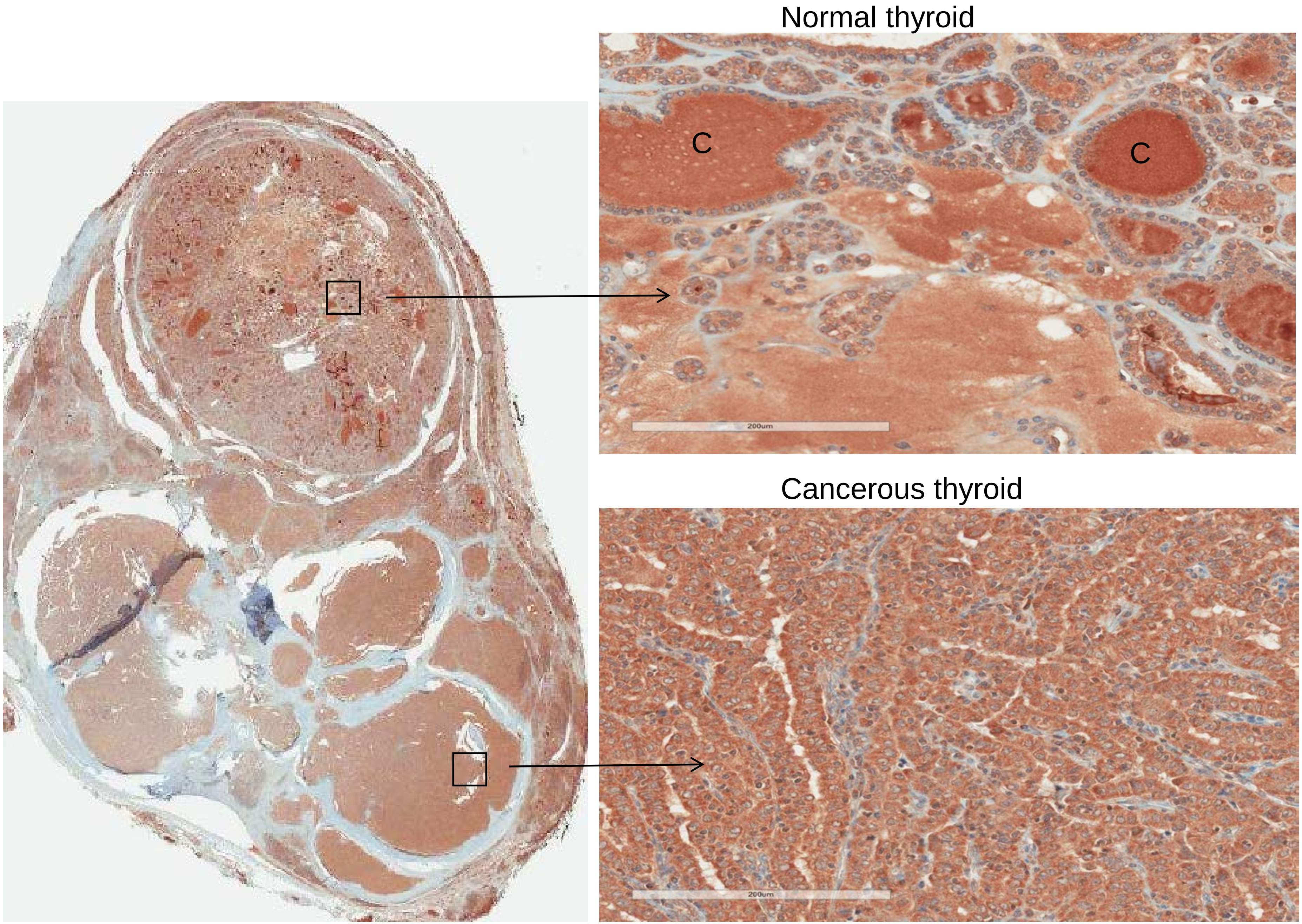

SI Figure 198. Example image of stained tissue from thyroid. IHC was performed on tissue sections using a monoclonal antibody raised against CCK2R. C denotes colloid staining in both normal and cancerous tissue. The staining of colloid did not factor into the scoring.

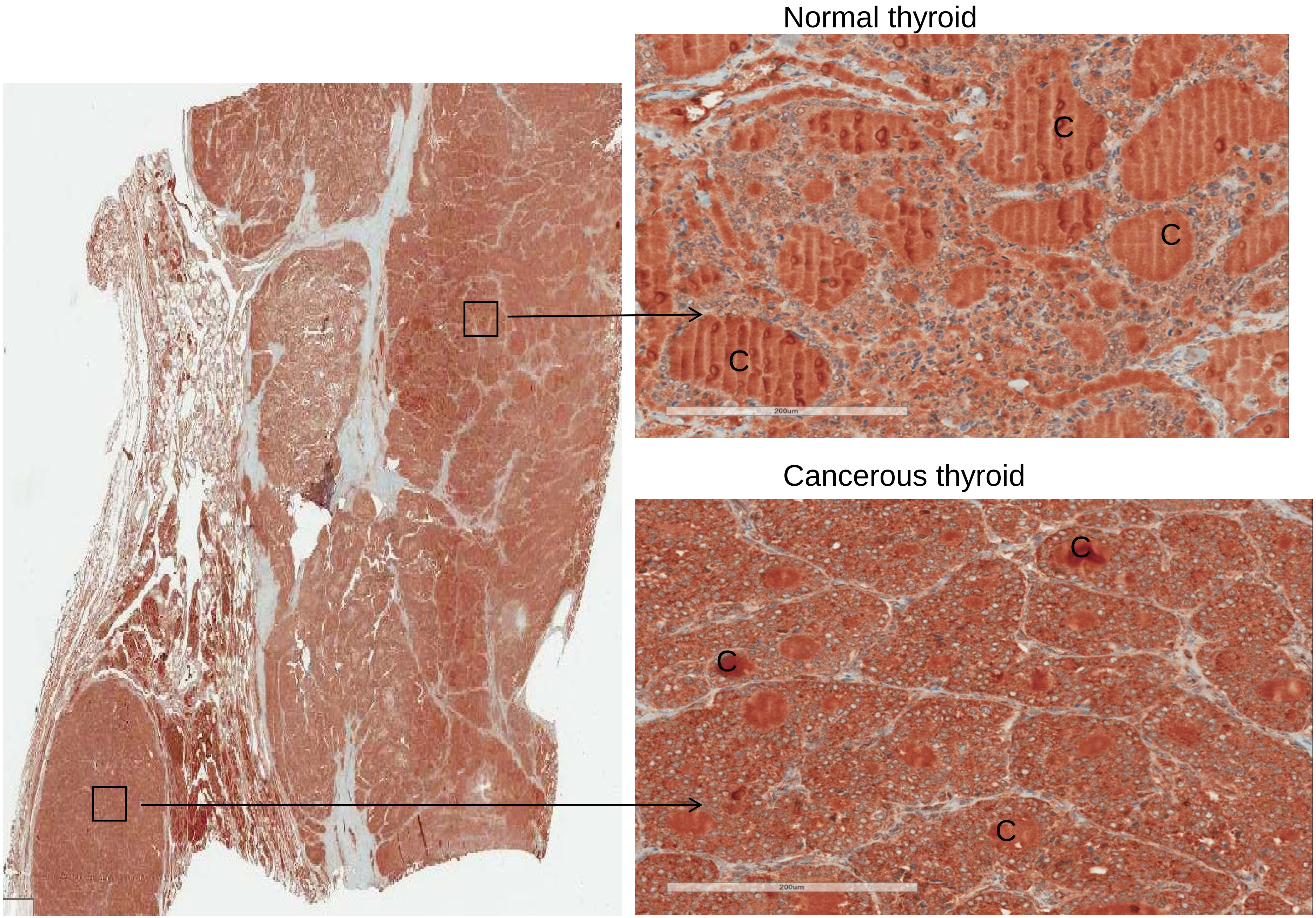

SI Figure 199. Example image of stained tissue from thyroid. IHC was performed on tissue sections using a monoclonal antibody raised against CCK2R. C denotes colloid staining in both normal and cancerous tissue. The staining of colloid did not factor into the scoring.

Thyroid Cancer
Overall Summary

Thyroid - Spearman Correlation

	Sex	Age at Diagnosis	Primary Tumor Type	Primary Tumor Site	Stage	Grade	Tumor Size (TNM, T)	Tumor Size (longest dimension)	Lymph Node Involvement (TNM, N)	Metastatic (TNM, M)	Metastatic Site	Survival after Diagnosis	Survival after Stage IV Diagnosis
**Staining Intensity**	N.A.	No 0.873	N.A.	N.A.	No 0.251	No 0.978	No 0.890	No 0.953	**Yes 0.011**	N.D.	N.A.	N.D.	N.D.
**Coverage Score**	N.A.	No 0.825	N.A.	N.A.	No 0.216	No 1.00	No 0.154	No 0.245	No 0.361	N.D.	N.A.	N.D.	N.D.
**Total Staining Score**	N.A.	No 0.800	N.A.	N.A.	No 0.380	No 0.978	No 0.919	No 0.811	**Yes 0.010**	N.D.	N.A.	N.D.	N.D.

Thyroid - Spearman Correlation - ANOVA/t-test

	Sex	Age at Diagnosis	Primary Tumor Type	Primary Tumor Site	Stage	Grade	Tumor Size (TNM, T)	Tumor Size (longest dimension)	Lymph Node Involvement (TNM, N)	Metastatic (TNM, M)	Metastatic Site	Survival after Diagnosis	Survival after Stage IV Diagnosis
**Staining Intensity**	No 0.570	No 0.842	No 0.681	N.D.	No 0.312	No 1.00	No 0.805	No 0.776	**Yes 0.010**	N.D.	N.D.	N.D.	N.D.
**Coverage Score**	No 0.185	N.D.	**Yes 0.036**	N.D.	No 0.429	No 1.00	No 0.298	N.D.	No 0.362	N.D.	N.D.	N.D.	N.D.
**Total Staining Score**	No 0.670	No 0.843	No 0.684	N.D.	No 0.369	No 1.00	No 0.764	No 0.992	**Yes 0.009**	N.D.	N.D.	N.D.	N.D.

SI Figure 200. Correlation summary of CCK2R in thyroid cancer. IHC was performed on thyroid tumor tissue sections using a monoclonal antibody raised against CCK2R. The staining intensity, coverage score and total staining score were compared against available patient data. If appropriate, a spearman analysis was used to determine if any significant correlation exists while a 1-way ANOVA or t-test was used to determine if a significant difference exists between groups. Whether the test was statistically significant and the p-value is listed. N.A. – not applicable (this statistical test was not applicable to this data set). N.D. – not determined (this statistical test could not be performed, generally due to a lack of the number of samples within a group or all data was in a single group).

Thyroid Cancer
Staining Intensity

Thyroid Cancer

	n	Average	St. Dev	St. Error
Staining Intensity	27	1.59	0.64	0.12

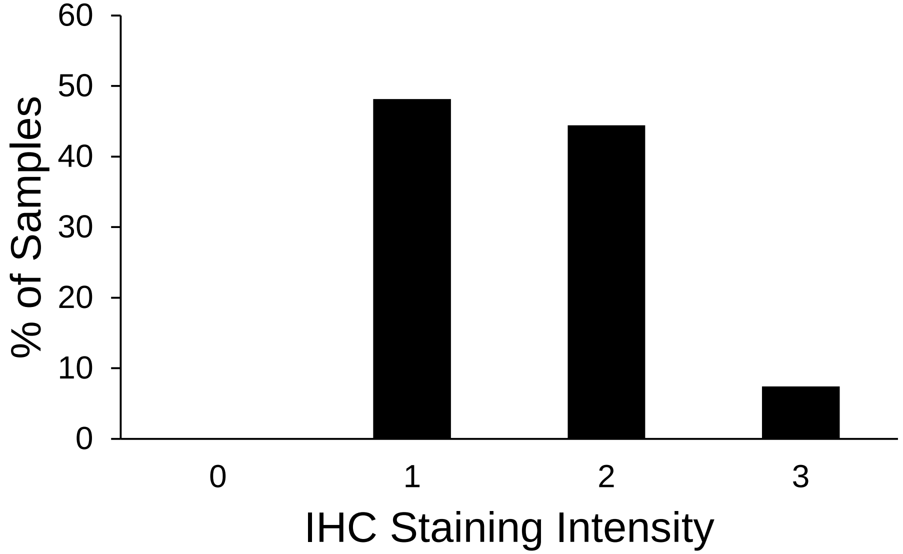

	Staining Intensity			
	0	1	2	3
n	0	13	12	2
%	0.00	48.15	44.44	7.41

Normal Thyroid

	n	Average	St. Dev	St. Error
Staining Intensity	28	1.07	0.26	0.05

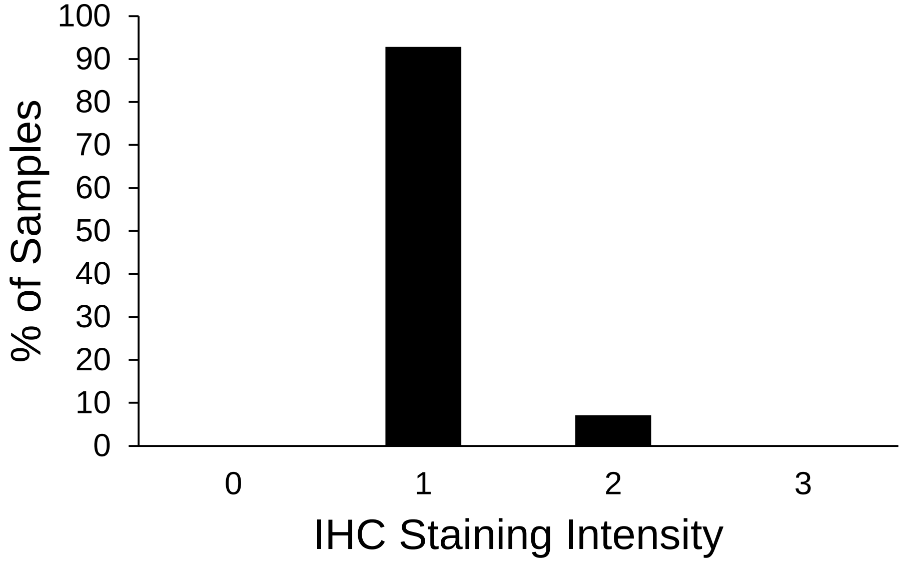

	Staining Intensity			
	0	1	2	3
n	0	26	2	0
%	0.00	92.86	7.14	0.00

SI Figure 201. CCK2R Staining Intensity for cancer and normal tissue from the thyroid. IHC was performed on tissue sections using a monoclonal antibody raised against CCK2R. The intensity of staining was graded on a scale of 0 to 3 and plotted.

Thyroid Cancer - Staining Intensity

	Sex	Age at Diagnosis	Primary Tumor Type	Primary Tumor Site	Stage	Grade	Tumor Size (TNM, T)	Tumor Size (longest dimension)	Lymph Node Involvement (TNM, N)	Metastatic (TNM, M)	Metastatic Site	Survival after Diagnosis	Survival after Stage IV Diagnosis
**Spearman Correlation**	N.A.	No 0.873	N.A.	N.A.	No 0.251	No 0.978	No 0.890	No 0.953	**Yes 0.011**	N.D.	N.A.	N.D.	N.D.
**ANOVA/t-test**	No 0.570	No 0.842	No 0.681	N.D.	No 0.312	No 1.00	No 0.805	No 0.776	**Yes 0.010**	N.D.	N.D.	N.D.	N.D.

SI Figure 202. Staining intensity correlation summary of CCK2R in thyroid cancer. IHC was performed on thyroid tumor tissue sections using a monoclonal antibody raised against CCK2R. The staining intensity was compared against available patient data. If appropriate, a spearman analysis was used to determine if any significant correlation exists while a 1-way ANOVA or t-test was used to determine if a significant difference exists between groups. Whether the test was statistically significant and the p-value is listed. N.A. – not applicable (this statistical test was not applicable to this data set). N.D. – not determined (this statistical test could not be performed, generally due to a lack of the number of samples within a group or all data was in a single group).

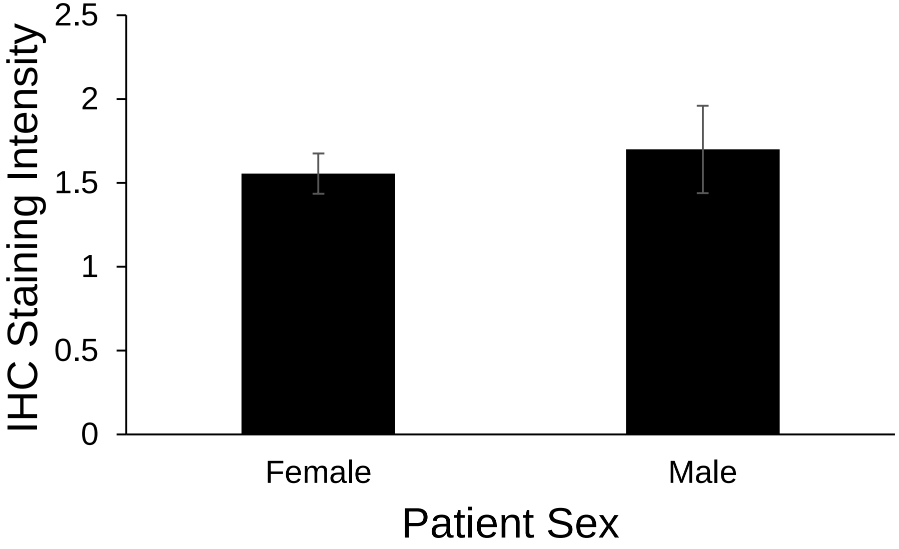

Spearman Analysis
ρ (rho) = -
t = -
p-value = -

	n	Average	St. Dev	St. Error
Female	18	1.56	0.51	0.12
Male	10	1.70	0.82	0.26

t-test

p-value = 0.5702

SI Figure 203. Correlation analysis of CCK2R staining intensity in thyroid cancer versus patient sex. IHC was performed on thyroid cancer tissue sections using a monoclonal antibody raised against CCK2R. The staining intensity was graded on a scale of 0 to 3 and plotted (error bars represent standard error of the mean). A t-test was used to determine if there were any significant differences between groups.

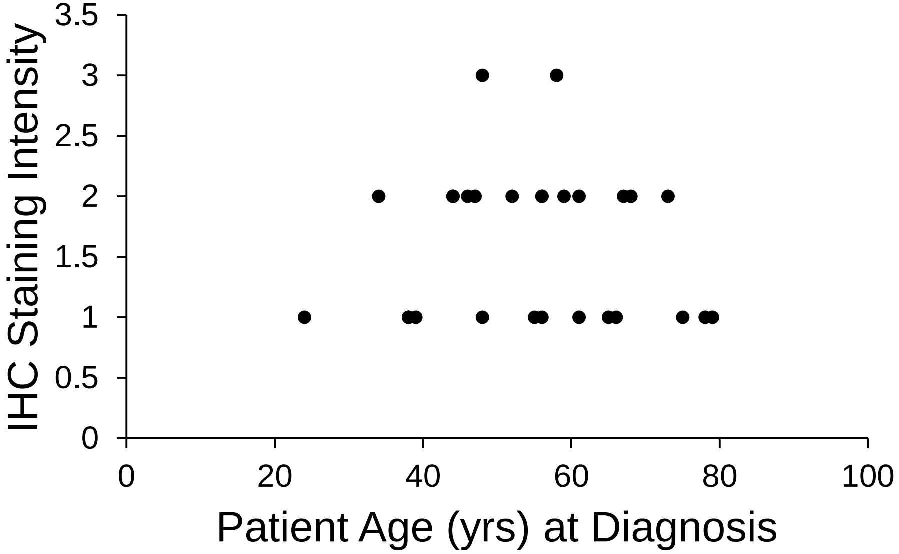

Spearman Analysis

ρ (rho) = -0.0316
t = -0.1613
p-value = 0.8731

	n	Average	St. Dev	St. Error
0	0	-	-	-
1	13	55.54	17.33	4.81
2	13	54.38	11.27	3.12
3	2	53.00	7.07	5.00

t-test

p-value = 0.8422

SI Figure 204. Correlation analysis of CCK2R staining intensity in thyroid cancer versus patient age at diagnosis. IHC was performed on thyroid cancer tissue sections using a monoclonal antibody raised against CCK2R. The staining intensity was graded on a scale of 0 to 3 and plotted. A Spearman analysis was used to determine if there was a statistically significant correlation and a t-test was used to determine if there were any significant differences between groups.

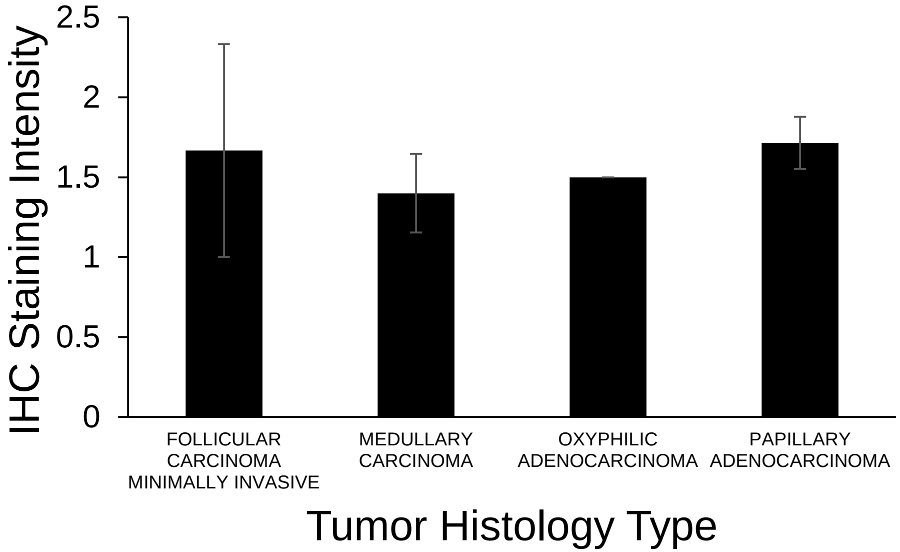

Spearman Analysis		
	ρ (rho) =	-
	t =	-
	p-value =	-

	n	Average	St. Dev	St. Error
FOLLICULAR CARCINOMA MINIMALLY INVASIVE	3	1.67	1.15	0.67
MEDULLARY CARCINOMA	5	1.40	0.55	0.24
OXYPHILIC ADENOCARCINOMA	2	1.50	-	-
PAPILLARY ADENOCARCINOMA	14	1.71	0.61	0.16

1-Way Anova

	SS	df	MS	F	p
Between	0.36	2	0.179	0.392	0.681
Within	8.69	19	0.457		
Total	9.05	21			

SI Figure 205. Correlation analysis of CCK2R staining intensity in thyroid cancer versus type of primary tumor. IHC was performed on thyroid cancer tissue sections using a monoclonal antibody raised against CCK2R. The staining intensity was graded on a scale of 0 to 3 and plotted (error bars represent standard error of the mean). A 1-way ANOVA was used to determine if there were any significant differences between groups.

Spearman Analysis		
	ρ (rho) =	0.2285
	t =	1.1735
	p-value =	0.2512

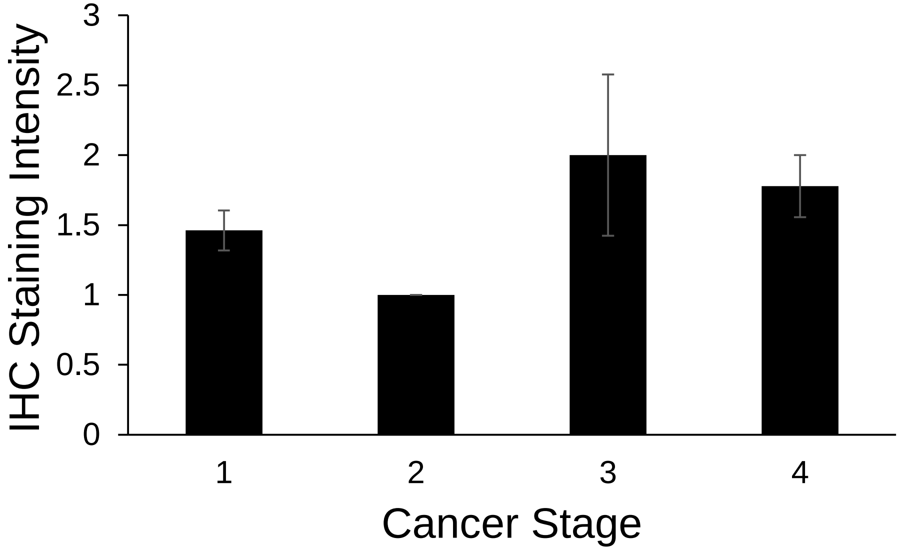

	n	Average	St. Dev	St. Error
1	13	1.46	0.52	0.14
2	2	1.00	-	-
3	3	2.00	1.00	0.58
4	9	1.78	0.67	0.22

1-Way Anova

	SS	df	MS	F	p
Between	0.99	2	0.493	1.228	0.312
Within	8.84	22	0.402		
Total	9.82	24			

SI Figure 206. Correlation analysis of CCK2R staining intensity in thyroid cancer versus cancer stage. IHC was performed on thyroid cancer tissue sections using a monoclonal antibody raised against CCK2R. The staining intensity was graded on a scale of 0 to 3 and plotted (error bars represent standard error of the mean). A Spearman analysis was used to determine if there was a statistically significant correlation and a 1-way ANOVA was used to determine if there were any significant differences between groups.

Spearman Analysis

ρ (rho) = 0.0084
t = 0.0280
p-value = 0.9782

	n	Average	St. Dev	St. Error
Undifferentiated	2	1.50	-	-
Poorly	2	2.00	-	-
Moderately	6	1.67	0.82	0.33
Well	3	1.67	0.58	0.33

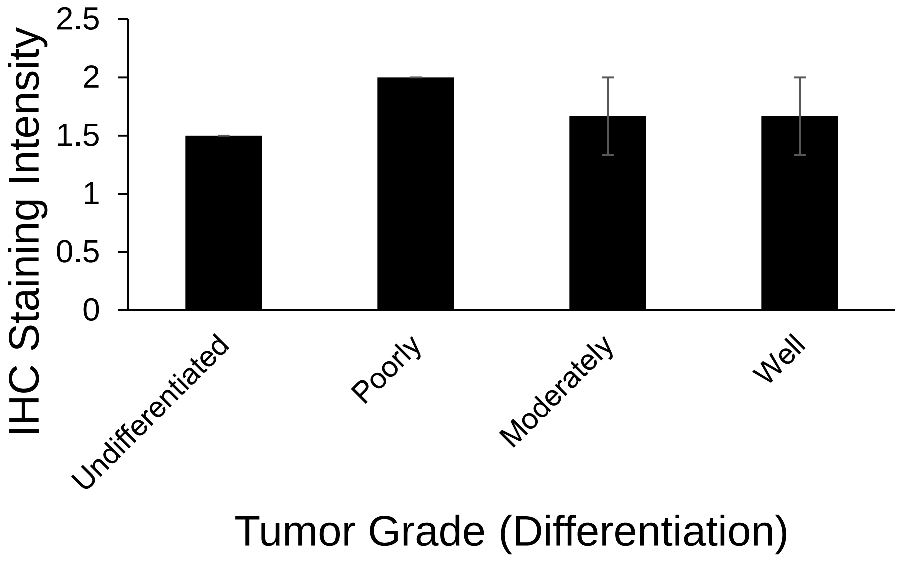

t-test

p-value = 1.0000

SI Figure 207. Correlation analysis of CCK2R staining intensity in thyroid cancer versus primary tumor grade. IHC was performed on thyroid cancer tissue sections using a monoclonal antibody raised against CCK2R. The staining intensity was graded on a scale of 0 to 3 and plotted (error bars represent standard error of the mean). A Spearman analysis was used to determine if there was a statistically significant correlation and a t-test was used to determine if there were any significant differences between groups.

Spearman Analysis		
	ρ (rho) =	0.0274
	t =	0.1398
	p-value =	0.8898

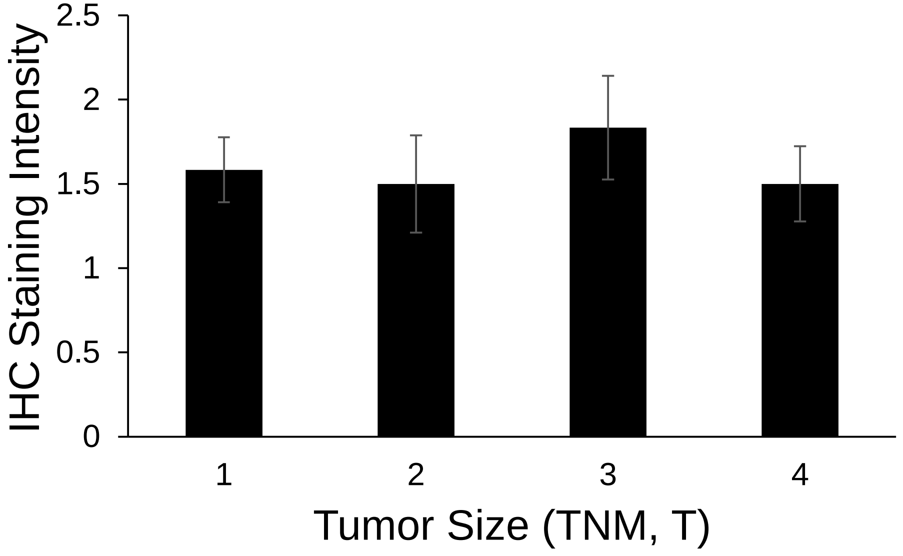

	n	Average	St. Dev	St. Error
1	12	1.58	0.67	0.19
2	4	1.50	0.58	0.29
3	6	1.83	0.75	0.31
4	6	1.50	0.55	0.22

1-Way Anova

	SS	df	MS	F	p
Between	0.42	3	0.141	0.328	0.805
Within	10.27	24	0.428		
Total	10.69	27			

SI Figure 208. Correlation analysis of CCK2R staining intensity in thyroid cancer versus primary tumor size. IHC was performed on thyroid cancer tissue sections using a monoclonal antibody raised against CCK2R. The staining intensity was graded on a scale of 0 to 3 and plotted (error bars represent standard error of the mean). A Spearman analysis was used to determine if there was a statistically significant correlation and a 1-way ANOVA was used to determine if there were any significant differences between groups.

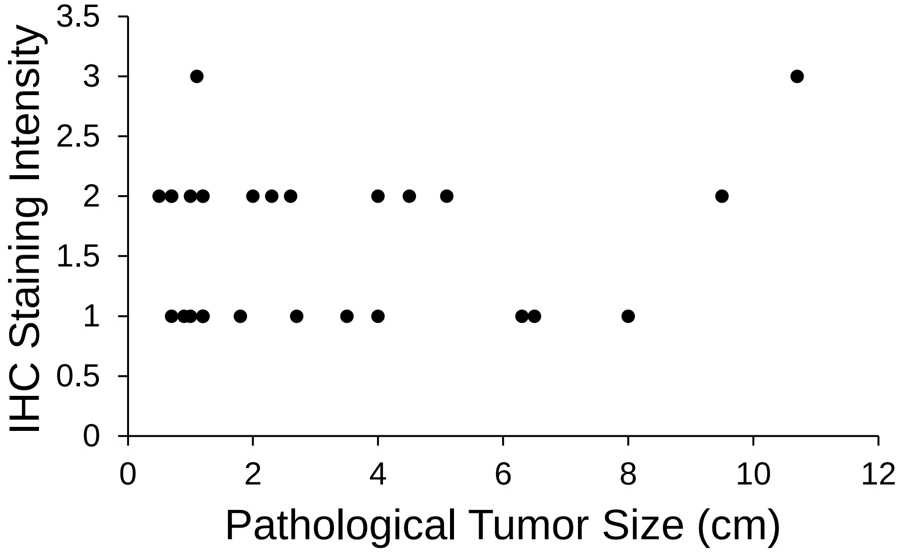

Spearman Analysis

ρ (rho) = -0.0117

t = -0.0595

p-value = 0.9530

	n	Average	St. Dev	St. Error
0	0	-	-	-
1	13	3.00	2.49	0.69
2	13	2.72	2.55	0.71
3	2	5.90	6.79	4.80

t-test

p-value = 0.7757

SI Figure 209. Correlation analysis of CCK2R staining intensity in thyroid cancer versus size of primary tumor (length of longest side). IHC was performed on thyroid cancer tissue sections using a monoclonal antibody raised against CCK2R. The staining intensity was graded on a scale of 0 to 3 and plotted. A Spearman analysis was used to determine if there was a statistically significant correlation and a t-test was used to determine if there were any significant differences between groups.

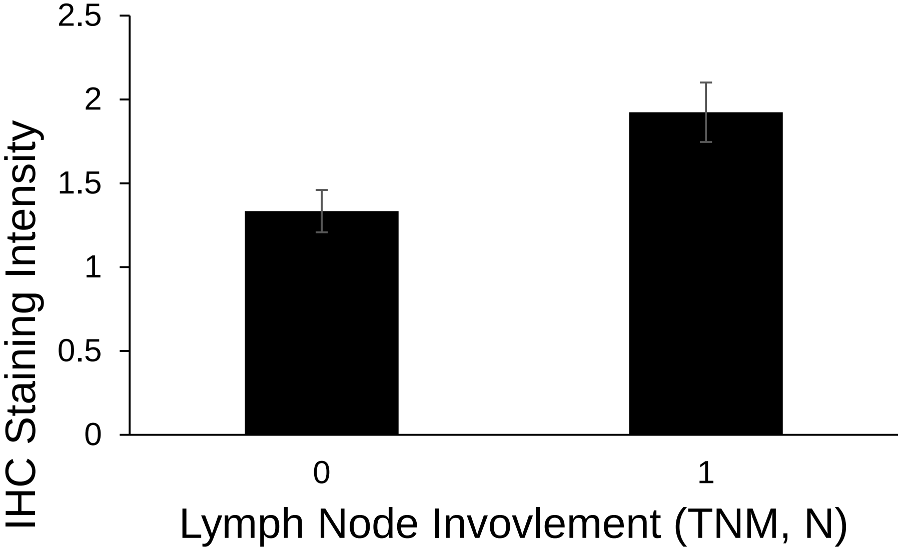

Spearman Analysis

ρ (rho) = 0.4707
t = 2.7200
p-value = **0.0113**

	n	Average	St. Dev	St. Error
0	15	1.33	0.49	0.13
1	13	1.92	0.64	0.18

t-test

p-value = **0.0104**

SI Figure 210. Correlation analysis of CCK2R staining intensity in thyroid cancer versus lymph node involvement. IHC was performed on thyroid cancer tissue sections using a monoclonal antibody raised against CCK2R. The staining intensity was graded on a scale of 0 to 3 and plotted (error bars represent standard error of the mean). A Spearman analysis was used to determine if there was a statistically significant correlation and a t-test was used to determine if there were any significant differences between groups.

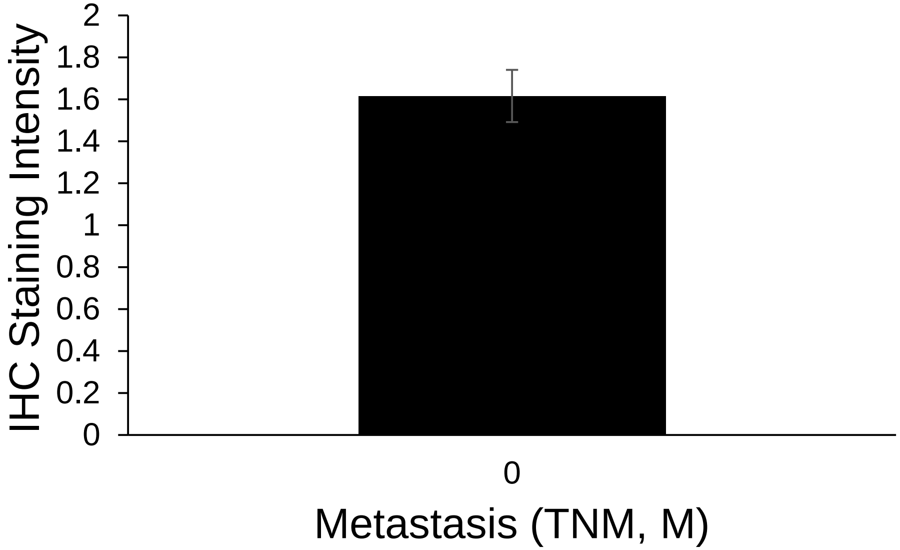

Spearman Analysis

ρ (rho) = -
t = -
p-value = -

	n	Average	St. Dev	St. Error
0	26	1.62	0.64	0.12

t-test

p-value = -

SI Figure 211. Correlation analysis of CCK2R staining intensity in thyroid cancer versus metastases. IHC was performed on thyroid cancer tissue sections using a monoclonal antibody raised against CCK2R. The staining intensity was graded on a scale of 0 to 3 and plotted (error bars represent standard error of the mean). No statistical tests could be performed.

Thyroid Cancer
Coverage Score Correlations

Thyroid Cancer

	n	Average	St. Dev	St. Error
Coverage Score	27	2.96	0.19	0.04

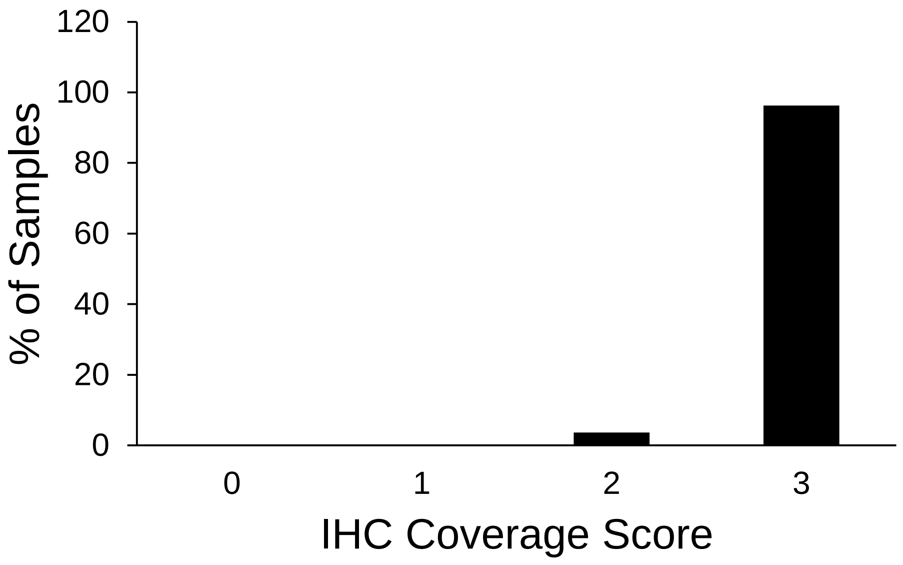

	Coverage Score			
	0	1	2	3
n	0	0	1	26
%	0.00	0.00	3.70	96.30

Normal Thyroid

	n	Average	St. Dev	St. Error
Coverage Score	28	2.96	0.19	0.04

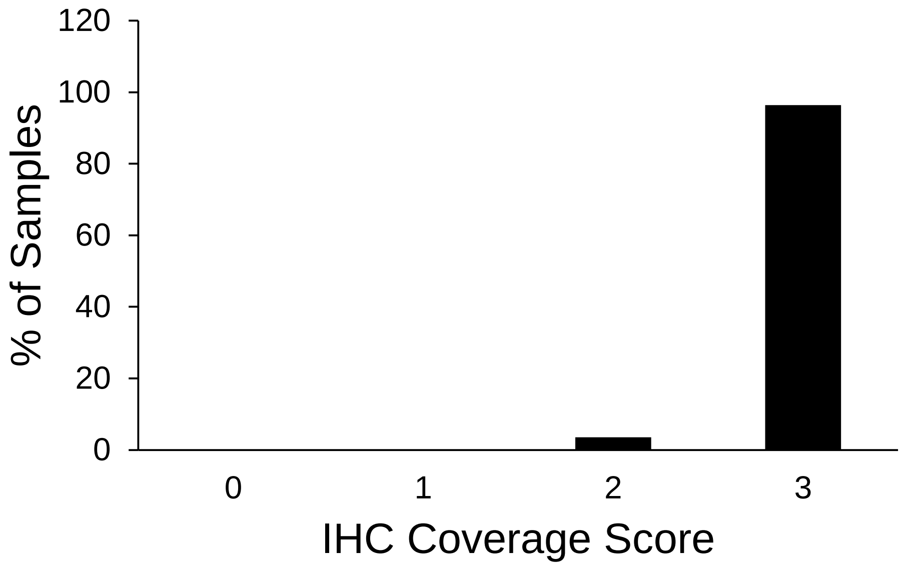

	Coverage Score			
	0	1	2	3
n	0	0	1	27
%	0.00	0.00	3.57	96.43

SI Figure 212. CCK2R Coverage Score for cancer and normal tissue from the thyroid. IHC was performed on tissue sections using a monoclonal antibody raised against CCK2R. The area stained (coverage) was graded on a scale of 0 to 3 and plotted.

Thyroid Cancer - Coverage Score

	Sex	Age at Diagnosis	Primary Tumor Type	Primary Tumor Site	Stage	Grade	Tumor Size (TNM, T)	Tumor Size (longest dimension)	Lymph Node Involvement (TNM, N)	Metastatic (TNM, M)	Metastatic Site	Survival after Diagnosis	Survival after Stage IV Diagnosis
**Spearman Correlation**	N.A.	No 0.825	N.A.	N.A.	No 0.216	No 1.00	No 0.154	No 0.245	No 0.361	N.D.	N.A.	N.D.	N.D.
**ANOVA/t-test**	No 0.185	N.D.	**Yes 0.036**	N.D.	No 0.429	No 1.00	No 0.298	N.D.	No 0.362	N.D.	N.D.	N.D.	N.D.

SI Figure 213. Coverage score correlation summary of CCK2R in thyroid cancer. IHC was performed on thyroid tumor tissue sections using a monoclonal antibody raised against CCK2R. The coverage score was compared against available patient data. If appropriate, a spearman analysis was used to determine if any significant correlation exists while a 1-way ANOVA or t-test was used to determine if a significant difference exists between groups. Whether the test was statistically significant and the p-value is listed. N.A. – not applicable (this statistical test was not applicable to this data set). N.D. – not determined (this statistical test could not be performed, generally due to a lack of the number of samples within a group or all data was in a single group).

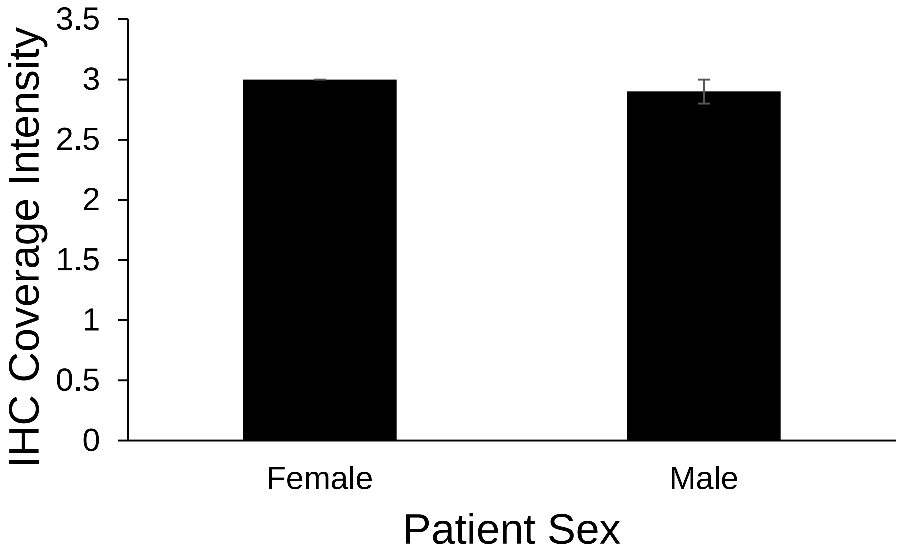

Spearman Analysis

ρ (rho) = -

t = -

p-value = -

	n	Average	St. Dev	St. Error
Female	18	3.00	0.00	0.00
Male	10	2.90	0.32	0.10

t-test

p-value = 0.1846

SI Figure 214. Correlation analysis of CCK2R coverage score in thyroid cancer versus patient sex. IHC was performed on thyroid cancer tissue sections using a monoclonal antibody raised against CCK2R. The coverage score was graded on a scale of 0 to 3 and plotted (error bars represent standard error of the mean). A t-test was used to determine if there were any significant differences between groups.

Spearman Analysis

ρ (rho) = 0.0437
t = 0.2229
p-value = 0.8253

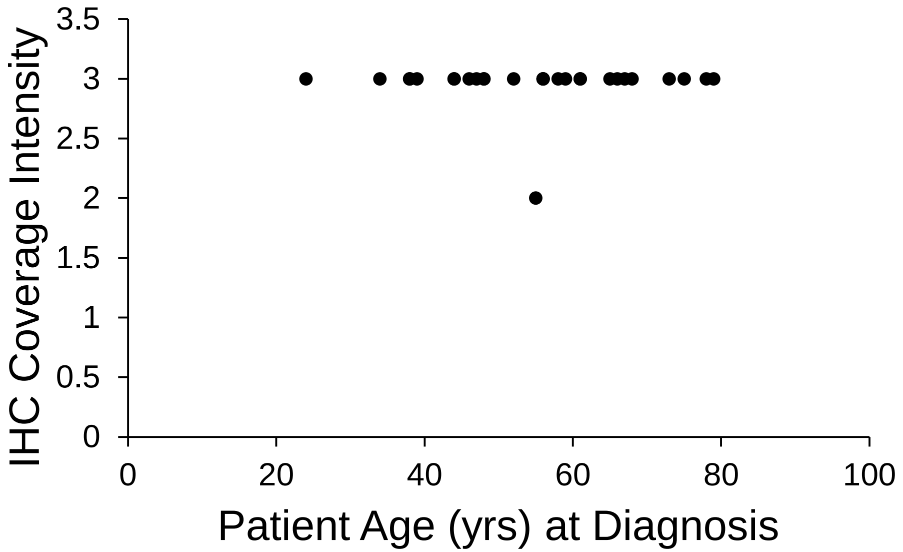

	n	Average	St. Dev	St. Error
0	0	-	-	-
1	0	-	-	-
2	1	55.00	-	-
3	27	54.81	14.13	2.72

t-test

p-value = -

SI Figure 215. Correlation analysis of CCK2R coverage score in thyroid cancer versus patient age at diagnosis. IHC was performed on thyroid cancer tissue sections using a monoclonal antibody raised against CCK2R. The coverage score was graded on a scale of 0 to 3 and plotted. A Spearman analysis was used to determine if there was a statistically significant correlation.

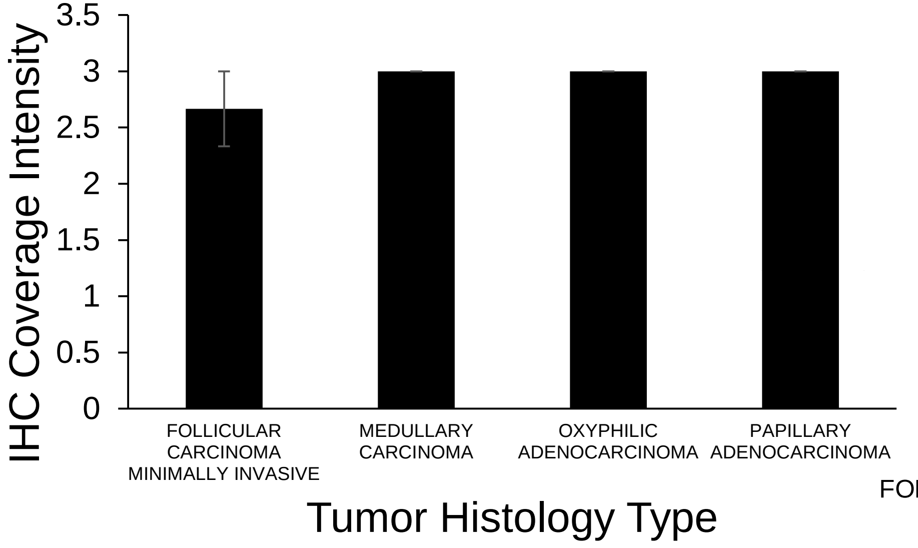

Spearman Analysis		
	ρ (rho) =	-
	t =	-
	p-value =	-

	n	Average	St. Dev	St. Error
FOLLICULAR CARCINOMA MINIMALLY INVASIVE	3	2.67	0.58	0.33
MEDULLARY CARCINOMA	5	3.00	0.00	0.00
OXYPHILIC ADENOCARCINOMA	2	3.00	-	-
PAPILLARY ADENOCARCINOMA	14	3.00	0.00	0.00

1-Way Anova

	SS	df	MS	F	p
Between	0.28	2	0.141	3.984	**0.036**
Within	0.67	19	0.035		
Total	0.96	21			

Tukey-Kramer (p-value)

	FCMI	MC	PA
FOLLICULAR CARCINOMA MINIMALLY INVASIVE	-	**0.045**	**0.025**
MEDULLARY CARCINOMA		-	0.900
PAPILLARY ADENOCARCINOMA			-

SI Figure 216. Correlation analysis of CCK2R coverage score in thyroid cancer versus type of primary tumor. IHC was performed on thyroid cancer tissue sections using a monoclonal antibody raised against CCK2R. The coverage score was graded on a scale of 0 to 3 and plotted (error bars represent standard error of the mean). A 1-way ANOVA followed by a Tukey-Kramer ad hoc post analysis was used to determine if there were any significant differences between groups.

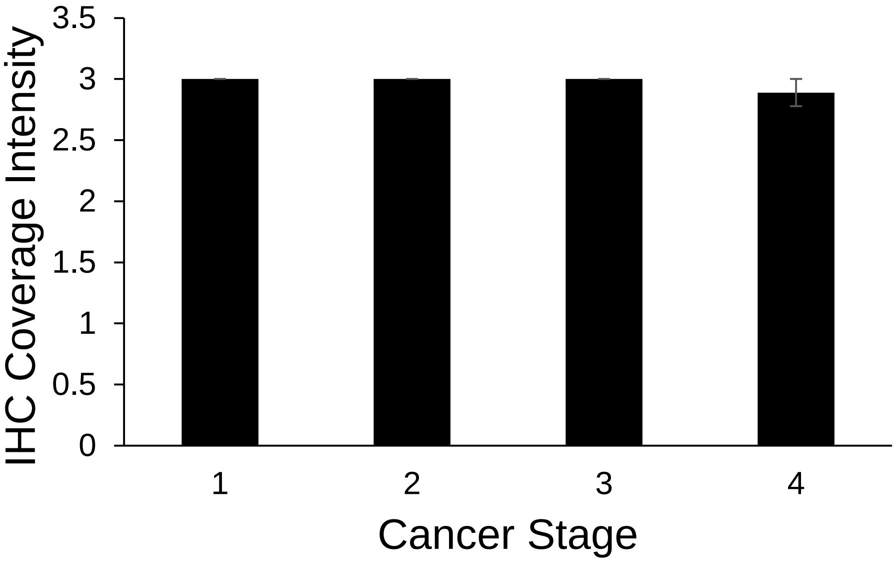

Spearman Analysis		
	ρ (rho) =	-0.2457
	t =	-1.2673
	p-value =	0.2163

	n	Average	St. Dev	St. Error
1	13	3.00	0.00	0.00
2	2	3.00	-	-
3	3	3.00	0.00	0.00
4	9	2.89	0.33	0.11

1-Way Anova

	SS	df	MS	F	p
Between	0.07	2	0.035	0.880	0.429
Within	0.87	22	0.040		
Total	0.94	24			

SI Figure 217. Correlation analysis of CCK2R coverage score in thyroid cancer versus cancer stage. IHC was performed on thyroid cancer tissue sections using a monoclonal antibody raised against CCK2R. The coverage score was graded on a scale of 0 to 3 and plotted (error bars represent standard error of the mean). A Spearman analysis was used to determine if there was a statistically significant correlation and a 1-way ANOVA was used to determine if there were any significant differences between groups.

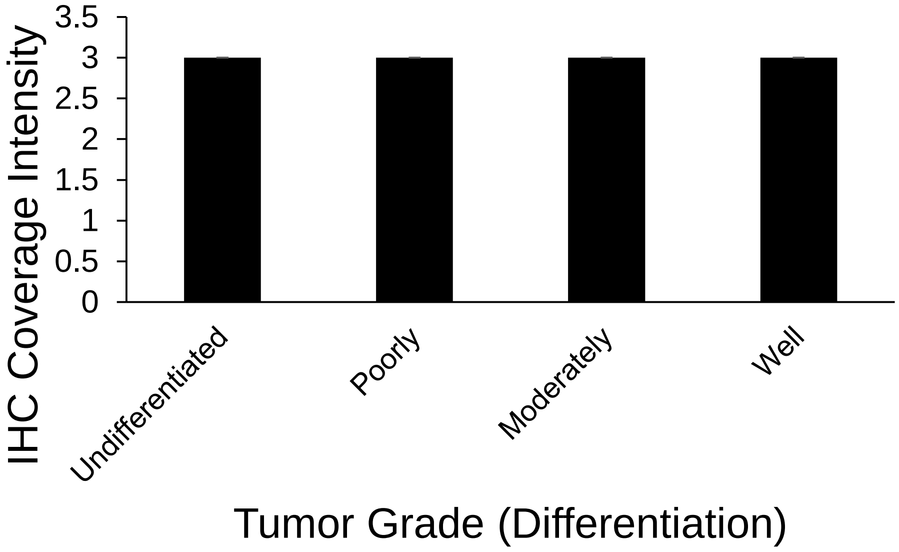

Spearman Analysis		
	ρ (rho) =	0.0000
	t =	0.0000
	p-value =	1.0000

	n	Average	St. Dev	St. Error
Undifferentiated	2	3.00	-	-
Poorly	2	3.00	-	-
Moderately	6	3.00	0.00	0.00
Well	3	3.00	0.00	0.00

t-test

p-value = 1.0000

SI Figure 218. Correlation analysis of CCK2R coverage score in thyroid cancer versus primary tumor grade. IHC was performed on thyroid cancer tissue sections using a monoclonal antibody raised against CCK2R. The coverage score was graded on a scale of 0 to 3 and plotted (error bars represent standard error of the mean). A Spearman analysis was used to determine if there was a statistically significant correlation and a t-test was used to determine if there were any significant differences between groups.

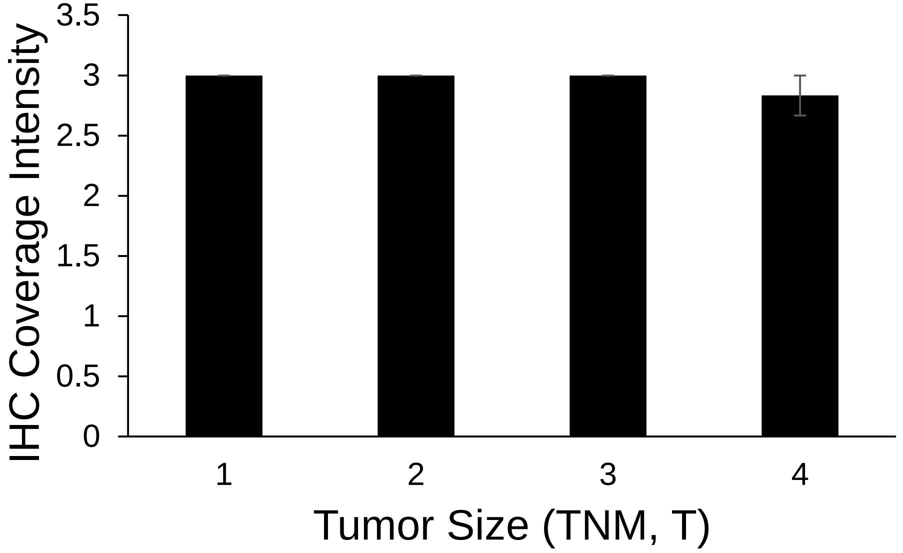

Spearman Analysis

ρ (rho) =	-0.2763
t =	-1.4658
p-value =	0.1543

	n	Average	St. Dev	St. Error
1	12	3.00	0.00	0.00
2	4	3.00	0.00	0.00
3	6	3.00	0.00	0.00
4	6	2.83	0.41	0.17

1-Way Anova

	SS	df	MS	F	p
Between	0.14	3	0.045	1.297	0.298
Within	0.84	24	0.035		
Total	0.98	27			

SI Figure 219. Correlation analysis of CCK2R coverage score in thyroid cancer versus primary tumor size. IHC was performed on thyroid cancer tissue sections using a monoclonal antibody raised against CCK2R. The coverage score was graded on a scale of 0 to 3 and plotted (error bars represent standard error of the mean). A Spearman analysis was used to determine if there was a statistically significant correlation and a 1-way ANOVA was used to determine if there were any significant differences between groups.

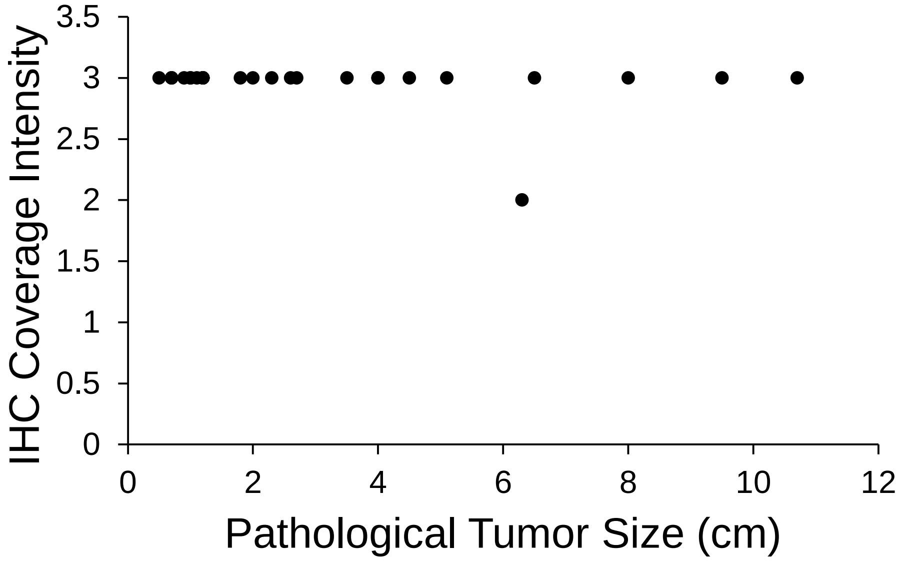

Spearman Analysis

ρ (rho) = -0.2271
t = -1.1893
p-value = 0.2447

	n	Average	St. Dev	St. Error
0	0	-	-	-
1	0	-	-	-
2	1	6.30	-	-
3	27	2.96	2.81	0.54

t-test

p-value = -

SI Figure 220. Correlation analysis of CCK2R coverage score in thyroid cancer versus size of primary tumor (length of longest side). IHC was performed on thyroid cancer tissue sections using a monoclonal antibody raised against CCK2R. The coverage score was graded on a scale of 0 to 3 and plotted. A Spearman analysis was used to determine if there was a statistically significant correlation.

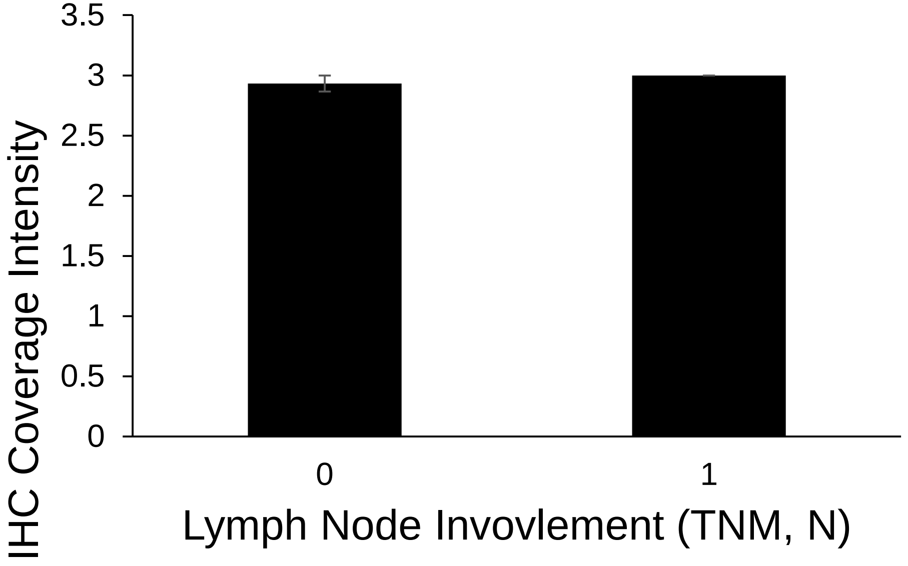

Spearman Analysis		
	ρ (rho) =	0.1792
	t =	0.9286
	p-value =	0.3613

	n	Average	St. Dev	St. Error
0	15	2.93	0.26	0.07
1	13	3.00	0.00	0.00

t-test

p-value = 0.3617

SI Figure 221. Correlation analysis of CCK2R coverage score in thyroid cancer versus lymph node involvement. IHC was performed on thyroid cancer tissue sections using a monoclonal antibody raised against CCK2R. The coverage score was graded on a scale of 0 to 3 and plotted (error bars represent standard error of the mean). A Spearman analysis was used to determine if there was a statistically significant correlation and a t-test was used to determine if there were any significant differences between groups.

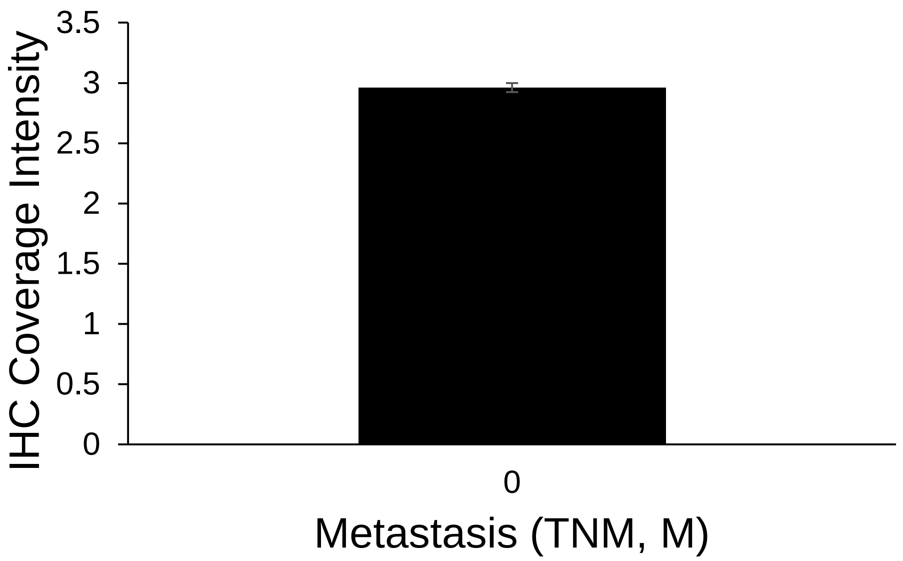

Spearman Analysis	
ρ (rho) =	-
t =	-
p-value =	-

	n	Average	St. Dev	St. Error
0	26	2.96	0.20	0.04

t-test

p-value = -

SI Figure 222. Correlation analysis of CCK2R coverage score in thyroid cancer versus metastases. IHC was performed on thyroid cancer tissue sections using a monoclonal antibody raised against CCK2R. The coverage score was graded on a scale of 0 to 3 and plotted (error bars represent standard error of the mean). No statistical tests could be performed.

Thyroid Cancer
Total Staining Score Correlations

Thyroid Cancer

	n	Average	St. Dev	St. Error
Total Score	27	4.74	1.95	0.38

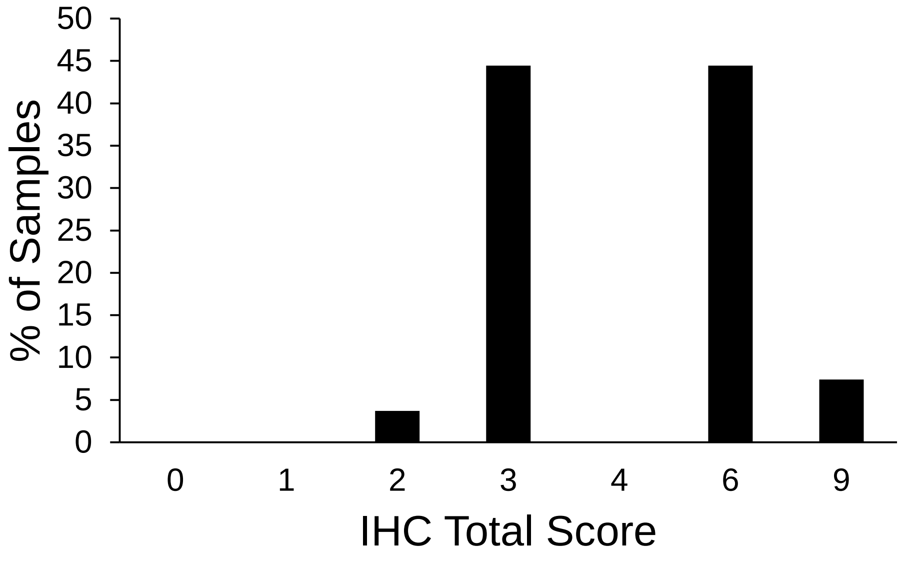

	Total Staining Score						
	0	1	2	3	4	6	9
n	0	0	1	12	0	12	2
%	0.00	0.00	3.70	44.44	0.00	44.44	7.41

Normal Thyroid

	n	Average	St. Dev	St. Error
Total Score	28	3.18	0.82	0.15

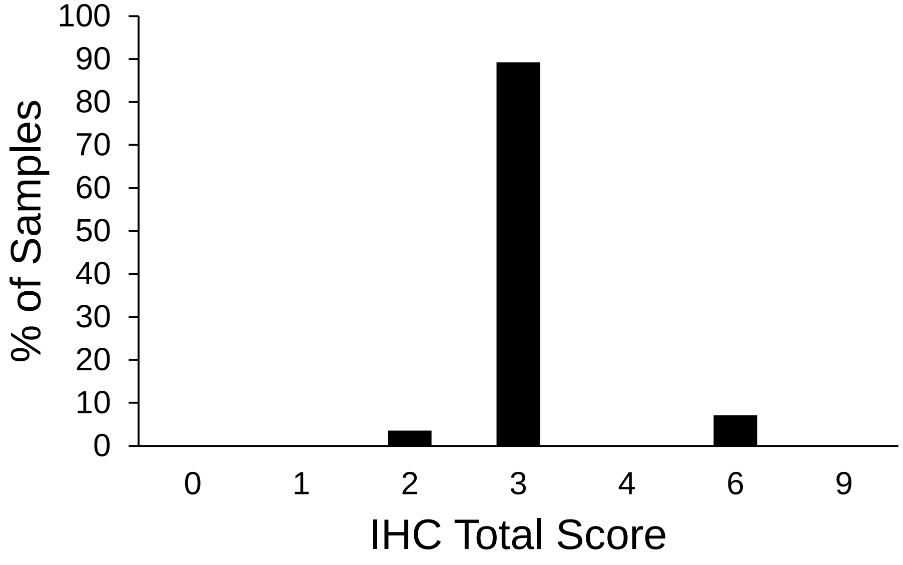

	Total Staining Score						
	0	1	2	3	4	6	9
n	0	0	1	25	0	2	0
%	0.00	0.00	3.57	89.29	0.00	7.14	0.00

SI Figure 223. CCK2R Total Staining Score for cancer and normal tissue from the thyroid. IHC was performed on tissue sections using a monoclonal antibody raised against CCK2R. The staining intensity and coverage score was multiplied to obtain the total staining score.

Thyroid Cancer - Total Staining Score

	Sex	Age at Diagnosis	Primary Tumor Type	Primary Tumor Site	Stage	Grade	Tumor Size (TNM, T)	Tumor Size (longest dimension)	Lymph Node Involvement (TNM, N)	Metastatic (TNM, M)	Metastatic Site	Survival after Diagnosis	Survival after Stage IV Diagnosis
**Spearman Correlation**	N.A.	No 0.800	N.A.	N.A.	No 0.380	No 0.978	No 0.919	No 0.811	**Yes 0.010**	N.D.	N.A.	N.D.	N.D.
**ANOVA/t-test**	No 0.670	No 0.843	No 0.684	N.D.	No 0.369	No 1.00	No 0.764	No 0.992	**Yes 0.009**	N.D.	N.D.	N.D.	N.D.

SI Figure 224. Total staining score correlation summary of CCK2R in thyroid cancer. IHC was performed on thyroid tumor tissue sections using a monoclonal antibody raised against CCK2R. The total staining score was compared against available patient data. If appropriate, a spearman analysis was used to determine if any significant correlation exists while a 1-way ANOVA or t-test was used to determine if a significant difference exists between groups. Whether the test was statistically significant and the p-value is listed. N.A. – not applicable (this statistical test was not applicable to this data set). N.D. – not determined (this statistical test could not be performed, generally due to a lack of the number of samples within a group or all data was in a single group).

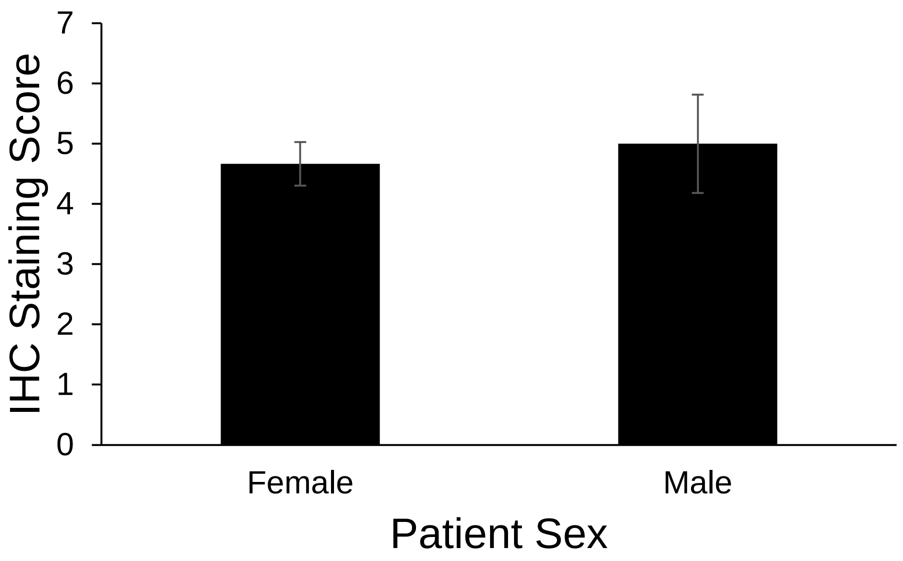

Spearman Analysis

ρ (rho) = -
t = -
p-value = -

	n	Average	St. Dev	St. Error
Female	18	4.67	1.53	0.36
Male	10	5.00	2.58	0.82

t-test

p-value = 0.6701

SI Figure 225. Correlation analysis of CCK2R total staining score in thyroid cancer versus patient sex. IHC was performed on thyroid cancer tissue sections using a monoclonal antibody raised against CCK2R. The total staining score was derived by multiplying the staining intensity with the coverage score and plotted (error bars represent standard error of the mean). A t-test was used to determine if there were any significant differences between groups.

Spearman Analysis		
	ρ (rho) =	-0.0500
	t =	-0.2554
	p-value =	0.8004

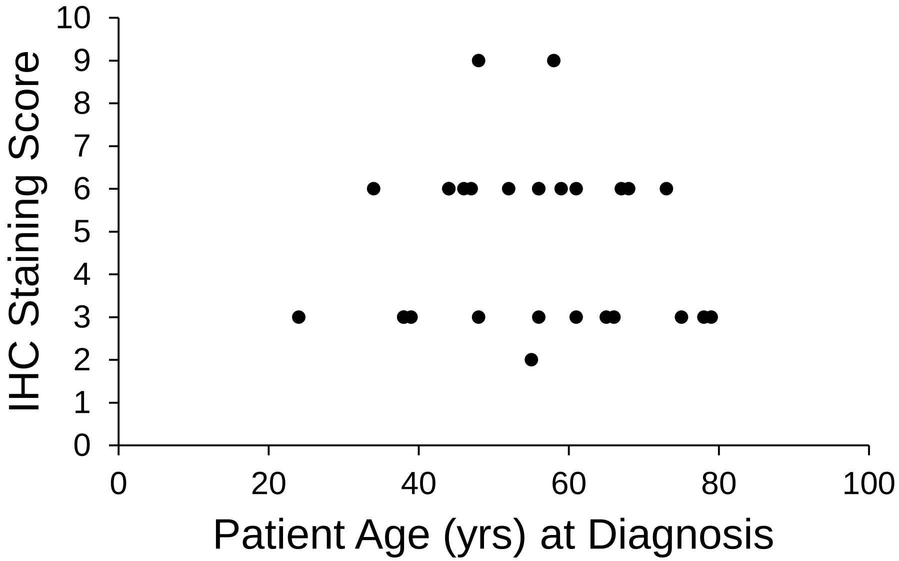

	n	Average	St. Dev	St. Error
0	0	-	-	-
1	0	-	-	-
2	1	55.00	-	-
3	12	55.58	18.10	5.22
4	0	-	-	-
6	13	54.38	11.27	3.12
9	2	53.00	-	-

t-test

p-value = 0.8428

SI Figure 226. Correlation analysis of CCK2R total staining score in thyroid cancer versus patient age at diagnosis. IHC was performed on thyroid cancer tissue sections using a monoclonal antibody raised against CCK2R. The total staining score was derived by multiplying the staining intensity with the coverage score and plotted. A Spearman analysis was used to determine if there was a statistically significant correlation and a t-test was used to determine if there were any significant differences between groups.

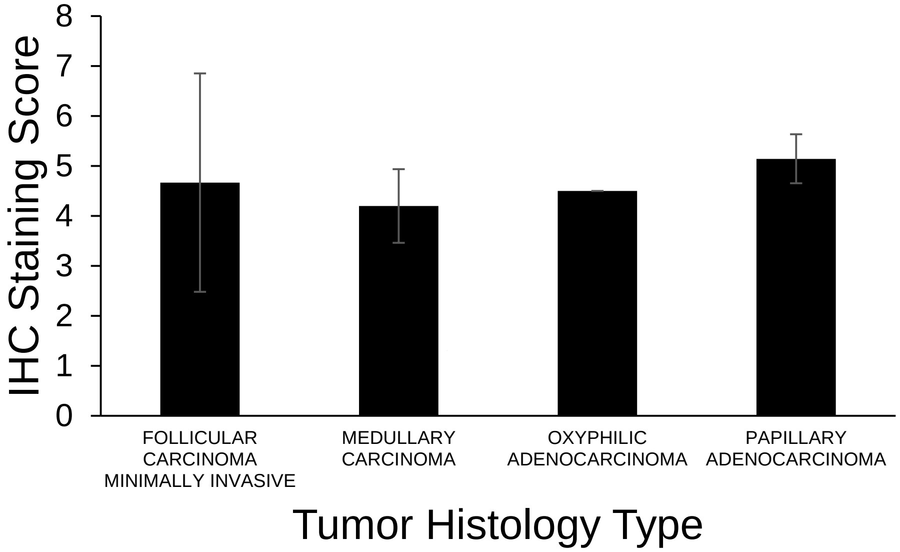

Spearman Analysis	
ρ (rho) =	-
t =	-
p-value =	-

	n	Average	St. Dev	St. Error
FOLLICULAR CARCINOMA MINIMALLY INVASIVE	3	4.67	3.79	2.19
MEDULLARY CARCINOMA	5	4.20	1.64	0.73
OXYPHILIC ADENOCARCINOMA	2	4.50	-	-
PAPILLARY ADENOCARCINOMA	14	5.14	1.83	0.49

1-Way Anova

	SS	df	MS	F	p
Between	3.38	2	1.692	0.387	0.684
Within	83.02	19	4.370		
Total	86.41	21			

SI Figure 227. Correlation analysis of CCK2R total staining score in thyroid cancer versus type of primary tumor. IHC was performed on thyroid cancer tissue sections using a monoclonal antibody raised against CCK2R. The total staining score was derived by multiplying the staining intensity with the coverage score and plotted (error bars represent standard error of the mean). A 1-way ANOVA was used to determine if there were any significant differences between groups.

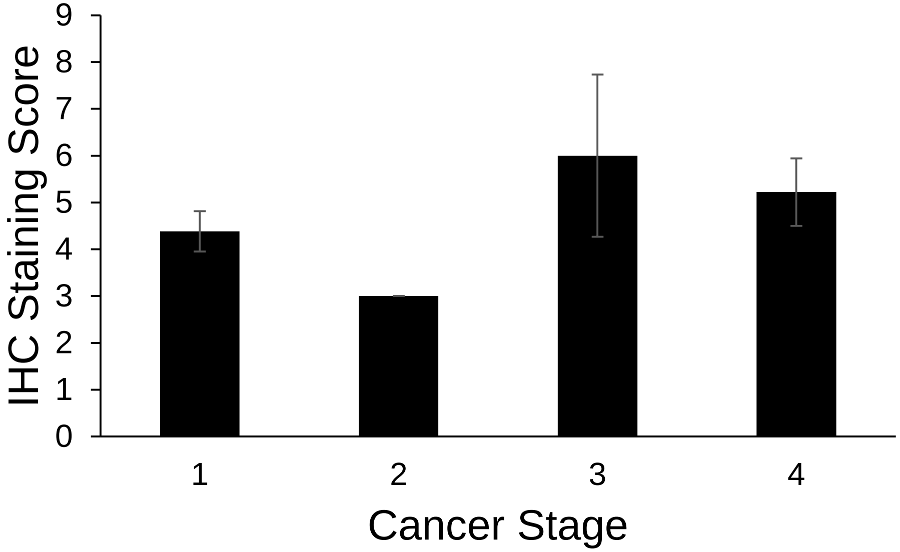

Spearman Analysis

Spearman Analysis	ρ (rho) =	0.1760
	t =	0.8938
	p-value =	0.3796

	n	Average	St. Dev	St. Error
1	13	4.38	1.56	0.43
2	2	3.00	-	-
3	3	6.00	3.00	1.73
4	9	5.22	2.17	0.72

1-Way Anova

	SS	df	MS	F	p
Between	8.05	2	4.03	1.044	0.369
Within	84.87	22	3.86		
Total	92.93	24			

SI Figure 228. Correlation analysis of CCK2R total staining score in thyroid cancer versus cancer stage. IHC was performed on thyroid cancer tissue sections using a monoclonal antibody raised against CCK2R. The total staining score was derived by multiplying the staining intensity with the coverage score and plotted (error bars represent standard error of the mean). A Spearman analysis was used to determine if there was a statistically significant correlation and a 1-way ANOVA was used to determine if there were any significant differences between groups.

Spearman Analysis

ρ (rho) =	0.0084
t =	0.0280
p-value =	0.9782

	n	Average	St. Dev	St. Error
Undifferentiated	2	4.50	-	-
Poorly	2	6.00	-	-
Moderately	6	5.00	2.45	1.00
Well	3	5.00	1.73	1.00

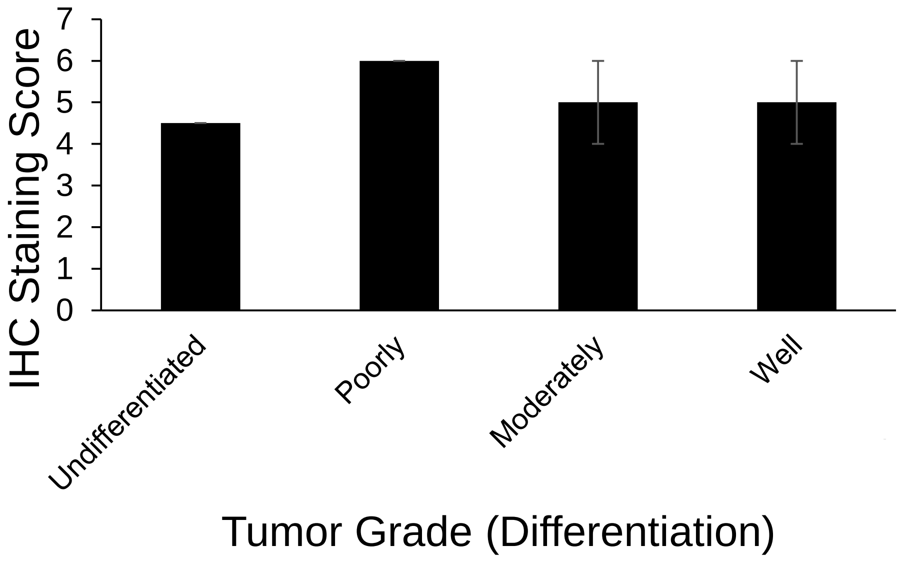

t-test

p-value = 1.0000

SI Figure 229. Correlation analysis of CCK2R total staining score in thyroid cancer versus primary tumor grade. IHC was performed on thyroid cancer tissue sections using a monoclonal antibody raised against CCK2R. The total staining score was derived by multiplying the staining intensity with the coverage score and plotted (error bars represent standard error of the mean). A Spearman analysis was used to determine if there was a statistically significant correlation and a t-test was used to determine if there were any significant differences between groups.

Spearman Analysis		
	ρ (rho) =	-0.0200
	t =	-0.1023
	p-value =	0.9193

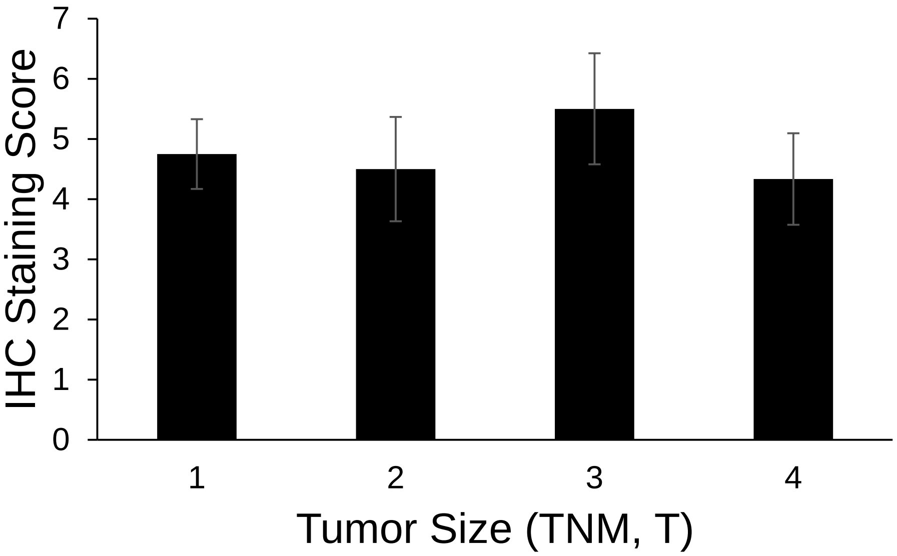

	n	Average	St. Dev	St. Error
1	12	4.75	2.01	0.58
2	4	4.50	1.73	0.87
3	6	5.50	2.26	0.92
4	6	4.33	1.86	0.76

1-Way Anova

	SS	df	MS	F	p
Between	4.65	3	1.55	0.386	0.764
Within	96.26	24	4.01		
Total	100.91	27			

SI Figure 230. Correlation analysis of CCK2R total staining score in thyroid cancer versus primary tumor size. IHC was performed on thyroid cancer tissue sections using a monoclonal antibody raised against CCK2R. The total staining score was derived by multiplying the staining intensity with the coverage score and plotted (error bars represent standard error of the mean). A Spearman analysis was used to determine if there was a statistically significant correlation and a 1-way ANOVA was used to determine if there were any significant differences between groups.

Spearman Analys ρ (rho) = -0.0473
t = -0.2413
p-value = 0.8112

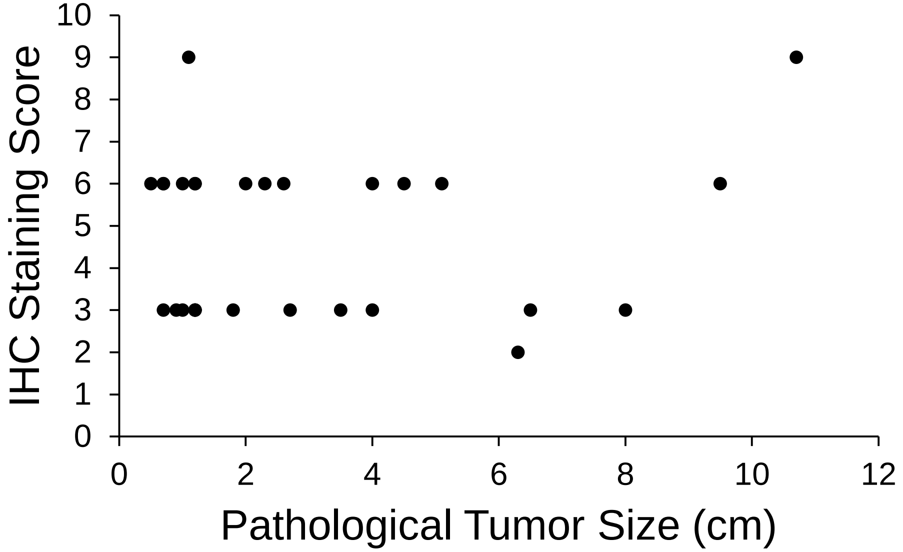

	n	Average	St. Dev	St. Error
0	0	-	-	-
1	0	-	-	-
2	1	6.30	-	-
3	12	2.73	2.38	0.69
4	0	-	-	-
6	13	2.72	2.55	0.71
9	2	5.90	-	-

t-test

p-value = 0.9923

SI Figure 231. Correlation analysis of CCK2R total staining score in thyroid cancer versus size of primary tumor (length of longest side). IHC was performed on thyroid cancer tissue sections using a monoclonal antibody raised against CCK2R. The total staining score was derived by multiplying the staining intensity with the coverage score and plotted. A Spearman analysis was used to determine if there was a statistically significant correlation and a t-test was used to determine if there were any significant differences between groups.

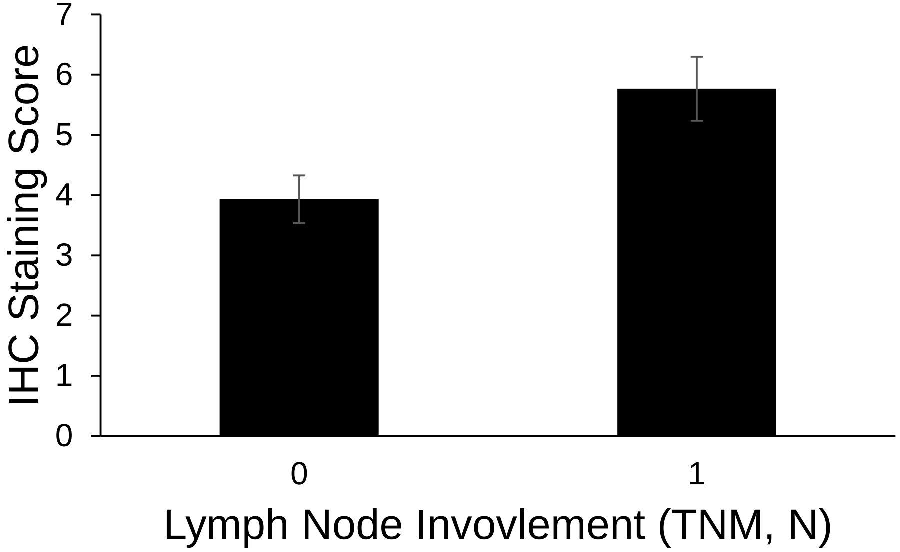

Spearman Analysis

ρ (rho) =	0.4792
t =	2.7837
p-value =	**0.0097**

	n	Average	St. Dev	St. Error
0	15	3.93	1.53	0.40
1	13	5.77	1.92	0.53

t-test

p-value = **0.0093**

SI Figure 232. Correlation analysis of CCK2R total staining score in thyroid cancer versus lymph node involvement. IHC was performed on thyroid cancer tissue sections using a monoclonal antibody raised against CCK2R. The total staining score was derived by multiplying the staining intensity with the coverage score and plotted (error bars represent standard error of the mean). A Spearman analysis was used to determine if there was a statistically significant correlation and a t-test was used to determine if there were any significant differences between groups.

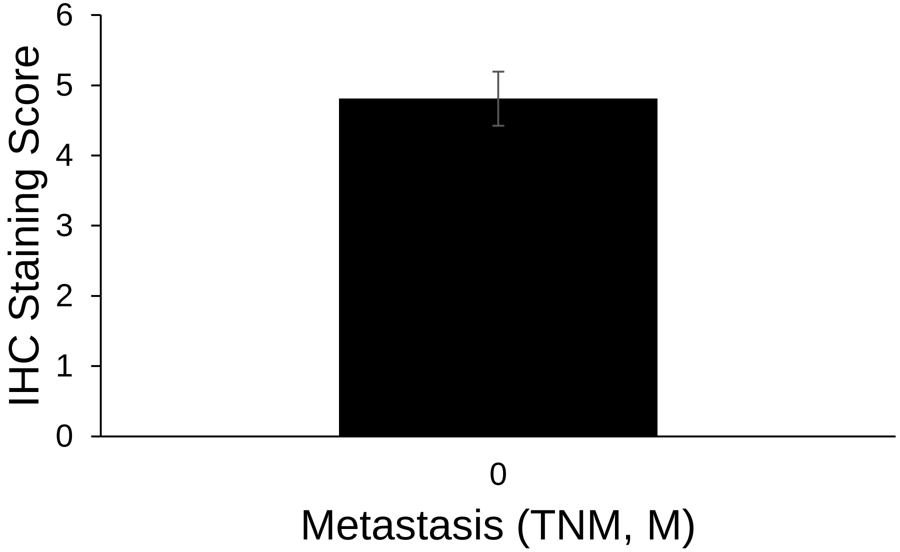

Spearman Analysis ρ (rho) = -
t = -
p-value = -

	n	Average	St. Dev	St. Error
0	26	4.81	1.96	0.38

t-test

p-value = -

SI Figure 233. Correlation analysis of CCK2R total staining score in thyroid cancer versus metastases. IHC was performed on thyroid cancer tissue sections using a monoclonal antibody raised against CCK2R. The total staining score was derived by multiplying the staining intensity with the coverage score and plotted (error bars represent standard error of the mean). No statistical tests could be performed.

Normal Tissue

Normal Tissue
Global Summary

Normal Tissue
Global Staining Intensity

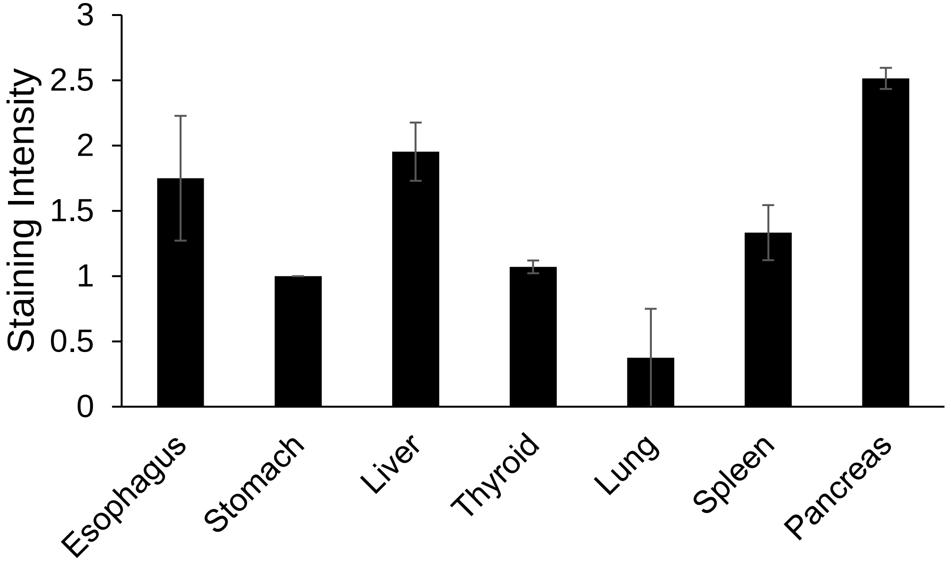

	Intensity Score			
	n	Average	St. Dev.	SEM
Esophagus	4	1.75	0.96	0.48
Stomach	3	1.00	0.00	0.00
Liver	22	1.95	1.05	0.22
Thyroid	28	1.07	0.26	0.05
Lung	8	0.38	1.06	0.38
Spleen	6	1.33	0.52	0.21
Pancreas	66	2.52	0.66	0.08

SI Figure 234. Average CCK2R Staining Intensity for all normal tissues tested. IHC was performed on tissue sections using a monoclonal antibody raised against CCK2R. The intensity of staining was graded on a scale of 0 to 3 and plotted (error bars represent standard error of the mean).

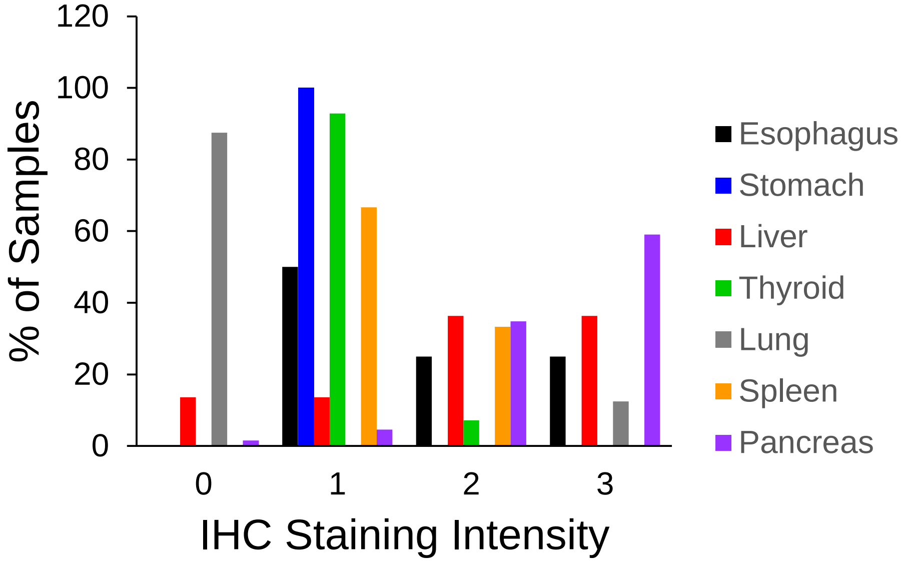

	Staining Intensity (n in each group)			
	0	1	2	3
Esophagus	0	2	1	1
Stomach	0	3	0	0
Liver	3	3	8	8
Thyroid	0	26	2	0
Lung	7	0	0	1
Spleen	0	4	2	0
Pancreas	1	3	23	39

	Staining Intensity (% in each group)			
	0	1	2	3
Esophagus	0.00	50.00	25.00	25.00
Stomach	0.00	100.00	0.00	0.00
Liver	13.64	13.64	36.36	36.36
Thyroid	0.00	92.86	7.14	0.00
Lung	87.50	0.00	0.00	12.50
Spleen	0.00	66.67	33.33	0.00
Pancreas	1.52	4.55	34.85	59.09

SI Figure 235. CCK2R Staining Intensity for all normal tissues tested. IHC was performed on tissue sections using a monoclonal antibody raised against CCK2R. The intensity of staining was graded on a scale of 0 to 3 and plotted.

Normal Tissue
Global Coverage Score

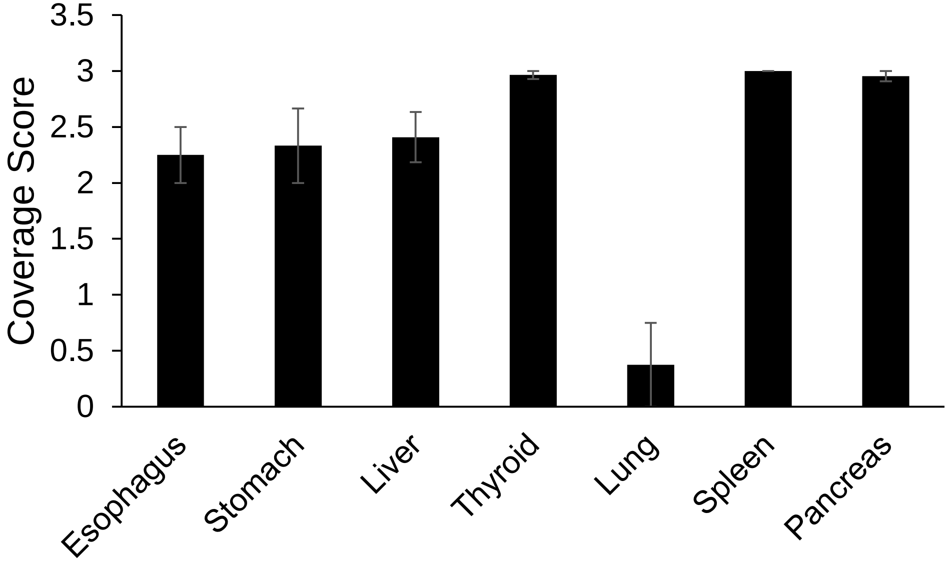

	Coverage Score			
	n	Average	St. Dev.	SEM
Esophagus	4	2.25	0.50	0.25
Stomach	3	2.33	0.58	0.33
Liver	22	2.41	1.05	0.22
Thyroid	28	2.96	0.19	0.04
Lung	8	0.38	1.06	0.38
Spleen	6	3.00	0.00	0.00
Pancreas	66	2.95	0.37	0.05

SI Figure 236. Average CCK2R Coverage Score for all normal tissues tested. IHC was performed on tissue sections using a monoclonal antibody raised against CCK2R. The area stained (coverage) was graded on a scale of 0 to 3 and plotted (error bars represent standard error of the mean.

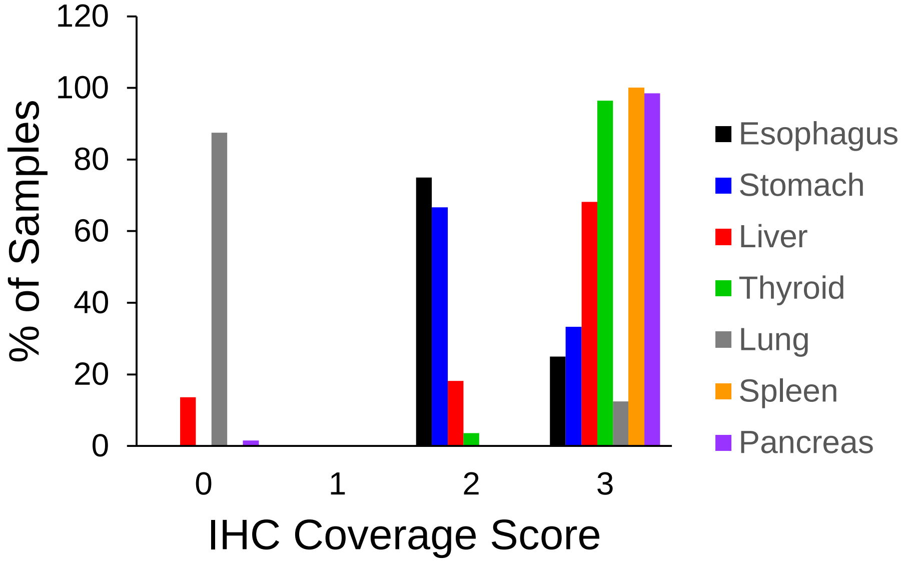

	Coverage Score (n in each group)			
	0	1	2	3
Esophagus	0	0	3	1
Stomach	0	0	2	1
Liver	3	0	4	15
Thyroid	0	0	1	27
Lung	7	0	0	1
Spleen	0	0	0	6
Pancreas	1	0	0	65

	Coverage Score (% in each group)			
	0	1	2	3
Esophagus	0.00	0.00	75.00	25.00
Stomach	0.00	0.00	66.67	33.33
Liver	13.64	0.00	18.18	68.18
Thyroid	0.00	0.00	3.57	96.43
Lung	87.50	0.00	0.00	12.50
Spleen	0.00	0.00	0.00	100.00
Pancreas	1.52	0.00	0.00	98.48

SI Figure 237. CCK2R Coverage Score for all normal tissues tested. IHC was performed on tissue sections using a monoclonal antibody raised against CCK2R. The area stained (coverage) was graded on a scale of 0 to 3 and plotted.

Normal Tissue
Global Total Staining Score

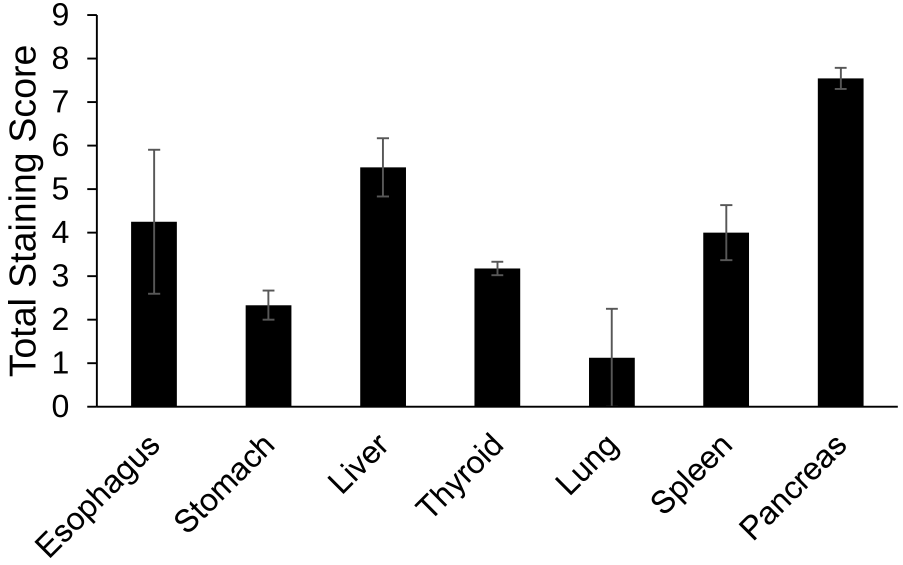

	Total Score			
	n	Average	St. Dev.	SEM
Esophagus	4	4.25	3.30	1.65
Stomach	3	2.33	0.58	0.33
Liver	22	5.50	3.14	0.67
Thyroid	28	3.18	0.82	0.15
Lung	8	1.13	3.18	1.13
Spleen	6	4.00	1.55	0.63
Pancreas	66	7.55	1.99	0.24

SI Figure 238. Average CCK2R Total Staining Score for all normal tissues tested. IHC was performed on tissue sections using a monoclonal antibody raised against CCK2R. The staining intensity and coverage score was multiplied to obtain the total staining score and plotted (error bars represent standard error of the mean).

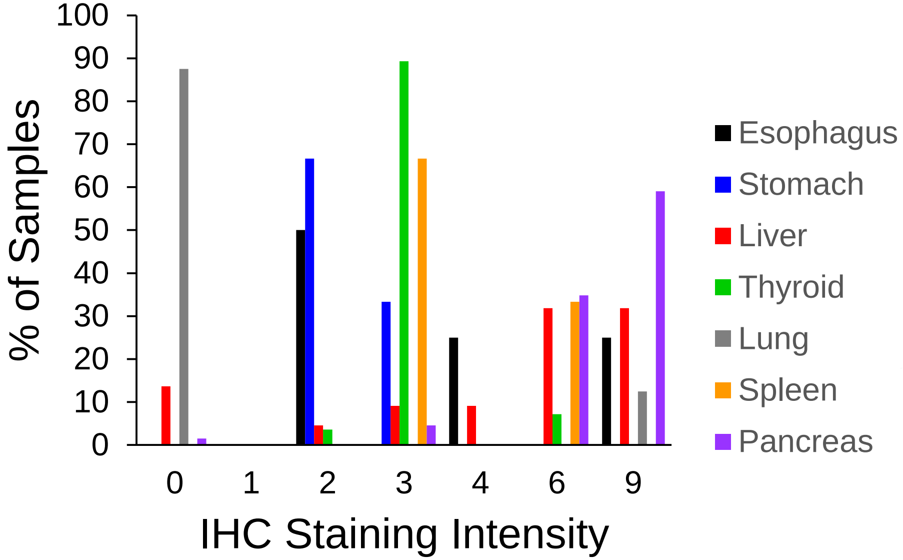

Total Staining Score (n in each group)

	0	1	2	3	4	6	9
Esophagus	0	0	2	0	1	0	1
Stomach	0	0	2	1	0	0	0
Liver	3	0	1	2	2	7	7
Thyroid	0	0	1	25	0	2	0
Lung	7	0	0	0	0	0	1
Spleen	0	0	0	4	0	2	0
Pancreas	1	0	0	3	0	23	39

Total Staining Score (% in each group)

	0	1	2	3	4	6	9
Esophagus	0.00	0.00	50.00	0.00	25.00	0.00	25.00
Stomach	0.00	0.00	66.67	33.33	0.00	0.00	0.00
Liver	13.64	0.00	4.55	9.09	9.09	31.82	31.82
Thyroid	0.00	0.00	3.57	89.29	0.00	7.14	0.00
Lung	87.50	0.00	0.00	0.00	0.00	0.00	12.50
Spleen	0.00	0.00	0.00	66.67	0.00	33.33	0.00
Pancreas	1.52	0.00	0.00	4.55	0.00	34.85	59.09

SI Figure 239. CCK2R Total Staining Score for all normal tissues tested. IHC was performed on tissue sections using a monoclonal antibody raised against CCK2R. The staining intensity and coverage score was multiplied to obtain the total staining score and plotted.

Normal Esophagus

Normal Esophagus Tissue Images

Normal Esophagus

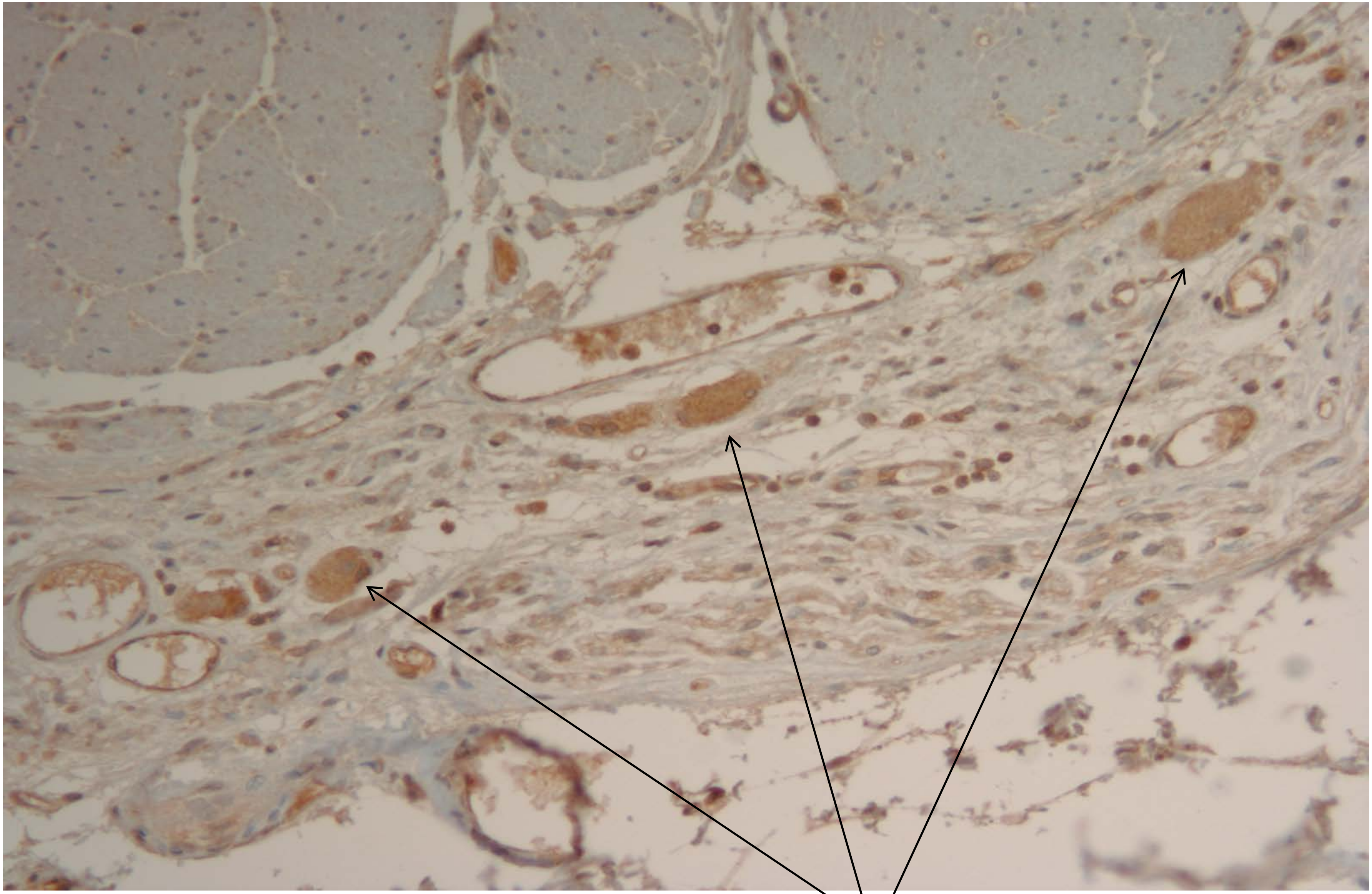

SI Figure 240. Example image of normal stained tissue from esophagus. IHC was performed on tissue sections using a monoclonal antibody raised against CCK2R.

Normal Esophagus

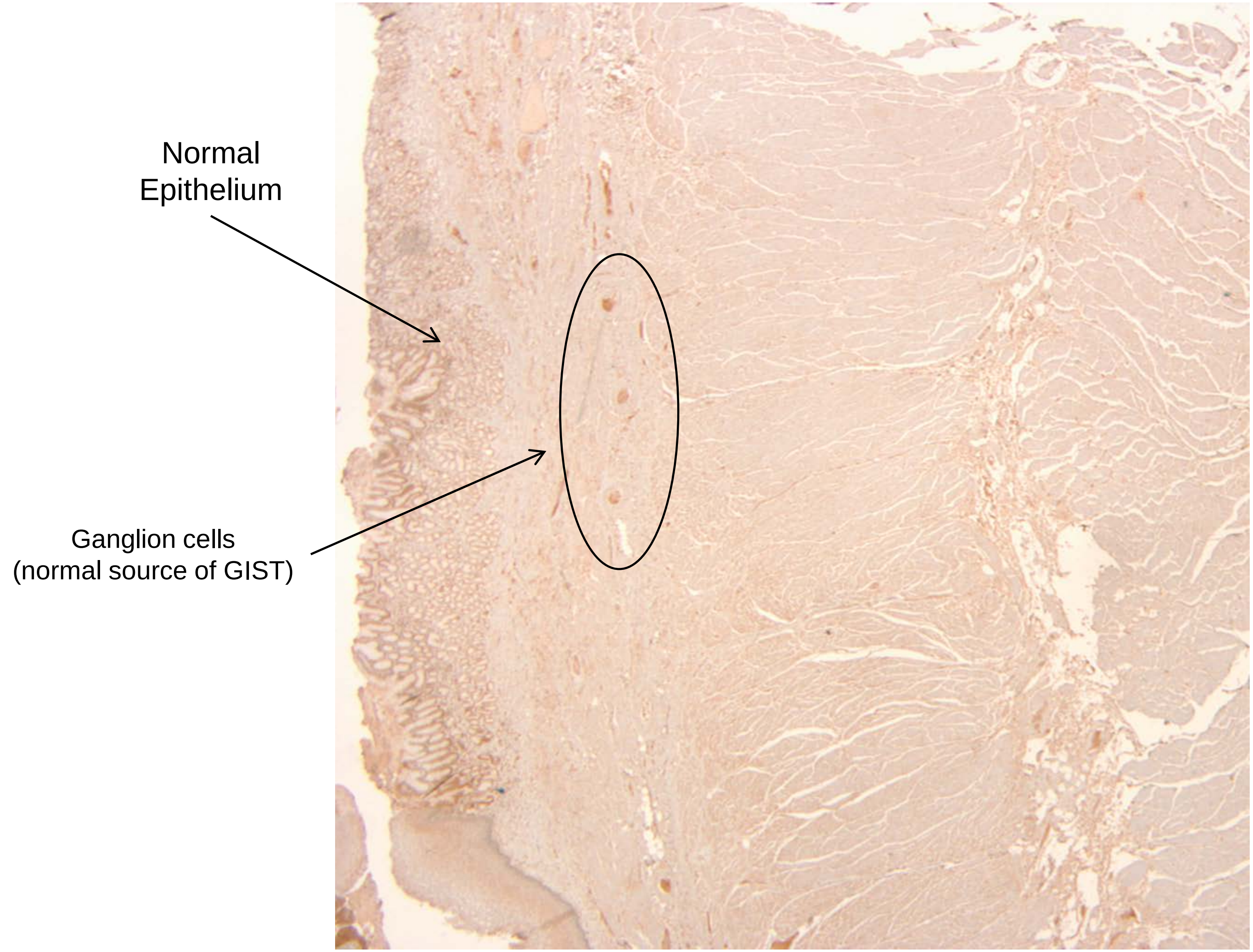

SI Figure 241. Example image of normal stained tissue from esophagus. IHC was performed on tissue sections using a monoclonal antibody raised against CCK2R.

Normal Esophagus Tissue Staining Intensity

	n	Average	St. Dev	St. Error
Staining Intensity	4	1.75	0.96	0.48

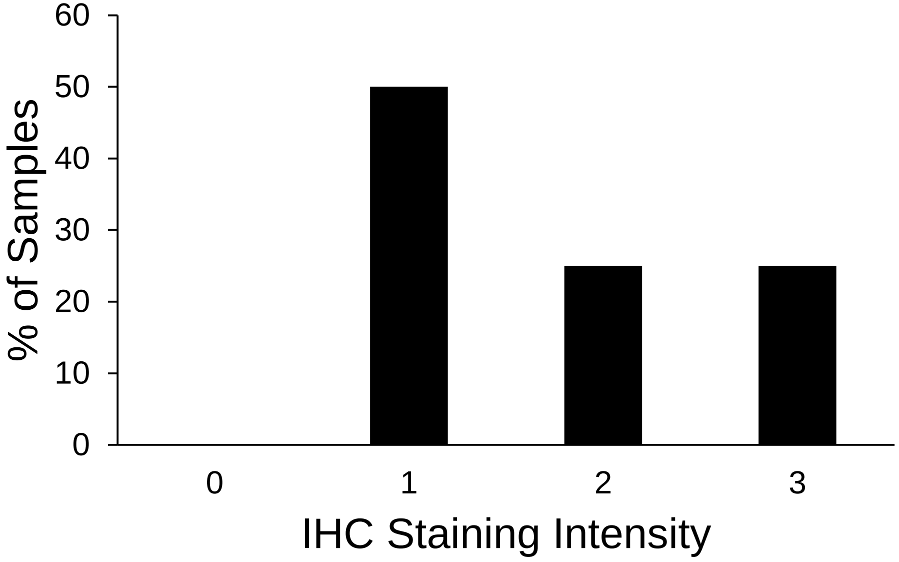

	Staining Intensity			
	0	1	2	3
n	0	2	1	1
%	0.00	50.00	25.00	25.00

SI Figure 242. CCK2R Staining Intensity for normal tissue from the esophagus. IHC was performed on tissue sections using a monoclonal antibody raised against CCK2R. The intensity of staining was graded on a scale of 0 to 3 and plotted.

Normal Esophagus Tissue Coverage Score Correlations

	n	Average	St. Dev	St. Error
Coverage Score	4	2.25	0.50	0.25

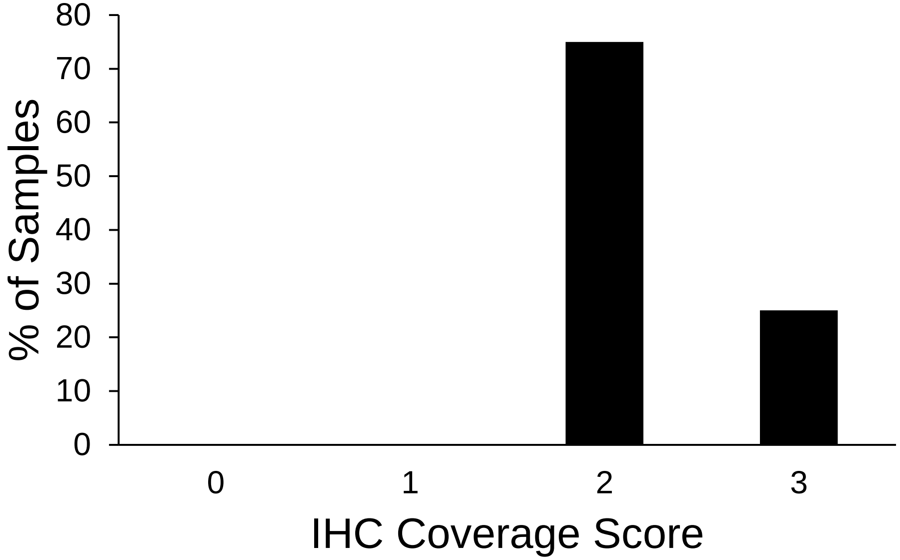

	Coverage Score			
	0	1	2	3
n	0	0	3	1
%	0.00	0.00	75.00	25.00

SI Figure 243. CCK2R Coverage Score for normal tissue from the esophagus. IHC was performed on tissue sections using a monoclonal antibody raised against CCK2R. The area stained (coverage) was graded on a scale of 0 to 3 and plotted.

Normal Esophagus Tissue
Total Staining Score Correlations

	n	Average	St. Dev	St. Error
Total Score	4	4.25	3.30	1.65

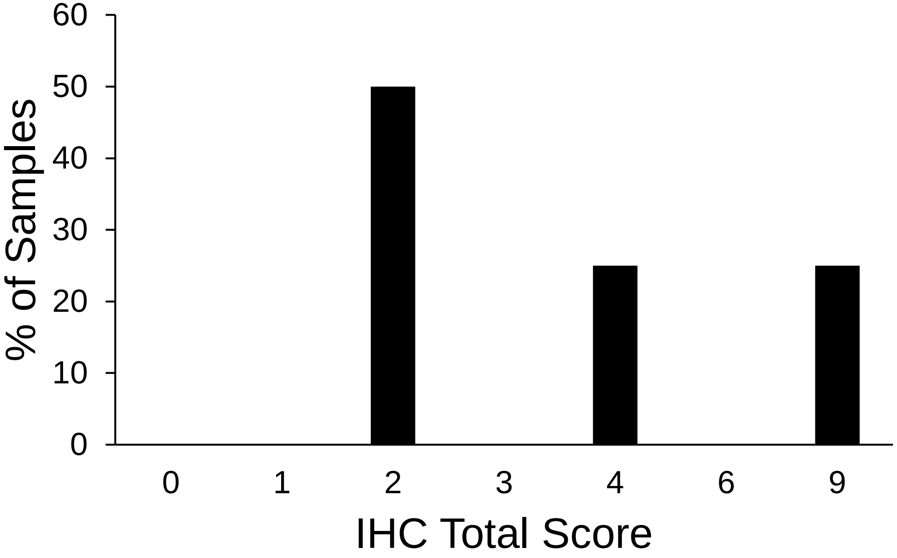

	Total Staining Score						
	0	1	2	3	4	6	9
n	0	0	2	0	1	0	1
%	0.00	0.00	50.00	0.00	25.00	0.00	25.00

SI Figure 244. CCK2R Total Staining Score for normal tissue from the esophagus. IHC was performed on tissue sections using a monoclonal antibody raised against CCK2R. The staining intensity and coverage score was multiplied to obtain the total staining score.

Normal Liver

Normal Liver Tissue Images

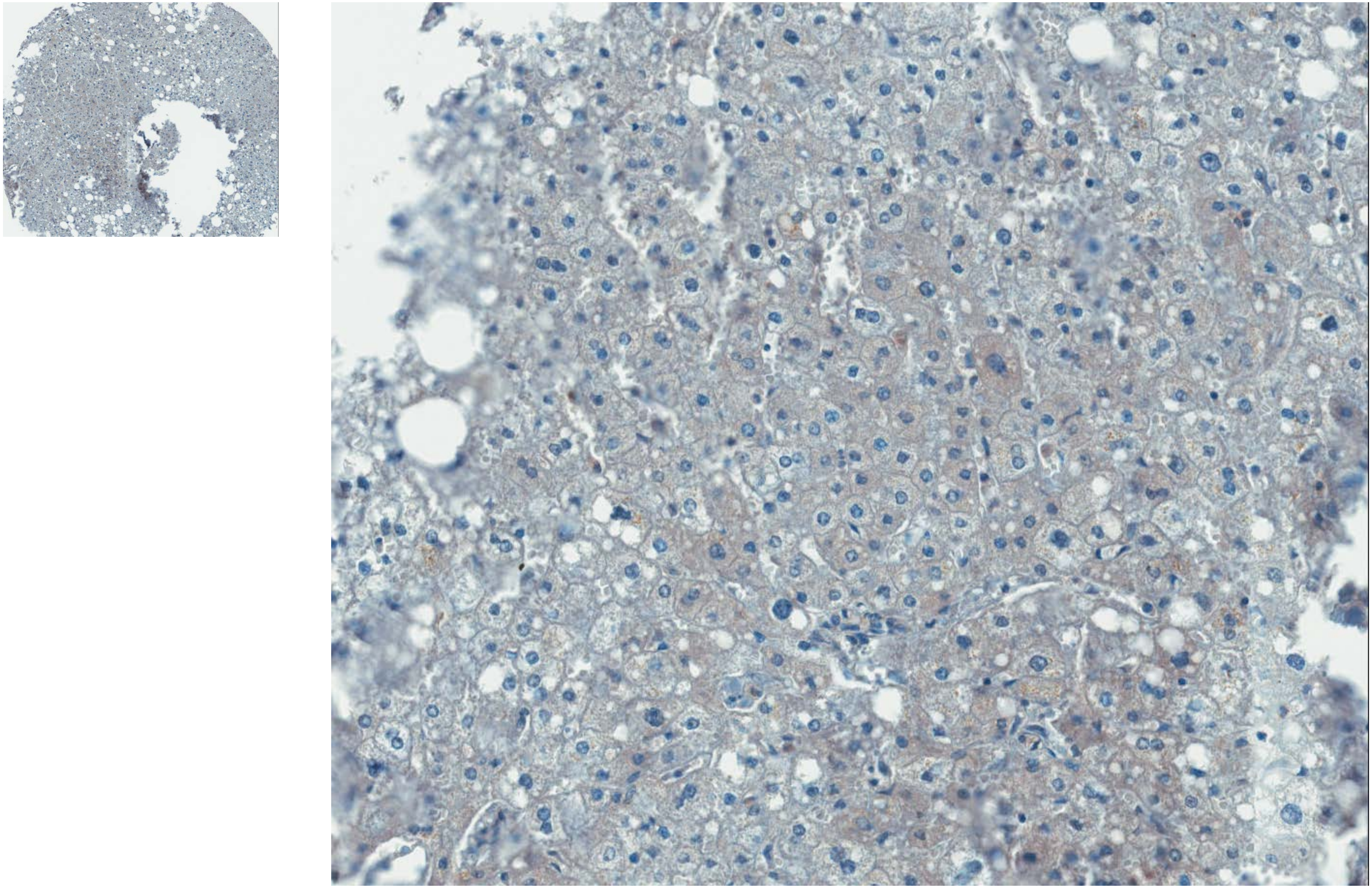

SI Figure 245. Example image of stained tissue from normal liver. IHC was performed on tissue sections using a monoclonal antibody raised against CCK2R.

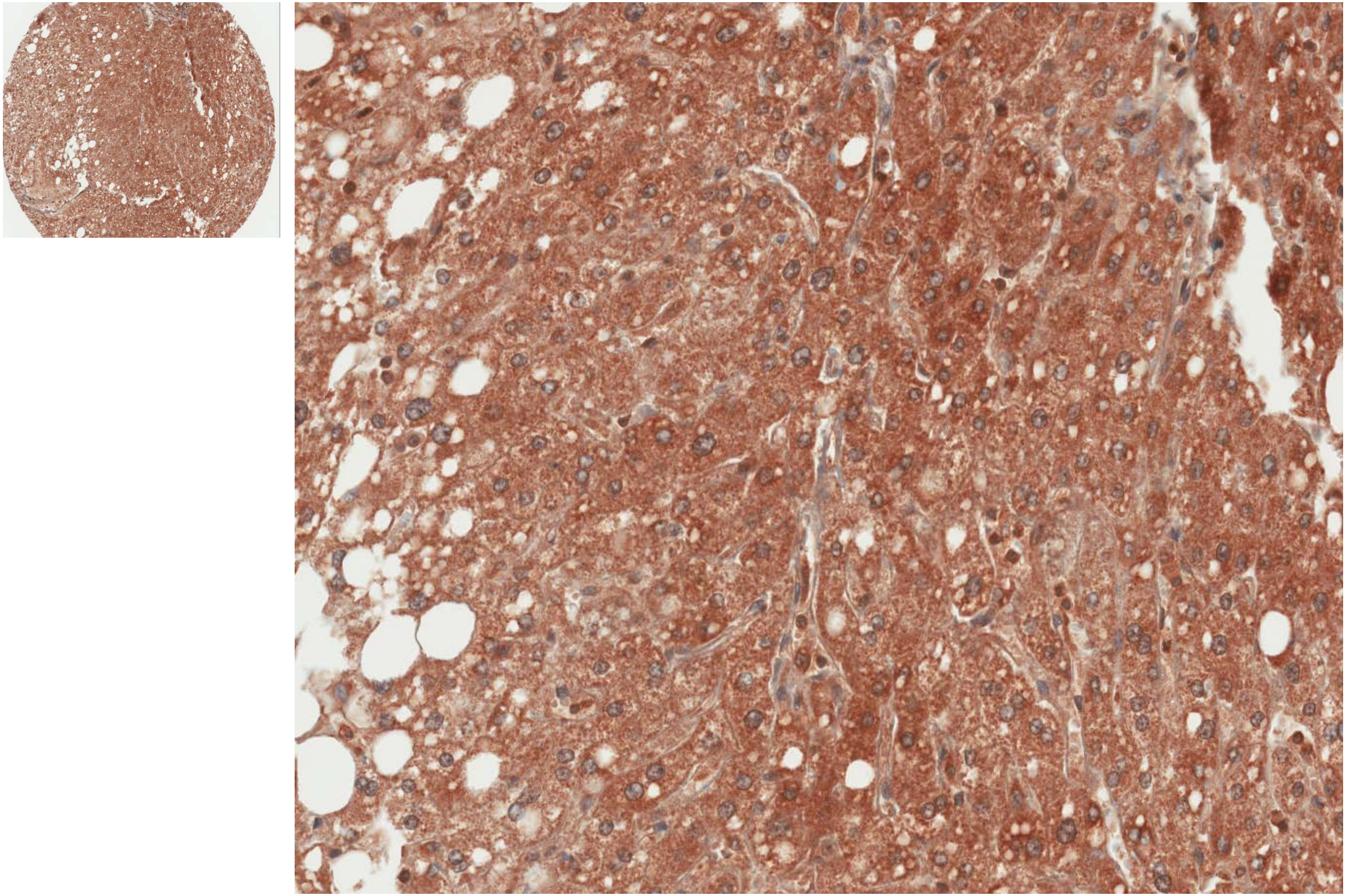

SI Figure 246. Example image of stained tissue from normal liver. IHC was performed on tissue sections using a monoclonal antibody raised against CCK2R.

Normal Liver Tissue Staining Intensity

	n	Average	St. Dev	St. Error
Staining Intensity	22	1.95	1.05	0.22

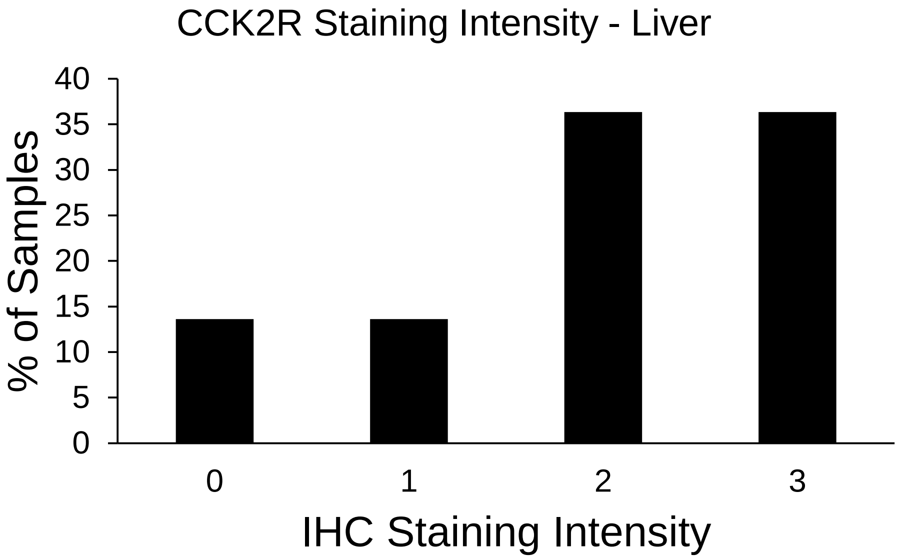

	Staining Intensity			
	0	1	2	3
n	3	3	8	8
%	13.64	13.64	36.36	36.36

SI Figure 247. CCK2R Staining Intensity for normal tissue from the liver. IHC was performed on tissue sections using a monoclonal antibody raised against CCK2R. The intensity of staining was graded on a scale of 0 to 3 and plotted.

Normal Liver Tissue
Coverage Score Correlations

	n	Average	St. Dev	St. Error
Coverage Score	22	2.41	1.05	0.22

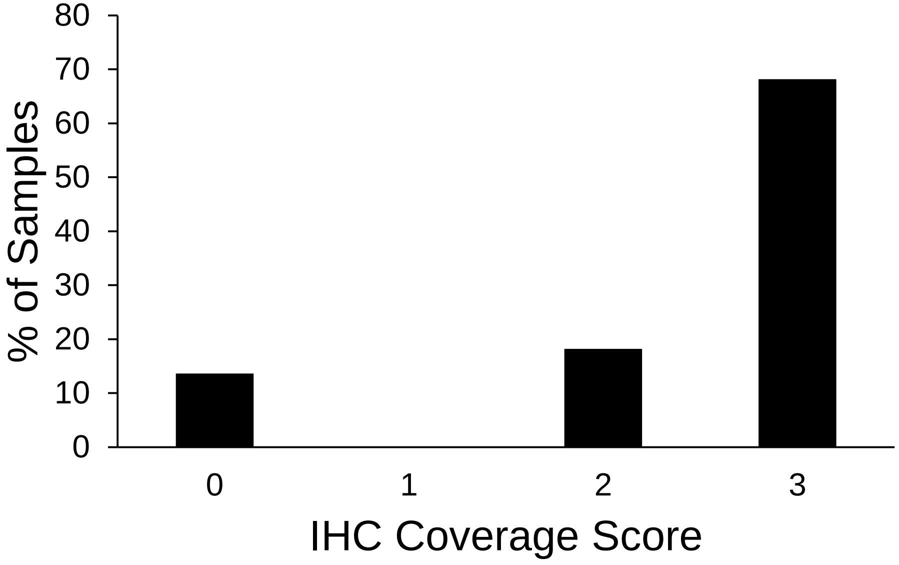

	Coverage Score			
	0	1	2	3
n	3	0	4	15
%	13.64	0.00	18.18	68.18

SI Figure 248. CCK2R Coverage Score for normal tissue from the liver. IHC was performed on tissue sections using a monoclonal antibody raised against CCK2R. The area stained (coverage) was graded on a scale of 0 to 3 and plotted.

Normal Liver Tissue
Total Staining Score Correlations

	n	Average	St. Dev	St. Error
Total Score	22	5.50	3.14	0.67

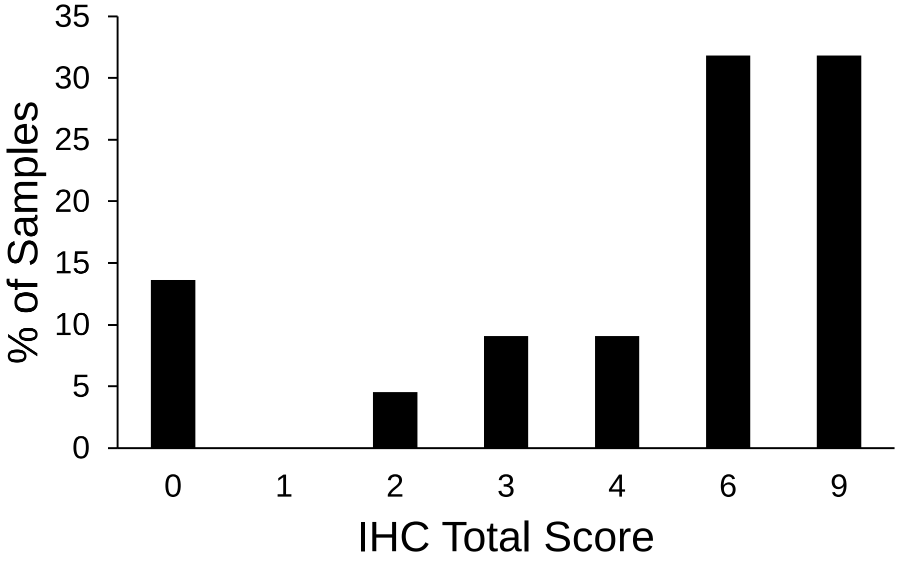

	Total Staining Score						
	0	1	2	3	4	6	9
n	3	0	1	2	2	7	7
%	13.64	0.00	4.55	9.09	9.09	31.82	31.82

SI Figure 249. CCK2R Total Staining Score for normal tissue from the liver. IHC was performed on tissue sections using a monoclonal antibody raised against CCK2R. The staining intensity and coverage score was multiplied to obtain the total staining score.

Normal Lung

Normal Lung Tissue Images

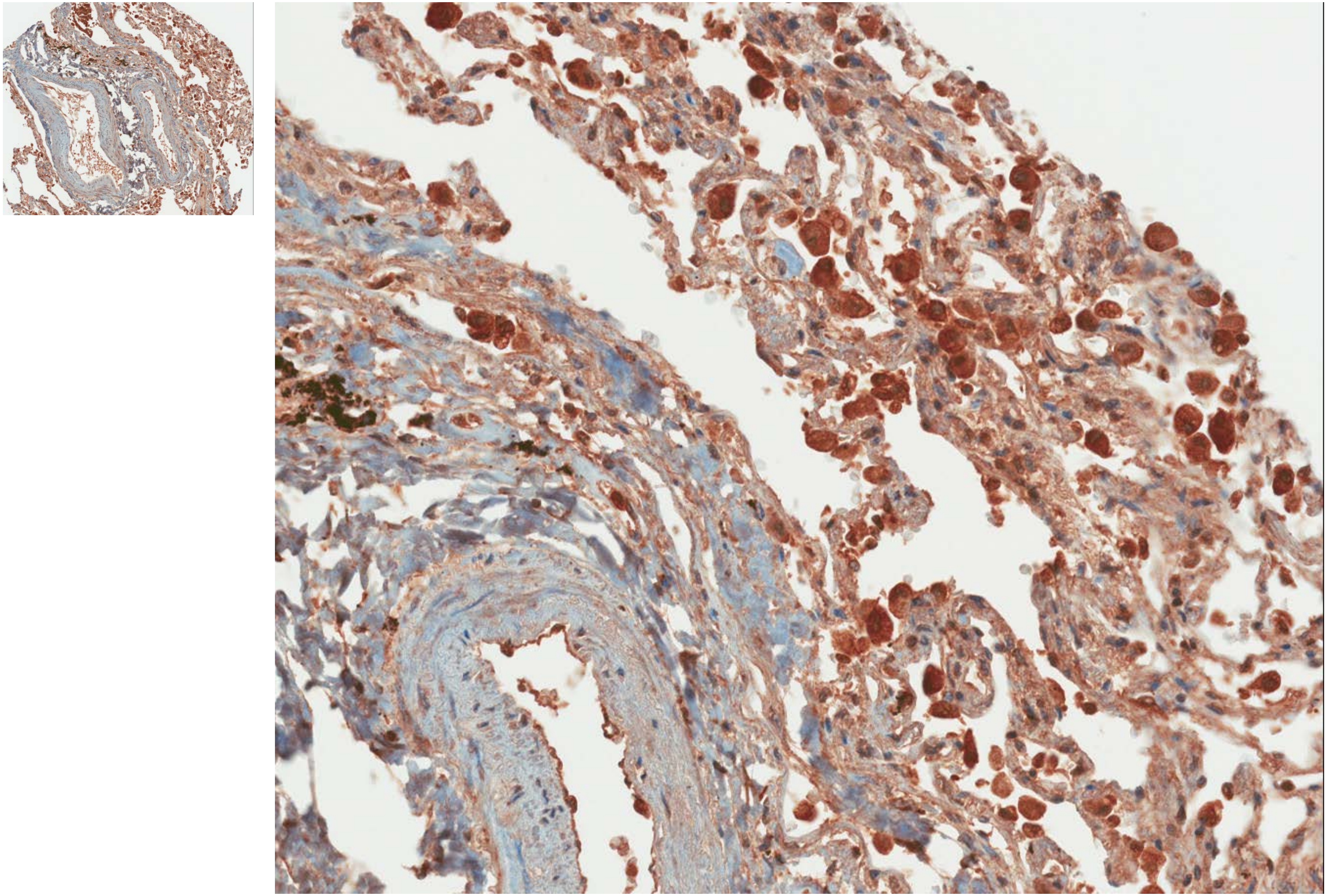

SI Figure 250. Example image of stained normal tissue from lung. IHC was performed on tissue sections using a monoclonal antibody raised against CCK2R.

Normal Lung Tissue
Staining Intensity

	n	Average	St. Dev	St. Error
Staining Intensity	8	0.38	1.06	0.38

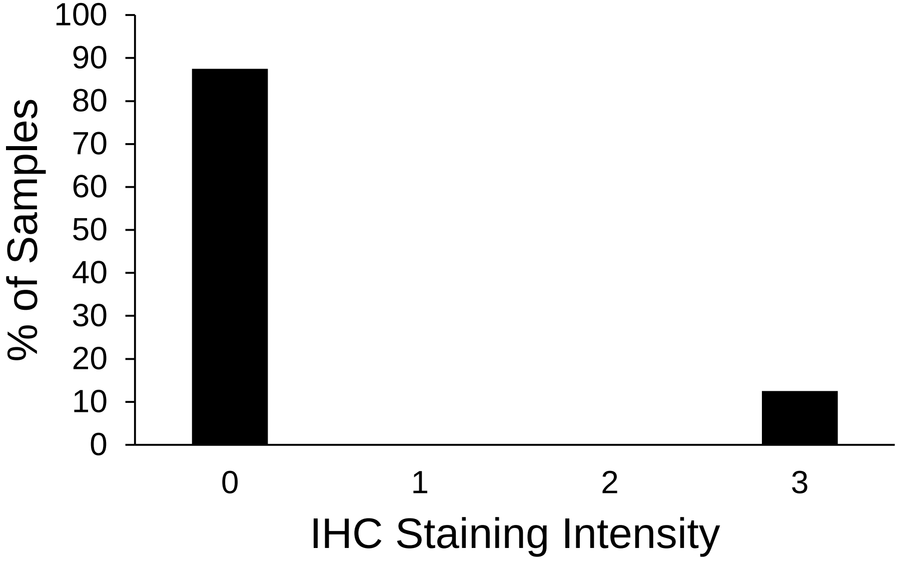

	Staining Intensity			
	0	1	2	3
n	7	0	0	1
%	87.50	0.00	0.00	12.50

SI Figure 251. CCK2R Staining Intensity for normal tissue from the lung. IHC was performed on tissue sections using a monoclonal antibody raised against CCK2R. The intensity of staining was graded on a scale of 0 to 3 and plotted.

Normal Lung Tissue
Coverage Score Correlations

	n	Average	St. Dev	St. Error
Coverage Score	8	0.38	1.06	0.38

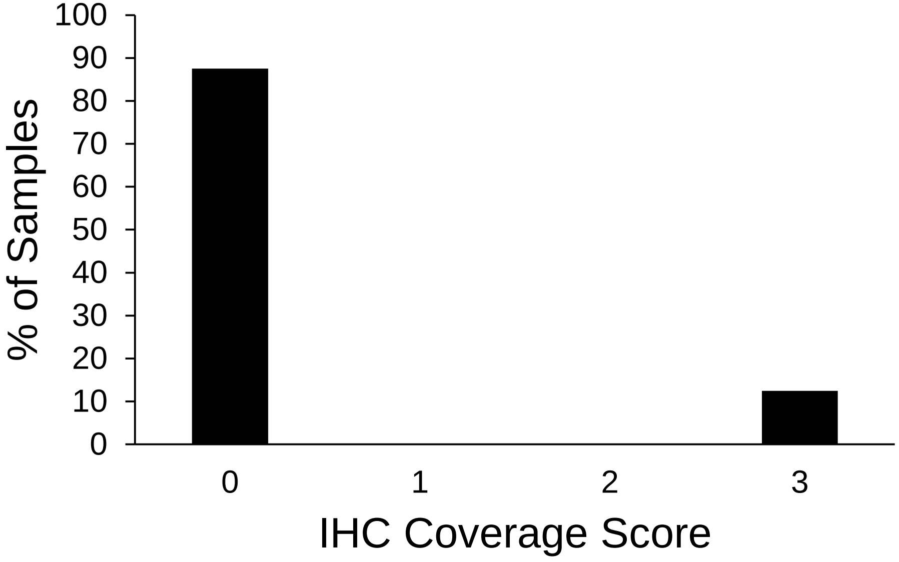

	Coverage Score			
	0	1	2	3
n	7	0	0	1
%	87.50	0.00	0.00	12.50

SI Figure 252. CCK2R Coverage Score for normal tissue from the lung. IHC was performed on tissue sections using a monoclonal antibody raised against CCK2R. The area stained (coverage) was graded on a scale of 0 to 3 and plotted.

Normal Lung Tissue
Total Staining Score Correlations

	n	Average	St. Dev	St. Error
Total Score	8	1.13	3.18	1.13

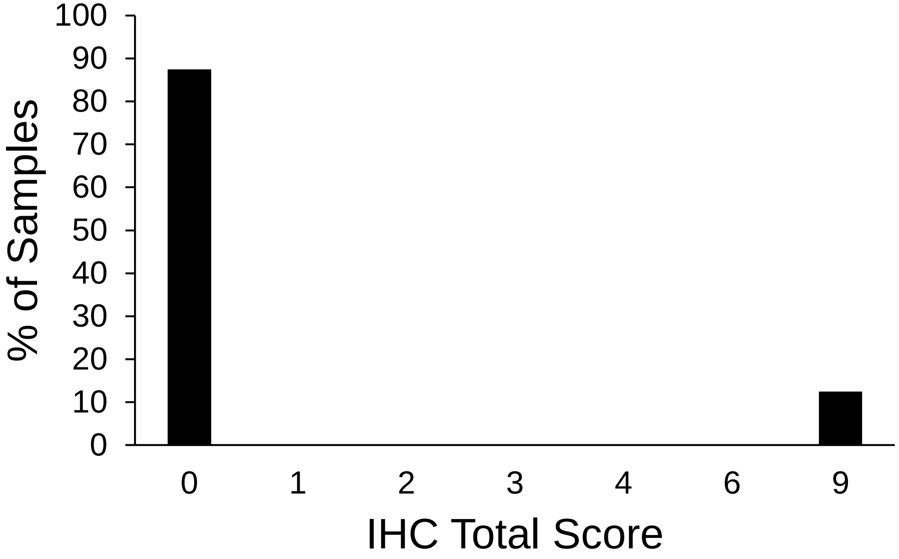

	Total Staining Score						
	0	1	2	3	4	6	9
n	7	0	0	0	0	0	1
%	87.50	0.00	0.00	0.00	0.00	0.00	12.50

SI Figure 253. CCK2R Total Staining Score for normal tissue from the lung. IHC was performed on tissue sections using a monoclonal antibody raised against CCK2R. The staining intensity and coverage score was multiplied to obtain the total staining score.

Normal Pancreas

Normal Pancreas Tissue Images

Large Duct

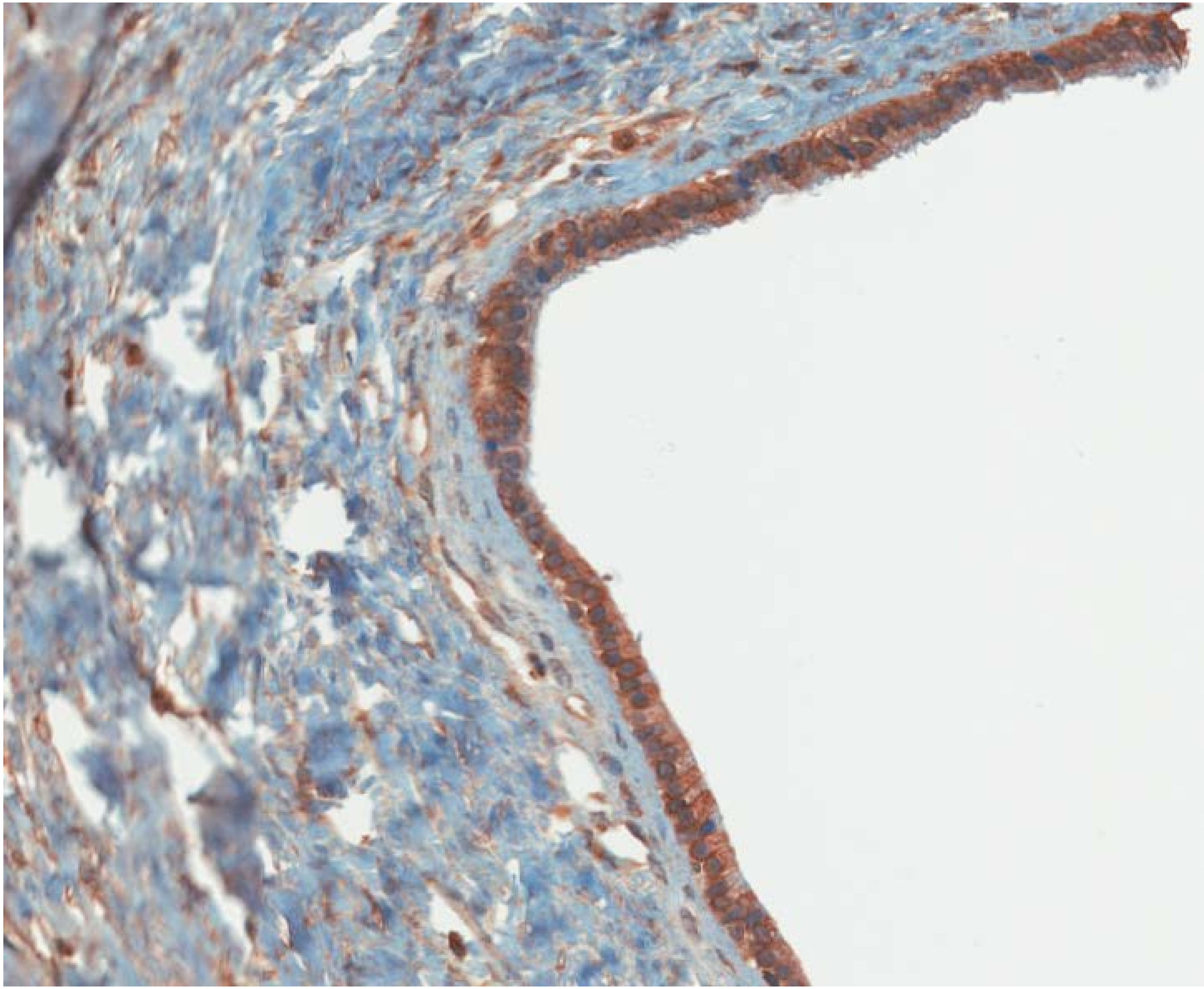

SI Figure 254. Example image of stained tissue from pancreas containing cells of the large duct. IHC was performed on tissue sections using a monoclonal antibody raised against CCK2R.

Intercalated Duct

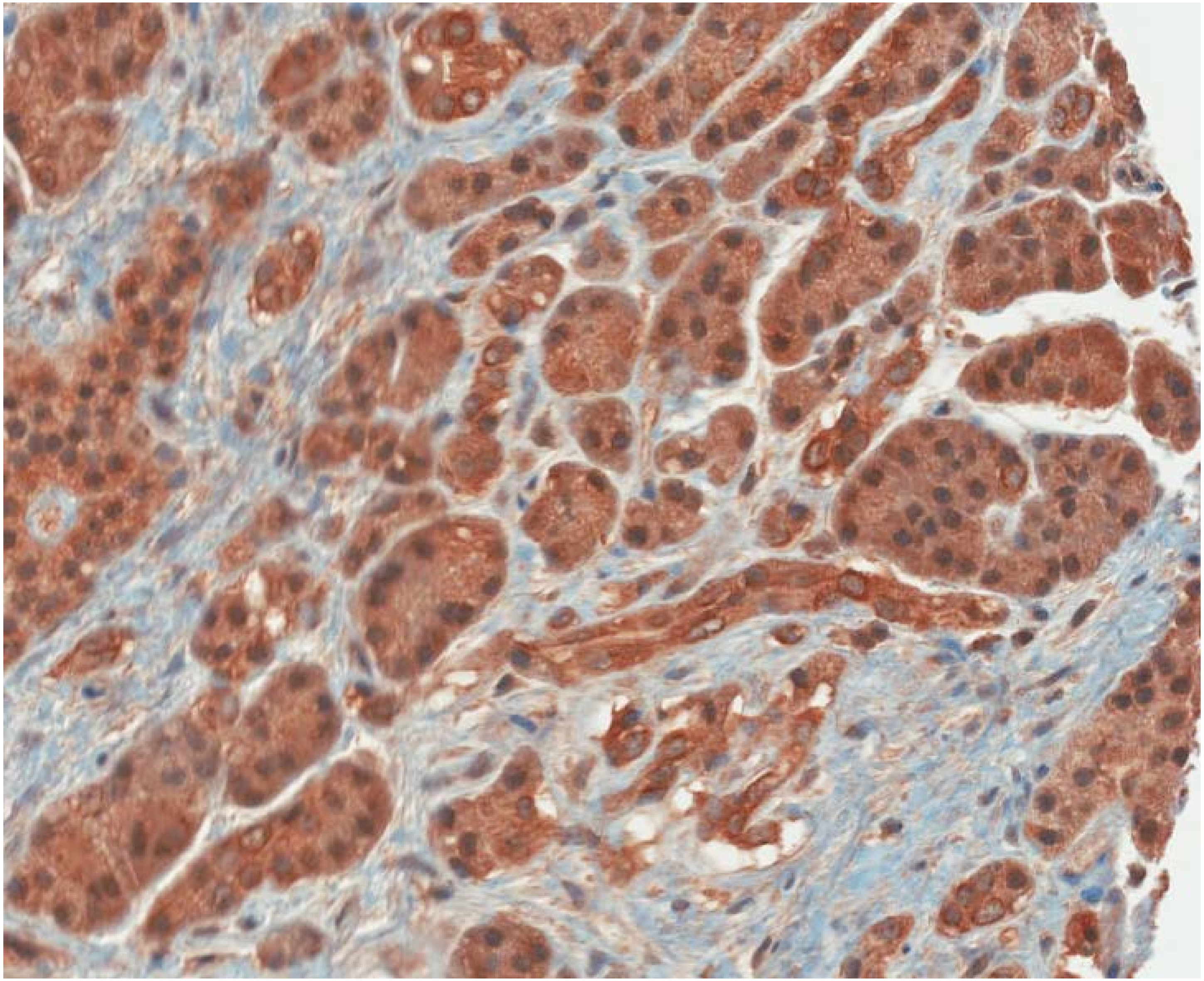

SI Figure 255. Example image of stained tissue from pancreas containing cells of the intercalated duct. IHC was performed on tissue sections using a monoclonal antibody raised against CCK2R.

Intercalated Cells

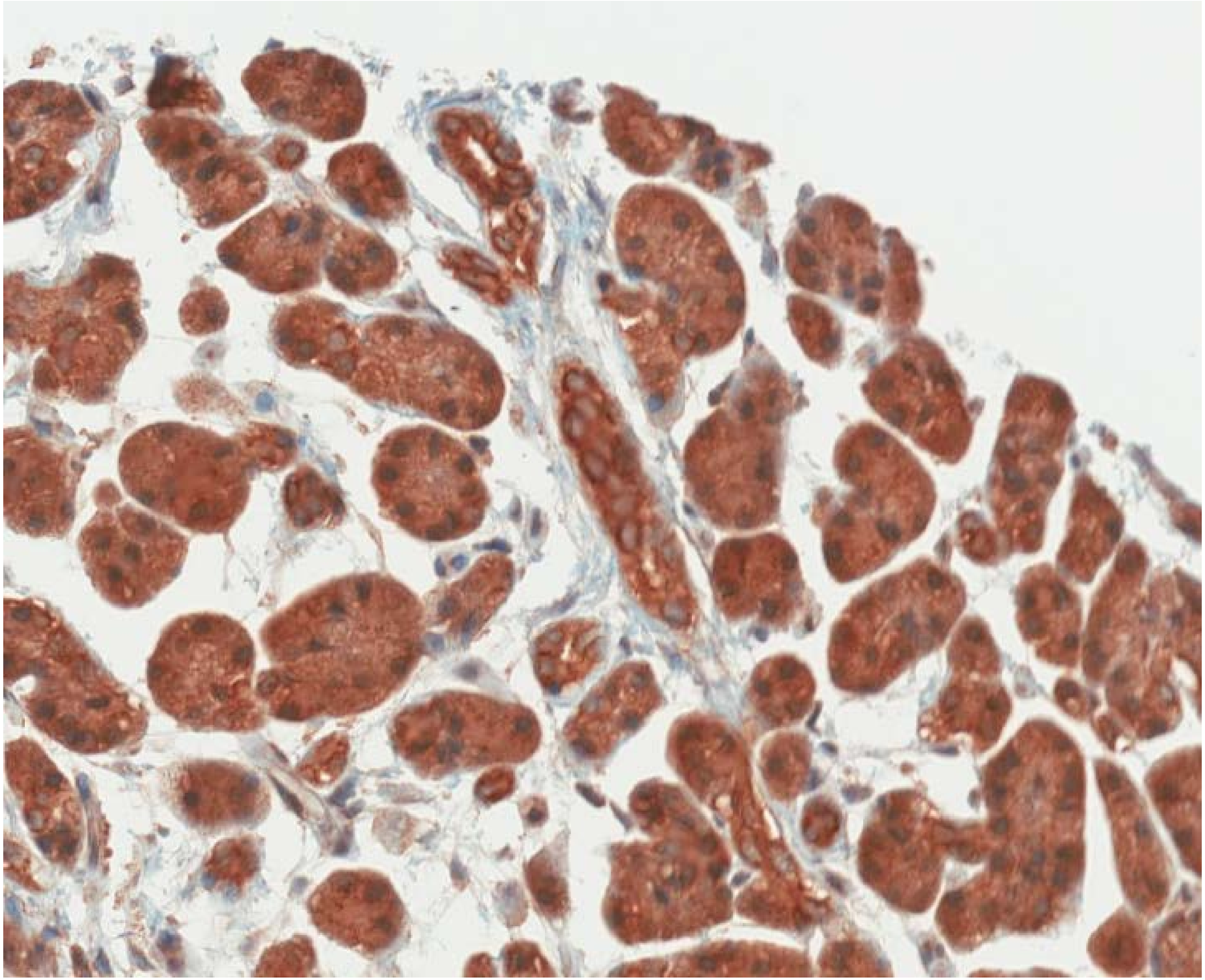

SI Figure 256. Example image of stained tissue from pancreas containing intercalated cells. IHC was performed on tissue sections using a monoclonal antibody raised against CCK2R.

Acinar Cells

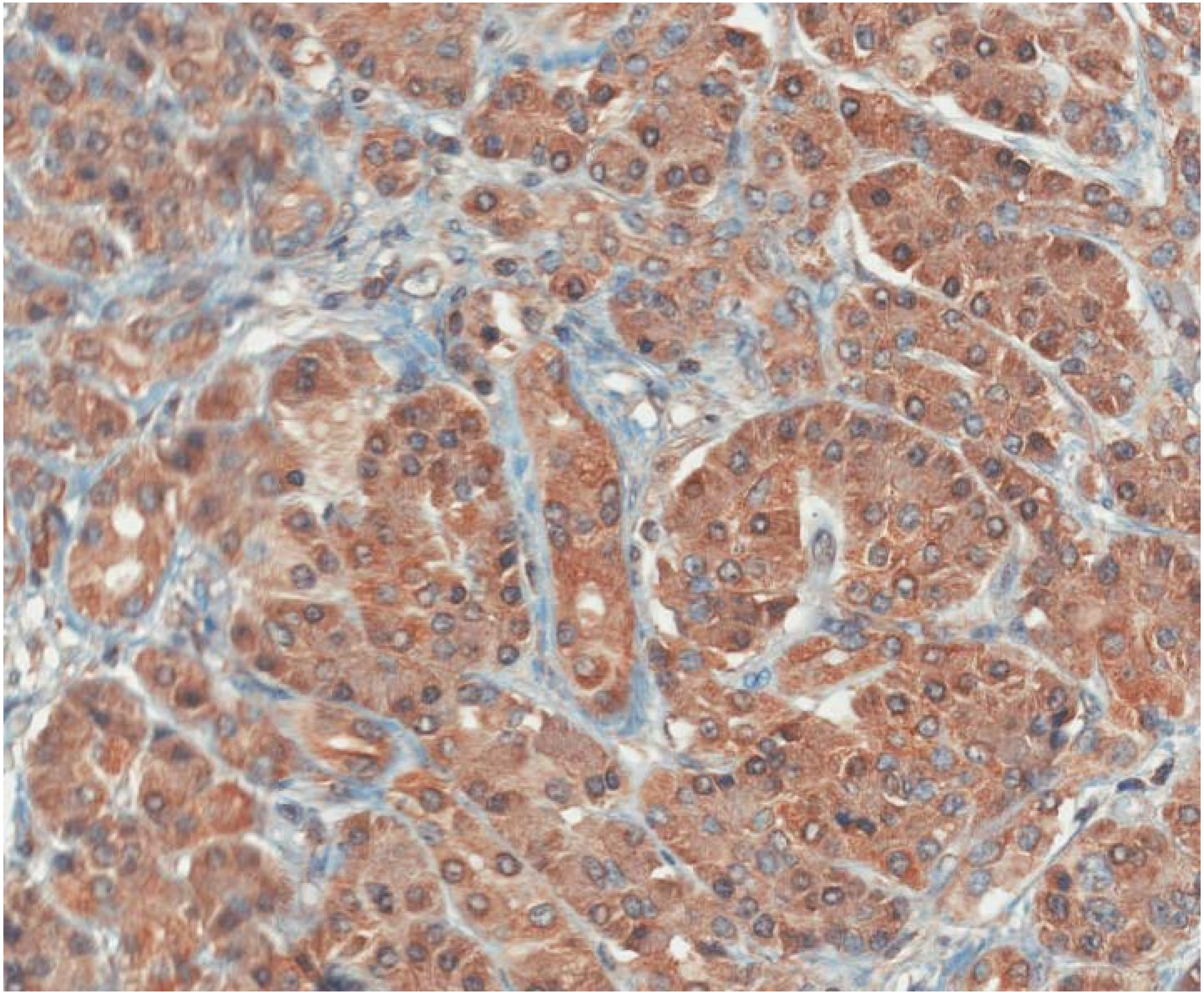

SI Figure 257. Example image of stained tissue from pancreas containing acinar cells. IHC was performed on tissue sections using a monoclonal antibody raised against CCK2R.

Benign Acini

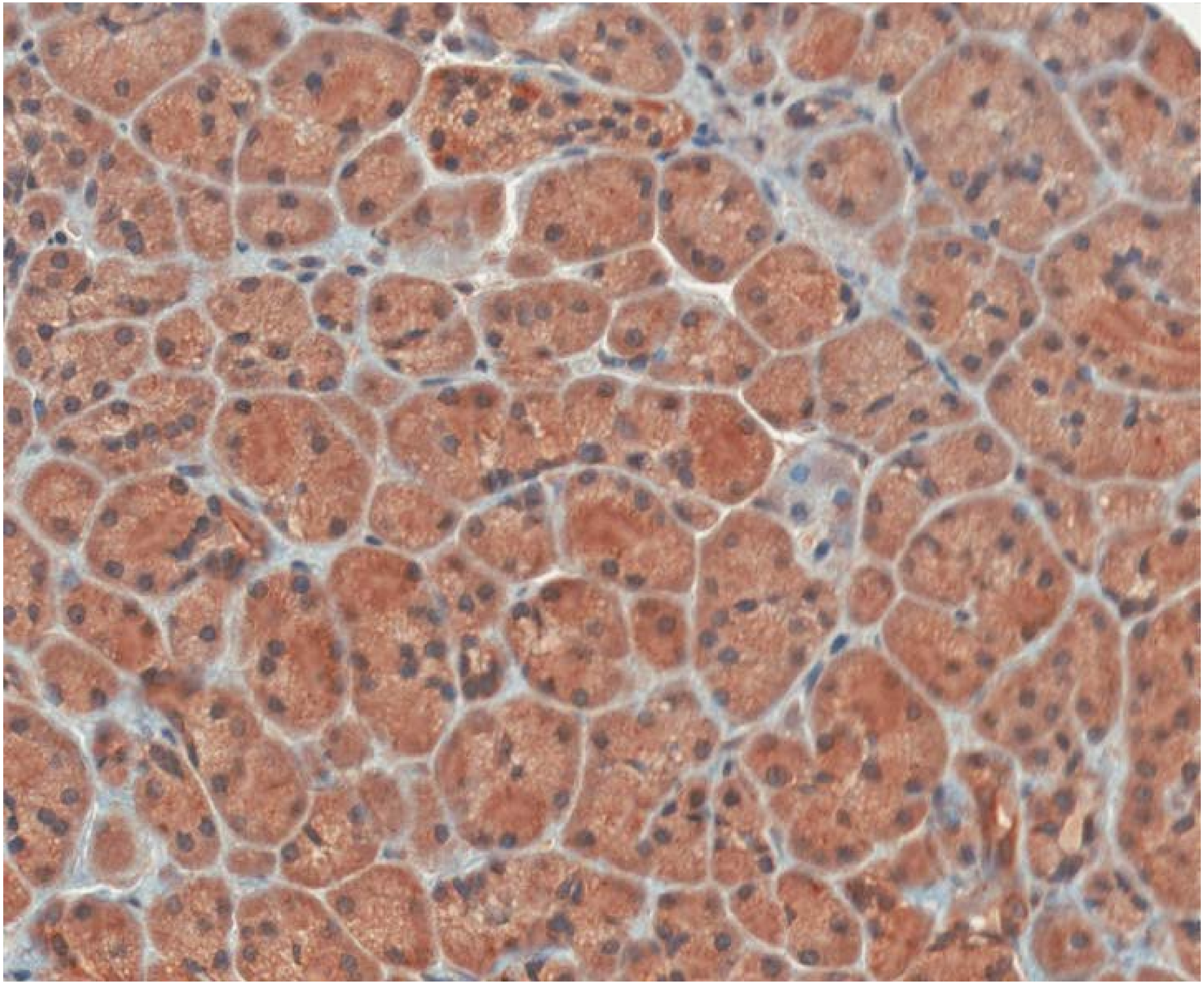

SI Figure 258. Example image of stained tissue from pancreas containing benign acini cells. IHC was performed on tissue sections using a monoclonal antibody raised against CCK2R.

Atrophic Acini

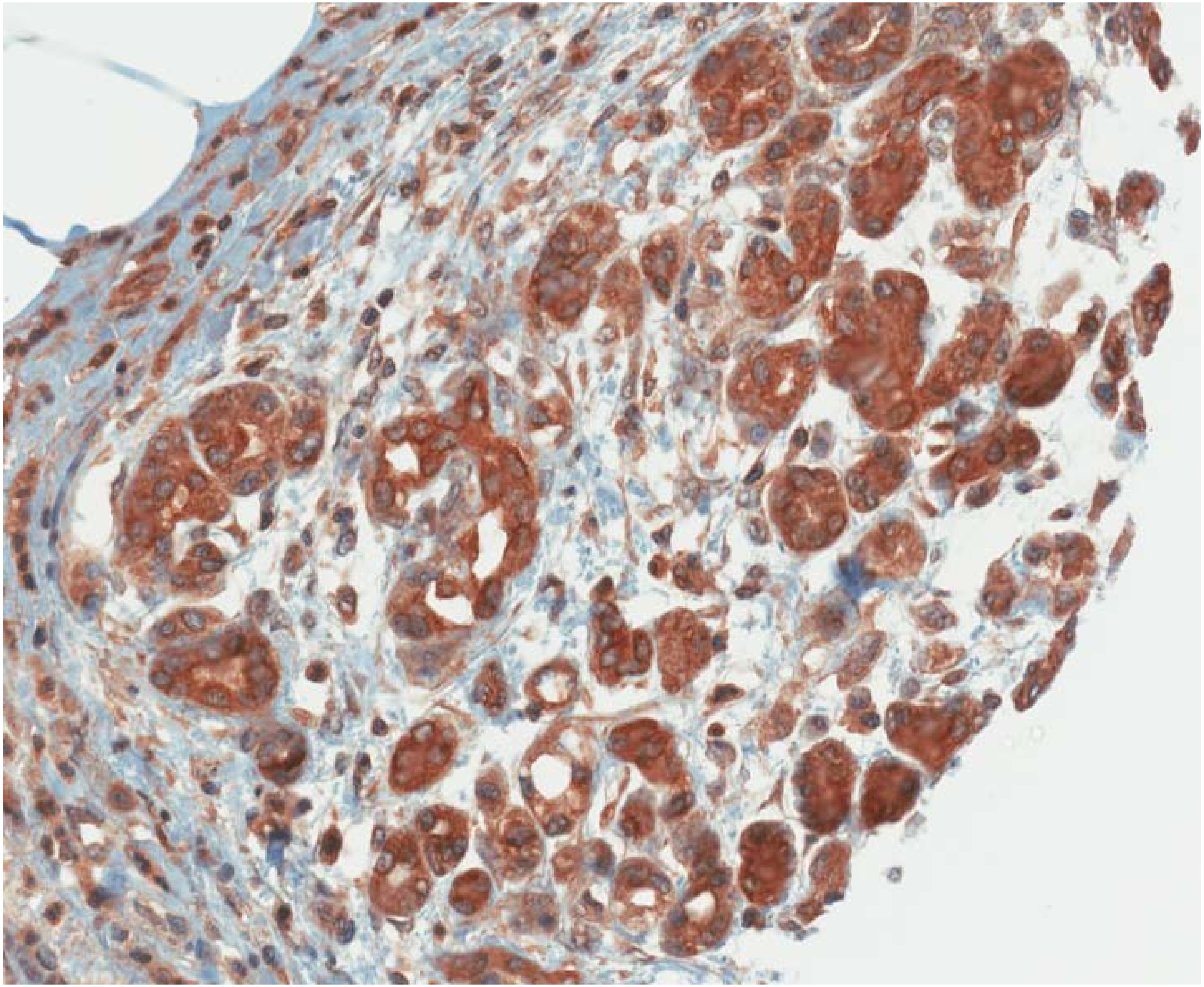

SI Figure 259. Example image of stained tissue from pancreas containing atrophic acini cells. IHC was performed on tissue sections using a monoclonal antibody raised against CCK2R.

Normal Pancreas Tissue Staining Intensity

	n	Average	St. Dev	St. Error
Staining Intensity	66	2.52	0.66	0.08

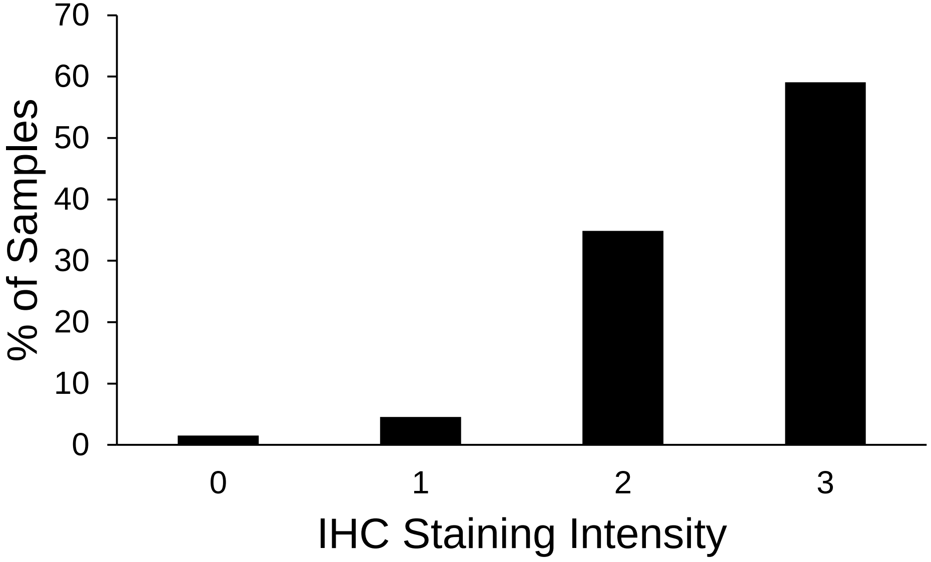

	Staining Intensity			
	0	1	2	3
n	1	3	23	39
%	1.52	4.55	34.85	59.09

SI Figure 260. CCK2R Staining Intensity for normal tissue from the pancreas. IHC was performed on tissue sections using a monoclonal antibody raised against CCK2R. The intensity of staining was graded on a scale of 0 to 3 and plotted.

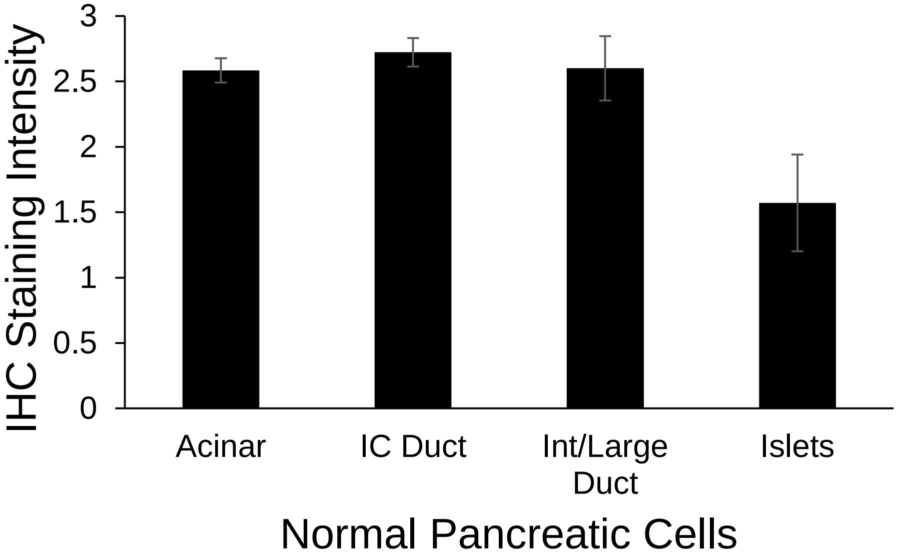

	Intensity Score			
	n	Average	St. Dev.	SEM
Acinar	36	2.58	0.55	0.09
IC Duct	18	2.72	0.46	0.11
Int/Large Duct	5	2.60	0.55	0.24
Islets	7	1.57	0.98	0.37

SI Figure 261. CCK2R Staining Intensity for normal tissue from cell types within the pancreas. IHC was performed on tissue sections using a monoclonal antibody raised against CCK2R. The intensity of staining was graded on a scale of 0 to 3 and plotted.

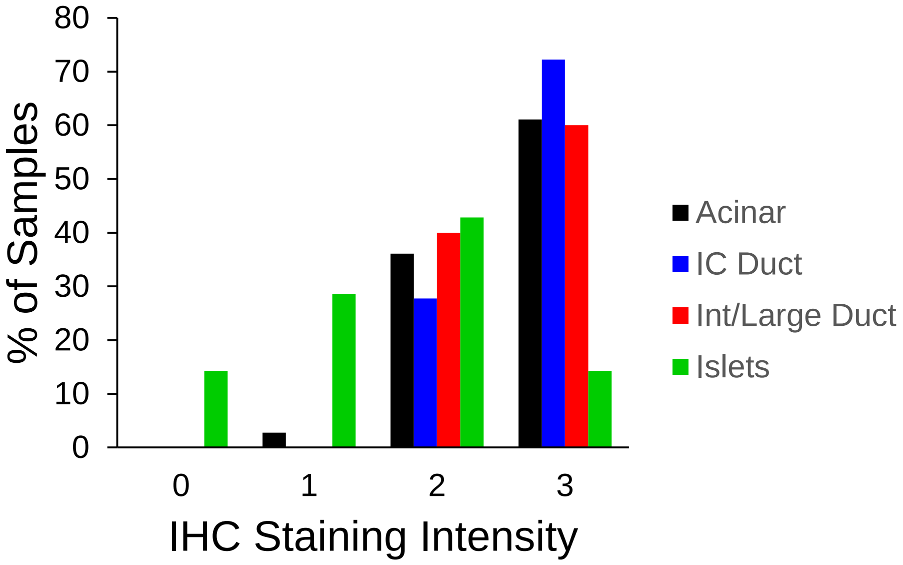

Staining Intensity (n in each group)

	0	1	2	3
Acinar	0	1	13	22
IC Duct	0	0	5	13
Int/Large Duct	0	0	2	3
Islets	1	2	3	1

Staining Intensity (% in each group)

	0	1	2	3
Acinar	0.00	2.78	36.11	61.11
IC Duct	0.00	0.00	27.78	72.22
Int/Large Duct	0.00	0.00	40.00	60.00
Islets	14.29	28.57	42.86	14.29

SI Figure 262. CCK2R Staining Intensity for normal tissue from cell types within the pancreas. IHC was performed on tissue sections using a monoclonal antibody raised against CCK2R. The intensity of staining was graded on a scale of 0 to 3 and plotted.

Normal Pancreas Tissue Coverage Score Correlations

	n	Average	St. Dev	St. Error
Coverage Score	66	2.95	0.37	0.05

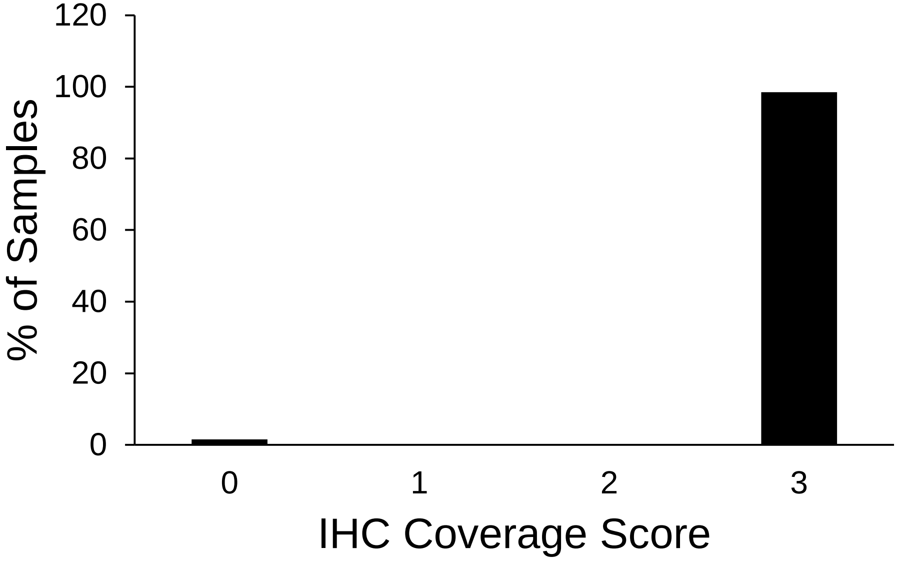

	Coverage Score			
	0	1	2	3
n	1	0	0	65
%	1.52	0.00	0.00	98.48

SI Figure 263. CCK2R Coverage Score for normal tissue from the pancreas. IHC was performed on tissue sections using a monoclonal antibody raised against CCK2R. The area stained (coverage) was graded on a scale of 0 to 3 and plotted.

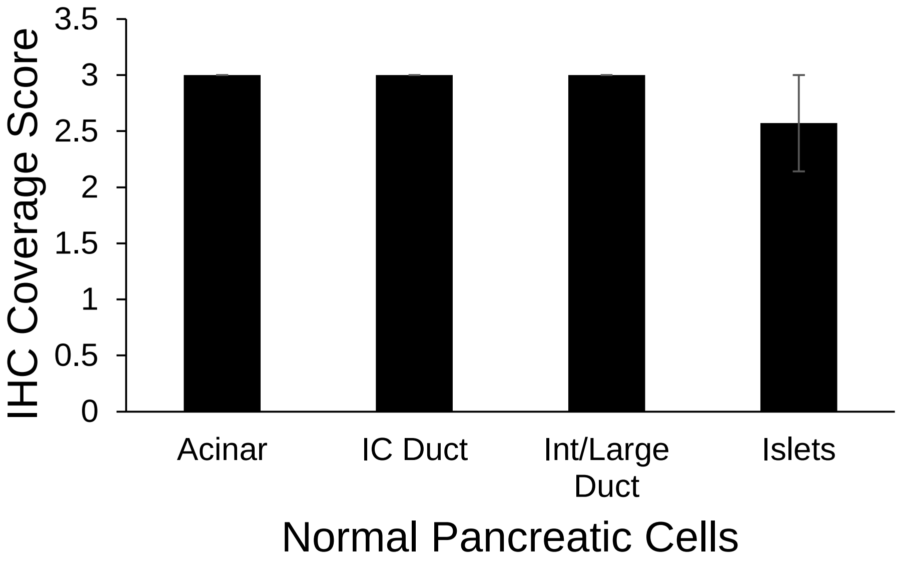

		Coverage Score		
	n	Average	St. Dev.	SEM
Acinar	36	3.00	0.00	0.00
IC Duct	18	3.00	0.00	0.00
Int/Large Duct	5	3.00	0.00	0.00
Islets	7	2.57	1.13	0.43

SI Figure 264. CCK2R Coverage Score for normal tissue from cell types within the pancreas. IHC was performed on tissue sections using a monoclonal antibody raised against CCK2R. The intensity of staining was graded on a scale of 0 to 3 and plotted.

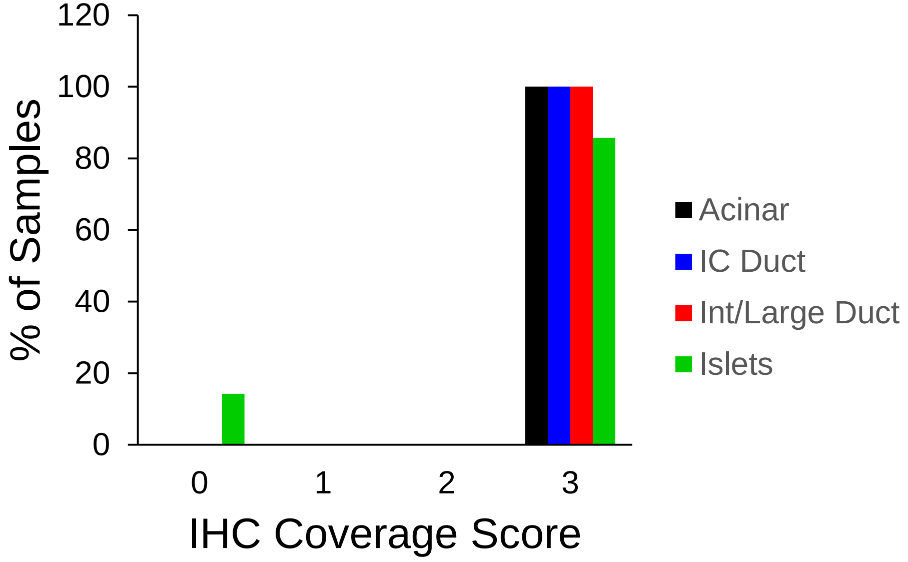

Coverage Score (n in each group)	0	1	2	3
Acinar	0	0	0	36
IC Duct	0	0	0	18
Int/Large Duct	0	0	0	5
Islets	1	0	0	6

Coverage Score (% in each group)	0	1	2	3
Acinar	0.00	0.00	0.00	100.00
IC Duct	0.00	0.00	0.00	100.00
Int/Large Duct	0.00	0.00	0.00	100.00
Islets	14.29	0.00	0.00	85.71

SI Figure 265. CCK2R Coverage Score for normal tissue from cell types within the pancreas. IHC was performed on tissue sections using a monoclonal antibody raised against CCK2R. The intensity of staining was graded on a scale of 0 to 3 and plotted.

Normal Pancreas Tissue
Total Staining Score Correlations

	n	Average	St. Dev	St. Error
Total Score	66	7.55	1.99	0.24

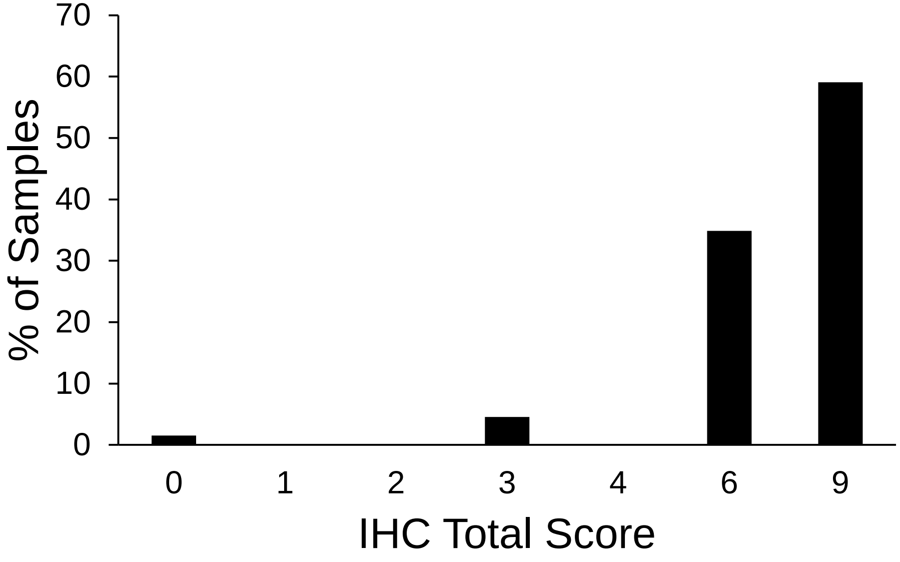

	Total Staining Score						
	0	1	2	3	4	6	9
n	1	0	0	3	0	23	39
%	1.52	0.00	0.00	4.55	0.00	34.85	59.09

SI Figure 266. CCK2R Total Staining Score for normal tissue from the pancreas. IHC was performed on tissue sections using a monoclonal antibody raised against CCK2R. The staining intensity and coverage score was multiplied to obtain the total staining score.

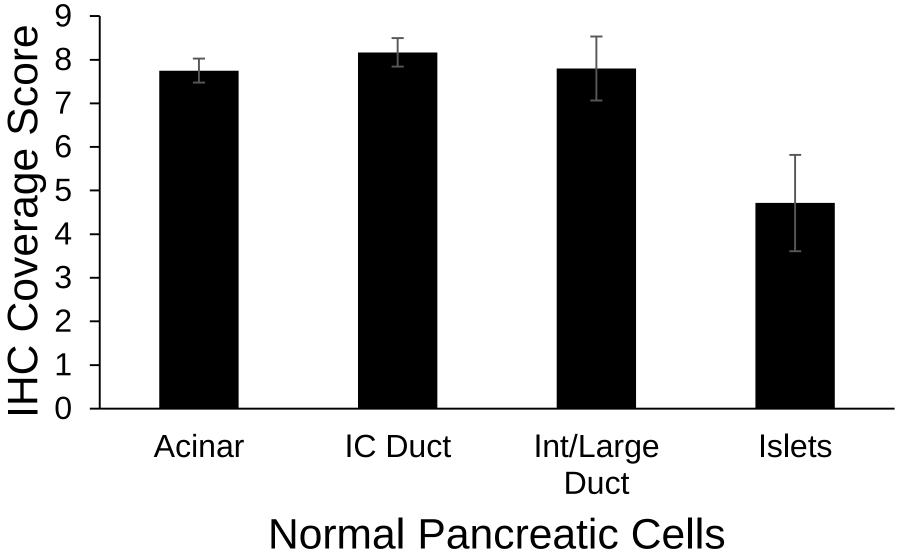

	Total Score			
	n	Average	St. Dev.	SEM
Acinar	36	7.75	1.66	0.28
IC Duct	18	8.17	1.38	0.33
Int/Large Duct	5	7.80	1.64	0.73
Islets	7	4.71	2.93	1.11

SI Figure 267. CCK2R Total Staining Score for normal tissue from cell types within the pancreas. IHC was performed on tissue sections using a monoclonal antibody raised against CCK2R. The total staining score was derived by multiplying the staining intensity and the coverage score and plotted.

	Total Staining Score (n in each group)						
	0	1	2	3	4	6	9
Acinar	0	0	0	1	0	13	22
IC Duct	0	0	0	0	0	5	13
Int/Large Duct	0	0	0	0	0	2	3
Islets	1	0	0	2	0	3	1

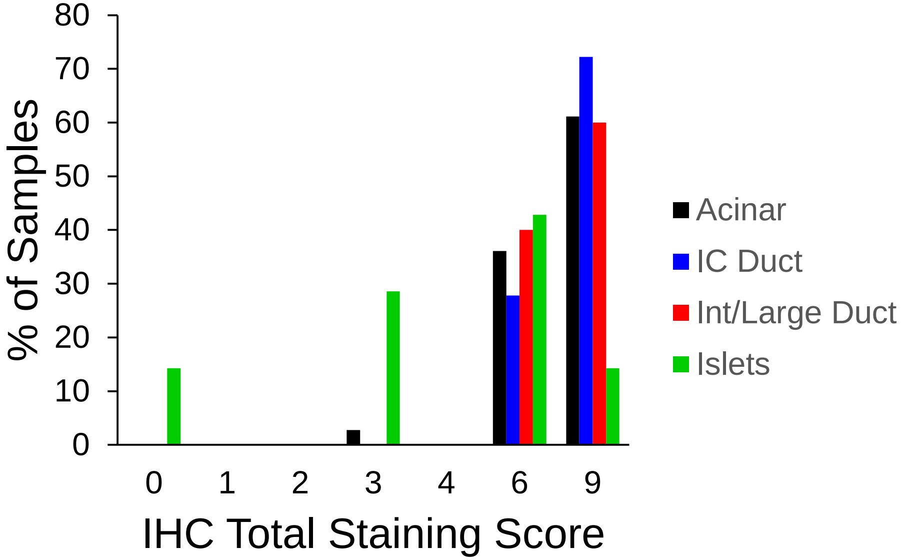

	Total Staining Score (% in each group)						
	0	1	2	3	4	6	9
Acinar	0.00	0.00	0.00	2.78	0.00	36.11	61.11
IC Duct	0.00	0.00	0.00	0.00	0.00	27.78	72.22
Int/Large Duct	0.00	0.00	0.00	0.00	0.00	40.00	60.00
Islets	14.29	0.00	0.00	28.57	0.00	42.86	14.29

SI Figure 268. CCK2R Total Staining Score for normal tissue from cell types within the pancreas. IHC was performed on tissue sections using a monoclonal antibody raised against CCK2R. The total staining score was derived by multiplying the staining intensity and the coverage score and plotted.

Normal Spleen

Normal Spleen Tissue Staining Intensity

	n	Average	St. Dev	St. Error
Staining Intensity	6	1.33	0.52	0.21

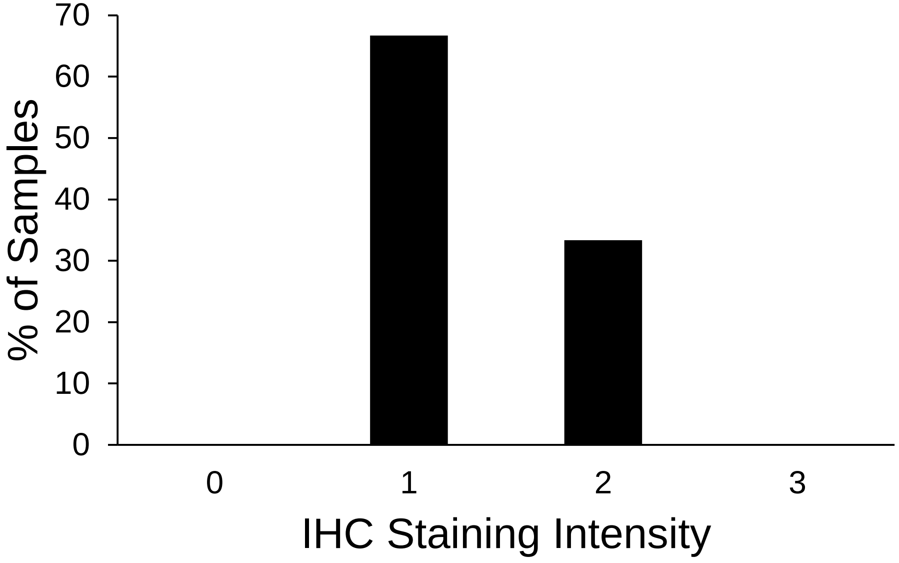

	Staining Intensity			
	0	1	2	3
n	0	4	2	0
%	0.00	66.67	33.33	0.00

SI Figure 269. CCK2R Staining Intensity for normal tissue from the spleen. IHC was performed on tissue sections using a monoclonal antibody raised against CCK2R. The intensity of staining was graded on a scale of 0 to 3 and plotted.

Normal Spleen Tissue
Coverage Score Correlations

	n	Average	St. Dev	St. Error
Coverage Score	6	3.00	0.00	0.00

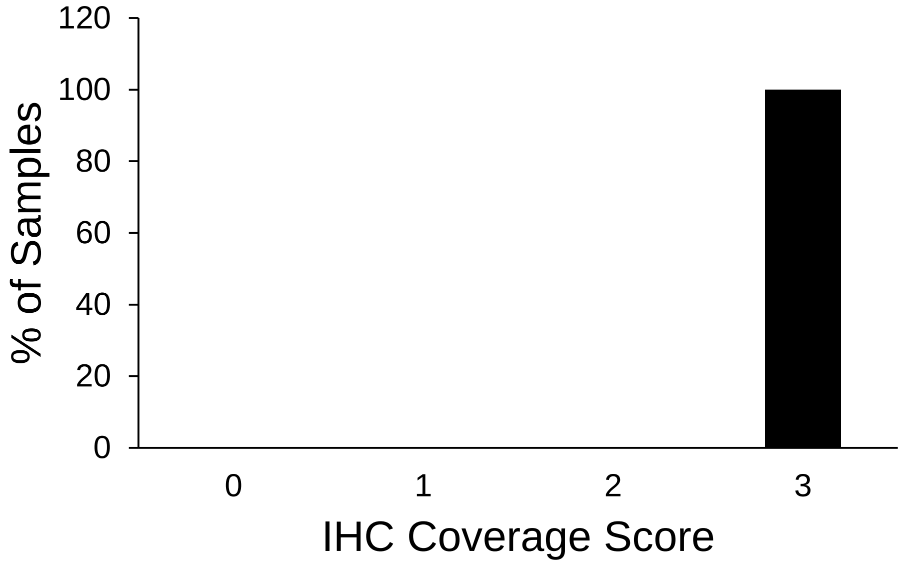

	Coverage Score			
	0	1	2	3
n	0	0	0	6
%	0.00	0.00	0.00	100.00

SI Figure 270. CCK2R Coverage Score for normal tissue from the spleen. IHC was performed on tissue sections using a monoclonal antibody raised against CCK2R. The area stained (coverage) was graded on a scale of 0 to 3 and plotted.

Normal Spleen Tissue
Total Staining Score Correlations

	n	Average	St. Dev	St. Error
Total Score	6	4.00	1.55	0.63

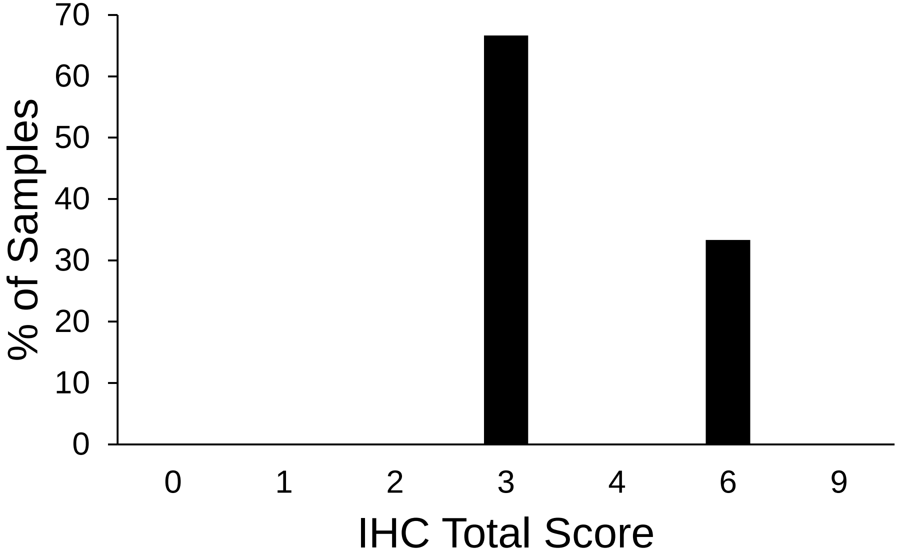

	Total Staining Score						
	0	1	2	3	4	6	9
n	0	0	0	4	0	2	0
%	0.00	0.00	0.00	66.67	0.00	33.33	0.00

SI Figure 271. CCK2R Total Staining Score for normal tissue from the spleen. IHC was performed on tissue sections using a monoclonal antibody raised against CCK2R. The staining intensity and coverage score was multiplied to obtain the total staining score.

Normal Stomach

Normal Stomach Tissue Images

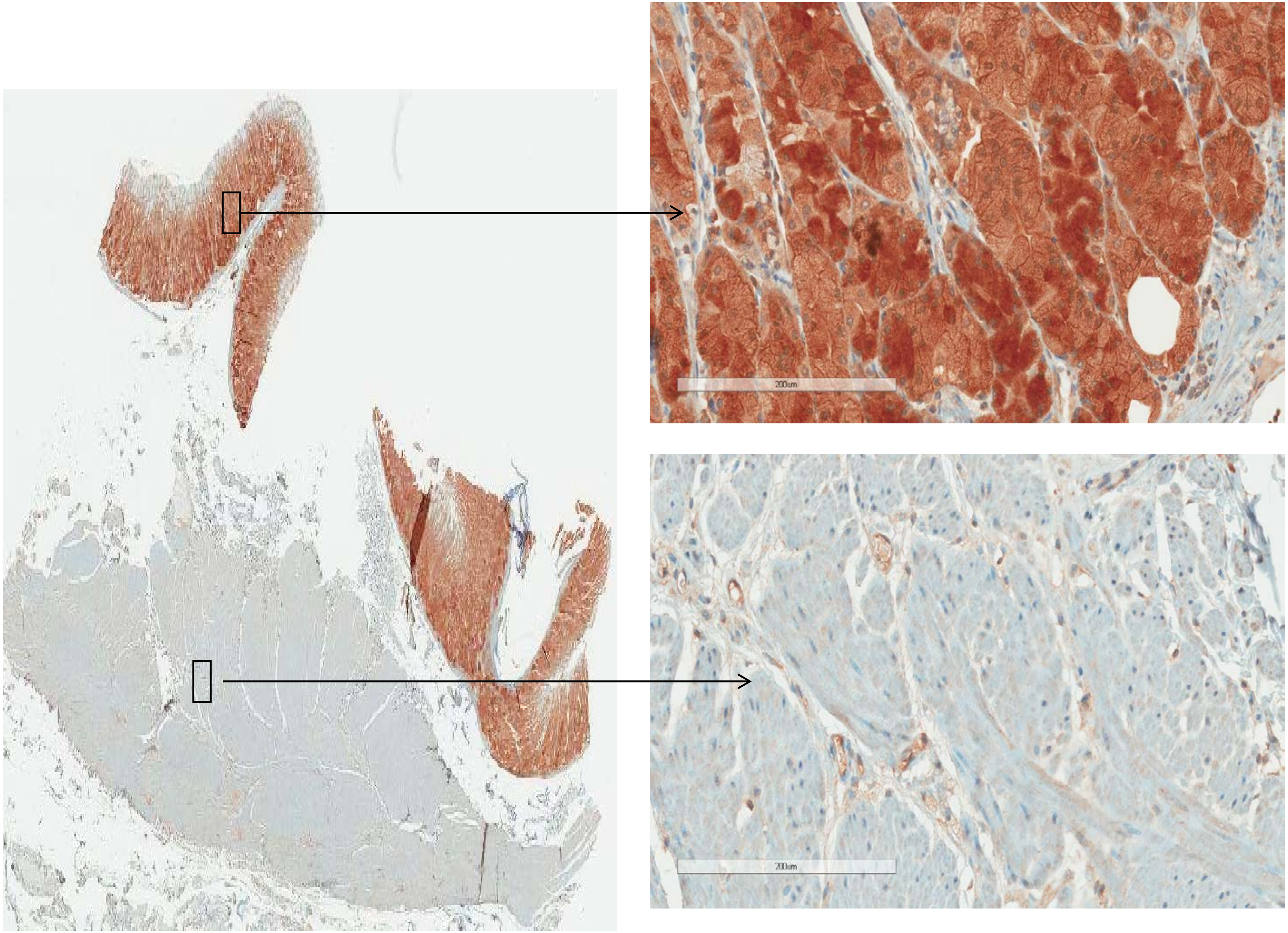

SI Figure 272. Example image of normal stained tissue from stomach. IHC was performed on tissue sections using a monoclonal antibody raised against CCK2R.

Normal Stomach, inset stroma pathology total score = 0

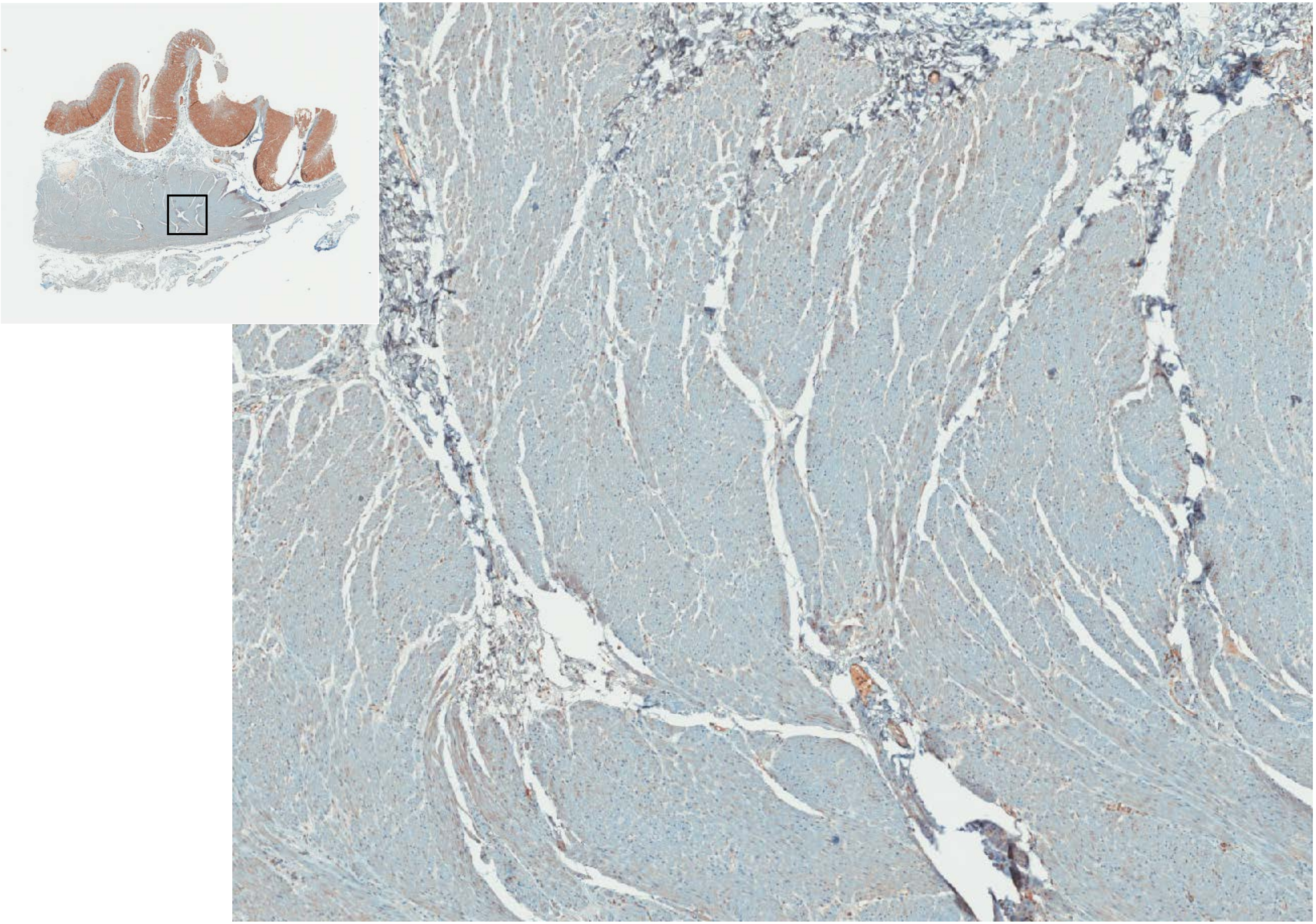

SI Figure 273. Example image of normal stained tissue from stomach. IHC was performed on tissue sections using a monoclonal antibody raised against CCK2R. Inset has a magnification of 4x.

Normal Stomach, inset epithelium pathology total score = 6

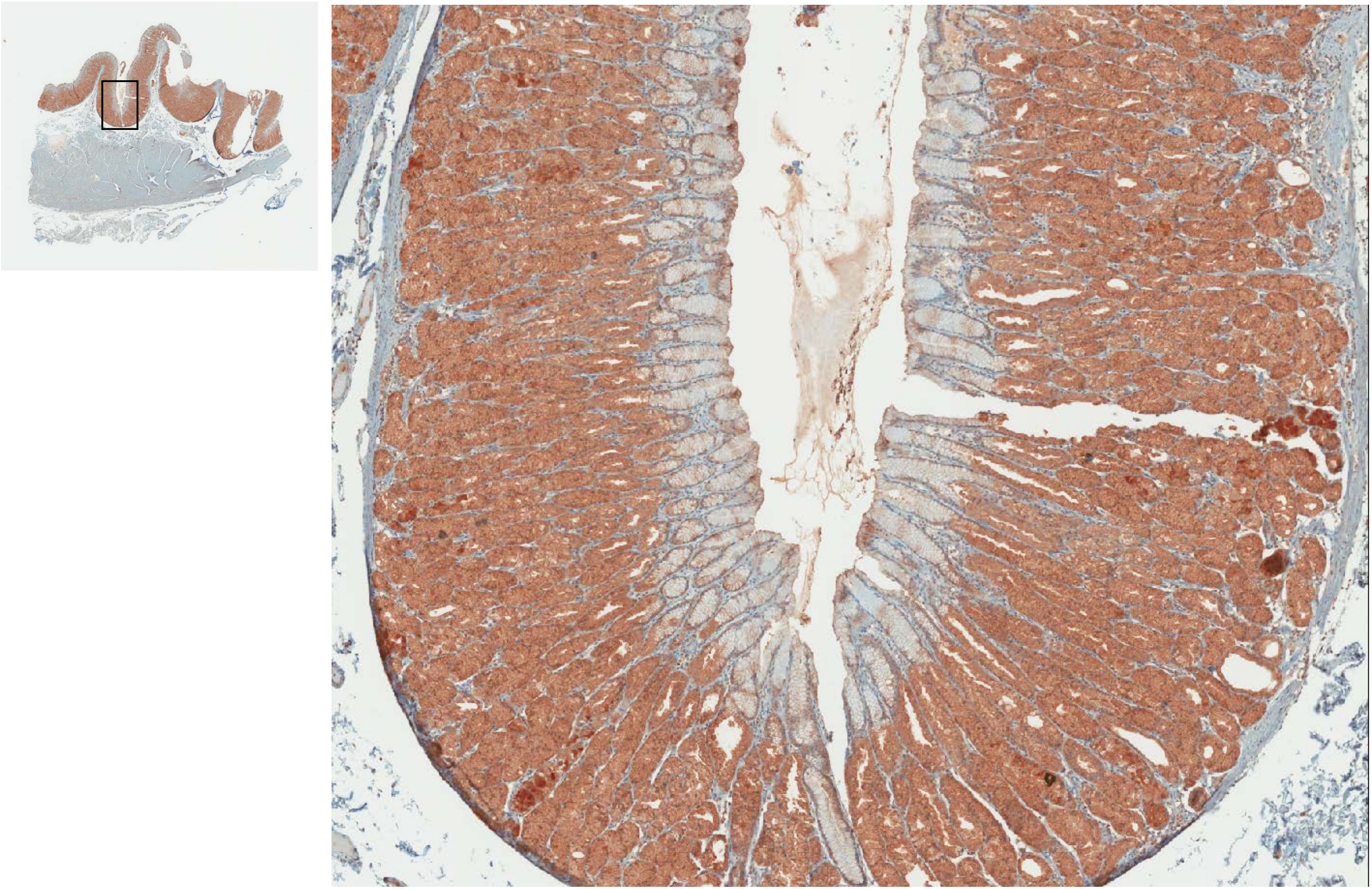

SI Figure 274. Example image of normal stained tissue from stomach. IHC was performed on tissue sections using a monoclonal antibody raised against CCK2R. Inset has a magnification of 4x.

Normal Stomach, inset epithelium pathology total score = 6

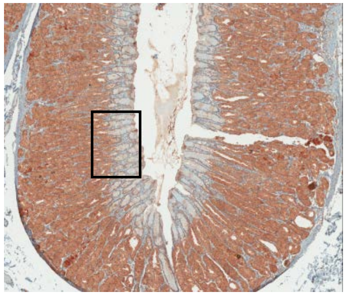

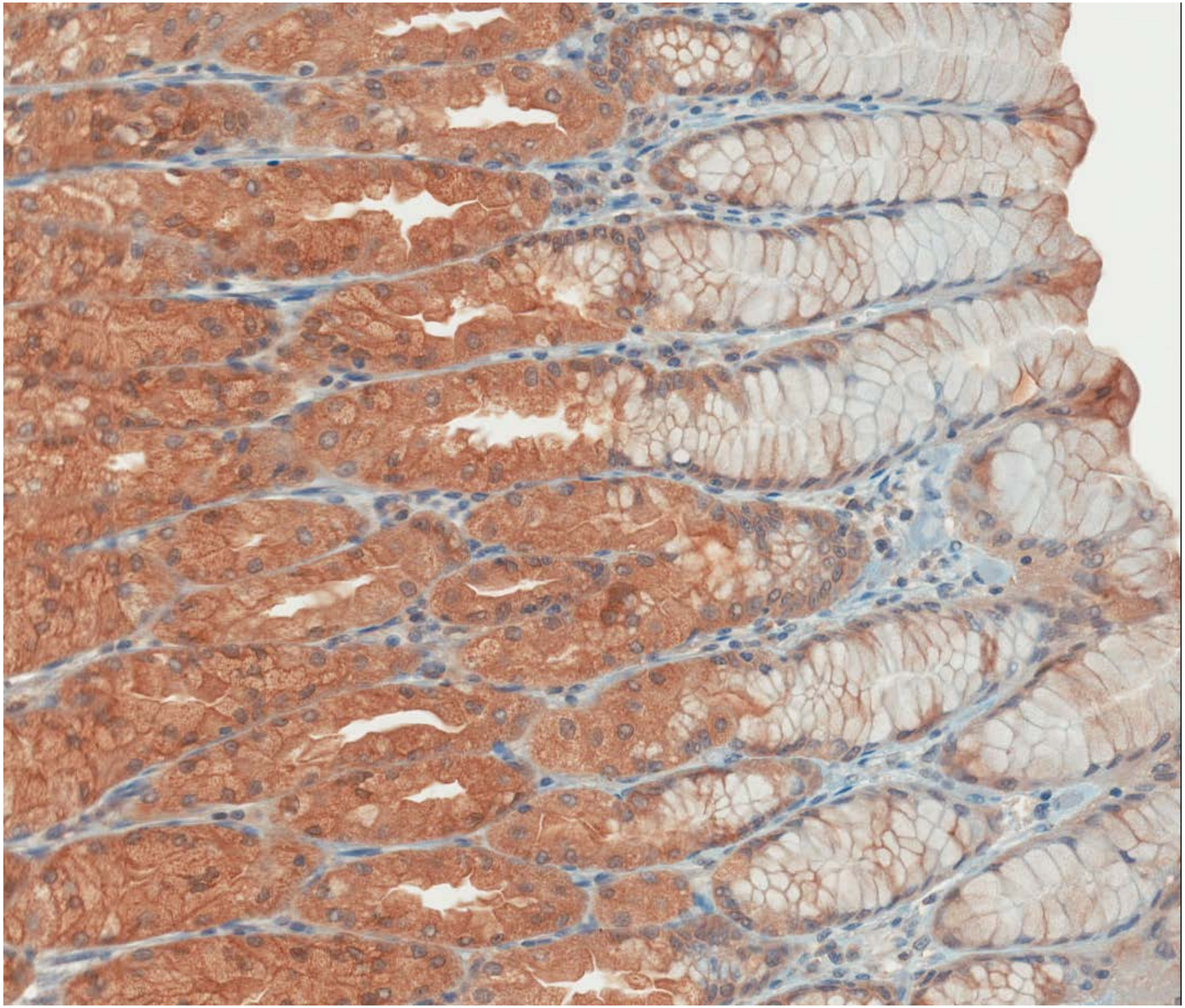

SI Figure 275. Example image of normal stained tissue from stomach. IHC was performed on tissue sections using a monoclonal antibody raised against CCK2R. Inset has a magnification of 20x.

Normal Stomach Tissue Staining Intensity

	n	Average	St. Dev	St. Error
Staining Intensity	3	1.00	0.00	0.00

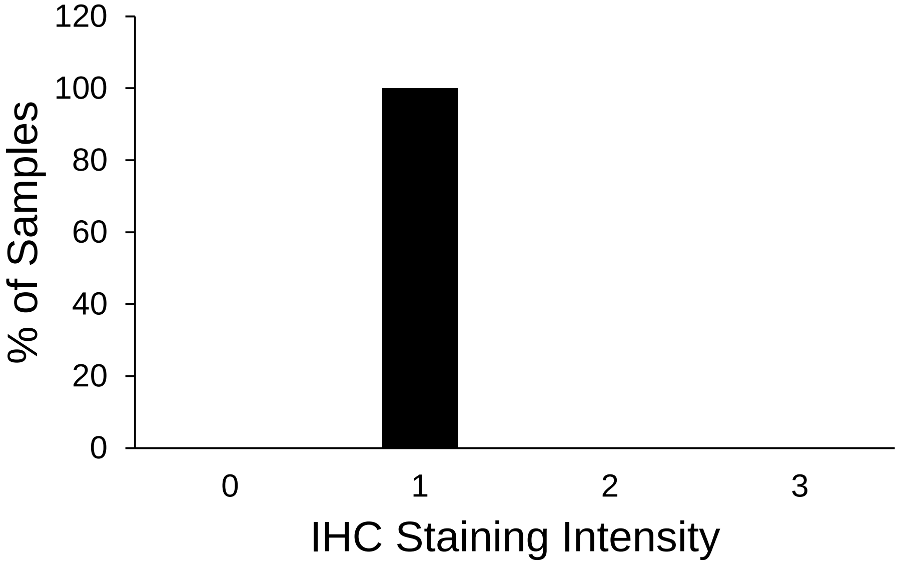

	Staining Intensity			
	0	1	2	3
n	0	3	0	0
%	0.00	100.00	0.00	0.00

SI Figure 276. CCK2R Staining Intensity for normal tissue from the stomach. IHC was performed on tissue sections using a monoclonal antibody raised against CCK2R. The intensity of staining was graded on a scale of 0 to 3 and plotted.

Normal Stomach Tissue Coverage Score Correlations

	n	Average	St. Dev	St. Error
Coverage Score	3	2.33	0.58	0.33

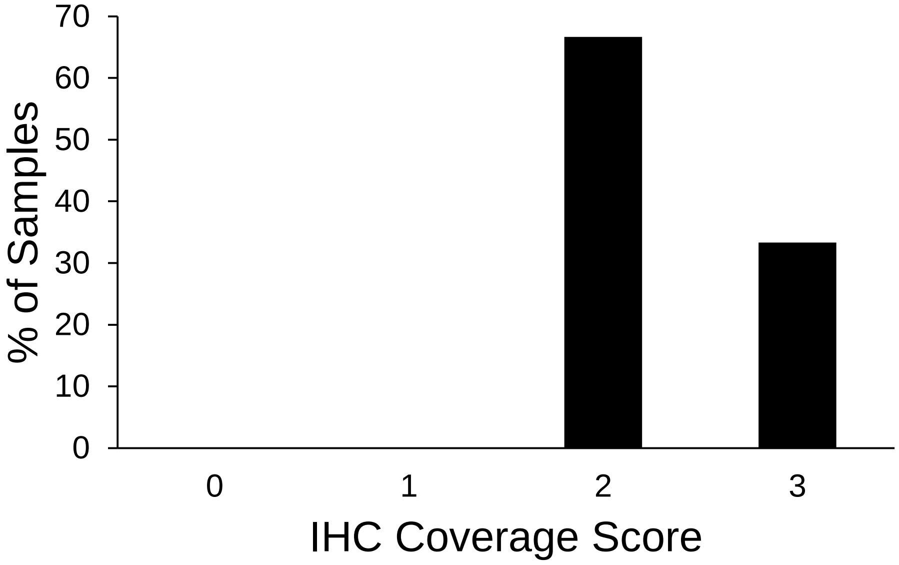

	Coverage Score			
	0	1	2	3
n	0	0	2	1
%	0.00	0.00	66.67	33.33

SI Figure 277. CCK2R Coverage Score for normal tissue from the stomach. IHC was performed on tissue sections using a monoclonal antibody raised against CCK2R. The area stained (coverage) was graded on a scale of 0 to 3 and plotted.

Normal Stomach Tissue
Total Staining Score Correlations

	n	Average	St. Dev	St. Error
Total Score	3	2.33	0.58	0.33

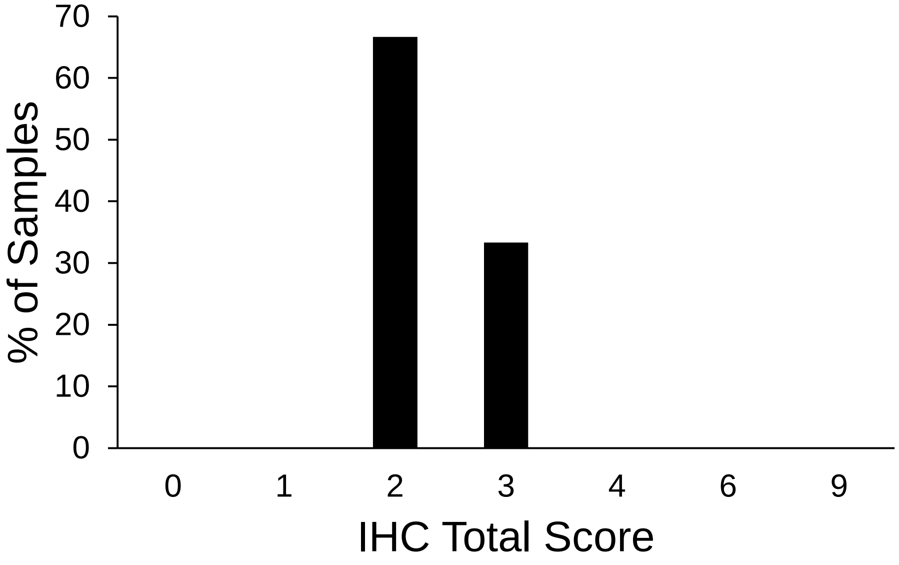

	Total Staining Score						
	0	1	2	3	4	6	9
n	0	0	2	1	0	0	0
%	0.00	0.00	66.67	33.33	0.00	0.00	0.00

SI Figure 278. CCK2R Total Staining Score for normal tissue from the stomach. IHC was performed on tissue sections using a monoclonal antibody raised against CCK2R. The staining intensity and coverage score was multiplied to obtain the total staining score.

Normal Thyroid

Normal Thyroid
Overall Summary

Normal Thyroid - Spearman Correlation

	Sex	Age at Diagnosis
**Staining Intensity**	N.A.	No 0.424
**Coverage Score**	N.A.	No 0..692
**Total Staining Score**	N.A.	No 0.977

Normal Thyroid - ANOVA/t-test

	Sex	Age at Diagnosis
**Staining Intensity**	No 0.107	No 0.246
**Coverage Score**	No 0.281	No 0.997
**Total Staining Score**	No 0.073	No 0.467

SI Figure 279. Correlation summary of CCK2R in normal thyroid. IHC was performed on normal thyroid tissue sections using a monoclonal antibody raised against CCK2R. The staining intensity, coverage score and total staining score were compared against available patient data. If appropriate, a spearman analysis was used to determine if any significant correlation exists while a 1-way ANOVA or t-test was used to determine if a significant difference exists between groups. Whether the test was statistically significant and the p-value is listed. N.A. – not applicable (this statistical test was not applicable to this data set). N.D. – not determined (this statistical test could not be performed, generally due to a lack of the number of samples within a group or all data was in a single group).

Normal Thyroid Tissue Staining Intensity

	n	Average	St. Dev	St. Error
Staining Intensity	28	1.07	0.26	0.05

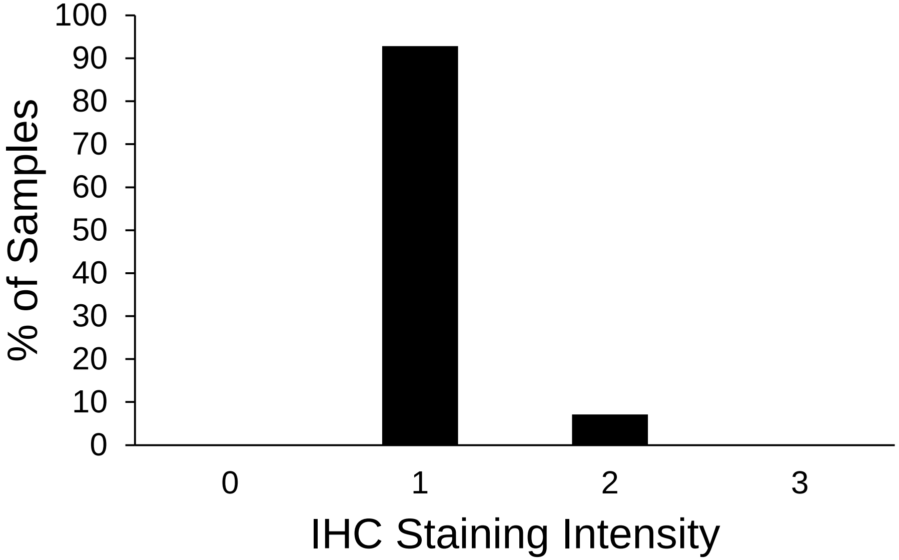

	0	1	2	3
n	0	26	2	0
%	0.00	92.86	7.14	0.00

SI Figure 280. CCK2R Staining Intensity for normal tissue from the thyroid. IHC was performed on tissue sections using a monoclonal antibody raised against CCK2R. The intensity of staining was graded on a scale of 0 to 3 and plotted.

Normal Thyroid - Staining Intensity

	Sex	Age at Diagnosis
**Spearman Correlation**	N.A.	No<br>0.424
**ANOVA/t-test**	No<br>0.107	No<br>0.246

SI Figure 281. Staining intensity correlation summary of CCK2R in normal thyroid. IHC was performed on normal thyroid tissue sections using a monoclonal antibody raised against CCK2R. The staining intensity was compared against available patient data. If appropriate, a spearman analysis was used to determine if any significant correlation exists while a 1-way ANOVA or t-test was used to determine if a significant difference exists between groups. Whether the test was statistically significant and the p-value is listed. N.A. – not applicable (this statistical test was not applicable to this data set). N.D. – not determined (this statistical test could not be performed, generally due to a lack of the number of samples within a group or all data was in a single group).

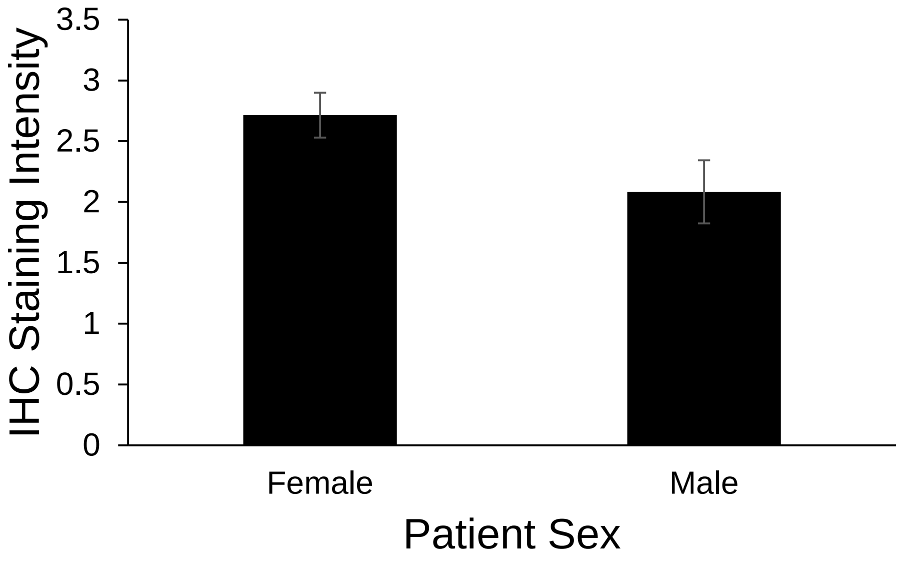

Spearman Analysis

ρ (rho) = -

t = -

p-value = -

	n	Average	St. Dev	St. Error
Female	7	2.71	0.49	0.18
Male	12	2.08	0.90	0.26

t-test

p-value = 0.107

SI Figure 282. Correlation analysis of CCK2R staining intensity in normal thyroid versus patient sex. IHC was performed on normal thyroid tissue sections using a monoclonal antibody raised against CCK2R. The staining intensity was graded on a scale of 0 to 3 and plotted (error bars represent standard error of the mean). A t-test was used to determine if there were any significant differences between groups.

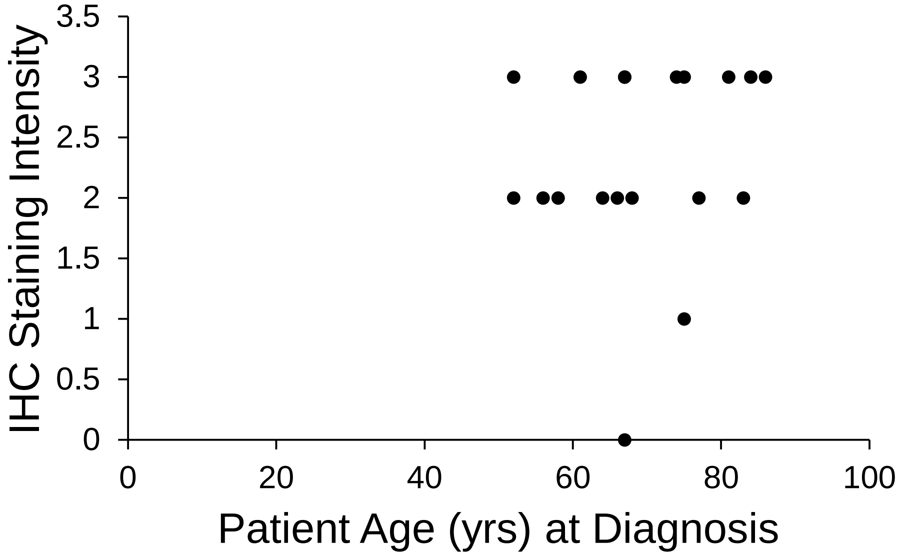

Spearman Analysis

ρ (rho) = 0.1946
t = 0.8180
p-value = 0.4240

	n	Average	St. Dev	St. Error
0	1	67.00	-	-
1	1	75.00	-	-
2	8	65.50	10.53	2.11
3	9	71.89	11.21	2.24

t-test

p-value = 0.2462

SI Figure 283. Correlation analysis of CCK2R staining intensity in normal thyroid versus patient age at diagnosis. IHC was performed on normal thyroid tissue sections using a monoclonal antibody raised against CCK2R. The staining intensity was graded on a scale of 0 to 3 and plotted. A Spearman analysis was used to determine if there was a statistically significant correlation and a t-test was used to determine if there were any significant differences between groups.

Normal Thyroid Tissue
Coverage Score Correlations

	n	Average	St. Dev	St. Error
Coverage Score	28	2.96	0.19	0.04

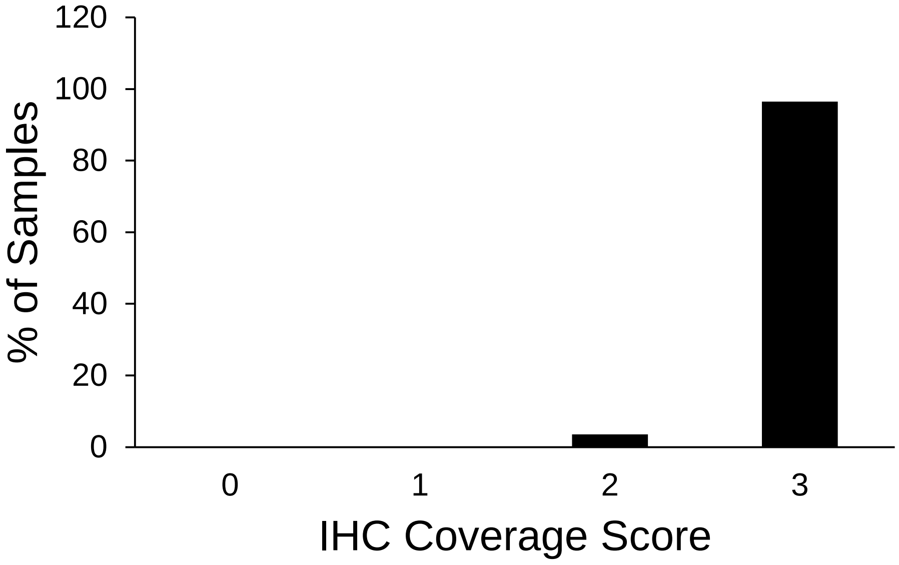

	Coverage Score			
	0	1	2	3
n	0	0	1	27
%	0.00	0.00	3.57	96.43

SI Figure 284. CCK2R Coverage Score for normal tissue from the thyroid. IHC was performed on tissue sections using a monoclonal antibody raised against CCK2R. The area stained (coverage) was graded on a scale of 0 to 3 and plotted.

Normal Thyroid - Coverage Score

	**Sex**	**Age at Diagnosis**
**Spearman Correlation**	N.A.	No<br>0..692
**ANOVA/t-test**	No<br>0.281	No<br>0.997

SI Figure 285. Coverage Score correlation summary of CCK2R in normal thyroid. IHC was performed on normal thyroid tissue sections using a monoclonal antibody raised against CCK2R. The coverage score was compared against available patient data. If appropriate, a spearman analysis was used to determine if any significant correlation exists while a 1-way ANOVA or t-test was used to determine if a significant difference exists between groups. Whether the test was statistically significant and the p-value is listed. N.A. – not applicable (this statistical test was not applicable to this data set). N.D. – not determined (this statistical test could not be performed, generally due to a lack of the number of samples within a group or all data was in a single group).

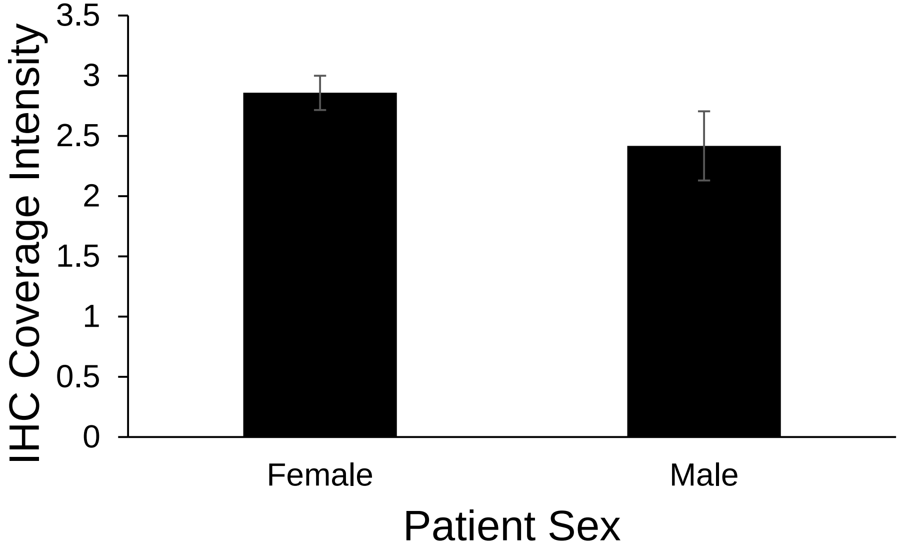

Spearman Analysis		
	ρ (rho) =	-
	t =	-
	p-value =	-

	n	Average	St. Dev	St. Error
Female	7	2.86	0.38	0.14
Male	12	2.42	1.00	0.29

t-test

p-value = 0.2813

SI Figure 286. Correlation analysis of CCK2R coverage score in normal thyroid versus patient sex. IHC was performed on normal thyroid tissue sections using a monoclonal antibody raised against CCK2R. The coverage score was graded on a scale of 0 to 3 and plotted (error bars represent standard error of the mean). A t-test was used to determine if there were any significant differences between groups.

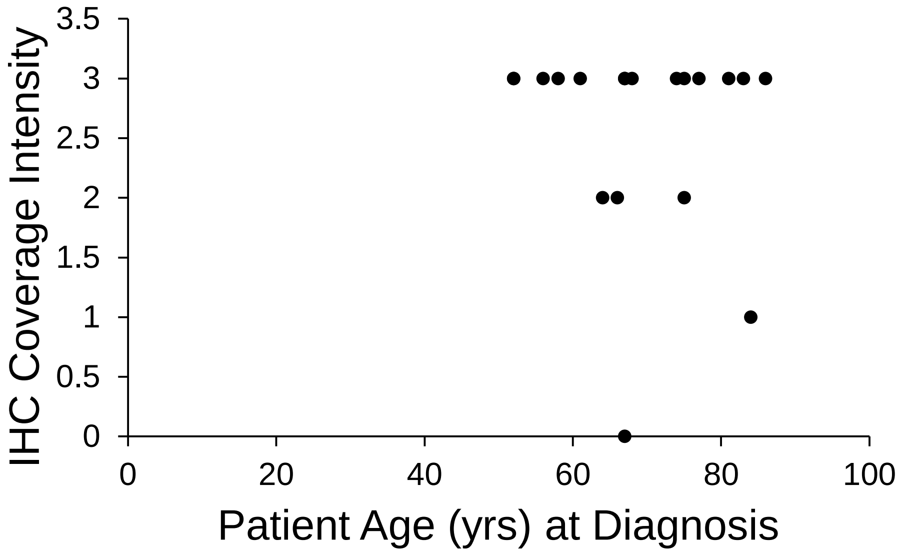

Spearman Analysis

ρ (rho) = -0.0973
t = -0.4031
p-value = 0.6916

	n	Average	St. Dev	St. Error
0	1	67.00	-	-
1	1	84.00	-	-
2	3	68.33	5.86	3.38
3	14	68.36	11.40	3.05

t-test

p-value = 0.9973

SI Figure 287. Correlation analysis of CCK2R coverage score in normal thyroid versus patient age at diagnosis. IHC was performed on normal thyroid tissue sections using a monoclonal antibody raised against CCK2R. The coverage score was graded on a scale of 0 to 3 and plotted. A Spearman analysis was used to determine if there was a statistically significant correlation and a t-test was used to determine if there were any significant differences between groups.

Normal Thyroid Tissue
Total Staining Score Correlations

	n	Average	St. Dev	St. Error
Total Score	28	3.18	0.82	0.15

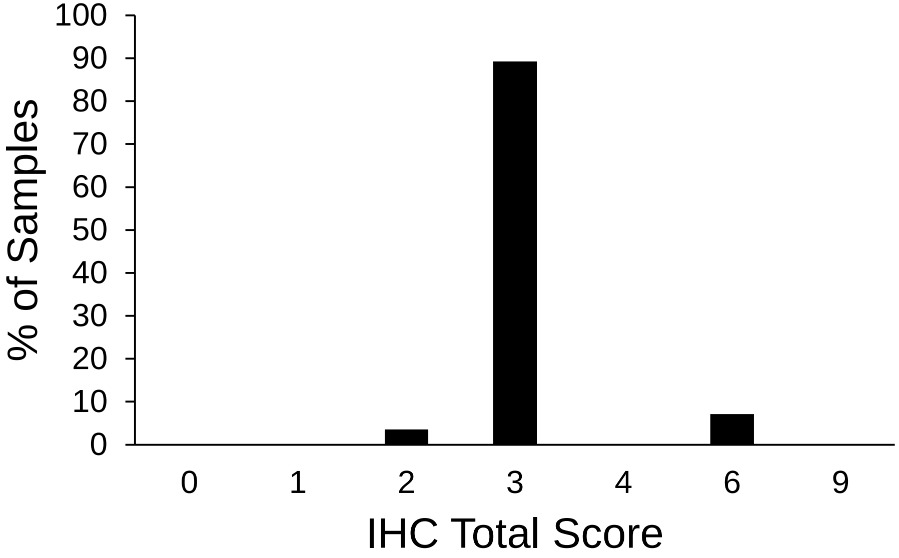

	Total Staining Score						
	0	1	2	3	4	6	9
n	0	0	1	25	0	2	0
%	0.00	0.00	3.57	89.29	0.00	7.14	0.00

SI Figure 288. CCK2R Total Staining Score for normal tissue from the thyroid. IHC was performed on tissue sections using a monoclonal antibody raised against CCK2R. The staining intensity and coverage score was multiplied to obtain the total staining score.

Normal Thyroid - Total Staining Score

	Sex	Age at Diagnosis
**Spearman Correlation**	N.A.	No<br>0.977
**ANOVA/t-test**	No<br>0.073	No<br>0.467

SI Figure 289. Total staining score correlation summary of CCK2R in normal thyroid. IHC was performed on normal thyroid tissue sections using a monoclonal antibody raised against CCK2R. The total staining score was compared against available patient data. If appropriate, a spearman analysis was used to determine if any significant correlation exists while a 1-way ANOVA or t-test was used to determine if a significant difference exists between groups. Whether the test was statistically significant and the p-value is listed. N.A. – not applicable (this statistical test was not applicable to this data set). N.D. – not determined (this statistical test could not be performed, generally due to a lack of the number of samples within a group or all data was in a single group).

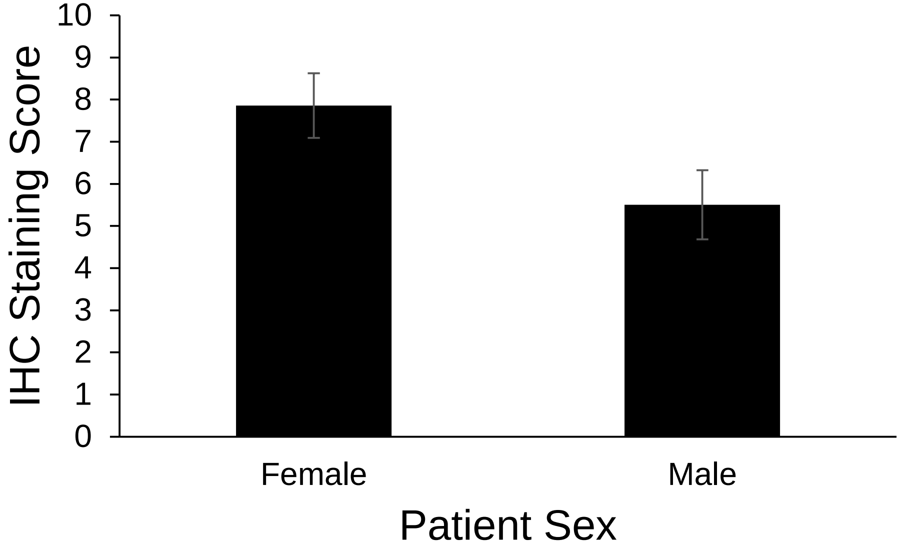

Spearman Analysis		
	ρ (rho) =	-
	t =	-
	p-value =	-

	n	Average	St. Dev	St. Error
Female	7	7.86	2.04	0.77
Male	12	5.50	2.84	0.82

t-test

p-value = 0.0725

SI Figure 290. Correlation analysis of CCK2R total staining score in normal thyroid versus patient sex. IHC was performed on normal thyroid tissue sections using a monoclonal antibody raised against CCK2R. The total staining score was derived by multiplying the staining intensity and the coverage score and plotted (error bars represent standard error of the mean). A t-test was used to determine if there were any significant differences between groups.

Spearman Analysis

ρ (rho) =	0.0070
t =	0.0288
p-value =	0.9774

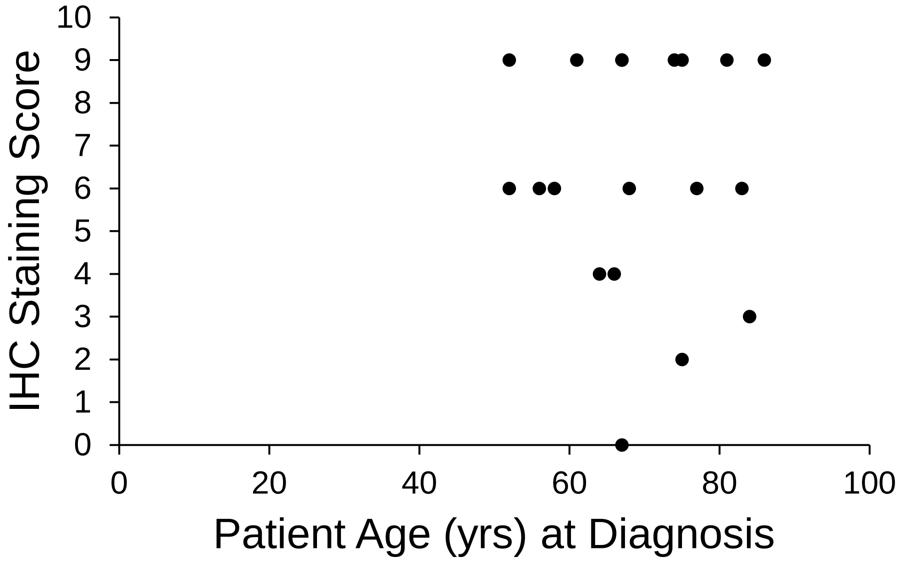

	n	Average	St. Dev	St. Error
0	1	67.00	-	-
1	0	-	-	-
2	1	75.00	-	-
3	1	84.00	-	-
4	2	65.00	1.41	1.00
6	6	65.67	12.44	5.08
9	8	70.38	10.95	3.87

t-test

p-value = 0.4666

SI Figure 291. Correlation analysis of CCK2R total staining score in normal thyroid versus patient age at diagnosis. IHC was performed on normal thyroid tissue sections using a monoclonal antibody raised against CCK2R. The total staining score was derived by multiplying the staining intensity and the coverage score and plotted . A Spearman analysis was used to determine if there was a statistically significant correlation and a t-test was used to determine if there were any significant differences between groups.
